# Synchronous Seasonality in the Gut Microbiota of Wild Mouse Populations

**DOI:** 10.3389/fmicb.2022.809735

**Published:** 2022-04-25

**Authors:** Kirsty J. Marsh, Aura M. Raulo, Marc Brouard, Tanya Troitsky, Holly M. English, Bryony Allen, Rohan Raval, Saudamini Venkatesan, Amy B. Pedersen, Joanne P. Webster, Sarah C. L. Knowles

**Affiliations:** ^1^Department of Pathobiology and Population Sciences, The Royal Veterinary College, University of London, Hatfield, United Kingdom; ^2^College of Life and Environmental Sciences, University of Exeter, Cornwall, United Kingdom; ^3^Department of Zoology, University of Oxford, Oxford, United Kingdom; ^4^Department of Life Sciences, Imperial College London, Silwood Park, Ascot, United Kingdom; ^5^Institute of Evolutionary Biology, School of Biology, University of Edinburgh, Edinburgh, United Kingdom

**Keywords:** Bacteroidales, core, individuality, lab vs. wild, microbiome, mouse, seasonality, 16S

## Abstract

The gut microbiome performs many important functions in mammalian hosts, with community composition shaping its functional role. However, the factors that drive individual microbiota variation in wild animals and to what extent these are predictable or idiosyncratic across populations remains poorly understood. Here, we use a multi-population dataset from a common rodent species (the wood mouse, *Apodemus sylvaticus*), to test whether a consistent “core” gut microbiota is identifiable in this species, and to what extent the predictors of microbiota variation are consistent across populations. Between 2014 and 2018 we used capture-mark-recapture and 16S rRNA profiling to intensively monitor two wild wood mouse populations and their gut microbiota, as well as characterising the microbiota from a laboratory-housed colony of the same species. Although the microbiota was broadly similar at high taxonomic levels, the two wild populations did not share a single bacterial amplicon sequence variant (ASV), despite being only 50km apart. Meanwhile, the laboratory-housed colony shared many ASVs with one of the wild populations from which it is thought to have been founded decades ago. Despite not sharing any ASVs, the two wild populations shared a phylogenetically more similar microbiota than either did with the colony, and the factors predicting compositional variation in each wild population were remarkably similar. We identified a strong and consistent pattern of seasonal microbiota restructuring that occurred at both sites, in all years, and within individual mice. While the microbiota was highly individualised, some seasonal convergence occurred in late winter/early spring. These findings reveal highly repeatable seasonal gut microbiota dynamics in multiple populations of this species, despite different taxa being involved. This provides a platform for future work to understand the drivers and functional implications of such predictable seasonal microbiome restructuring, including whether it might provide the host with adaptive seasonal phenotypic plasticity.

## Introduction

The gastrointestinal tracts of vertebrates harbour complex microbial communities known as the gut microbiota, that can perform a wide range of important functions for the host. These include regulating the immune system (Round and Mazmanian, 2009), extracting nutrients from otherwise indigestible parts of the diet (Flint et al., 2012), and defence against pathogens (Buffie and Pamer, 2013). With such important roles, one might expect these symbiotic communities to be under strong host influence, such that individuals of a given species harbour a characteristic and relatively invariant community. Yet the emerging picture from microbiome research firmly contradicts this. Vertebrate gut microbiotas are typified by immense compositional variation, both among individuals and within individuals over time. Each individual’s gut microbiota constitutes a diverse community of microbes shaped by environmental influences such as diet, habitat and xenobiotics, microbial interactions, metacommunity-level processes such as transmission among hosts, and mechanisms of host selection, arising, for example, through physiological differences among host genotypes or age. The relative importance of these different ecological processes in shaping wild animal microbiomes remains a key open question.

In humans, cross-population microbiome comparisons have shown that only a small set of “core” gut bacteria are consistently detected across individuals within a population, and fewer are shared across populations (Tap et al., 2009; Qin et al., 2010; Li et al., 2013; Falony et al., 2016). Rather, the human gut microbiota is highly individualised, with each person harbouring a characteristic microbial fingerprint which is distinct from that of others over time (Turnbaugh and Gordon, 2009; Faith et al., 2013; Bergström et al., 2014; De Muinck and Trosvik, 2018). However, given the unique ecology of industrialised humans, whether such patterns apply to non-human wild vertebrates remains poorly understood. Cross-population studies in the wild examining to what extent taxa are shared by conspecifics across geographical space, and at what scale this might occur, are rare (Lankau et al., 2012; Linnenbrink et al., 2013; Smith et al., 2015; Grieneisen et al., 2019; Funosas et al., 2021). Moreover, most wild animal microbiota studies are cross-sectional or group-level, with few that have characterised the microbiota of repeat-sampled individuals. Such longitudinal data are crucial for understanding microbiota stability at both the individual and population level, and how this may be shaped by fluctuations in the environment.

A growing number of studies have revealed seasonal dynamics in the gut microbiota of wild animals (Kobayashi et al., 2006; Williams et al., 2013; Amato et al., 2014a; Fogel, 2015; Maurice et al., 2015; Sun et al., 2016; Ren et al., 2017; Springer et al., 2017; Liu et al., 2019; Orkin et al., 2019). Pronounced seasonal microbiota dynamics have also been observed among humans leading traditional, hunter-gatherer lifestyles (Davenport et al., 2014; Smits et al., 2017). These findings contrast with those from industrialised humans, where microbiota composition is considered relatively stable in adulthood (Faith et al., 2013). The causes of such seasonal dynamics are not fully understood, but existing studies have implicated seasonal shifts in diet (Amato et al., 2014a; Ren et al., 2017), or hibernation (Carey et al., 2013; Sommer et al., 2016). However, in many studies the fine-scale seasonal dynamics are not well-understood as temporal resolution is relatively course (e.g., winter vs. summer), and the extent to which seasonal dynamics are idiosyncratic or repeatable across years or populations of a species remains unknown. Understanding the repeatability of such seasonal dynamics across time and space is a key step towards understanding their potential functional significance. If such temporal dynamics are general across populations and years, this would suggest a broadly applicable underlying process (for example, a predictable environmental or intrinsic host seasonal change), and allow for the possibility that such changes could provide the host with repeatable adaptive seasonal plasticity (Amato et al., 2014a; Alberdi et al., 2016).

Here, we present a longitudinal analysis of gut microbiota dynamics in two wild wood mouse (*Apodemus sylvaticus*) populations, together with a comparison from a wild-derived captive colony of the same species. Wood mice are an ideal study system for questions about wild gut microbiome dynamics, as they are common and amenable to capture-mark-recapture protocols with accurate individual identification. Furthermore, a previous study on wood mice in the United Kingdom described seasonal shifts in gut microbiota composition, occurring between summer and autumn (Maurice et al., 2015). Here, we provide a comprehensive analysis of variation in gut microbial community structure and the factors associated with this variation operating at three levels of organisation: across host populations, among individuals within a population and within individuals over time. We predict that (i) gut communities will be more similar within a host population than between host populations, but that there will be a small set of “core” taxa consistently identified across hosts, (ii) environmental drivers of variation will be stronger than host-related factors, (iii) seasonal restructuring of the gut microbiota will be consistent across two wild United Kingdom populations living in woodland habitats, and (iv) microbiota composition will be repeatable within individuals sampled over time, but the relative strength of inter-vs. intra-individual variation may fluctuate throughout the year.

## Materials and Methods

### Sample Collection

Rodents were live-trapped using a capture-mark-recapture study design in two mixed deciduous woodlands approximately 50 km apart in the United Kingdom: Wytham Woods, Oxfordshire (“Wytham”, 51°46’N,1°20’W) and Silwood Park, Berkshire (“Silwood”, 51°24’N, 0°38’W). Fieldwork methods were consistent across sites, with trapping performed for one night every 2–4 weeks across all seasons. In Silwood, trapping occurred on a single 2.47 ha grid over a 1-year period (November 2014 to December 2015), while in Wytham trapping took place over a 3-year period (October 2015–2018), initially on a 1 ha grid that was then expanded to 2.4 ha in October 2017. Wood mice (*A. sylvaticus*) were the most commonly caught rodent at both sites, with yellow-necked mice (*Apodemus flavicollis*) and bank voles (*Myodes glareolus*) also captured. Small Sherman live traps were baited with six peanuts and a slice of apple, with sterile non-absorbent cotton wool provided for insulation. Traps were set in alternate 10 m × 10 m grid cells at dusk and collected at dawn. All newly captured rodents were injected subcutaneously with a PIT tag for identification purposes, with ear notches used as a back-up form of identification. The following information was recorded for all captures: species, ID, sex, age (juvenile, sub-adult or adult, according to size and pelage characteristics), reproductive state (active or inactive according to signs of reproduction such as testes size in males, perforation, pregnancy or lactation in females), body mass (to the nearest 0.1 g), subcutaneous fat score (0–4, measured by palpating the lower spine and hips). All animals were released within the 10 m × 10 m grid cell of their capture. Faecal samples (approximately 40–300 mg) were collected from the bedding material with sterilised tweezers, and frozen at −80°C within 10 h of trap collection for molecular work. Traps that showed any sign of animal contact (both traps that held captured animals and those where an animal had interfered with but not triggered it) were washed thoroughly with 20% bleach solution between trapping sessions to prevent cross-contamination. Live-trapping work was conducted with institutional ethical approval, and at Wytham under Home Office licence PPL-I4C48848E.

Faecal samples were also collected from an outbred, captive colony of wood mice housed at the University of Edinburgh, and included for comparison to wild populations. This colony is thought to have been established over 30 years ago at the University of Oxford (Department of Zoology) using wild caught mice from the local area (presumed to be Wytham Woods, where department members were trapping rodents at the time, though this has not been confirmed). Since this time, the colony has been bred for several decades, has been housed at several different locations, and may have been expanded with wild-caught mice from other locations in the United Kingdom (Hughes et al., 2010). Captive wood mice included in this study were sampled as part of a diet shift experiment carried out in 2017 under Home Office Project license 70/8543. Further details about the colony and experiment are given in the Supporting Information.

### 16S rRNA Gene Sequencing

Genomic DNA was extracted from faecal samples using Zymo Quick-DNA faecal/soil Microbe 96 (96-well plate format) extraction kits, according to manufacturer instructions. A 254 bp region of the bacterial 16S rRNA gene (V4 region) was amplified using primers 515F/806R (Caporaso et al., 2011; Supplementary Table S1). Library preparations followed a two-step (tailed-tag) approach with dual-indexing (D’Amore et al., 2016) and sequencing was performed on an Illumina® MiSeq with 250 bp paired-end reads, at the Centre for Genomic Research in Liverpool. Samples were extracted in 17 batches and sequenced in five lanes, with some Wytham and colony samples extracted and sequenced together, while samples from Silwood were extracted and sequenced separately with minor adjustments to the protocol (Supporting Information). To avoid further batch effects influencing results, wild samples from each trapping session and colony samples from each timepoint and animal were evenly distributed across extraction plates. Full details of 16S rRNA sequencing methods are provided in Supporting Information.

### Bioinformatics

Raw sequence data were processed through the DADA2 pipeline (v1.6) in R to infer amplicon sequence variants (ASVs; Callahan et al., 2016, 2017). This was done separately for each Miseq run, but using identical parameters. In brief, reads were trimmed and filtered for quality, ASVs inferred and putative chimeras removed before taxonom assignment using the v128 SILVA reference database. Full details of the bioinformatics pipeline are in Supporting Information. A single *phyloseq* object (McMurdie and Holmes, 2013) containing data from all three populations was created for further processing and analyses. ASVs taxonomically assigned as chloroplast or mitochondria were removed, after which the dataset contained 11,404 ASVs. The R package “iNEXT” (Chao et al., 2014; Hsieh et al., 2016) was used to examine sample completeness and rarefaction curves, which showed that sample completeness plateaued at approximately 8,000 reads (Supplementary Figure S1). Thirty-one samples with read counts below this threshold were removed, leaving data from 1,052 samples and 281 mice overall (Wytham: *n* = 448 samples from 178 individuals; Silwood: *n* = 253 from 75 individuals; captive colony: *n* = 351 from 28 individuals), with a mean read count of 40,478 (range 8,841–150,932). For beta diversity analyses, further taxon filtering was performed by retaining only those ASVs with more than one copy in at least 1% of samples, to guard against the influence of potential PCR or sequencing artefacts, or contaminants. After this additional ASV filtering step, the dataset contained 2,662 ASVs.

### Statistical Analyses

All R code for the analyses presented here can be found in the online Supplementary Material (Supplementary File 2; R Core Team, 2021).

#### Defining Core Taxa

To delimit core taxa, we estimated each ASV’s population-wide prevalence as the proportion of samples in which it was detected, in a dataset containing one randomly selected sample per individual, averaged over 100 iterations. We also estimated each ASV’s within-individual persistence, as the mean proportion of samples per repeat-sampled individual it was detected in, using data from mice sampled at least three times (*n* = 57 mice in Wytham, mean N captures 4.86 ± 1.94 s.d., *n* = 39 mice in Silwood, mean N captures = 5.95 ± 1.95 s.d.).

#### Alpha-Diversity Analyses

ASV richness was estimated using the R package “breakaway” (Willis and Bunge, 2015), using a dataset for which taxon filtering was limited to the removal of ASVs assigned as chloroplast or mitochondria.

#### Beta-Diversity Analyses

ASV read counts were normalised to relative abundance per sample before calculating pairwise dissimilarity indices among samples (Bray–Curtis dissimilarity; McKnight et al., 2019), which were subsequently used in population-specific principal coordinates analysis (PCoA) and permutational analyses of variance (PERMANOVA). Weighted and unweighted UniFrac distances were used to assess the phylogenetic relatedness of the gut microbiota among host populations. To assess the decay of within-individual community similarity over time (time-decay), we modelled the log-linear change in community structure (pairwise Bray–Curtis dissimilarity) as a function of the number of days between samples. For this, community dissimilarities were converted to similarities (1-dissimilarity) and log-transformed. The rate of change at different time lags was calculated by dividing the Bray–Curtis dissimilarity by the time between sampling points to obtain an average rate of change for each time lag (Faith et al., 2013; Shade et al., 2013). To compare inter- and intra-individual variation in gut microbiota composition, pairwise dissimilarity values were compared across different types of sample pair, using Wilcoxon tests with 1,000 permutations: those collected from the same individual at different timepoints (“Same mouse”) and those collected from different individuals within the same trapping session (“Same date”).

To analyse temporal change in microbiota composition, generalised additive mixed models (GAMM) were run in package “mgcv”, using sample scores along the first axis of a Bray-Curtis PCoA (PC1) as the response variable. A cyclic cubic spline was fitted for day of the year, fitting an interaction with sampling year (October 2015–2016, 2016–2017, and 2017–2018) to model within-year seasonal patterns. A number of sample-level host and methodological terms were included as covariates (age, sex, reproductive status, body mass, body condition, and read count). MiSeq run was also fitted as a covariate in analyses for Wytham. Animal ID was included as a random factor to control for repeated measures. The same model structure was used to assess seasonal patterns in microbial richness.

To test the relative effects of host, environmental and methodological factors on variation in gut community structure, a marginal PERMANOVA was performed using the *adonis2* function in package “vegan” (Oksanen et al., 2019) with 999 permutations, on a subset of data including one randomly chosen sample per individual, to avoid pseudoreplication. Subsequent tests for homogeneity of variance between factor levels of significant terms were performed using the *betadisper* function. Since subsetting to one sample per individual decreases sample size and power, we also performed a partial redundancy analysis (pRDA) on Hellinger-transformed community data, using the same explanatory terms as used in the PERMANOVA, but with individual ID used as a condition. The marginal significance of the explanatory terms were then calculated from the reduced model.

To identify bacterial taxa (ASVs) involved in detected trends, we used Random Forest models with default parameters (package “randomForest”). “IncNodePurity” was used as a measure of ASV importance.

## Results

### Assessing Core Microbes Across Populations

**Figure 1:**
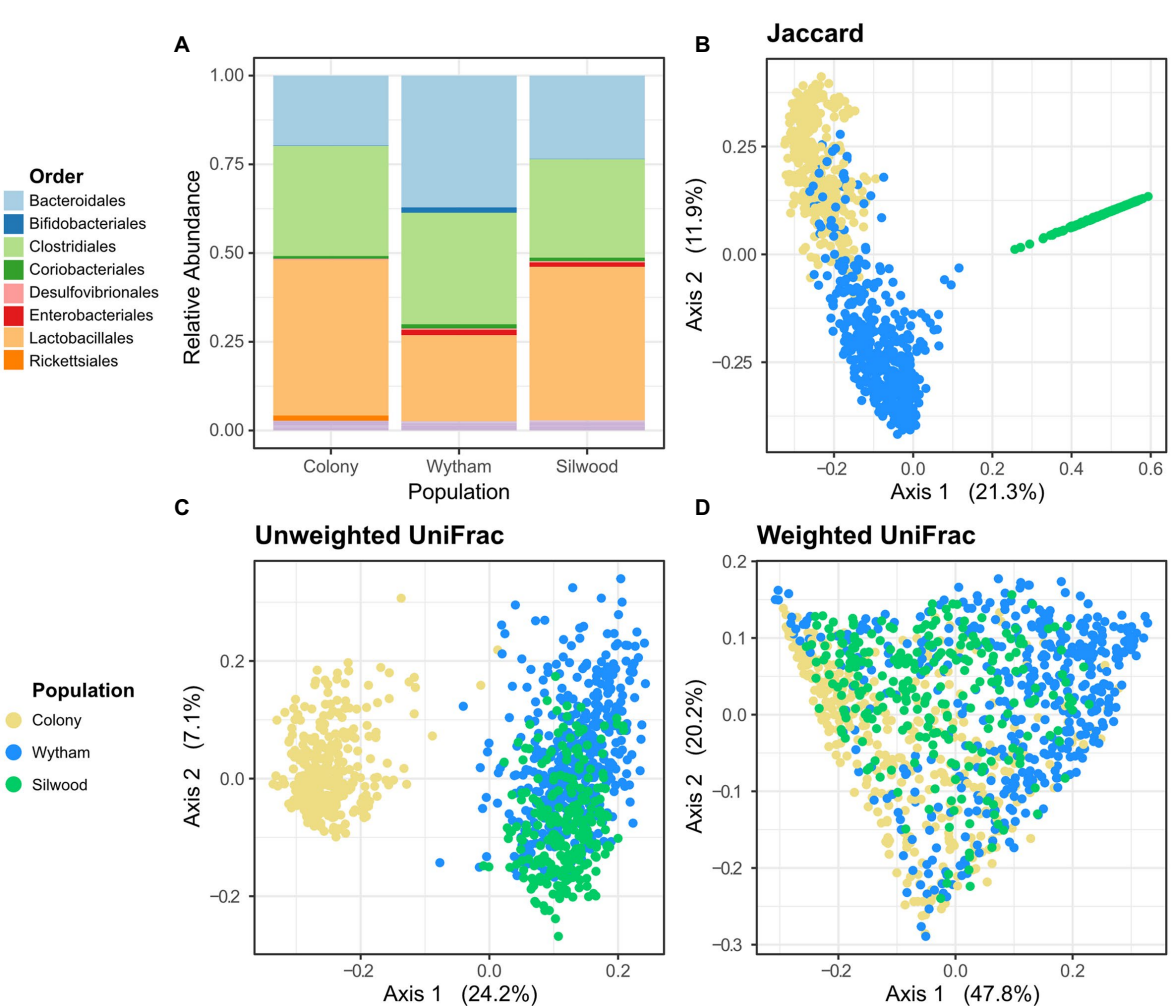
Gut microbiota composition across populations of wood mice. **(A)** Comparison of microbiota composition at the Order level in faecal samples from a captive colony (*n* = 351) and two wild populations, Wytham (*n* = 448) and Silwood (*n* = 253). Read abundances were summed across samples per population and their relative proportions are coloured by bacterial Order. Samples from all populations were used in principal coordinates analysis based on **(B)** Jaccard, **(C)** Uniweighted UniFrac, and **(D)** Weighted UniFrac distances.

Across all three (wild and captive) populations, the gut microbiota of wood mice was broadly comparable in terms of proportional abundances of high-level taxa, being dominated by the orders Lactobacillales, Clostridiales and Bacteroidales (Figure 1A). However, a large amount of compositional variation was evident within populations (Supplementary Figure S2). At the order level, Wytham mice had a slightly higher relative abundance of Bacteroidales, while Silwood mice had a slightly higher relative abundance of Lactobacillales. There was a large degree of overlap in taxa shared between populations at the Phylum level, but as taxonomic resolution increased the overlap in shared taxa decreased (Supplementary Figure S3). Nine phyla were detected in total, eight of which in all populations, while Spirochaetes were only found in Wytham and the colony, and Elusimicrobia only in Silwood. The majority (74%) of identified bacterial orders were common to all populations and similarly 62% of identified genera were shared by all three populations, wild and lab (Supplementary Figure S3). Interestingly, Wytham and the colony shared a large number of ASVs (52.1% of Wytham- and 95% of Colony-detected ASVs), while the two wild populations shared no exact ASVs (Figure 1B; Supplementary Figure S3). This finding was unaltered when taxon filtering was minimal and limited to the removal of reads assigned as chloroplast/mitochondria. Differences in the proportion of taxa shared by populations were unaltered when these comparisons were made using samples sequenced in the same vs. different runs (Supplementary Figure S4), indicating they are very unlikely to be an artefact of the fact Silwood samples were sequenced separately. In addition, the proportion of shared ASVs across populations were robust to differences in sample sizes, as bootstrapped subsampling produced similar estimates (Supplementary Table S2). On average, a pair of mice from Wytham and the colony shared 8.4% ASVs (mean Jaccard Index), while the mean proportion of shared ASVs within each population was slightly higher (Wytham = 11.5%, Colony = 23.2%, Silwood = 18.0%). The ASVs common to Wytham and colony mice came from a broad range of bacterial families, representative of those observed in each population (Supplementary Figure S5). Interestingly, the use of phylogenetically-informed community dissimilarity metrics altered patterns of population-level differences. Although Wytham and Silwood shared no exact ASVs, these two wild populations were more similar in composition when using Unweighted UniFrac, while colony mice had a more phylogenetically distinct microbiota (Figure 1C). The phylogenetically distinct ASVs that are differentially present in colony vs wild populations are likely to be rare community members, since incorporating relative abundance information (using Weighted UniFrac) reduced the extent to which colony mice clustered separately from wild mice (Figure 1D).

### Assessing Core Microbes Within Populations

There were no ASVs shared by all samples within each population, i.e., no population-specific “core microbiota”. To explore how we might define a set of “core” microbiota members using a less strict criterion than universal colonisation, we first examined the relationship between ASV prevalence across hosts and persistence within them. In both wild populations, there was a strong positive correlation between the prevalence and persistence of ASVs (Spearman’s rank correlation; Wytham: rho = 0.932, *p* < 0.001, Silwood; rho = 0.946, *p* < 0.001, Supplementary Figure S6). ASVs that were both prevalent and persistent (>50% for each) formed a taxonomically biased subset, being enriched for the order Bacteroidales (and slightly for Lactobacillales) compared to the total set of ASVs in each wild population (Supplementary Figure S6).

Defining core taxa using both prevalence and abundance thresholds (present in at least 60% samples at 0.1% relative abundance or more) identified a similar set of ASVs in each population. In Wytham, this gave a set of 23 core ASVs, belonging to Muribaculaceae (*n* = 16, 69%), Peptococcaceae (*n* = 1), Ruminococcaeae (*n* = 3) and Lactobacillaceae (*n* = 3). Only two of these “core” ASVs could be identified to genus level and belonged to *Lactobacillus* and *Ruminiclostridium 9*, respectively. Using the same core definition, the Silwood population contained 21 core ASVs, belonging largely to the Muribaculaceae (*n* = 11) as well as Ruminococcaceae (*n* = 4), Lactobacillaceae (*n* = 4), Helicobacteraceae (*n* = 1) and Coriobacteraceae (*n* = 1).

### Predictors of Gut Microbiota Composition

In both wild populations, month was the strongest predictor of gut microbiota composition, explaining approximately twice as much variation as all host factors combined in marginal PERMANOVAs (Table 1). Although population age structure varies seasonally (Supplementary Figure S7), the seasonal effect detected here is independent of this, as host age was controlled for in the model (Table 1). Microbiota composition also differed between years in Wytham. Weaker effects of host factors (body mass and reproductive status) were detected, but each in only one of the two populations (Table 1). Dispersion tests indicated that the effect of year may have been influenced by dispersion differences in Wytham while the effect of month may also contain some influence of dispersion in Silwood (Table 1). Results from the pRDA on Bray–Curtis dissimilarity agreed with those from PERMANOVAs, in that month and year strongly predicted gut microbiota variation in both wild populations, while measured host factors did not (Supplementary Table S3). However, the pRDA also revealed that individual ID (as a condition) explained around half the total variation in each dataset (55.2% in Wytham and 48.1% in Silwood).

**Table 1:** Predictors of microbiota composition in two populations of wild mice.

Variable	Wytham				Silwood			
	*df*	*F*	*p*	Partial *R*^2^	*df*	*F*	*p*	Partial *R*^2^
Read count	1	1.338	0.104	0.009	1	0.629	0.895	0.007
MiSeq run	3	1.460	**0.006**	0.030				
Month	11	1.758	**0.001**	0.133	10	1.584	**0.001** ^‡^	0.187
Year	3	2.591	**0.001** ^†^	0.053				
Sex: reproductive status	1	0.840	0.699	0.006	1	0.835	0.628	0.009
Sex	1	0.908	0.598	0.006	1	0.786	0.684	0.009
Reproductive status	1	1.320	0.126	0.009	1	1.731	0.066	0.020
Age	2	1.001	0.428	0.014	2	0.930	0.563	0.022
Body mass	1	1.382	0.065	0.010	1	1.018	0.382	0.012
Body condition	4	0.944	0.642	0.026	4	0.928	0.615	0.044

Results are shown from marginal PERMANOVAs on Bray–Curtis dissimilarity values. One randomly selected sample per individual was included in each model (Wytham; *n* = 128 and Silwood; *n* = 75). Values of *p* < 0.05 are in bold. For significant terms (factors only), tests for multivariate homogeneity of group dispersions were carried out; ^†^ and ^‡^ indicate terms for which dispersion tests indicated significant differences in dispersion among groups, with *p* = 0.003. and *p* = 0.01. respectively. The interaction between sex and reproductive status was fitted in a separate model including this interaction term, results for all other terms are from a model without this interaction term.

### Repeatable Seasonal Restructuring of the Microbiota

**Figure 2:**
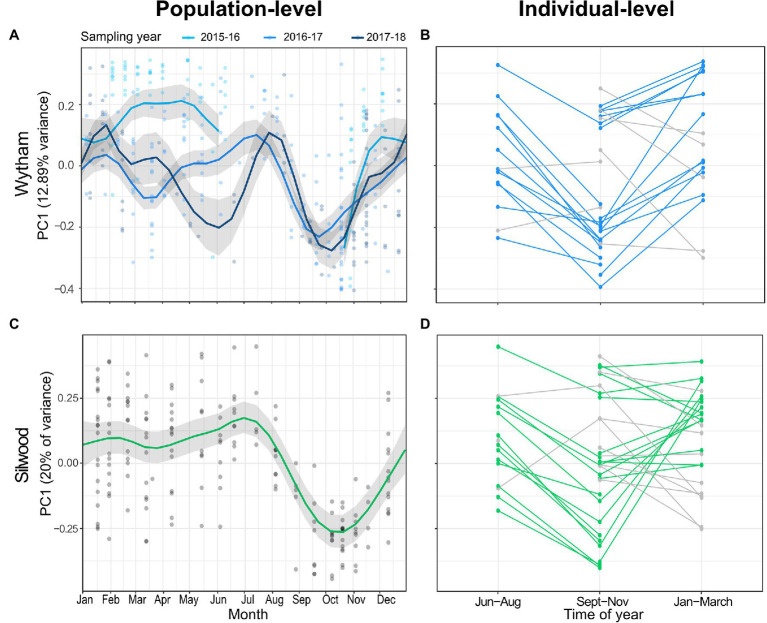
Seasonal restructuring of the wood mouse gut microbiota in two wild populations. **(A,C)** Seasonal dynamics in population-level PC1 value (the position of samples along the first axis of a principal coordinates analysis on Bray–Curtis dissimilarity) in **(A)** Wytham and **(C)** Silwood show compositional change over time. Data from Wytham mice come from a 3-year period (October 2015–2018), while Silwood mice were sampled for 1 year (November 2014–2015). Predicted values and 95% CIs for the smoothed day of the year from generalised additive mixed models (GAMMs) are plotted along with raw PC1 values. **(B,D)** Changes in PC1 within repeat-sampled individual mice typically track the population-level seasonal shifts in both populations (coloured lines), with some exceptions (grey lines).

The major axis of microbiota compositional variation (PC1 from a Bray-Curtis PCoA) fluctuated strongly between July, October and February, and this seasonal pattern was remarkably consistent across populations, explaining 48-55% variance in PC1 (Figures 2A,C; Supplementary Table S4). PC1 was not significantly predicted by any other host or methodological fixed effects apart from MiSeq run (Supplementary Table S4). In both populations, seasonal changes in PC1 within repeat-sampled individuals typically tracked population-level seasonal shifts, with few exceptions (Figures 2B,D; paired *t*-tests for Wytham June-August vs. September-November; *t* = −5.152, *p* < 0.001, *n* = 14, September-November vs. January-March; *t* = 2.676, *p* = 0.015, *n* = 20, for Silwood June-August vs. September-November; *t* = −3.23, *p* = 0.006, *n* = 16, September-November vs. January-March; *t* = 0.250, *p* = 0.805, *n* = 26). Of the few individuals that were not consistent with the population-level patterns, there were no unique or unusual observations in the measured host traits, though repeat captures were too few between these months to formally test factors associated with individual differences in the direction or magnitude of seasonal change in PC1. The pattern of seasonal change in microbiota composition was similar when using PC1 from a PCoA based on Jaccard distance (a presence/absence based distance metric) as the response, although in Silwood the Jaccard-based PC1 explained a lower proportion of gut community variation than Bray-Curtis PC1 (Supplementary Figure S8). This suggests that in Silwood, gut microbiota seasonality consists more of changes in relative abundance than turnover, as can be seen in Sankey plots showing the flux of ASVs between seasons (Supplementary Figure S9).

Despite these prominent seasonal shifts in microbiota composition, the relative abundances of bacterial families did not change dramatically across the year (Supplementary Figure S10). Exploratory analyses also showed that while in Silwood the second and third PCoA axes showed some temporal fluctuations during the same periods that PC1 shifted (oscillation between July and December), in Wytham other PCoA axes besides PC1 did not show strong seasonal variation (Supplementary Figure S11).

Seasonal changes in mean gut microbiota richness were weaker compared to seasonal changes in composition. In models where richness was the response, day of the year was non-significant for Wytham mice, and in Silwood was significant though explained a relatively small amount of variation (14%) compared to similar analyses of composition (Supplementary Figure S12, Wytham: approximate significance of smoothed date term *F* = 0.00, edf < 0.001, *p* = 0.516, adjusted *R*^2^ = 0.09, *n* = 328; Silwood: approximate significance of smoothed date term; *F* = 1.767, edf = 4.722, *p* < 0.001, adjusted *R*^2^ = 0.14, *n* = 185).

**Figure 3:**
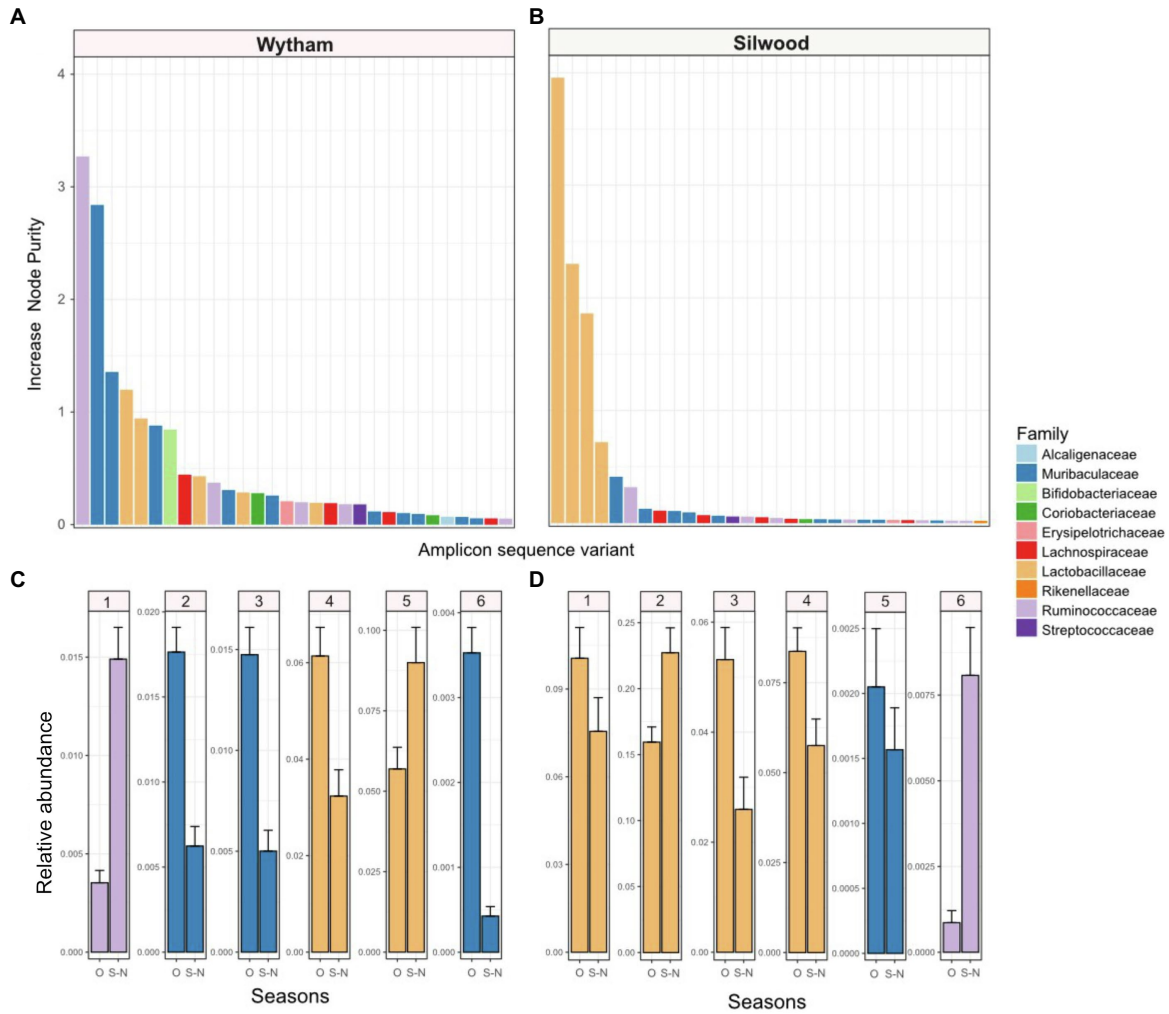
The importance of bacterial taxa in driving consistent seasonal patterns in PC1 (first axis of a Bray-Curtis PCoA) in two wild wood mouse populations. Random forest regressions (RFR) were used to identify bacterial amplicon sequence variants (ASVs) important for predicting PC1, which has a strong seasonal signal, in each population. IncNodePurity” was used as a measure of feature importance in models, and the top 30 ASVs those with the (highest IncNodePurity) are shown for each population **(A)** Wytham and **(B)** Silwood, coloured by the bacterial family they belong to. The relative abundance (mean ± SE) of the top six ASVs in each population, **(C)** Wytham and **(D)** Silwood, are shown in September-November (S-N) compared to all other months (O).

We used random forest regressions to explore which bacterial taxa might drive the consistent seasonal changes in PC1 seen in each wild population. For both populations, the top six ASVs predicting PC1 values belonged to three bacterial families: Lactobacillaceae, Muribaculaceae and Ruminococcaceae, with the exact order of importance for these families differing slightly between populations (Figure 3). In Wytham, the model explained 92.11% of variation in PC1 values, and the most important ASVs predicting PC1 belonged to Ruminococcaceae, followed by Muribaculaceae, Lactobacillaceae, and Bifidobacteriaceae (Figure 3A). In Silwood, the model explained 93.31% in PC1 and all top four ASVs predicting PC1 belonged to Lactobacillaceae, followed by ASVs belonging to Muribaculaceae and Ruminococcaceae (Figure 3B). Although the exact order of importance differed slightly between host populations, the bacterial families of these top six ASVs showed consistent seasonal patterns across the two host populations. Ruminococcaceae ASVs increased in September-November compared to other times of the year, while Muribaculaceae decreased and Lactobacillaceae ASVs showed variable patterns in both populations (Figures 3C,D). The bacterial ASVs most strongly associated with variation along Silwood PC2 and PC3 belonged to Lactobacillaceae, Ruminococcaceae, Lachnospiraceae and Muribaculaceae (Supplementary Figure S13).

### Individuality and Seasonal Convergence in the Gut Microbiota

**Figure 4:**
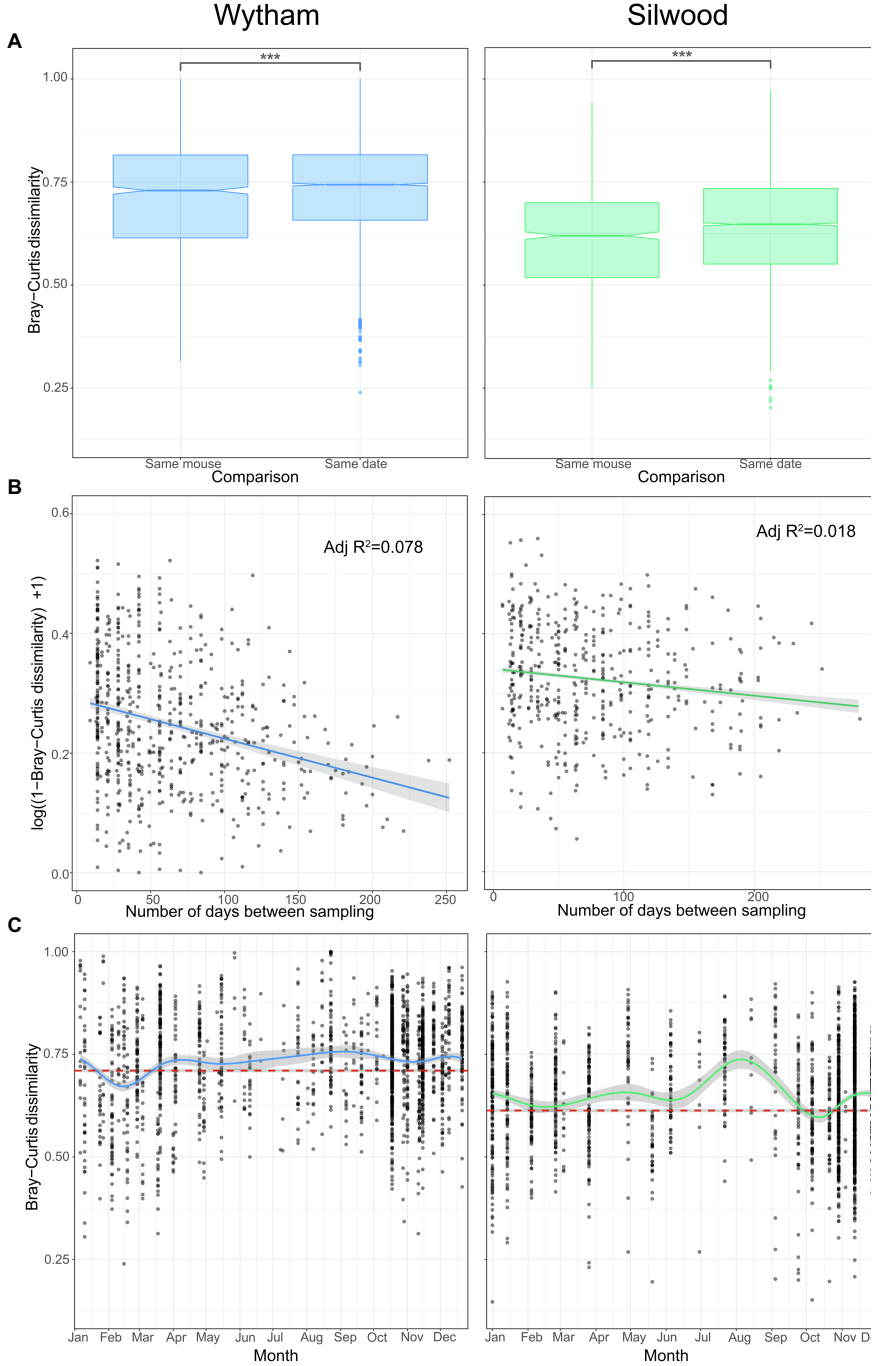
Intra- and inter-individual variation in gut community structure varies with time in two wild populations. **(A)** Pairwise Bray–Curtis dissimilarity between samples from the same host at different times (same mouse) and those taken from different hosts on the same day (same date) were used to compare intra- and inter-individual variation in Wytham (blue) and Silwood (green) wild populations. Significant differences between groups were tested with permutational Wilcoxon tests and are denoted by asterisks (***; *p* < 0.001). **(B)** Intra-individual variation decays with sampling interval. Community similarity (1-Bray–Curtis dissimilarity) between pairs of samples collected from the same individual host (in mice that were captured three or more times, Wytham; *n* = 277 samples from 57 hosts and Silwood; *n* = 197 samples from 39 hosts) are plotted against the sampling interval. Community similarity is log-transformed and the relationship fitted using a log-linear model. **(C)** Inter-individual variation across the year was visualised by plotting the Bray–Curtis dissimilarity between individuals sampled in the same trapping session, with a loess smoothing line for each population. The mean (±SE) intra-individual Bray–Curtis dissimilarity per population is shown as a dashed reference line in red.

Despite strong and repeatable seasonal shifts in average gut microbiota composition (Figure 2), we also identified a large degree of individuality in the microbiota of wild mice. In each wild population, individuals were, on average, more similar in gut microbiota composition (Bray–Curtis dissimilarity) to themselves at other time-points than to other mice sampled on the same day (Figure 4A; permutation tests; Wytham observed U-statistic = 4,222,156, *p* < 0.001; Silwood observed U-statistic = 1,802,548, *p* < 0.001). This signal of individuality decayed with increasing sampling interval (Figure 4B; log-linear model for Wytham; *F* = 107.6_(1268)_, *p* < 0.001, adjusted *R*^2^ = 0.078, Silwood; *F* = 17.51_(1,918)_, *p* < 0.001, adjusted *R*^2^ = 0.018), with the rate of decay highest at short time intervals and less strong as the sampling interval increased (Supplementary Table S5).

Although there was a strong signal of individuality overall (intra-individual variation is on average lower than inter-individual variation), this does not account for temporal dynamics in community composition. We therefore also assessed seasonal changes in inter-individual variation (Bray–Curtis dissimilarity) to determine whether the signal of individuality remains strong throughout the year. In general, there tended to be more convergent gut microbiotas between individuals in winter but more inter-individual variation in the summer months (Figure 4C). Inter-individual variation remained higher than the average within-individual variation for most of the year (Figure 4C), showing that the signal of individuality remains consistently strong. However, in Wytham, the seasonal convergence of gut microbiota composition seen in late winter/early spring was sufficiently strong to briefly override the signal of individuality (Figure 4C); mice sampled in February were more similar to each other than they were to themselves at other times of the year (permutation test; observed U-statistic = 223,084, *p* < 0.001). A weaker convergence was observed in the Silwood population at this same time of year (February), which nullified the signal of individuality, i.e., mice caught at this time were no more similar to themselves across the year than they were to others sampled simultaneously (permutation test; observed U-statistic = 33,916, *p* = 0.712). This reduction in inter-individual variation was observed again in October in Silwood (permutation test; observed U-statistic = 30,240, *p* = 0.046) but not in Wytham (Figure 4C).

## Discussion

Here we provide an in-depth examination of natural variation in the gut microbiota of a wild mammalian host, the wood mouse, using detailed longitudinal analyses from multiple populations and years. We found no universal core microbial community (shared by all members of the species) when considering bacterial ASVs, genera or families across all populations. The proportion of bacterial taxa shared between host populations decreased with increasing taxonomic. At the finest resolution of ASVs there was a high degree of population-specificity in the taxa identified, suggesting high turnover in the species/strains present even between populations ~50 km apart. Geographic variation in gut microbial communities has been observed in a number of host species, with processes such as differences in diet and/or host genetics (Smith et al., 2015; Goertz et al., 2019; Suzuki et al., 2019), environmental differences such as soil properties (Grieneisen et al., 2019) or ecological drift (Lankau et al., 2012; Linnenbrink et al., 2013; Stothart et al., 2020) cited as drivers of this variation. In contrast, the captive colony of wood mice retained approximately half of the diversity of the wild population from which it likely originated, indicating a slow rate of taxon loss over time in captivity with minimal input of new microbes, as has been observed in other mouse systems (Kohl and Dearing, 2014; Sonnenburg et al., 2016). The degree of ASV sharing between the wild and captive populations studied here could be driven either by the degree of host or microbial genetic divergence between populations (highest between the two wild populations, but lower between the colony and Wytham), or by variation in of environmental microbial input, which is high in wild populations but limited in captivity.

Associations between microbiota structure and host body mass (Turnbaugh et al., 2006; Sommer et al., 2016), age (Degnan et al., 2012; Bennett et al., 2016) and reproductive status (Amato et al., 2014b) have been detected previously. However, here we show that sampling month remains a strong predictor of microbiota variation after accounting for these intrinsic host factors, and seasonality is therefore likely driven by external factors in this system. These seasonal microbiota changes were much stronger than any host-associated effects examined, with sampling month explaining more than twice the variation in community structure than any other variable tested, and more than all host-related variables combined. This suggests that associations with host factors are relatively weak compared to external drivers of gut microbiota variation, in line with studies on other wild animal populations (Ren et al., 2017; Goertz et al., 2019) and humans (Rothschild et al., 2018).

By sampling with high temporal resolution throughout multiple years, we detected a strong seasonal oscillation in microbiota structure that consistently occurred between July and February. This seasonal shift occurred independently of host age, sex or reproductive status, and was also observed within individuals. The timing and form of this seasonal microbiota restructuring was remarkably consistent across both populations and all years examined, and strongly resembled patterns detected in a 2-year study of a separate United Kingdom wood mouse population (Maurice et al., 2015). Other studies in wild animals have similarly reported a dominant role of season in restructuring the gut microbiota compared to host factors (Kobayashi et al., 2006; Williams et al., 2013; Amato et al., 2014a; Fogel, 2015; Maurice et al., 2015; Sun et al., 2016; Ren et al., 2017; Springer et al., 2017; Liu et al., 2019). However, this is the first time such seasonality has been shown to have such a consistent pattern across multiple populations and years. The striking repeatability of this seasonal pattern across populations suggests common drivers are at play. These could include predictable seasonal shifts in diet, parasitic infection, host physiology or social behaviour (David et al., 2014; Kreisinger et al., 2015; Perofsky et al., 2017). Hibernation has been linked to restructuring of the gut microbiota in ground squirrels and brown bears (Carey et al., 2013; Sommer et al., 2016). Although wood mice do not hibernate, they can enter short periods of torpor during colder months, with decreased body temperature and metabolic activity. However, this explanation of seasonal restructuring in the wood mouse gut microbiota is unlikely, as the major seasonal restructuring begins in summer. Therefore, seasonal shifts in diet are a strong candidate mechanism. Wood mice are omnivorous species that in woodland habitats rely on nuts that mature in summer and are cached for consumption until the following spring, such that their diet shows a strong increase in this material during the autumn/winter (Watts, 1968). This predictable influx of nuts to the diet could be what drives consistent seasonal restructuring across populations and years in woodland habitats, a hypothesis that could be tested in this system through direct measurement of seasonal diet-microbiota links.

Despite strong seasonal changes in the gut microbiota that were observed consistently across individuals and populations, a high degree of individuality in the gut microbiota was still detectable. On average, individuals were more similar to themselves over time than to other individuals sampled simultaneously. This is in contrast to recent findings from group-living primates, which showed spatiotemporal dynamics at the group rather than individual level (Perofsky et al., 2021). Such individuality could be explained by genetic differences (Benson et al., 2010), or persistent environmental or behavioural differences, for example, in habitat, dietary preferences or parasite burden. This individuality signal weakened as sampling interval increased, suggesting that temporal changes (e.g., environmental or aging-related) can affect the strength of this individual gut microbial signature (Faith et al., 2013). We also found seasonal variation in the degree of microbiota similarity between hosts, with the gut microbiota being more similar among mice caught in winter and early spring. This convergence of gut microbiotas in February-March was sufficiently strong to reduce the signal of individuality and even temporarily override it within the Wytham population. This could be due to a more homogenous diet at this time of year, when wood mice are thought to eat mainly cached seeds and nuts, compared to later spring/summer, when a more diverse array of both plant and animal items is available (Watts, 1968). Alternatively, seasonal convergence could be driven by seasonal change in population density and/or social interactions, which could influence rates or patterns of microbial transmission among hosts (Wolton, 1985; Raulo et al., 2021). Seasonal convergence between individual hosts in relation to the signal of individuality in the gut microbiota has not been previously examined. However, this could have consequences for the host population in terms of how well the gut microbiota can provide resilience to perturbations such as altered food availability or pathogen invasion at different times of the year.

The repeatability of seasonal microbiota dynamics across populations observed here is particularly striking given these two host populations shared no bacterial ASVs in common, although the ASVs they possessed were phylogenetically similar. Some, but not all, of the higher order taxa associated with seasonal changes were also consistent across populations. For instance, Lactobacillaceae ASVs were implicated in both Wytham and Silwood, consistent with previous work on wood mice (Maurice et al., 2015), while members of Ruminococcaceae, Muribaculaceae, Bifidobacteriaceae and Lachnospiraceae were important in Wytham only. Thus across populations, different bacterial taxa appear to respond synchronously to the same seasonal change. This suggests a level of functional redundancy in the gut microbial taxa of wood mice that respond seasonally to the same stimuli, at broad geographical scales. Convergence of gut microbiota composition or functional capability linked to environmental conditions has been shown in primates and humans (Gomez et al., 2019; Sharma et al., 2020), myrmecophagous mammals (Delsuc et al., 2014) and in yaks and Tibetan sheep (Zhang et al., 2016; Guo et al., 2021). Further functional studies, for example, using metagenomic or metatranscriptomic approaches, would be valuable to illuminate what seasonal functions these microbes might perform for the wood mouse host, and the potential significance of such microbiome shifts in providing hosts living in variable environments with adaptive seasonal plasticity.

## Data Availability Statement

The data presented in the study are deposited in the European Nucleotide Archive, accession number PRJEB49639.

## Ethics Statement

The animal study was reviewed and approved by Animal Welfare and Ethical Review Body, Royal Veterinary College, University of London.

## Author Contributions

KJM and SCLK conceived the ideas and designed the methodology. KJM, AMR, MB, TT, HME, BA, SV, and RR collected the data. ABP provided the mouse colony and supported research using it. KJM analysed the data and led the writing of the manuscript. All authors contributed to writing the article and approved the submitted version.

## Funding

This work was supported by a NERC fellowship (NE.L011867/1) to SCLK, and funding from the European Research Council (ERC) under the European Union’s Horizon 2020 research and innovation programme (grant agreement n° 851550). KJM was supported by a Royal Veterinary College (RVC) studentship (supervised by SCLK and JPW). SV was supported by a Darwin Trust studentship at the University of Edinburgh. ABP was supported by a University of Edinburgh Chancellors Fellowship, a Wellcome Trust Institutional Strategic Support Fund (ISSF) grant (J22737), and a Wellcome Trust Strategic Grant for the Centre for Immunity Infection and Evolution (095831).

## Conflict of Interest

The authors declare that the research was conducted in the absence of any commercial or financial relationships that could be construed as a potential conflict of interest.

## Publisher’s Note

All claims expressed in this article are solely those of the authors and do not necessarily represent those of their affiliated organizations, or those of the publisher, the editors and the reviewers. Any product that may be evaluated in this article, or claim that may be made by its manufacturer, is not guaranteed or endorsed by the publisher.
