# Supplementary material for: Synchronous Seasonality in the Gut Microbiota of Wild Mouse Populations
Source: Front Microbiol. 2022 Apr 25;13:809735. doi: 10.3389/fmicb.2022.809735 (PMC9083407; doi:10.3389/fmicb.2022.809735)
Supplement: Supplementary file 2 [file Data_Sheet_2.pdf]

Wood_mouse_FrontiersMS_Xpop_seasonal_microbiome_analyses

K Marsh

16/02/2022

The following script provides the analyses complementing the article published in Frontiers 'Marsh et al. (2022) Synchronous seasonality in the gut microbiota of wild wood mouse populations. Front. Microbiol. 13:809735'

This script is split into the following sections in line with the structure of the results. 1. Combining datasets and making phyloseq object (Wytham, Silwood, Colony) 2. Data filtering and cleaning 3. Cross-population comparisons 4. Core microbiomes 5. Predictors of gut microbiota composition 6. Seasonal restructuring 7. Individuality and seasonal convergence

Section 1: Combining datasets and making phyloseq objects

Load microbiome data (otu table and taxonomic assignments)

Load sample metadata

```
wyt_dat <- read.csv('Wytham_sample_metadata_full_forPhyloseq.csv')
exp_dat <- read.csv('Diet_shift_experiment_April_2017_results_forR.csv')
sil_dat <- read.csv('metadata_sample_with_nodemetrics_Silwood.csv')

#wytham metadata
#colnames(wyt_dat) # 4,8,10,12,13,17,18,19
wyt_dat_sub <- wyt_dat[,c(4,8,10,13,17,18,19)]
#head(wyt_dat_sub)
colnames(wyt_dat_sub)[colnames(wyt_dat_sub) == "XPIT_tag"] <- "Mouse.ID"
colnames(wyt_dat_sub)[colnames(wyt_dat_sub) == "Month"] <- "Diet_Month"
wyt_dat_sub$System <- "Wytham"
#colnames(wyt_dat_sub)
#summary(wyt_dat_sub$Sequencing_round)

#experiment metadata
#colnames(exp_dat) #1,7,9,33,38
exp_dat_sub <- exp_dat[,c(1,7,9,33,38)]
#head(exp_dat_sub)
exp_dat_sub$System <- "Colony"
exp_dat_sub$Sequencing_round <- "Exp_Wyt"
exp_dat_sub$Species <- "AS"
colnames(exp_dat_sub)[colnames(exp_dat_sub) == "Seq_ID"] <-
"Sequence_sample_code"
colnames(exp_dat_sub)[colnames(exp_dat_sub) == "Diet.type"] <- "Diet_Month"
```

```
colnames(exp_dat_sub)[colnames(exp_dat_sub) == "Batch"] <- "Extraction_batch"
exp_dat_sub$Extraction_batch <- paste("B", exp_dat_sub$Extraction_batch,
sep="")
exp_dat_sub$Mouse.ID <- paste(exp_dat_sub$System, exp_dat_sub$Mouse.ID,
sep="_")
#head(exp_dat_sub)
exp_dat_sub <- exp_dat_sub[,c("Mouse.ID", "Species",
"Diet_Month","Extraction_batch","Sequencing_round",
                              "Miseq_run", "Sequence_sample_code", "System"
)]

WEWD_meta <- rbind(wyt_dat_sub,exp_dat_sub)
summary(WEWD_meta)
```

```
##    Mouse.ID           Species           Diet_Month        Extraction_batch
##  Length:893         Length:893         Length:893         Length:893
##  Class :character   Class :character   Class :character   Class :character
##  Mode  :character   Mode  :character   Mode  :character   Mode  :character
##
##
##
##  Sequencing_round     Miseq_run     Sequence_sample_code    System
##  Length:893         Min.   :1.000   Length:893           Length:893
##  Class :character   1st Qu.:1.000   Class :character     Class :character
##  Mode  :character   Median :1.000   Mode  :character     Mode  :character
##                     Mean   :1.389
##                     3rd Qu.:2.000
##                     Max.   :2.000
```

```
colnames(sil_dat) #2, 8, 9, 17
```

```
##  [1] "X"                   "ID"                  "trapdate"
##  [4] "date"                "trap"                "pcr_plate"
##  [7] "success"             "Sample_name"         "Species"
## [10] "Sex"                 "Body_mass_grams"     "Age"
## [13] "Reprod"              "Ectoparasites_seen" "Body_condition"
## [16] "AGD"                 "month"               "year"
## [19] "months"              "season"              "degree"
## [22] "evc"                 "betweenness"         "habitat.common"
## [25] "grid.x_mean"         "grid.y_mean"
```

```
#head(sil_dat)
sil_dat_sub <- sil_dat[,c(2,8,9,17)]
sil_dat_sub$System <- "Silwood"
colnames(sil_dat_sub)[colnames(sil_dat_sub) == "month"] <- "Diet_Month"
colnames(sil_dat_sub)[colnames(sil_dat_sub) == "ID"] <- "Mouse.ID"
colnames(sil_dat_sub)[colnames(sil_dat_sub) == "Sample_name"] <-
"Sequence_sample_code"
sil_dat_sub$Sequencing_round <- "Silwood"
sil_dat_sub$Extraction_batch <- 'NA'
sil_dat_sub$Miseq_run <- 'NA'
```

Make phyloseq object

```
#Wytham&Colony
OTU<-otu_table(seqtab_WEWD_comb, taxa_are_rows=FALSE)
#sample_names(OTU)
SAMP <-sample_data(WEWD_meta)
rownames(SAMP)<- SAMP$Sequence_sample_code
#sample_names(SAMP)
TAX <- tax_table(taxa_Silva)
#rownames(TAX)
#taxa_names(OTU)
ps.WEWD <- phyloseq(OTU, SAMP, TAX)
#check that this contains right number of samples
#ps.WEWD

#Silwood
#rownames(seqtab_silwood)
rownames(seqtab_silwood)<-sapply(strsplit(rownames(seqtab_silwood),"\\-"),
`[`, 2)
rownames(sil_dat_sub)<-sil_dat_sub$Sequence_sample_code
ps.Silwood <- phyloseq(otu_table(seqtab_silwood, taxa_are_rows = FALSE),
                               sample_data(sil_dat_sub),
                               tax_table(seqtab_silwood_taxtab))
ps.Silwood

## phyloseq-class experiment-level object
## otu_table()   OTU Table:         [ 3823 taxa and 286 samples ]
## sample_data() Sample Data:       [ 286 samples by 8 sample variables ]
## tax_table()   Taxonomy Table:    [ 3823 taxa by 7 taxonomic ranks ]

#now merge the WEWD and Silwood phyloseq objects
#?merge_phyloseq
ps.all <- merge_phyloseq(ps.WEWD, ps.Silwood)
#ps.all
```

Section 2: Data filtering and cleaning

```
library(iNEXT)
library(ggplot2)
library(vegan)

## Loading required package: permute

## Loading required package: lattice

## This is vegan 2.5-7

library(forcats)

# add sample-wise read depth to metadata
sample_data(ps.all)$readDepth <- rowSums(otu_table(ps.all))
```

```
# Filter non-gut microbial taxa
tax_tab <- as.data.frame(tax_table(ps.all))
ps.all.taxfilt <- subset_taxa(ps.all, Kingdom!="Eukaryota" &
Kingdom!="Archaea" &
                                Phylum!="NA" & Phylum!="Cyanobacteria" &
Class!="Chloroplast" &
                                Family!="Mitochondria")
#get rid of taxa not found in any of these samples
ps.all.taxfilt2 <- prune_taxa(taxa_sums(ps.all.taxfilt) > 0, ps.all.taxfilt)

#change ASV names to something nicer to read, and store original full
sequences for each ASV in ref.seq slot
dna <- Biostrings::DNAStringSet(taxa_names(ps.all.taxfilt2))
names(dna) <- taxa_names(ps.all.taxfilt2)
ps.all.taxfilt2 <- merge_phyloseq(ps.all.taxfilt2, dna)
taxa_names(ps.all.taxfilt2) <- paste0("ASV", seq(ntaxa(ps.all.taxfilt2)))
#taxa_names(ps.all.taxfilt2)

#save master phyloseq object
#saveRDS(ps.all.taxfilt2,
'~/Full_16S_WEWD_plus_Silwood_phyloseq_taxfilt.rds')
```

Sample-wise filtering using iNEXT analyses

```
ps.all <- ps.all.taxfilt2
#what does read depth across samples look like before filtering?
summary(sample_data(ps.all)$readDepth)

##    Min. 1st Qu.  Median    Mean 3rd Qu.    Max.
##      26   30846   38545   39752   47167  508841

taxa_are_rows(ps.all)

## [1] FALSE

otu_tab <- as.data.frame(otu_table(ps.all))
otu_tab <- t(otu_tab) #make sure sample are columns and species are rows

#run iNEXT
ps.all_q0 <- iNEXT(otu_tab)

## Warning in EstiBootComm.Ind(Spec): This site has only one species.
Estimation is
## not robust.

## Warning in if (class(x) == "numeric") {: the condition has length > 1 and
only
## the first element will be used

## Warning in if (class(x) == "integer") {: the condition has length > 1 and
only
## the first element will be used
```

```
## Warning in if (class(x) == "list") {: the condition has length > 1 and
only the
## first element will be used

## Warning in if (class(x)[1] == "matrix" | class(x) == "data.frame") {: the
## condition has length > 1 and only the first element will be used

## Warning in if (class(x) == "numeric") {: the condition has length > 1 and
only
## the first element will be used

## Warning in if (class(x) == "integer") {: the condition has length > 1 and
only
## the first element will be used

## Warning in if (class(x) == "list") {: the condition has length > 1 and
only the
## first element will be used

## Warning in if (class(x)[1] == "matrix" | class(x) == "data.frame") {: the
## condition has length > 1 and only the first element will be used

## Warning in BootstrapFun.abun(x = x, FunName, datatype, B): The Bootstrap
## community has only one species. Estimation is not robust.

## Warning in if (class(x) == "numeric") {: the condition has length > 1 and
only
## the first element will be used

## Warning in if (class(x) == "integer") {: the condition has length > 1 and
only
## the first element will be used

## Warning in if (class(x) == "list") {: the condition has length > 1 and
only the
## first element will be used

## Warning in if (class(x)[1] == "matrix" | class(x) == "data.frame") {: the
## condition has length > 1 and only the first element will be used

## Warning in BootstrapFun.abun(x = x, FunName, datatype, B): The Bootstrap
## community has only one species. Estimation is not robust.

## Warning in if (class(x) == "numeric" | class(x) == "integer") {: the
condition
## has length > 1 and only the first element will be used

## Warning in if (class(x) == "list") {: the condition has length > 1 and
only the
## first element will be used

## Warning in if (class(x)[1] == "matrix" | class(x) == "data.frame") {: the
## condition has length > 1 and only the first element will be used
```

```
#plot curves
ggiNEXT(ps.all_q0, type = 1, color.var = "none") +
  theme(legend.position="none") +
  ggtitle("iNEXT sample-sized based rarefaction/extrapolation curve \n Full
16S dataset") +
  xlim(0,15000)
```

```
## Warning in ggiNEXT.iNEXT(ps.all_q0, type = 1, color.var = "none"): invalid
## color.var setting, the iNEXT object consists multiple orders, change
setting as
## order
```

```
## Warning: The shape palette can deal with a maximum of 6 discrete values
because
## more than 6 becomes difficult to discriminate; you have 1179. Consider
## specifying shapes manually if you must have them.
```

```
## Warning: Removed 1179 rows containing missing values (geom_point).
```

```
## Warning: Removed 36283 row(s) containing missing values (geom_path).
```

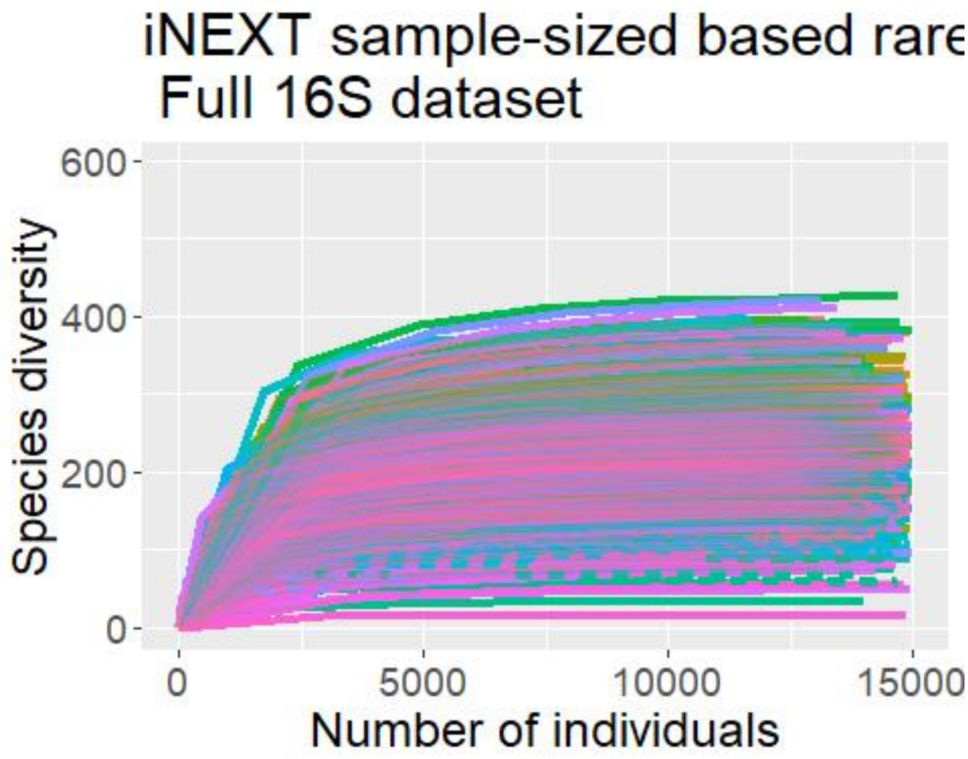


```
ggiNEXT(ps.all_q0, type = 2, color.var = "none") +
  theme(legend.position="none") +
  ggtitle("iNEXT sample completedness curve \n Full 16S dataset") +
  xlim(0,8300)
```

```
## Warning in ggiNEXT.iNEXT(ps.all_q0, type = 2, color.var = "none"): invalid
## color.var setting, the iNEXT object consists multiple orders, change
```

```
setting as
## order

## Warning: The shape palette can deal with a maximum of 6 discrete values
because
## more than 6 becomes difficult to discriminate; you have 1179. Consider
## specifying shapes manually if you must have them.

## Warning: Removed 1179 rows containing missing values (geom_point).

## Warning: Removed 40498 row(s) containing missing values (geom_path).
```

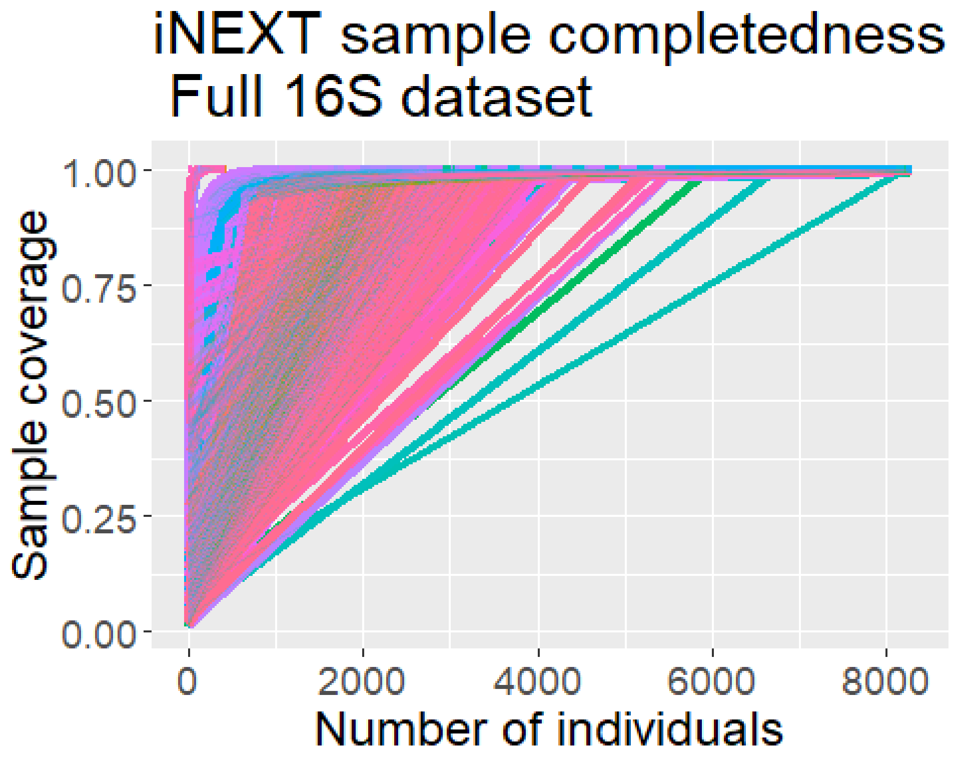


```
#prune samples where read depth < 8000
ps.all.prune <- prune_samples(sample_sums(ps.all)>=8000, ps.all)
#ps.all.prune
#saveRDS(ps.all.prune, '~/Full_16S_WEWD_plus_Silwood_phyloseq_sampfilt.rds')
#summary(as.factor(sample_data(ps.all.prune)$System))
```

OTU prevalence/abundance filtering

```
##criteria; keep taxa with > 1 copy in at least 1% samples
#removes any OTUs that weren't present in at least than 1% samples with a
copy number more than 1 (which will remove singletons)
ps.all.prune.filt = filter_taxa(ps.all.prune, function(x) sum(x > 1) >
(0.01*length(x)), prune=TRUE)
#ps.all.prune.filt
#saveRDS(ps.all.prune.filt,
'~/Full_16S_WEWD_plus_Silwood_phyloseq_OTUfilt.rds')
```

Make separate phyloseq objects per dataset (Wytham/Silwood) and add back in full metadata

```
sample_data(ps.all.prune.filt)$System <-
as.factor(sample_data(ps.all.prune.filt)$System)

## Wytham
ps.wyt <- subset_samples(ps.all.prune.filt, System=="Wytham")
ps.wyt <- prune_taxa(taxa_sums(ps.wyt) > 0, ps.wyt)#get rid of taxa not found
in any of these samples
wyt_dat2 <- as(sample_data(ps.wyt), 'data.frame')
wyt_dat_comb <- merge(wyt_dat, wyt_dat2, by="Sequence_sample_code")
SAMP <- sample_data(wyt_dat_comb)
rownames(SAMP) <- SAMP$Sequence_sample_code
ps.wyt.comb <- phyloseq(otu_table(otu_table(ps.wyt)),
tax_table(tax_table(ps.wyt)), sample_data(SAMP), refseq(refseq(ps.wyt)))
#saveRDS(ps.wyt.comb, '~/Wythmam_phyloseq_WEWDSIL_combfilt_raw.rds')

## Silwood
ps.sil <- subset_samples(ps.all.prune.filt, System=="Silwood")
ps.sil <- prune_taxa(taxa_sums(ps.sil) > 0, ps.sil)#get rid of taxa not found
in any of these samples
colnames(sil_dat)[colnames(sil_dat) == "Sample_name"] <-
"Sequence_sample_code"
sil_dat2 <- as(sample_data(ps.sil), 'data.frame')
sil_dat_comb <- merge(sil_dat, sil_dat2, by="Sequence_sample_code")
SAMP <- sample_data(sil_dat_comb)
rownames(SAMP) <- SAMP$Sequence_sample_code
ps.sil.comb <- phyloseq(otu_table(otu_table(ps.sil)),
tax_table(tax_table(ps.sil)), sample_data(SAMP))
#saveRDS(ps.sil.comb, '~/Silwood_phyloseq_WEWDSIL_combfilt_raw.rds')
```

Section 3: Cross-population comparisons

```
library(phyloseq)
library(ggplot2)
library(forcats)
library(reshape2)
library(plyr) ; library(dplyr)
library(compositions)
# install.packages("remotes")
# remotes::install_github("microbiome/microbiome")
library(microbiome)
library(gridExtra)
library(RColorBrewer)
library(DECIPHER)
library(phangorn)
library(VennDiagram)

ps.WSC<-ps.all.prune.filt
#ps.WSC <- subset_samples(ps.WSC, System=="Wytham" | System=="Silwood" |
```

```
System=="Colony")
ps.WSC <- subset_samples(ps.WSC, Species=="AS")
#summary(sample_data(ps.WSC))
ps.WSC <- prune_taxa(taxa_sums(ps.WSC) > 0, ps.WSC)#get rid of taxa not found
in any of these samples
ps.WSC.ra <- transform_sample_counts(ps.WSC, function(x) x/sum(x))#transform
to relative abudnace
levels(sample_data(ps.WSC.ra)$Mouse.ID)[levels(sample_data(ps.WSC.ra)$Mouse.I
D)==''] <- NA
meta <- as(sample_data(ps.WSC.ra), 'data.frame')
#levels(meta$Mouse.ID)
```

Ordinations of samples across populations

```
## Bray-Curtis
sample_ord1 <- ordinate(ps.WSC.ra, method= "PCoA", distance= "bray")
plot_bray <- plot_ordination(ps.WSC.ra, sample_ord1, type = "samples", color
= "System") +
  theme_bw() + ggtitle("Bray-Curtis") +
  scale_colour_manual(values=c("lightgoldenrod", "springgreen3",
"dodgerblue")) +
  coord_equal(ratio=1)
#plot_bray
##Jaccard
ps.WSC.pa <- transform_sample_counts(ps.WSC.ra, function(x) 1*(x>0))
sample_ord2 <- ordinate(ps.WSC.pa, method= "PCoA", distance= "jaccard")
plot_jacc <-
  plot_ordination(ps.WSC.pa, sample_ord1, type = "samples", color = "System")
+
  theme_bw() + ggtitle("Jaccard") +
  theme(legend.position = "none") +
  scale_colour_manual(values=c("lightgoldenrod", "springgreen3",
"dodgerblue"))
#plot_jacc
#ggsave('WSC_jaccard_ordination_biplot.pdf',plot_jacc, width=15, height=10)

##UniFrac (need to build phy_tree)
seq <- DNAStringSet(refseq(ps.WSC.ra)) #align ASVs
alignment <- AlignSeqs(DNAStringSet(seq), anchor=NA)

## Determining distance matrix based on shared 8-mers:
##
==============================================================================
===
##
## Time difference of 60.96 secs
##
## Clustering into groups by similarity:
##
==============================================================================
```

```
===
##
## Time difference of 6 secs
##
## Aligning Sequences:
##
==============================================================================
===
##
## Time difference of 59.2 secs
##
## Iteration 1 of 2:
##
## Determining distance matrix based on alignment:
##
==============================================================================
===
##
## Time difference of 6.4 secs
##
## Reclustering into groups by similarity:
##
==============================================================================
===
##
## Time difference of 5.26 secs
##
## Realigning Sequences:
##
==============================================================================
===
##
## Time difference of 52.19 secs
##
## Iteration 2 of 2:
##
## Determining distance matrix based on alignment:
##
==============================================================================
===
##
## Time difference of 5.04 secs
##
## Reclustering into groups by similarity:
##
==============================================================================
===
##
## Time difference of 1.47 secs
##
```

```
## Realigning Sequences:
##
================================================================================
===
##
## Time difference of 3.52 secs
```

```
#construct phylogenetic tree, using neighbour-joining tree as starting point
for GTR+G+I
phang.align <- phyDat(as(alignment, "matrix"), type="DNA" ) #phyDat in
Phangorn package
dm <- dist.ml (phang.align) #function dist.ml in phangorn package
treeNJ <- NJ(dm)
fit = pml(treeNJ, data =phang.align)
```

```
## negative edges length changed to 0!
```

```
fitGTR <- update(fit, k=4, inv=0.2 )
fitGTR <- optim.pml(fitGTR, model="GTR", optInv=TRUE, optGamma=TRUE ,
                    rearrangement = "stochastic", control = pml.control(trace
= 0 ))

ps_WSC_plustree <- phyloseq(tax_table(ps.WSC.ra),
                               sample_data (ps.WSC.ra),
                               otu_table(ps.WSC.ra),
                               refseq(refseq(ps.WSC.ra)),
                               phy_tree(fitGTR$tree)) #this is the newly
added phylogenetic tree of microbial sequences
#ps_WSC_plustree
#saveRDS(ps_WSC_plustree,
'~/Full_Wytham_Silwood_Colony_physeq_raTrans_plusphytree.rds')

#unweighted
sample_ord_unifrac <- ordinate(ps_WSC_plustree, method= "PCoA", distance=
"unifrac")
```

```
## Warning in UniFrac(physeq, ...): Randomly assigning root as -- ASV6721 --
in the
## phylogenetic tree in the data you provided.
```

```
uni_ord1 <- plot_ordination(ps_WSC_plustree, sample_ord_unifrac, type =
"samples", color = "System") +
  theme_bw() + ggtitle("Unweighted UniFrac") + theme(legend.position =
"none") +
  scale_colour_manual(values=c("lightgoldenrod", "springgreen3",
"dodgerblue"))
#weighted
sample_ord_wunifrac <- ordinate(ps_WSC_plustree, method= "PCoA", distance=
"wunifrac")
```

```
## Warning in UniFrac(physeq, weighted = TRUE, ...): Randomly assigning root
as --
## ASV269 -- in the phylogenetic tree in the data you provided.
```

```
uni_ord2 <- plot_ordination(ps_WSC_plustree, sample_ord_wunifrac, type =
"samples", color = "System") +
  theme_bw() + ggtitle("Weighted UniFrac") + theme(legend.position = "none")
+
  scale_colour_manual(values=c("lightgoldenrod", "springgreen3",
"dodgerblue"))
#unifrac_combplot <- grid.arrange(uni_ord1, uni_ord2, ncol=2)
#ggsave('WSC_unifrac_ordination_biplots.pdf',unifrac_combplot, width=15,
height=10)
```

Relative abundance composition at Order level

```
ps.WSC.ra.Ordglom <- ps.WSC.ra %>%
  tax_glom(taxrank = "Order") %>%
  merge_samples(group = "System") %>%
  psmelt() %>%
  arrange(Order)
```

```
## Warning in asMethod(object): NAs introduced by coercion

## Warning in asMethod(object): NAs introduced by coercion

## Warning in asMethod(object): NAs introduced by coercion

## Warning in asMethod(object): NAs introduced by coercion

## Warning in asMethod(object): NAs introduced by coercion

## Warning in asMethod(object): NAs introduced by coercion

## Warning in asMethod(object): NAs introduced by coercion
```

```
## Warning in psmelt(.): The sample variables:
## Species
##  have been renamed to:
## sample_Species
## to avoid conflicts with taxonomic rank names.
```

```
#too many categories, group less abundant into 'other'
ps.WSC.ra.Ordglom$Order <- ps.WSC.ra.Ordglom$Order %>%
  fct_collapse(Other = c("NA", "Unknown_Order" , "Mycoplasmatales",
"Rhodospirillales",

"Burkholderiales","Campylobacterales","Erysipelotrichales",
                         "Anaeroplasmatales", "NB1-n", "Mollicutes_RF9",
"Legionellales",
                         "Micrococcales", "Elusimicrobiales",
```

```
"Actinomycetales",
                         "Deferribacterales", "Flavobacteriales",
"Hot_Creek_32",
                         "Opitutae_vadinHA64", "Rhizobiales",
"Chthoniobacterales",
                         "Corynebacteriales", "Pasteurellales",
"Xanthomonadales", NA,
                         "Pseudomonadales" , "Spirochaetales",
"Propionibacteriales",
                         "Bacillales"))

## Warning: Unknown levels in `f`: NA, NB1-n, Mollicutes_RF9,
Flavobacteriales,
## Hot_Creek_32, Opitutae_vadinHA64, NA

colourCount = length(unique(ps.WSC.ra.Ordglom$Order))
getPalette = colorRampPalette(brewer.pal(12, "Paired"))

level_order2 <- c("Colony", "Wytham", "Silwood")
level_order3 <- c("Bacteroidales", "Bifidobacteriales", "Clostridiales",
"Coriobacteriales",
                  "Desulfovibrionales", "Enterobacteriales",
"Lactobacillales",
                  "Rickettsiales", "Other")

ps.WSC.ra.Ordglom$Order <- factor(ps.WSC.ra.Ordglom$Order, ordered = F,
levels = level_order3)

p2 <- ggplot(ps.WSC.ra.Ordglom, aes(x = factor(Sample, levels =
level_order2),
                                    y = Abundance, fill = Order)) +
  geom_bar(stat = "identity", position="fill")  +
  ylab("Relative Abundance") + xlab("Population") +
  theme_bw() +
  # theme(axis.text=element_text(size=16),axis.title=element_text(size=16),
  #       title = element_text(size=16), legend.text = element_text(size=14),
  #       legend.title = element_text(size=16))+
  theme(legend.text = element_text(size=8), legend.title =
element_text(size=10),
        legend.key.size = unit(0.4,'cm'), legend.position = "top")+
  scale_fill_manual(values = brewer.pal(9,"Paired"))

###Combine with jaccard and unifrac ordination plots
grid.arrange(p2, plot_jacc, uni_ord1, uni_ord2, nrow=2)
```

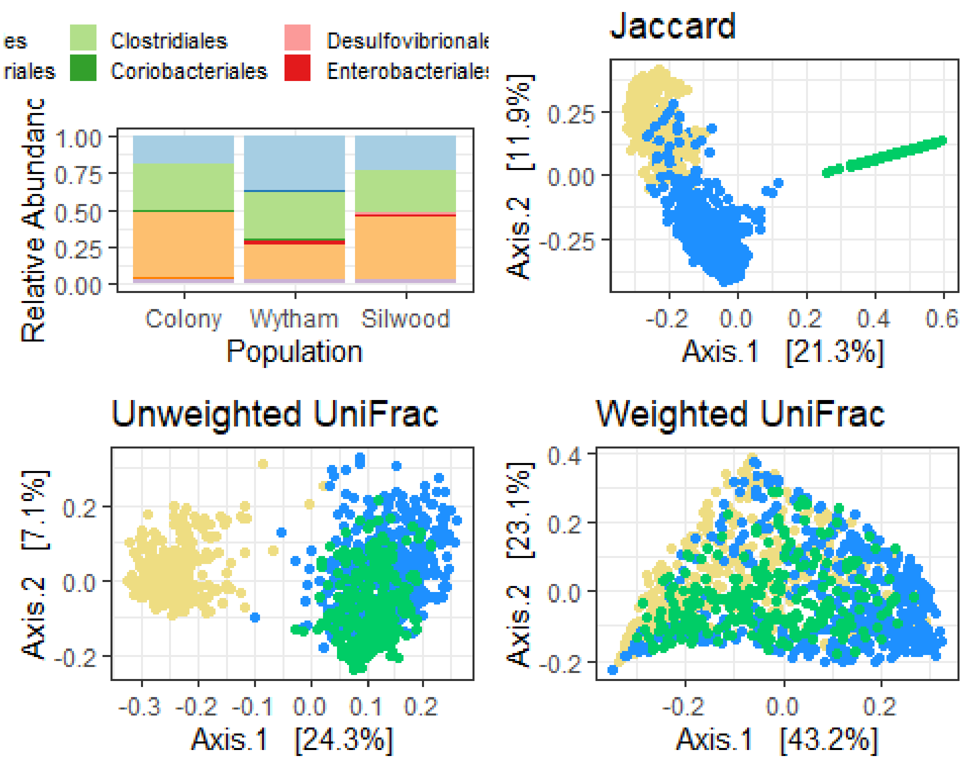

Plot per sample

```
ps.WSC.ra.Ordglom2 <- ps.WSC.ra %>%
  tax_glom(taxrank = "Order") %>%
  psmelt() %>%
  #filter(Abundance > 0.02) %>%
  arrange(Order)

## Warning in psmelt(.): The sample variables:
## Species
##  have been renamed to:
## sample_Species
## to avoid conflicts with taxonomic rank names.

ps.WSC.ra.Ordglom2$Order <- ps.WSC.ra.Ordglom2$Order %>%
  fct_collapse(Other = c("NA", "Unknown_Order" , "Mycoplasmatales",
"Rhodospirillales",

"Burkholderiales","Campylobacterales","Erysipelotrichales",
                         "Anaeroplasmatales", "NB1-n", "Mollicutes_RF9",
"Legionellales",
                         "Micrococcales", "Elusimicrobiales",
"Actinomycetales",
                         "Deferribacterales", "Flavobacteriales",
"Hot_Creek_32",
                         "Opitutae_vadinHA64","Rhizobiales",
"Chthoniobacterales",
                         "Corynebacteriales", "Pasteurellales",
"Xanthomonadales", NA,
```

```
                         "Pseudomonadales" , "Spirochaetales",
"Propionibacteriales",
                         "Bacillales"))

## Warning: Unknown levels in `f`: NA, NB1-n, Mollicutes_RF9,
Flavobacteriales,
## Hot_Creek_32, Opitutae_vadinHA64, NA

ps.WSC.ra.Ordglom2$Order <- factor(ps.WSC.ra.Ordglom2$Order, ordered = F,
levels = level_order3)

p2b <- ggplot(ps.WSC.ra.Ordglom2, aes(x = factor(Sample),
                                      y = Abundance, fill = Order)) +
  geom_bar(stat = "identity", position="fill", width = 1)  +
  facet_grid(~factor(System, levels = level_order2), scales = "free_x", space
= "free_x") +
  ylab("Relative Abundance") + xlab("Population") +
  theme_bw() +
  # theme(axis.text=element_text(size=16),axis.title=element_text(size=16),
  #       title = element_text(size=16), legend.text = element_text(size=14),
  #       legend.title = element_text(size=16))+
  theme(legend.text = element_text(size=8), legend.title =
element_text(size=10),
        legend.key.size = unit(0.4,'cm'), legend.position = "top",
        axis.text.x = element_blank(), axis.ticks.x = element_blank())+
  scale_fill_manual(values = brewer.pal(9,"Paired"))
p2b
```

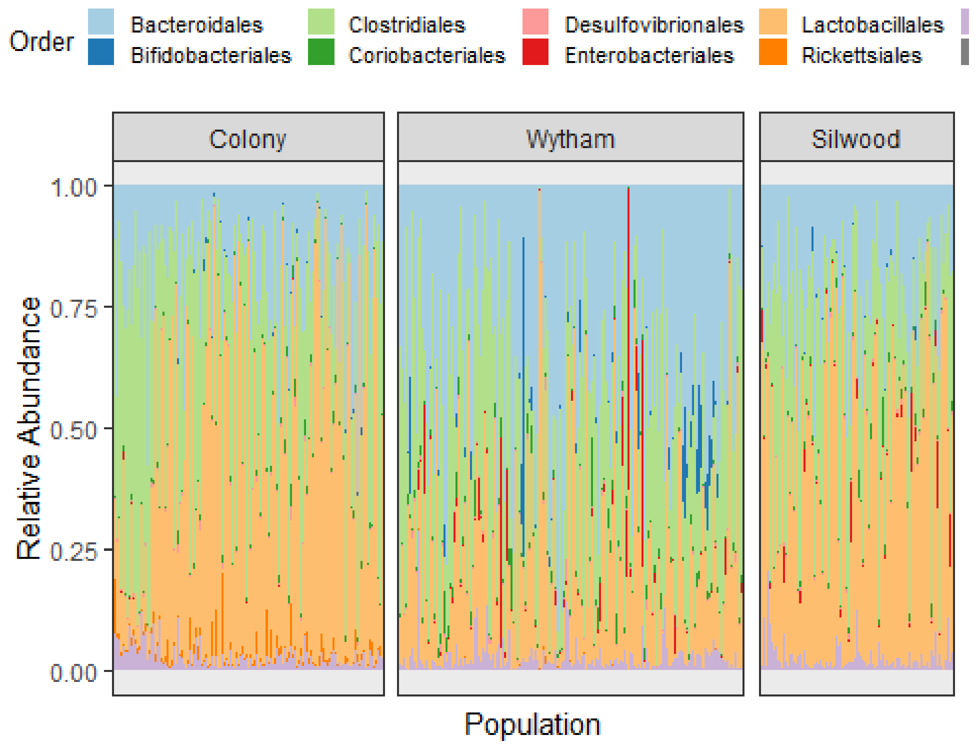

Overlap in taxa present across populations, at different taxonomic levels (Venn diagrams)

```
## (polygon[GRID.polygon.9940], polygon[GRID.polygon.9941],
polygon[GRID.polygon.9942], polygon[GRID.polygon.9943],
polygon[GRID.polygon.9944], polygon[GRID.polygon.9945], text[GRID.text.9946],
text[GRID.text.9947], text[GRID.text.9948], text[GRID.text.9949],
text[GRID.text.9950], text[GRID.text.9951], text[GRID.text.9952])
```

```
## (polygon[GRID.polygon.9953], polygon[GRID.polygon.9954],
polygon[GRID.polygon.9955], polygon[GRID.polygon.9956],
polygon[GRID.polygon.9957], polygon[GRID.polygon.9958], text[GRID.text.9959],
text[GRID.text.9960], text[GRID.text.9961], text[GRID.text.9962],
text[GRID.text.9963], text[GRID.text.9964], text[GRID.text.9965],
text[GRID.text.9966], text[GRID.text.9967])
```

```
## (polygon[GRID.polygon.9968], polygon[GRID.polygon.9969],
polygon[GRID.polygon.9970], polygon[GRID.polygon.9971],
polygon[GRID.polygon.9972], polygon[GRID.polygon.9973], text[GRID.text.9974],
text[GRID.text.9975], text[GRID.text.9976], text[GRID.text.9977],
text[GRID.text.9978], text[GRID.text.9979], text[GRID.text.9980],
text[GRID.text.9981], text[GRID.text.9982], text[GRID.text.9983])
```

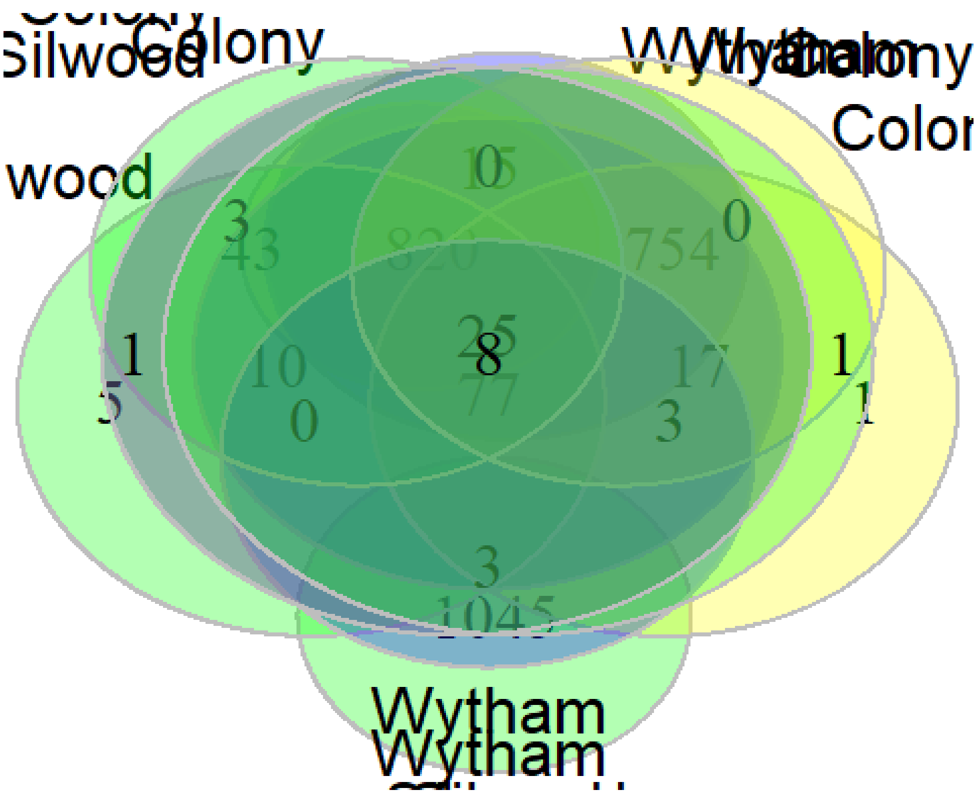


```
## (polygon[GRID.polygon.9984], polygon[GRID.polygon.9985],
polygon[GRID.polygon.9986], polygon[GRID.polygon.9987],
polygon[GRID.polygon.9988], polygon[GRID.polygon.9989], text[GRID.text.9990],
text[GRID.text.9991], text[GRID.text.9992], text[GRID.text.9993],
text[GRID.text.9994], text[GRID.text.9995])
```

Bootstrap subsampling routine for estimating proportions of shared taxa between groups

```
#add ASV column to tax_table
taxtab <- as.data.frame(tax_table(ps.WSC.ra))
taxtab$ASV <- rownames(taxtab)
head(taxtab)

##       Kingdom        Phylum       Class           Order
## ASV1 Bacteria    Firmicutes     Bacilli Lactobacillales
## ASV2 Bacteria    Firmicutes     Bacilli Lactobacillales
## ASV3 Bacteria    Firmicutes     Bacilli Lactobacillales
## ASV4 Bacteria Bacteroidetes Bacteroidia   Bacteroidales
## ASV5 Bacteria    Firmicutes     Bacilli Lactobacillales
## ASV6 Bacteria    Firmicutes     Bacilli Lactobacillales
##                          Family         Genus Species  ASV
## ASV1          Lactobacillaceae Lactobacillus    <NA> ASV1
## ASV2          Lactobacillaceae Lactobacillus    <NA> ASV2
## ASV3          Lactobacillaceae Lactobacillus    <NA> ASV3
## ASV4 Bacteroidales_S24-7_group          <NA>    <NA> ASV4
## ASV5          Lactobacillaceae Lactobacillus    <NA> ASV5
## ASV6          Lactobacillaceae Lactobacillus    <NA> ASV6

ps.WSC.ra <- phyloseq(sample_data(sample_data(ps.WSC.ra)),
                      tax_table(as.matrix(taxtab)),
                      otu_table(otu_table(ps.WSC.ra)),
                      refseq(refseq(ps.WSC.ra)))
colnames(tax_table(ps.WSC.ra))

## [1] "Kingdom" "Phylum"  "Class"   "Order"   "Family"  "Genus"   "Species"
## [8] "ASV"

tax_levels <- c("Phylum", "Order", "Genus", "ASV")

#create df to store results
subsample_iteration_dat <- data.frame(Iteration=NA, Ncommon=NA, NunqW=NA,
NunqC=NA, NunqS=NA,
                                      NsharedWC=NA, NsharedWS=NA,
NsharedSC=NA, Ntotal=NA, taxrank=NA)
subsample_iteration_dat.all <- list()

nrep <- 1000

for(j in 1:length(tax_levels)) {

   phy_glom <- tax_glom(ps.WSC.ra, taxrank = tax_levels[j])

    for(i in 1:nrep) {
      #select random samples for each habitat
      sample_sub <- as.data.frame(sample_data(phy_glom) %>%
                                    group_by(System) %>%
```

```
                                    sample_n(253))
      rownames(sample_sub) <- sample_sub$Sequence_sample_code
      temp.ps <- phyloseq(otu_table(otu_table(phy_glom)),
                         tax_table(tax_table(phy_glom)),
                         sample_data(sample_sub))
      temp.ps <- prune_taxa(taxa_sums(temp.ps) > 0,
                           temp.ps)
      ntax <- ntaxa(temp.ps)
      #subset phyloseq objects per system and get list of ASVs present
      phy_sil <- subset_samples(temp.ps,
                               System=="Silwood")
      phy_sil <- prune_taxa(taxa_sums(phy_sil) > 0,
                                 phy_sil)
      Sil_list <- taxa_names(phy_sil)
      phy_wyt <- subset_samples(temp.ps,
                               System=="Wytham")
      phy_wyt <- prune_taxa(taxa_sums(phy_wyt) > 0,
                                phy_wyt)
      Wyt_list <- taxa_names(phy_wyt)
      phy_col <- subset_samples(temp.ps,
                               System=="Colony")
      phy_col <- prune_taxa(taxa_sums(phy_col) > 0,
                              phy_col)
      Col_list <- taxa_names(phy_col)
      #calculate the number of shared/unique ASVs
      common <- (length(intersect(intersect(Col_list, Wyt_list),
Sil_list))/ntax)
      unqW <- (length(setdiff(setdiff(Wyt_list, Col_list),Sil_list))/ntax)
      unqC <- (length(setdiff(setdiff(Col_list, Wyt_list),Sil_list))/ntax)
      unqS <- (length(setdiff(setdiff(Sil_list, Wyt_list),Col_list))/ntax)
      shared_WC <- (length(setdiff(intersect(Wyt_list, Col_list),
common))/ntax)
      shared_WS <- (length(setdiff(intersect(Wyt_list, Sil_list),
common))/ntax)
      shared_SC <- (length(setdiff(intersect(Sil_list, Col_list),
common))/ntax)
      #store results in df
      subsample_iteration_dat[i, 1] <- i
      subsample_iteration_dat[i, 2] <- common
      subsample_iteration_dat[i, 3] <- unqW
      subsample_iteration_dat[i, 4] <- unqC
      subsample_iteration_dat[i, 5] <- unqS
      subsample_iteration_dat[i, 6] <- shared_WC
      subsample_iteration_dat[i, 7] <- shared_WS
      subsample_iteration_dat[i, 8] <- shared_SC
      subsample_iteration_dat[i, 9] <- ntax
      subsample_iteration_dat[i, 10] <- j

    }
```

```
  subsample_iteration_dat.all[[j]] <- subsample_iteration_dat

}

str(subsample_iteration_dat.all)

## List of 4
##  $ :'data.frame':    1000 obs. of  10 variables:
##   ..$ Iteration: int [1:1000] 1 2 3 4 5 6 7 8 9 10 ...
##   ..$ Ncommon  : num [1:1000] 0.7 0.7 0.7 0.7 0.7 0.7 0.7 0.7 0.7 0.7 ...
##   ..$ NunqW    : num [1:1000] 0.1 0.1 0.1 0.1 0.1 0.1 0.1 0.1 0.1 0.1 ...
##   ..$ NunqC    : num [1:1000] 0 0 0 0 0 0 0 0 0 0 ...
##   ..$ NunqS    : num [1:1000] 0.1 0.1 0.1 0.1 0.1 0.1 0.1 0.1 0.1 0.1 ...
##   ..$ NsharedWC: num [1:1000] 0.8 0.8 0.8 0.8 0.8 0.8 0.8 0.8 0.8 0.8 ...
##   ..$ NsharedWS: num [1:1000] 0.7 0.7 0.7 0.7 0.7 0.7 0.7 0.7 0.7 0.7 ...
##   ..$ NsharedSC: num [1:1000] 0.7 0.7 0.7 0.7 0.7 0.7 0.7 0.7 0.7 0.7 ...
##   ..$ Ntotal   : int [1:1000] 10 10 10 10 10 10 10 10 10 10 ...
##   ..$ taxrank  : int [1:1000] 1 1 1 1 1 1 1 1 1 1 ...
##  $ :'data.frame':    1000 obs. of  10 variables:
##   ..$ Iteration: int [1:1000] 1 2 3 4 5 6 7 8 9 10 ...
##   ..$ Ncommon  : num [1:1000] 0.71 0.71 0.71 0.71 0.71 ...
##   ..$ NunqW    : num [1:1000] 0.129 0.129 0.129 0.129 0.129 ...
##   ..$ NunqC    : num [1:1000] 0 0 0 0 0 0 0 0 0 0 ...
##   ..$ NunqS    : num [1:1000] 0.0645 0.0645 0.0645 0.0645 0.0645 ...
##   ..$ NsharedWC: num [1:1000] 0.806 0.806 0.806 0.806 0.806 ...
##   ..$ NsharedWS: num [1:1000] 0.71 0.71 0.71 0.71 0.71 ...
##   ..$ NsharedSC: num [1:1000] 0.71 0.71 0.71 0.71 0.71 ...
##   ..$ Ntotal   : int [1:1000] 31 31 31 31 31 31 31 31 31 31 ...
##   ..$ taxrank  : int [1:1000] 2 2 2 2 2 2 2 2 2 2 ...
##  $ :'data.frame':    1000 obs. of  10 variables:
##   ..$ Iteration: int [1:1000] 1 2 3 4 5 6 7 8 9 10 ...
##   ..$ Ncommon  : num [1:1000] 0.598 0.614 0.598 0.591 0.606 ...
##   ..$ NunqW    : num [1:1000] 0.1024 0.1024 0.1024 0.0945 0.1024 ...
##   ..$ NunqC    : num [1:1000] 0.01575 0.00787 0.00787 0.00787 0.02362 ...
##   ..$ NunqS    : num [1:1000] 0.0551 0.0551 0.0551 0.0551 0.0551 ...
##   ..$ NsharedWC: num [1:1000] 0.717 0.74 0.724 0.724 0.717 ...
##   ..$ NsharedWS: num [1:1000] 0.709 0.709 0.709 0.709 0.709 ...
##   ..$ NsharedSC: num [1:1000] 0.598 0.614 0.598 0.591 0.606 ...
##   ..$ Ntotal   : int [1:1000] 127 127 127 127 127 127 127 127 127 127 ...
##   ..$ taxrank  : int [1:1000] 3 3 3 3 3 3 3 3 3 3 ...
##  $ :'data.frame':    1000 obs. of  10 variables:
##   ..$ Iteration: int [1:1000] 1 2 3 4 5 6 7 8 9 10 ...
##   ..$ Ncommon  : num [1:1000] 0 0 0 0 0 0 0 0 0 0 ...
##   ..$ NunqW    : num [1:1000] 0.282 0.278 0.282 0.276 0.292 ...
##   ..$ NunqC    : num [1:1000] 0.0186 0.0201 0.0197 0.0194 0.0194 ...
##   ..$ NunqS    : num [1:1000] 0.421 0.421 0.421 0.421 0.421 ...
##   ..$ NsharedWC: num [1:1000] 0.279 0.281 0.278 0.284 0.268 ...
##   ..$ NsharedWS: num [1:1000] 0 0 0 0 0 0 0 0 0 0 ...
##   ..$ NsharedSC: num [1:1000] 0 0 0 0 0 0 0 0 0 0 ...
##   ..$ Ntotal   : int [1:1000] 2685 2686 2685 2685 2686 2685 2685 2686 2686
```

```
2686 ...
##   ..$ taxrank  : int [1:1000] 4 4 4 4 4 4 4 4 4 4 ...

subsample_iteration_df <- bind_rows(subsample_iteration_dat.all, .id =
"column_label")
head(subsample_iteration_df)

##   column_label Iteration Ncommon NunqW NunqC NunqS NsharedWC NsharedWS
## 1            1         1     0.7   0.1     0   0.1       0.8       0.7
## 2            1         2     0.7   0.1     0   0.1       0.8       0.7
## 3            1         3     0.7   0.1     0   0.1       0.8       0.7
## 4            1         4     0.7   0.1     0   0.1       0.8       0.7
## 5            1         5     0.7   0.1     0   0.1       0.8       0.7
## 6            1         6     0.7   0.1     0   0.1       0.8       0.7
##   NsharedSC Ntotal taxrank
## 1       0.7     10       1
## 2       0.7     10       1
## 3       0.7     10       1
## 4       0.7     10       1
## 5       0.7     10       1
## 6       0.7     10       1

#replace taxrank numbers with the actual taxrank
subsample_iteration_df$taxrank[subsample_iteration_df$taxrank==1] <- "Phylum"
subsample_iteration_df$taxrank[subsample_iteration_df$taxrank==2] <- "Order"
subsample_iteration_df$taxrank[subsample_iteration_df$taxrank==3] <- "Genus"
subsample_iteration_df$taxrank[subsample_iteration_df$taxrank==4] <- "ASV"

subsample_iteration_dat_melt <- melt(subsample_iteration_df[,c(3:9,11)],
id.vars="taxrank")
head(subsample_iteration_dat_melt)

##   taxrank variable value
## 1  Phylum  Ncommon   0.7
## 2  Phylum  Ncommon   0.7
## 3  Phylum  Ncommon   0.7
## 4  Phylum  Ncommon   0.7
## 5  Phylum  Ncommon   0.7
## 6  Phylum  Ncommon   0.7

colnames(subsample_iteration_dat_melt) <- c("Taxrank", "Community_subset",
"prop_taxa")

mu <- ddply(subsample_iteration_dat_melt, c("Taxrank","Community_subset"),
summarise,
            grp.mean=mean(prop_taxa),
            grp.sd=sd(prop_taxa),
            U_CI = grp.mean + 1.96*(grp.sd/sqrt(1000)),
            L_CI = grp.mean - 1.96*(grp.sd/sqrt(1000))
)
head(mu)
```

```
##   Taxrank Community_subset   grp.mean       grp.sd       U_CI       L_CI
## 1     ASV          Ncommon 0.00000000 0.0000000000 0.00000000 0.00000000
## 2     ASV            NunqW 0.28025272 0.0054410502 0.28058996 0.27991548
## 3     ASV            NunqC 0.02004319 0.0015575322 0.02013973 0.01994665
## 4     ASV            NunqS 0.42079379 0.0001016461 0.42080009 0.42078749
## 5     ASV        NsharedWC 0.27891030 0.0056240854 0.27925889 0.27856172
## 6     ASV        NsharedWS 0.00000000 0.0000000000 0.00000000 0.00000000
```

```
mu$CI <- mu$U_CI - mu$grp.mean
mu
```

```
##    Taxrank Community_subset    grp.mean       grp.sd        U_CI
L_CI
## 1      ASV          Ncommon 0.000000000 0.000000e+00 0.000000000
0.000000000
## 2      ASV            NunqW 0.280252717 5.441050e-03 0.280589957
0.279915477
## 3      ASV            NunqC 0.020043188 1.557532e-03 0.020139725
0.019946652
## 4      ASV            NunqS 0.420793791 1.016461e-04 0.420800091
0.420787490
## 5      ASV        NsharedWC 0.278910304 5.624085e-03 0.279258889
0.278561720
## 6      ASV        NsharedWS 0.000000000 0.000000e+00 0.000000000
0.000000000
## 7      ASV        NsharedSC 0.000000000 0.000000e+00 0.000000000
0.000000000
## 8    Genus          Ncommon 0.601787402 1.018261e-02 0.602418526
0.601156277
## 9    Genus            NunqW 0.099307087 5.162652e-03 0.099627071
0.098987102
## 10   Genus            NunqC 0.015858268 6.330877e-03 0.016250660
0.015465876
## 11   Genus            NunqS 0.055118110 0.000000e+00 0.055118110
0.055118110
## 12   Genus        NsharedWC 0.722842520 1.453776e-02 0.723743579
0.721941460
## 13   Genus        NsharedWS 0.708590551 7.439977e-04 0.708636665
0.708544438
## 14   Genus        NsharedSC 0.601858268 1.016807e-02 0.602488491
0.601228044
## 15   Order          Ncommon 0.700604301 1.462797e-02 0.701510952
0.699697650
## 16   Order            NunqW 0.129003226 9.180806e-04 0.129060129
0.128946323
## 17   Order            NunqC 0.001870968 7.543884e-03 0.002338543
0.001403393
## 18   Order            NunqS 0.064518280 6.800597e-05 0.064522495
0.064514065
## 19   Order        NsharedWC 0.795510753 1.657212e-02 0.796537903
```

```
0.794483602
## 20   Order        NsharedWS 0.709701075 7.480657e-04 0.709747441
0.709654710
## 21   Order        NsharedSC 0.700604301 1.462797e-02 0.701510952
0.699697650
## 22  Phylum          Ncommon 0.700000000 0.000000e+00 0.700000000
0.700000000
## 23  Phylum            NunqW 0.100000000 0.000000e+00 0.100000000
0.100000000
## 24  Phylum            NunqC 0.000000000 0.000000e+00 0.000000000
0.000000000
## 25  Phylum            NunqS 0.100000000 0.000000e+00 0.100000000
0.100000000
## 26  Phylum        NsharedWC 0.800000000 0.000000e+00 0.800000000
0.800000000
## 27  Phylum        NsharedWS 0.700000000 0.000000e+00 0.700000000
0.700000000
## 28  Phylum        NsharedSC 0.700000000 0.000000e+00 0.700000000
0.700000000
##              CI
## 1  0.000000e+00
## 2  3.372398e-04
## 3  9.653684e-05
## 4  6.300092e-06
## 5  3.485844e-04
## 6  0.000000e+00
## 7  0.000000e+00
## 8  6.311245e-04
## 9  3.199845e-04
## 10 3.923918e-04
## 11 0.000000e+00
## 12 9.010598e-04
## 13 4.611346e-05
## 14 6.302235e-04
## 15 9.066512e-04
## 16 5.690323e-05
## 17 4.675748e-04
## 18 4.215054e-06
## 19 1.027151e-03
## 20 4.636559e-05
## 21 9.066512e-04
## 22 0.000000e+00
## 23 0.000000e+00
## 24 0.000000e+00
## 25 0.000000e+00
## 26 0.000000e+00
## 27 0.000000e+00
## 28 0.000000e+00

levels(subsample_iteration_dat_melt$Community_subset)
```

```
## [1] "Ncommon"   "NunqW"     "NunqC"     "NunqS"     "NsharedWC"
"NsharedWS"
## [7] "NsharedSC"

subsample_iteration_dat_melt$Community_subset<-
factor(subsample_iteration_dat_melt$Community_subset,
                                                       level_order <-
c("NunqW","NunqC" , "NunqS",

"Ncommon","NsharedWC",

"NsharedWS", "NsharedSC"))

plot_bootstrap <- ggplot(subsample_iteration_dat_melt,
                aes(x=prop_taxa, group=Community_subset)) +
  geom_density() +
  geom_vline(data=mu, aes(xintercept=grp.mean,color=Taxrank),
             linetype="dashed") +
  facet_grid(Community_subset ~ Taxrank,
             scales = "free", drop=T) +
  theme_bw() +
  geom_rect(data=mu, aes(x=NULL, y=NULL,xmin = L_CI, xmax = U_CI,
                      ymin = -Inf, ymax = Inf),
        fill = "lightgrey", alpha=0.5) +
  theme(legend.position = "none",
        axis.text = element_text(size=20),
        axis.title = element_text(size=24),
        strip.text = element_text(size=22)) +
  xlab("Proportion of shared taxa (/total)") +
  ylab("Bootstrap density estimate")

plot_bootstrap
```

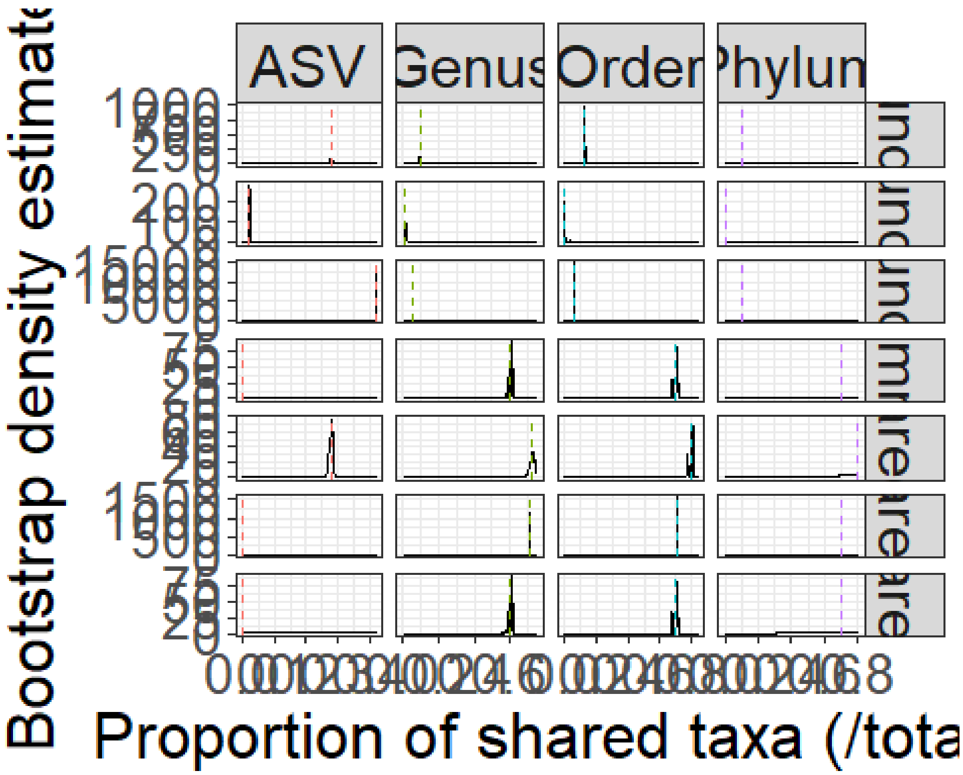


```
#ggsave('WCS_bootstrap_est_common_unique_subsets_density_plot.pdf',plot_boots
trap, width=20, height=20)
```

Pairwise Jaccard distances (% shared taxa) between W-S-C at the sample-level

```
levels(sample_data(ps.WSC.ra)$Mouse.ID)[levels(sample_data(ps.WSC.ra)$Mouse.I
D)==''] <- NA
ps.WSC.ra.known.ind <- subset_samples(ps.WSC.ra, !is.na(Mouse.ID))
ps.WSC.ra.known.ind <- prune_taxa(taxa_sums(ps.WSC.ra.known.ind) > 0,
ps.WSC.ra.known.ind)
#ps.WSC.ra.known.ind
D<- phyloseq::distance(ps.WSC.ra.known.ind, method = "jaccard", type =
"samples")
D <- as.matrix(D)
D_melt <- melt(D)
D_melt$Sample_pair <- paste(D_melt$Var1, D_melt$Var2, sep="-")
M <- as(sample_data(ps.WSC.ra.known.ind), 'data.frame')

colnames(D_melt)[1] <- "Sequence_sample_code"
D_melt$system.A <- "NA"
D_melt$system.A = M[match(D_melt$Sequence_sample_code,
M$Sequence_sample_code),"System"]
D_melt$ID.A = M[match(D_melt$Sequence_sample_code,
M$Sequence_sample_code),"Mouse.ID"]
colnames(D_melt)[1] <- "Var1"
colnames(D_melt)[2] <- "Sequence_sample_code"
D_melt$system.B <- "NA"
```

```r
D_melt$system.B = M[match(D_melt$Sequence_sample_code,
M$Sequence_sample_code),"System"]
D_melt$ID.B = M[match(D_melt$Sequence_sample_code,
M$Sequence_sample_code),"Mouse.ID"]
colnames(D_melt)[2] <- "Var2"

D_melt <- D_melt[D_melt$Var1!=D_melt$Var2, ]#remove pairwise comparison
between same samples
head(D_melt)

##    Var1  Var2     value Sample_pair system.A      ID.A system.B      ID.B
## 2 WE101 WE100 0.6435429 WE101-WE100   Colony Colony_29   Colony Colony_21
## 3 WE102 WE100 0.6872288 WE102-WE100   Colony  Colony_5   Colony Colony_21
## 4 WE103 WE100 0.7224795 WE103-WE100   Colony Colony_11   Colony Colony_21
## 5 WE104 WE100 0.7227435 WE104-WE100   Colony Colony_18   Colony Colony_21
## 6 WE105 WE100 0.7249419 WE105-WE100   Colony Colony_24   Colony Colony_21
## 7 WE106 WE100 0.5847609 WE106-WE100   Colony  Colony_1   Colony Colony_21

#keep only pairwise comparisons between different systems (w-c)
D_melt_diff <- D_melt[D_melt$system.A!=D_melt$system.B, ]
D_melt_w <- D_melt_diff[D_melt_diff$system.A=="Wytham", ]#w-c/s
D_melt_wc <- D_melt_w[D_melt_w$system.B=="Colony", ]#w-c

#keep only pairwise comparison between same system (different mice)
D_melt_same <- D_melt[D_melt$system.A==D_melt$system.B, ]
D_melt_same <- D_melt_same[D_melt_same$ID.A!=D_melt_same$ID.B, ]
D_melt_ww <- D_melt_same[D_melt_same$system.A=="Wytham", ]
D_melt_cc <- D_melt_same[D_melt_same$system.A=="Colony", ]
D_melt_ss <- D_melt_same[D_melt_same$system.A=="Silwood", ]

D_melt_wc$Comparison <- "Wytham-Colony"
D_melt_ww$Comparison <- "Wytham-Wytham"
D_melt_cc$Comparison <- "Colony-Colony"
D_melt_ss$Comparison <- "Silwood-Silwood"

#average proportion of shared ASVs between samples (mean Jdist)
1- mean(D_melt_ww$value)

## [1] 0.1142431

1- mean(D_melt_cc$value)

## [1] 0.232571

1- mean(D_melt_ss$value)

## [1] 0.1789376

#average proportion of shared ASVs between wytham and colony samples
1- mean(D_melt_wc$value)
```

```
## [1] 0.08393127
```

Taxonomic distributions of unique/shared subsets

```
#Common SV subset
ps.WSC.ra_commonSVsub <- subset_taxa(ps.WSC.ra,
taxa_names(ps.WSC.ra)%in%common_ASVs_WC)
ps.WSC.ra_commonSVsub <- prune_samples(sample_sums(ps.WSC.ra_commonSVsub)>0 ,
ps.WSC.ra_commonSVsub)
#Experiment - unique SV subset
ps.WSC.ra_unique_SVs <- subset_taxa(ps.WSC.ra,
!(taxa_names(ps.WSC.ra)%in%common_ASVs_WC))
ps.WSC.ra_exp_unique_SVs <- subset_samples(ps.WSC.ra_unique_SVs,
System=="Colony")
ps.WSC.ra_exp_unique_SVs <- prune_taxa(taxa_sums(ps.WSC.ra_exp_unique_SVs)>0,
ps.WSC.ra_exp_unique_SVs)
ps.WSC.ra_exp_unique_SVs <-
prune_samples(sample_sums(ps.WSC.ra_exp_unique_SVs)>0 ,
ps.WSC.ra_exp_unique_SVs)
#Wytham - unique SV subset
ps.WSC.ra_wyt_unique_SVs <- subset_samples(ps.WSC.ra_unique_SVs,
System=="Wytham")
ps.WSC.ra_wyt_unique_SVs <- prune_taxa(taxa_sums(ps.WSC.ra_wyt_unique_SVs)>0,
ps.WSC.ra_wyt_unique_SVs)
ps.WSC.ra_wyt_unique_SVs <-
prune_samples(sample_sums(ps.WSC.ra_wyt_unique_SVs)>0 ,
ps.WSC.ra_wyt_unique_SVs)
#Experiment- All
ps.WSC.ra_exp_all <- subset_samples(ps.WSC.ra, System=="Colony")
ps.WSC.ra_exp_all <- prune_taxa(taxa_sums(ps.WSC.ra_exp_all)>0,
ps.WSC.ra_exp_all)
ps.WSC.ra_exp_all <- prune_samples(sample_sums(ps.WSC.ra_exp_all)>0 ,
ps.WSC.ra_exp_all)
#Wytham- All
ps.WSC.ra_wyt_all <- subset_samples(ps.WSC.ra, System=="Wytham")
ps.WSC.ra_wyt_all <- prune_taxa(taxa_sums(ps.WSC.ra_wyt_all)>0,
ps.WSC.ra_wyt_all)
ps.WSC.ra_wyt_all <- prune_samples(sample_sums(ps.WSC.ra_wyt_all)>0 ,
ps.WSC.ra_wyt_all)

##plot the %ASVs belonging to different families (/other taxonomic levels) in
#different subsets of data; all/unique/common for wild and colony
#All SVs - captive
all_SV_taxtab_exp <- as.data.frame(tax_table(ps.WSC.ra_exp_all))
allSV_fam_exp <- as.data.frame(summary(as.factor(all_SV_taxtab_exp$Family)))
allSV_fam_exp$Family <-rownames(allSV_fam_exp)
allSV_fam_exp$SV_Group <- 'Exp_All'
colnames(allSV_fam_exp) <- c("SV_total", "Family", "SV_Group")
#allSV_fam_exp
#All SVs - wild
```

```
all_SV_taxtab_wyt <- as.data.frame(tax_table(ps.WSC.ra_wyt_all))
allSV_fam_wyt <- as.data.frame(summary(as.factor(all_SV_taxtab_wyt$Family)))
allSV_fam_wyt$Family <-rownames(allSV_fam_wyt)
allSV_fam_wyt$SV_Group <- 'Wyt_All'
colnames(allSV_fam_wyt) <- c("SV_total", "Family", "SV_Group")
#allSV_fam_wyt
##captive- unique SVs
unique_captive_SV_taxtab <-
as.data.frame(tax_table(ps.WSC.ra_exp_unique_SVs))
unique_captive_SV_fam <-
as.data.frame(summary(as.factor(unique_captive_SV_taxtab$Family)))
unique_captive_SV_fam$Family <- rownames(unique_captive_SV_fam)
unique_captive_SV_fam$SV_Group <- 'Unique_captive'
colnames(unique_captive_SV_fam) <- c("SV_total", "Family", "SV_Group")
#unique_captive_SV_fam
#wild - unique SVs
unique_wild_SV_taxtab <- as.data.frame(tax_table(ps.WSC.ra_wyt_unique_SVs))
unique_wild_SV_fam <-
as.data.frame(summary(as.factor(unique_wild_SV_taxtab$Family)))
unique_wild_SV_fam$Family <- rownames(unique_wild_SV_fam)
unique_wild_SV_fam$SV_Group <- 'Unique_wild'
colnames(unique_wild_SV_fam) <- c("SV_total", "Family", "SV_Group")
#unique_wild_SV_fam
#common SVs
common_SV_taxtab <- as.data.frame(tax_table(ps.WSC.ra_commonSVsub))
common_SV_fam <- as.data.frame(summary(as.factor(common_SV_taxtab$Family)))
common_SV_fam$Family <- rownames(common_SV_fam)
common_SV_fam$SV_Group <- 'Common'
colnames(common_SV_fam) <- c("SV_total", "Family", "SV_Group")
#common_SV_fam

#combine to get no. SVs in each family, split into all/unique/common
SV_fam_dat <- rbind( unique_wild_SV_fam, allSV_fam_wyt, common_SV_fam,
allSV_fam_exp, unique_captive_SV_fam)
SV_fam_dat <- arrange(SV_fam_dat, desc(SV_total))
all_fam_list <- SV_fam_dat$Family
head(all_fam_list, n=50)
```

```
##  [1] "Lachnospiraceae"                "Lachnospiraceae"
##  [3] "Lachnospiraceae"                "Lachnospiraceae"
##  [5] "Ruminococcaceae"                "Bacteroidales_S24-7_group"
##  [7] "Ruminococcaceae"                "Ruminococcaceae"
##  [9] "Ruminococcaceae"                "Bacteroidales_S24-7_group"
## [11] "Bacteroidales_S24-7_group"      "Clostridiales_vadinBB60_group"
## [13] "Clostridiales_vadinBB60_group" "Bacteroidales_S24-7_group"
## [15] "Clostridiales_vadinBB60_group" "Clostridiales_vadinBB60_group"
## [17] "Coriobacteriaceae"              "Coriobacteriaceae"
## [19] "Coriobacteriaceae"              "Rikenellaceae"
## [21] "Lachnospiraceae"                "Rikenellaceae"
## [23] "Rikenellaceae"                  "Lactobacillaceae"
```

```
## [25] "Desulfovibrionaceae"           "Erysipelotrichaceae"
## [27] "Lactobacillaceae"              "Enterobacteriaceae"
## [29] "Unknown_Family"                "Lactobacillaceae"
## [31] "Desulfovibrionaceae"           "Coriobacteriaceae"
## [33] "Rickettsiales_Incertae_Sedis"  "Desulfovibrionaceae"
## [35] "Unknown_Family"                "Unknown_Family"
## [37] "Peptococcaceae"                "Erysipelotrichaceae"
## [39] "Erysipelotrichaceae"           "Erysipelotrichaceae"
## [41] "Lactobacillaceae"              "Rikenellaceae"
## [43] "Enterobacteriaceae"            "Enterobacteriaceae"
## [45] "Streptococcaceae"              "Desulfovibrionaceae"
## [47] "Family_XIII"                   "Rhodospirillaceae"
## [49] "Streptococcaceae"              "Peptococcaceae"

#top10 most abundant families are;
keep_fam_list <- c("Lachnospiraceae", "Ruminococcaceae", "Bacteroidales_S24-
7_group", "Bifidobacteriaceae" ,
                   "Coriobacteriaceae" ,
"Deferribacteraceae","Rikenellaceae", "Lactobacillaceae",
                   "Desulfovibrionaceae",  "Prevotellaceae",
"Enterococcaceae")
combine_fam_list <- setdiff(all_fam_list, keep_fam_list)
#combine_fam_list
#group the rest of the families into 'other' category for plotting
SV_fam_dat$Family <- SV_fam_dat$Family %>% fct_collapse(Other =
combine_fam_list)
SV_fam_sum <-  ddply(SV_fam_dat, c( "Family", "SV_Group"), summarise,
                     N    = sum(SV_total))
SV_fam_sum$Family <- factor(SV_fam_sum$Family,
                            levels =c("Bacteroidales_S24-
7_group","Bifidobacteriaceae",
                                      "Coriobacteriaceae",
"Deferribacteraceae",
                                      "Desulfovibrionaceae",
"Enterococcaceae",
                                      "Lachnospiraceae", "Lactobacillaceae",
"Prevotellaceae" ,
                                      "Rikenellaceae", "Ruminococcaceae",
"Other"))
SV_fam_sum$SV_Group <- as.factor(SV_fam_sum$SV_Group)

#plot barchart of SVs in each group
#number of SVs in each group:
temp <- data.frame(x=c("Unique_wild", "Wyt_All", "Common","Exp_All",
"Unique_captive" ),
                   y=c(1.02, 1.02, 1.02, 1.02, 1.02),
                   size = c("n=754", "n=1574", "n=820", "n=863", "n=43"))

level_order <- c("Unique_wild", "Wyt_All", "Common", "Exp_All",
"Unique_captive")
```

```
plotbar_SVs<- ggplot(SV_fam_sum, aes( factor(x=SV_Group, levels =
level_order), y=N))  +
  geom_bar(aes(fill=Family), position='fill', stat="identity") +
  geom_text(data=temp, size=4,aes(x=x, y=y ,label=as.factor(size))) +
  labs(y= "Proportion of ASVs", x = "ASV subset") +
  theme_bw() +
  theme(axis.text=element_text(size=12), axis.title=element_text(size=12),
        title = element_text(size=12), legend.text = element_text(size=12)) +
  scale_x_discrete(labels=c( "Unique_wild" = "Unique to Wytham",
"Wyt_All"="All Wytham",
                             "Common" = "Shared",
                             "Exp_All" = "All Colony", "Unique_captive" =
"Unique to Colony")) +
  scale_fill_brewer(palette = "Paired")
plotbar_SVs
```

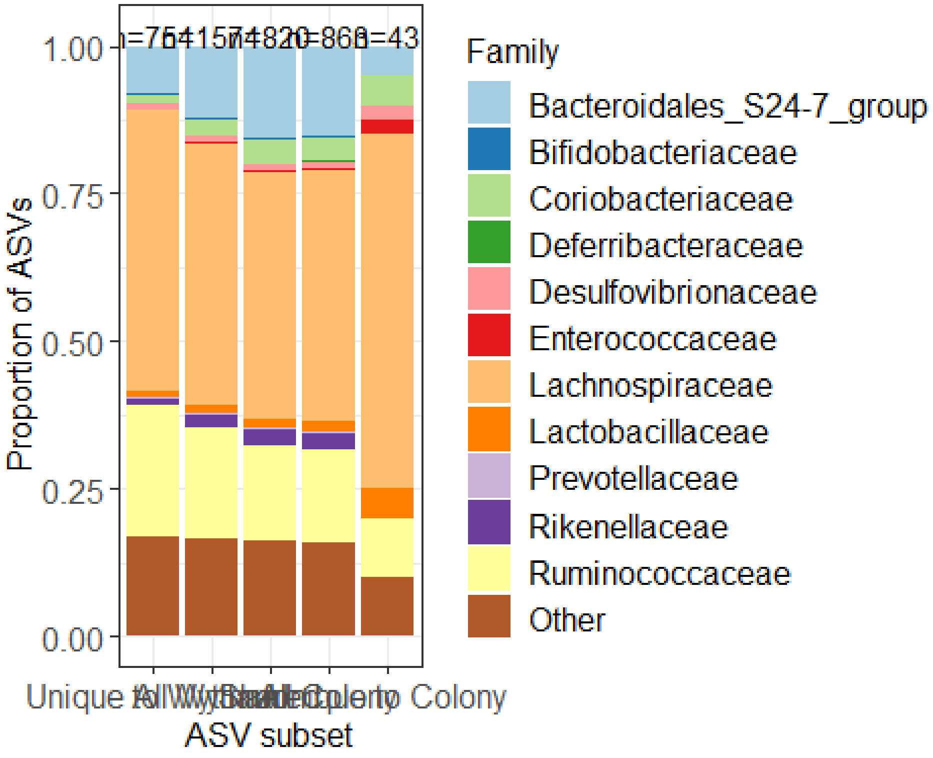

Section 4: Core microbiomes

##Deciding a ‘core’ threshold in Wytham and Silwood

```
library(phyloseq)
library(ggplot2)
library(forcats)
library(reshape2)
library(plyr) ; library(dplyr)
library(compositions)
library(microbiome)
```

```r
library(RColorBrewer)

ps.wyt.ra <- transform_sample_counts(ps.wyt.comb, function(x) x/sum(x))
ps.wyt.ra <- subset_samples(ps.wyt.ra, Species.x=="AS" & Grid=="Main")
ps.wyt.ra <- prune_taxa(taxa_sums(ps.wyt.ra) > 0, ps.wyt.ra)
ps.sil.ra <- transform_sample_counts(ps.sil.comb, function(x) x/sum(x))

det <- c(0, 0.1, 0.5, 2, 5, 20)/100
prevalences <- seq(0.1, 1, 0.1)
# det <- c( 0.001, 0.01, 0.05, 0.1, 0.3, 1, 2, 3.5,10)/100
# prevalences <- c(40,50,60,70,80,90,100)

colourCount = length(prevalences)
getPalette = colorRampPalette(brewer.pal(7, "Greys"))

#Wytham
plot_core(ps.wyt.ra, prevalences = prevalences, detections = det, plot.type =
"lineplot") +
  xlab("Relative Abundance (%)") +
  geom_point() +
  theme_bw() +
  geom_line(size=1) + ggtitle("") +
  scale_fill_manual(values = getPalette(colourCount)) +
  theme(axis.title = element_text(size=14), axis.text =
element_text(size=12),
        legend.text = element_text(size=12), legend.title =
element_text(size=14))

## Warning: Transformation introduced infinite values in continuous x-axis

## Warning: Transformation introduced infinite values in continuous x-axis

## Warning: Transformation introduced infinite values in continuous x-axis

## Warning: Transformation introduced infinite values in continuous x-axis
```

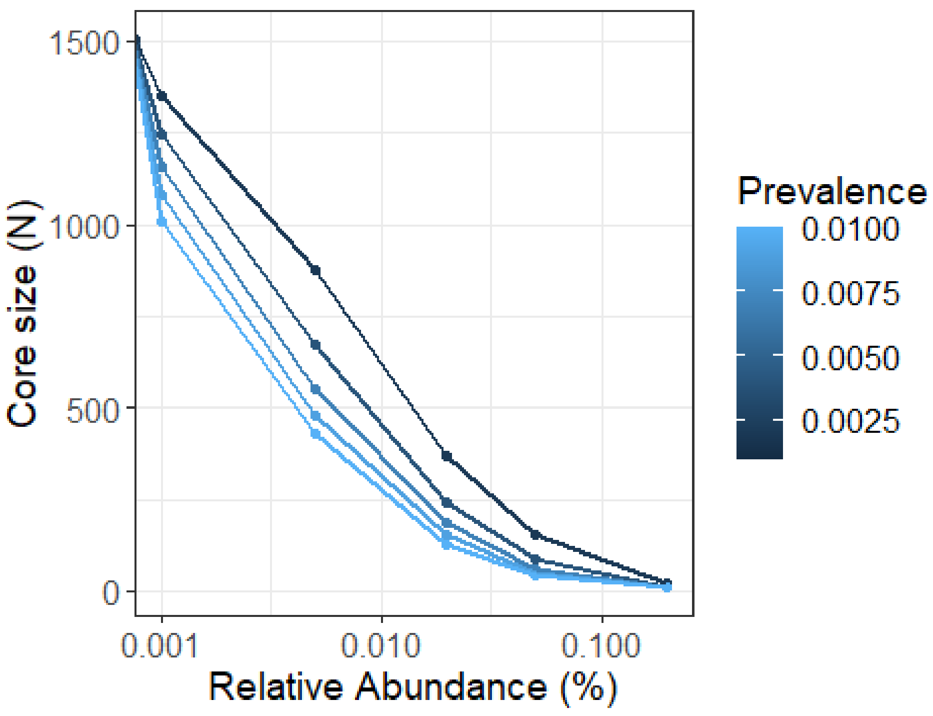


```
#Silwood
plot_core(ps.sil.ra, prevalences = prevalences, detections = det, plot.type =
"lineplot") +
  xlab("Relative Abundance (%)") +
  geom_point() +
  theme_bw() +
  geom_line(size=1) + ggtitle("") +
  scale_fill_manual(values = getPalette(colourCount)) +
  theme(axis.title = element_text(size=14), axis.text =
element_text(size=12),
        legend.text = element_text(size=12), legend.title =
element_text(size=14))

## Warning: Transformation introduced infinite values in continuous x-axis

## Warning: Transformation introduced infinite values in continuous x-axis

## Warning: Transformation introduced infinite values in continuous x-axis

## Warning: Transformation introduced infinite values in continuous x-axis
```

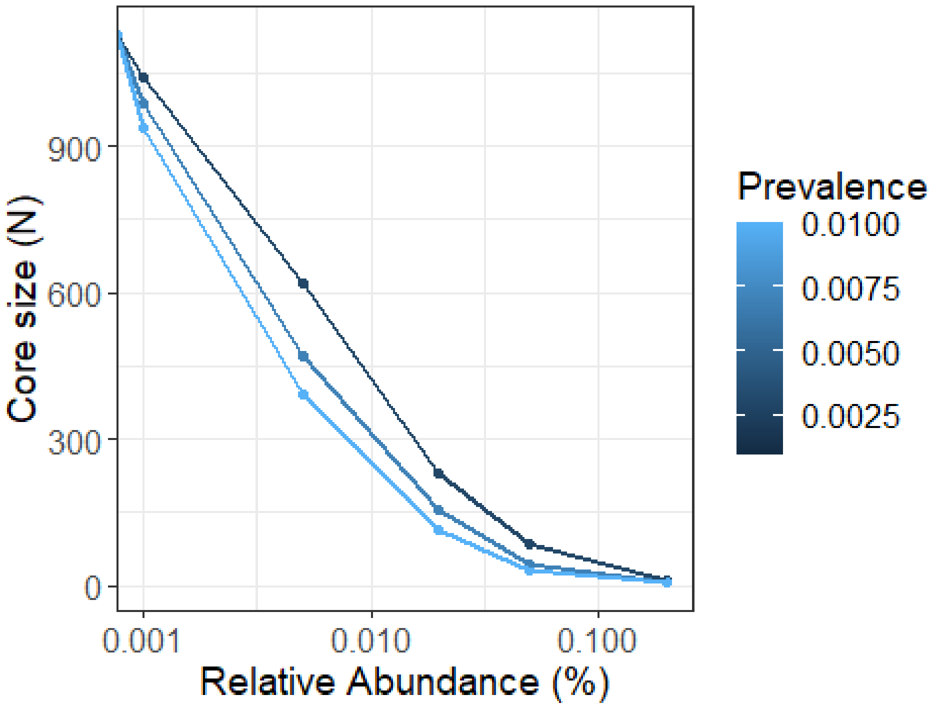


```
#subset phyloseq object to just the core taxa
#removes any OTUs that weren't present in at least than 60% samples with a
relative abudance of >0.1%
ps.wyt.core <- core(ps.wyt.ra, detection = 0.001, prevalence = 0.6)
#ps.wyt.core
#tax_table(ps.wyt.core)

ps.sil.core <- core(ps.sil.ra, detection = 0.001, prevalence = 0.6)
#ps.sil.core
#tax_table(ps.sil.core)

#get taxa names of core members
core.taxa <- as.list(taxa_names(ps.wyt.core))
core.taxa.sil <- as.list(taxa(ps.sil.core))
```

##Relationship between ASV prevalence and persistence

#Wytham

```
##convert to presence/absence
ps.wyt.bin <- transform_sample_counts(ps.wyt.ra, function(x) 1*(x>0))

####ASV persistence over time within individuals
#pick individuals caught 3 or more times
colnames(sample_data(ps.wyt.bin))[colnames(sample_data(ps.wyt.bin))=="XPIT_ta
g"] <- "Mouse.ID"
physeq_met <- as(sample_data(ps.wyt.bin), 'data.frame')
```

```
levels(sample_data(ps.wyt.bin)$Mouse.ID)[levels(sample_data(ps.wyt.bin)$Mouse
.ID)==''] <- NA
ps.wyt.bin2 <- subset_samples(ps.wyt.bin, !is.na(Mouse.ID))
mouse_recaps <-
as.data.frame(summary(as.factor(sample_data(ps.wyt.bin2)$Mouse.ID), maxsum =
300))
mouse_recaps_multi <- subset(mouse_recaps,
summary(as.factor(sample_data(ps.wyt.bin2)$Mouse.ID), maxsum = 300)>=3 )
#mean(mouse_recaps_multi$`summary(as.factor(sample_data(ps.wyt.bin2)$Mouse.ID
), maxsum = 300)`)
#sd(mouse_recaps_multi$`summary(as.factor(sample_data(ps.wyt.bin2)$Mouse.ID),
maxsum = 300)`)
mouse_recap_list <- as.list(rownames(mouse_recaps_multi))
ps.wyt.recaps <- subset_samples(ps.wyt.bin, Mouse.ID%in%mouse_recap_list)
sample_data(ps.wyt.recaps)$Mouse.ID <-
as.factor(sample_data(ps.wyt.recaps)$Mouse.ID)

recaps <- levels(sample_data(ps.wyt.recaps)$Mouse.ID)
ASV_names <- taxa_names(ps.wyt.recaps)
#length(unique(sample_data(ps.wyt.recaps)$Mouse.ID))

core.out.all <- data.frame(Mouse.no=NA, ASV_name=NA,  Persistence=NA,
N_recaps=NA)
core.out.all.out <- list()

for(i in 1:length(unique(sample_data(ps.wyt.recaps)$Mouse.ID))) {

  temp <- prune_samples(sample_data(ps.wyt.recaps)$Mouse.ID == recaps[i],
ps.wyt.recaps)       #subset phyloseq object to one mouse
  for(j in 1:length(taxa_names(ps.wyt.recaps))) {
    temp2 <- subset_taxa(temp,
taxa_names(ps.wyt.recaps)==ASV_names[j])#subset to each ASV
    prevalence <- taxa_sums(temp2)/nsamples(temp2)
    nsamp <- nsamples(temp2)
    core.out.all[j, 1] <- i
    core.out.all[j, 2] <- j
    core.out.all[j, 3] <- prevalence
    core.out.all[j, 4] <- nsamp
  }
  core.out.all.out[[i]] <- core.out.all

}

core.out.df.wyt <- bind_rows(core.out.all.out, .id = "column_label")
#summary(core.out.df.wyt) #now need to add actual Mouse.IDs and ASV names
instead of their position in the lists
recaps <- as.data.frame(recaps)
recaps <- cbind(rownames(recaps), recaps)
colnames(recaps) <- c("Mouse.no", "Mouse.ID")
```

```
#recaps
core.out.df.merge.wyt <- merge(core.out.df.wyt, recaps, by="Mouse.no", all.x
= T)
#head(core.out.df.merge.wyt)
core.out.df.merge.wyt$ASV_name <- paste0("ASV",
core.out.df.merge.wyt$ASV_name)
#not the correct ASV name, just the order in which they were sampled

#now get average persistence for each ASV acorss all mice
ASV_ave_pers.wyt <-  ddply(core.out.df.merge.wyt, c("ASV_name"), summarise,
                       N_mice    = length(Persistence),
                       mean_pers = mean(Persistence) ,
                       sd_pers   = sd(Persistence),
                       se_pers   = sd_pers / sqrt(N_mice))
#head(ASV_ave_pers.wyt)

####ASV prevalence across random samples over 100 iterations
ps.wyt.bin <- subset_samples(ps.wyt.bin, Mouse.ID!="NA")
#ps.wyt.bin

core.out.all <- data.frame(Iteration=NA, ASV_name=NA,  Prevalence=NA)
core.out.all.out <- list()
nrep <- 100

for(i in 1:nrep) {

  temp <- as.data.frame(sample_data(ps.wyt.bin) %>% group_by(Mouse.ID) %>%
sample_n(1))      #subset phyloseq object to one randomly selected sample per
mouse
  rownames(temp) <- temp$Sequence_sample_code
  temp.ps <- phyloseq(otu_table(otu_table(ps.wyt.bin)),
tax_table(tax_table(ps.wyt.bin)), sample_data(temp)) #make new ps object from
random subset
  for(j in 1:length(taxa_names(ps.wyt.bin))) {
    temp2 <- subset_taxa(temp.ps, taxa_names(ps.wyt.bin)==ASV_names[j])
#subset to each ASV
    prevalence <- taxa_sums(temp2)/nsamples(temp2)
    core.out.all[j, 1] <- i
    core.out.all[j, 2] <- j
    core.out.all[j, 3] <- prevalence
  }
  core.out.all.out[[i]] <- core.out.all

}

core.out.df.wyt <- bind_rows(core.out.all.out, .id = "column_label")
summary(core.out.df.wyt) #now need to add actual Mouse.IDs and ASV names
instead of their position in the lists
```

```
##   column_label         Iteration          ASV_name      Prevalence
##   Length:150700      Min.   :  1.00   Min.   :   1   Min.   :0.00000
##   Class :character   1st Qu.: 25.75   1st Qu.: 377   1st Qu.:0.03106
##   Mode  :character   Median : 50.50   Median : 754   Median :0.07453
##                      Mean   : 50.50   Mean   : 754   Mean   :0.14286
##                      3rd Qu.: 75.25   3rd Qu.:1131   3rd Qu.:0.18012
##                      Max.   :100.00   Max.   :1507   Max.   :1.00000

core.out.df.wyt$ASV_name <- paste0("ASV", core.out.df.wyt$ASV_name)

#now get average prevalence for each ASV acorss all mice
ASV_ave_prev_rand.wyt <-  ddply(core.out.df.wyt, c("ASV_name"), summarise,
                       N_samp    = length(Prevalence),
                       mean_prev = mean(Prevalence) ,
                       sd_prev   = sd(Prevalence),
                       se_prev   = sd_prev / sqrt(N_samp))
```

#Silwood

```
ps.sil.bin <- transform_sample_counts(ps.sil.ra, function(x) 1*(x>0))
#convert to presence/absence

###ASV persistence within individuals
physeq_met <- as(sample_data(ps.sil.ra), 'data.frame')
#colnames(physeq_met)
levels(sample_data(ps.sil.ra)$ID)[levels(sample_data(ps.sil.ra)$ID)==''] <-
NA
ps.sil2 <- subset_samples(ps.sil.ra, !is.na(ID))
#ps.sil2
mouse_recaps <- as.data.frame(summary(as.factor(sample_data(ps.sil2)$ID),
maxsum = 300))
mouse_recaps_multi <- subset(mouse_recaps,
summary(as.factor(sample_data(ps.sil2)$ID), maxsum = 300)>=3 )
#mean(mouse_recaps_multi$`summary(as.factor(sample_data(ps.sil2)$ID), maxsum
= 300)`)
#sd(mouse_recaps_multi$`summary(as.factor(sample_data(ps.sil2)$ID), maxsum =
300)`)
mouse_recap_list <- as.list(rownames(mouse_recaps_multi))
#mouse_recap_list
ps.sil.recaps <- subset_samples(ps.sil.ra, ID%in%mouse_recap_list)
ps.sil.recaps <- transform_sample_counts(ps.sil.recaps, function(x) 1*(x>0))
#summary(sample_data(ps.sil.recaps)$ID)
sample_data(ps.sil.recaps)$ID <- as.factor(sample_data(ps.sil.recaps)$ID)
recaps <- levels(sample_data(ps.sil.recaps)$ID)
#length(unique(sample_data(ps.sil.recaps)$ID))
ASV_names <- taxa_names(ps.sil.recaps)

core.out.all <- data.frame(Mouse.no=NA, ASV_name=NA,  Persistence=NA,
N_recaps=NA)
core.out.all.out <- list()
```

```
for(i in 1:length(unique(sample_data(ps.sil.recaps)$ID))) {

  temp <- prune_samples(sample_data(ps.sil.recaps)$ID == recaps[i],
ps.sil.recaps)#subset phyloseq object to one mouse
  for(j in 1:length(taxa_names(ps.sil.recaps))) {
    temp2 <- subset_taxa(temp,
taxa_names(ps.sil.recaps)==ASV_names[j])#subset to each ASV
    prevalence <- taxa_sums(temp2)/nsamples(temp2)
    nsamp <- nsamples(temp2)
    core.out.all[j, 1] <- i
    core.out.all[j, 2] <- j
    core.out.all[j, 3] <- prevalence
    core.out.all[j, 4] <- nsamp
  }
  core.out.all.out[[i]] <- core.out.all

}
#str(core.out.all.out)
core.out.df <- bind_rows(core.out.all.out, .id = "column_label")

#summary(core.out.df) #now need to add actual Mouse.IDs and ASV names instead
of their position in the lists
recaps <- as.data.frame(recaps)
recaps <- cbind(rownames(recaps), recaps)
colnames(recaps) <- c("Mouse.no", "Mouse.ID")
core.out.df.merge.sil <- merge(core.out.df, recaps, by="Mouse.no", all.x = T)
core.out.df.merge.sil$ASV_name <- paste0("ASV",
core.out.df.merge.sil$ASV_name)#not the correct ASV name, just the order in
which they were sampled

ASV_ave_pers.sil <-  ddply(core.out.df.merge.sil, c("ASV_name"), summarise,
                           N_mice    = length(Persistence),
                           mean_pers = mean(Persistence) ,
                           sd_pers   = sd(Persistence),
                           se_pers   = sd_pers / sqrt(N_mice))
head(ASV_ave_pers.sil)

##   ASV_name N_mice  mean_pers   sd_pers    se_pers
## 1     ASV1     46 1.00000000 0.0000000 0.00000000
## 2    ASV10     46 0.64844720 0.3656728 0.05391552
## 3   ASV100     46 0.69977571 0.3030617 0.04468401
## 4  ASV1000     46 0.05541753 0.1015523 0.01497308
## 5  ASV1001     46 0.13616287 0.1672575 0.02466077
## 6  ASV1002     46 0.04435818 0.1058105 0.01560090

####ASV prevalence across random samples over 100 iterations
core.out.all <- data.frame(Iteration=NA, ASV_name=NA,  Prevalence=NA)
core.out.all.out <- list()
nrep <- 100
```

```
for(i in 1:nrep) {

  temp <- as.data.frame(sample_data(ps.sil.bin) %>% group_by(ID) %>%
sample_n(1))      #subset phyloseq object to one randomly selected sample per
mouse
  rownames(temp) <- temp$Sequence_sample_code
  temp.ps <- phyloseq(otu_table(otu_table(ps.sil.bin)),
tax_table(tax_table(ps.sil.bin)), sample_data(temp)) #make new ps object from
random subset
  for(j in 1:length(taxa_names(ps.sil.bin))) {
    temp2 <- subset_taxa(temp.ps, taxa_names(ps.sil.bin)==ASV_names[j])
#subset to each ASV
    prevalence <- taxa_sums(temp2)/nsamples(temp2)
    core.out.all[j, 1] <- i
    core.out.all[j, 2] <- j
    core.out.all[j, 3] <- prevalence
  }
  core.out.all.out[[i]] <- core.out.all

}

## Warning in class(x) <- c(setdiff(subclass, tibble_class), tibble_class):
Setting
## class(x) to multiple strings ("tbl_df", "tbl", ...); result will no longer
be an
## S4 object

## Warning in class(x) <- c(setdiff(subclass, tibble_class), tibble_class):
Setting
## class(x) to multiple strings ("grouped_df", "tbl_df", ...); result will no
## longer be an S4 object

## Warning in class(x) <- c(setdiff(subclass, tibble_class), tibble_class):
Setting
## class(x) to multiple strings ("tbl_df", "tbl", ...); result will no longer
be an
## S4 object

## Warning in class(x) <- c(setdiff(subclass, tibble_class), tibble_class):
Setting
## class(x) to multiple strings ("grouped_df", "tbl_df", ...); result will no
## longer be an S4 object

## Warning in class(x) <- c(setdiff(subclass, tibble_class), tibble_class):
Setting
## class(x) to multiple strings ("tbl_df", "tbl", ...); result will no longer
be an
## S4 object
```

```
## Warning in class(x) <- c(setdiff(subclass, tibble_class), tibble_class):
Setting
## class(x) to multiple strings ("grouped_df", "tbl_df", ...); result will no
## longer be an S4 object

## Warning in class(x) <- c(setdiff(subclass, tibble_class), tibble_class):
Setting
## class(x) to multiple strings ("tbl_df", "tbl", ...); result will no longer
be an
## S4 object

## Warning in class(x) <- c(setdiff(subclass, tibble_class), tibble_class):
Setting
## class(x) to multiple strings ("grouped_df", "tbl_df", ...); result will no
## longer be an S4 object

## Warning in class(x) <- c(setdiff(subclass, tibble_class), tibble_class):
Setting
## class(x) to multiple strings ("tbl_df", "tbl", ...); result will no longer
be an
## S4 object

## Warning in class(x) <- c(setdiff(subclass, tibble_class), tibble_class):
Setting
## class(x) to multiple strings ("grouped_df", "tbl_df", ...); result will no
## longer be an S4 object

## Warning in class(x) <- c(setdiff(subclass, tibble_class), tibble_class):
Setting
## class(x) to multiple strings ("tbl_df", "tbl", ...); result will no longer
be an
## S4 object

## Warning in class(x) <- c(setdiff(subclass, tibble_class), tibble_class):
Setting
## class(x) to multiple strings ("grouped_df", "tbl_df", ...); result will no
## longer be an S4 object

## Warning in class(x) <- c(setdiff(subclass, tibble_class), tibble_class):
Setting
## class(x) to multiple strings ("tbl_df", "tbl", ...); result will no longer
be an
## S4 object

## Warning in class(x) <- c(setdiff(subclass, tibble_class), tibble_class):
Setting
## class(x) to multiple strings ("grouped_df", "tbl_df", ...); result will no
## longer be an S4 object

## Warning in class(x) <- c(setdiff(subclass, tibble_class), tibble_class):
Setting
## class(x) to multiple strings ("tbl_df", "tbl", ...); result will no longer
```

```
be an
## S4 object

## Warning in class(x) <- c(setdiff(subclass, tibble_class), tibble_class):
Setting
## class(x) to multiple strings ("grouped_df", "tbl_df", ...); result will no
## longer be an S4 object

## Warning in class(x) <- c(setdiff(subclass, tibble_class), tibble_class):
Setting
## class(x) to multiple strings ("tbl_df", "tbl", ...); result will no longer
be an
## S4 object

## Warning in class(x) <- c(setdiff(subclass, tibble_class), tibble_class):
Setting
## class(x) to multiple strings ("grouped_df", "tbl_df", ...); result will no
## longer be an S4 object

## Warning in class(x) <- c(setdiff(subclass, tibble_class), tibble_class):
Setting
## class(x) to multiple strings ("tbl_df", "tbl", ...); result will no longer
be an
## S4 object

## Warning in class(x) <- c(setdiff(subclass, tibble_class), tibble_class):
Setting
## class(x) to multiple strings ("grouped_df", "tbl_df", ...); result will no
## longer be an S4 object

## Warning in class(x) <- c(setdiff(subclass, tibble_class), tibble_class):
Setting
## class(x) to multiple strings ("tbl_df", "tbl", ...); result will no longer
be an
## S4 object

## Warning in class(x) <- c(setdiff(subclass, tibble_class), tibble_class):
Setting
## class(x) to multiple strings ("grouped_df", "tbl_df", ...); result will no
## longer be an S4 object

## Warning in class(x) <- c(setdiff(subclass, tibble_class), tibble_class):
Setting
## class(x) to multiple strings ("tbl_df", "tbl", ...); result will no longer
be an
## S4 object

## Warning in class(x) <- c(setdiff(subclass, tibble_class), tibble_class):
Setting
## class(x) to multiple strings ("grouped_df", "tbl_df", ...); result will no
## longer be an S4 object
```

```
## Warning in class(x) <- c(setdiff(subclass, tibble_class), tibble_class):
Setting
## class(x) to multiple strings ("tbl_df", "tbl", ...); result will no longer
be an
## S4 object

## Warning in class(x) <- c(setdiff(subclass, tibble_class), tibble_class):
Setting
## class(x) to multiple strings ("grouped_df", "tbl_df", ...); result will no
## longer be an S4 object

## Warning in class(x) <- c(setdiff(subclass, tibble_class), tibble_class):
Setting
## class(x) to multiple strings ("tbl_df", "tbl", ...); result will no longer
be an
## S4 object

## Warning in class(x) <- c(setdiff(subclass, tibble_class), tibble_class):
Setting
## class(x) to multiple strings ("grouped_df", "tbl_df", ...); result will no
## longer be an S4 object

## Warning in class(x) <- c(setdiff(subclass, tibble_class), tibble_class):
Setting
## class(x) to multiple strings ("tbl_df", "tbl", ...); result will no longer
be an
## S4 object

## Warning in class(x) <- c(setdiff(subclass, tibble_class), tibble_class):
Setting
## class(x) to multiple strings ("grouped_df", "tbl_df", ...); result will no
## longer be an S4 object

## Warning in class(x) <- c(setdiff(subclass, tibble_class), tibble_class):
Setting
## class(x) to multiple strings ("tbl_df", "tbl", ...); result will no longer
be an
## S4 object

## Warning in class(x) <- c(setdiff(subclass, tibble_class), tibble_class):
Setting
## class(x) to multiple strings ("grouped_df", "tbl_df", ...); result will no
## longer be an S4 object

## Warning in class(x) <- c(setdiff(subclass, tibble_class), tibble_class):
Setting
## class(x) to multiple strings ("tbl_df", "tbl", ...); result will no longer
be an
## S4 object

## Warning in class(x) <- c(setdiff(subclass, tibble_class), tibble_class):
Setting
```

```
## class(x) to multiple strings ("grouped_df", "tbl_df", ...); result will no
## longer be an S4 object

## Warning in class(x) <- c(setdiff(subclass, tibble_class), tibble_class):
Setting
## class(x) to multiple strings ("tbl_df", "tbl", ...); result will no longer
be an
## S4 object

## Warning in class(x) <- c(setdiff(subclass, tibble_class), tibble_class):
Setting
## class(x) to multiple strings ("grouped_df", "tbl_df", ...); result will no
## longer be an S4 object

## Warning in class(x) <- c(setdiff(subclass, tibble_class), tibble_class):
Setting
## class(x) to multiple strings ("tbl_df", "tbl", ...); result will no longer
be an
## S4 object

## Warning in class(x) <- c(setdiff(subclass, tibble_class), tibble_class):
Setting
## class(x) to multiple strings ("grouped_df", "tbl_df", ...); result will no
## longer be an S4 object

## Warning in class(x) <- c(setdiff(subclass, tibble_class), tibble_class):
Setting
## class(x) to multiple strings ("tbl_df", "tbl", ...); result will no longer
be an
## S4 object

## Warning in class(x) <- c(setdiff(subclass, tibble_class), tibble_class):
Setting
## class(x) to multiple strings ("grouped_df", "tbl_df", ...); result will no
## longer be an S4 object

## Warning in class(x) <- c(setdiff(subclass, tibble_class), tibble_class):
Setting
## class(x) to multiple strings ("tbl_df", "tbl", ...); result will no longer
be an
## S4 object

## Warning in class(x) <- c(setdiff(subclass, tibble_class), tibble_class):
Setting
## class(x) to multiple strings ("grouped_df", "tbl_df", ...); result will no
## longer be an S4 object

## Warning in class(x) <- c(setdiff(subclass, tibble_class), tibble_class):
Setting
## class(x) to multiple strings ("tbl_df", "tbl", ...); result will no longer
be an
## S4 object
```

```
## Warning in class(x) <- c(setdiff(subclass, tibble_class), tibble_class):
Setting
## class(x) to multiple strings ("grouped_df", "tbl_df", ...); result will no
## longer be an S4 object

## Warning in class(x) <- c(setdiff(subclass, tibble_class), tibble_class):
Setting
## class(x) to multiple strings ("tbl_df", "tbl", ...); result will no longer
be an
## S4 object

## Warning in class(x) <- c(setdiff(subclass, tibble_class), tibble_class):
Setting
## class(x) to multiple strings ("grouped_df", "tbl_df", ...); result will no
## longer be an S4 object

## Warning in class(x) <- c(setdiff(subclass, tibble_class), tibble_class):
Setting
## class(x) to multiple strings ("tbl_df", "tbl", ...); result will no longer
be an
## S4 object

## Warning in class(x) <- c(setdiff(subclass, tibble_class), tibble_class):
Setting
## class(x) to multiple strings ("grouped_df", "tbl_df", ...); result will no
## longer be an S4 object

## Warning in class(x) <- c(setdiff(subclass, tibble_class), tibble_class):
Setting
## class(x) to multiple strings ("tbl_df", "tbl", ...); result will no longer
be an
## S4 object

## Warning in class(x) <- c(setdiff(subclass, tibble_class), tibble_class):
Setting
## class(x) to multiple strings ("grouped_df", "tbl_df", ...); result will no
## longer be an S4 object

## Warning in class(x) <- c(setdiff(subclass, tibble_class), tibble_class):
Setting
## class(x) to multiple strings ("tbl_df", "tbl", ...); result will no longer
be an
## S4 object

## Warning in class(x) <- c(setdiff(subclass, tibble_class), tibble_class):
Setting
## class(x) to multiple strings ("grouped_df", "tbl_df", ...); result will no
## longer be an S4 object

## Warning in class(x) <- c(setdiff(subclass, tibble_class), tibble_class):
Setting
## class(x) to multiple strings ("tbl_df", "tbl", ...); result will no longer
```

```
be an
## S4 object

## Warning in class(x) <- c(setdiff(subclass, tibble_class), tibble_class):
Setting
## class(x) to multiple strings ("grouped_df", "tbl_df", ...); result will no
## longer be an S4 object

## Warning in class(x) <- c(setdiff(subclass, tibble_class), tibble_class):
Setting
## class(x) to multiple strings ("tbl_df", "tbl", ...); result will no longer
be an
## S4 object

## Warning in class(x) <- c(setdiff(subclass, tibble_class), tibble_class):
Setting
## class(x) to multiple strings ("grouped_df", "tbl_df", ...); result will no
## longer be an S4 object

## Warning in class(x) <- c(setdiff(subclass, tibble_class), tibble_class):
Setting
## class(x) to multiple strings ("tbl_df", "tbl", ...); result will no longer
be an
## S4 object

## Warning in class(x) <- c(setdiff(subclass, tibble_class), tibble_class):
Setting
## class(x) to multiple strings ("grouped_df", "tbl_df", ...); result will no
## longer be an S4 object

## Warning in class(x) <- c(setdiff(subclass, tibble_class), tibble_class):
Setting
## class(x) to multiple strings ("tbl_df", "tbl", ...); result will no longer
be an
## S4 object

## Warning in class(x) <- c(setdiff(subclass, tibble_class), tibble_class):
Setting
## class(x) to multiple strings ("grouped_df", "tbl_df", ...); result will no
## longer be an S4 object

## Warning in class(x) <- c(setdiff(subclass, tibble_class), tibble_class):
Setting
## class(x) to multiple strings ("tbl_df", "tbl", ...); result will no longer
be an
## S4 object

## Warning in class(x) <- c(setdiff(subclass, tibble_class), tibble_class):
Setting
## class(x) to multiple strings ("grouped_df", "tbl_df", ...); result will no
## longer be an S4 object
```

```
## Warning in class(x) <- c(setdiff(subclass, tibble_class), tibble_class):
Setting
## class(x) to multiple strings ("tbl_df", "tbl", ...); result will no longer
be an
## S4 object

## Warning in class(x) <- c(setdiff(subclass, tibble_class), tibble_class):
Setting
## class(x) to multiple strings ("grouped_df", "tbl_df", ...); result will no
## longer be an S4 object

## Warning in class(x) <- c(setdiff(subclass, tibble_class), tibble_class):
Setting
## class(x) to multiple strings ("tbl_df", "tbl", ...); result will no longer
be an
## S4 object

## Warning in class(x) <- c(setdiff(subclass, tibble_class), tibble_class):
Setting
## class(x) to multiple strings ("grouped_df", "tbl_df", ...); result will no
## longer be an S4 object

## Warning in class(x) <- c(setdiff(subclass, tibble_class), tibble_class):
Setting
## class(x) to multiple strings ("tbl_df", "tbl", ...); result will no longer
be an
## S4 object

## Warning in class(x) <- c(setdiff(subclass, tibble_class), tibble_class):
Setting
## class(x) to multiple strings ("grouped_df", "tbl_df", ...); result will no
## longer be an S4 object

## Warning in class(x) <- c(setdiff(subclass, tibble_class), tibble_class):
Setting
## class(x) to multiple strings ("tbl_df", "tbl", ...); result will no longer
be an
## S4 object

## Warning in class(x) <- c(setdiff(subclass, tibble_class), tibble_class):
Setting
## class(x) to multiple strings ("grouped_df", "tbl_df", ...); result will no
## longer be an S4 object

## Warning in class(x) <- c(setdiff(subclass, tibble_class), tibble_class):
Setting
## class(x) to multiple strings ("tbl_df", "tbl", ...); result will no longer
be an
## S4 object

## Warning in class(x) <- c(setdiff(subclass, tibble_class), tibble_class):
Setting
```

```
## class(x) to multiple strings ("grouped_df", "tbl_df", ...); result will no
## longer be an S4 object

## Warning in class(x) <- c(setdiff(subclass, tibble_class), tibble_class):
Setting
## class(x) to multiple strings ("tbl_df", "tbl", ...); result will no longer
be an
## S4 object

## Warning in class(x) <- c(setdiff(subclass, tibble_class), tibble_class):
Setting
## class(x) to multiple strings ("grouped_df", "tbl_df", ...); result will no
## longer be an S4 object

## Warning in class(x) <- c(setdiff(subclass, tibble_class), tibble_class):
Setting
## class(x) to multiple strings ("tbl_df", "tbl", ...); result will no longer
be an
## S4 object

## Warning in class(x) <- c(setdiff(subclass, tibble_class), tibble_class):
Setting
## class(x) to multiple strings ("grouped_df", "tbl_df", ...); result will no
## longer be an S4 object

## Warning in class(x) <- c(setdiff(subclass, tibble_class), tibble_class):
Setting
## class(x) to multiple strings ("tbl_df", "tbl", ...); result will no longer
be an
## S4 object

## Warning in class(x) <- c(setdiff(subclass, tibble_class), tibble_class):
Setting
## class(x) to multiple strings ("grouped_df", "tbl_df", ...); result will no
## longer be an S4 object

## Warning in class(x) <- c(setdiff(subclass, tibble_class), tibble_class):
Setting
## class(x) to multiple strings ("tbl_df", "tbl", ...); result will no longer
be an
## S4 object

## Warning in class(x) <- c(setdiff(subclass, tibble_class), tibble_class):
Setting
## class(x) to multiple strings ("grouped_df", "tbl_df", ...); result will no
## longer be an S4 object

## Warning in class(x) <- c(setdiff(subclass, tibble_class), tibble_class):
Setting
## class(x) to multiple strings ("tbl_df", "tbl", ...); result will no longer
be an
## S4 object
```

```
## Warning in class(x) <- c(setdiff(subclass, tibble_class), tibble_class):
Setting
## class(x) to multiple strings ("grouped_df", "tbl_df", ...); result will no
## longer be an S4 object

## Warning in class(x) <- c(setdiff(subclass, tibble_class), tibble_class):
Setting
## class(x) to multiple strings ("tbl_df", "tbl", ...); result will no longer
be an
## S4 object

## Warning in class(x) <- c(setdiff(subclass, tibble_class), tibble_class):
Setting
## class(x) to multiple strings ("grouped_df", "tbl_df", ...); result will no
## longer be an S4 object

## Warning in class(x) <- c(setdiff(subclass, tibble_class), tibble_class):
Setting
## class(x) to multiple strings ("tbl_df", "tbl", ...); result will no longer
be an
## S4 object

## Warning in class(x) <- c(setdiff(subclass, tibble_class), tibble_class):
Setting
## class(x) to multiple strings ("grouped_df", "tbl_df", ...); result will no
## longer be an S4 object

## Warning in class(x) <- c(setdiff(subclass, tibble_class), tibble_class):
Setting
## class(x) to multiple strings ("tbl_df", "tbl", ...); result will no longer
be an
## S4 object

## Warning in class(x) <- c(setdiff(subclass, tibble_class), tibble_class):
Setting
## class(x) to multiple strings ("grouped_df", "tbl_df", ...); result will no
## longer be an S4 object

## Warning in class(x) <- c(setdiff(subclass, tibble_class), tibble_class):
Setting
## class(x) to multiple strings ("tbl_df", "tbl", ...); result will no longer
be an
## S4 object

## Warning in class(x) <- c(setdiff(subclass, tibble_class), tibble_class):
Setting
## class(x) to multiple strings ("grouped_df", "tbl_df", ...); result will no
## longer be an S4 object

## Warning in class(x) <- c(setdiff(subclass, tibble_class), tibble_class):
Setting
## class(x) to multiple strings ("tbl_df", "tbl", ...); result will no longer
```

```
be an
## S4 object

## Warning in class(x) <- c(setdiff(subclass, tibble_class), tibble_class):
Setting
## class(x) to multiple strings ("grouped_df", "tbl_df", ...); result will no
## longer be an S4 object

## Warning in class(x) <- c(setdiff(subclass, tibble_class), tibble_class):
Setting
## class(x) to multiple strings ("tbl_df", "tbl", ...); result will no longer
be an
## S4 object

## Warning in class(x) <- c(setdiff(subclass, tibble_class), tibble_class):
Setting
## class(x) to multiple strings ("grouped_df", "tbl_df", ...); result will no
## longer be an S4 object

## Warning in class(x) <- c(setdiff(subclass, tibble_class), tibble_class):
Setting
## class(x) to multiple strings ("tbl_df", "tbl", ...); result will no longer
be an
## S4 object

## Warning in class(x) <- c(setdiff(subclass, tibble_class), tibble_class):
Setting
## class(x) to multiple strings ("grouped_df", "tbl_df", ...); result will no
## longer be an S4 object

## Warning in class(x) <- c(setdiff(subclass, tibble_class), tibble_class):
Setting
## class(x) to multiple strings ("tbl_df", "tbl", ...); result will no longer
be an
## S4 object

## Warning in class(x) <- c(setdiff(subclass, tibble_class), tibble_class):
Setting
## class(x) to multiple strings ("grouped_df", "tbl_df", ...); result will no
## longer be an S4 object

## Warning in class(x) <- c(setdiff(subclass, tibble_class), tibble_class):
Setting
## class(x) to multiple strings ("tbl_df", "tbl", ...); result will no longer
be an
## S4 object

## Warning in class(x) <- c(setdiff(subclass, tibble_class), tibble_class):
Setting
## class(x) to multiple strings ("grouped_df", "tbl_df", ...); result will no
## longer be an S4 object
```

```
## Warning in class(x) <- c(setdiff(subclass, tibble_class), tibble_class):
Setting
## class(x) to multiple strings ("tbl_df", "tbl", ...); result will no longer
be an
## S4 object

## Warning in class(x) <- c(setdiff(subclass, tibble_class), tibble_class):
Setting
## class(x) to multiple strings ("grouped_df", "tbl_df", ...); result will no
## longer be an S4 object

## Warning in class(x) <- c(setdiff(subclass, tibble_class), tibble_class):
Setting
## class(x) to multiple strings ("tbl_df", "tbl", ...); result will no longer
be an
## S4 object

## Warning in class(x) <- c(setdiff(subclass, tibble_class), tibble_class):
Setting
## class(x) to multiple strings ("grouped_df", "tbl_df", ...); result will no
## longer be an S4 object

## Warning in class(x) <- c(setdiff(subclass, tibble_class), tibble_class):
Setting
## class(x) to multiple strings ("tbl_df", "tbl", ...); result will no longer
be an
## S4 object

## Warning in class(x) <- c(setdiff(subclass, tibble_class), tibble_class):
Setting
## class(x) to multiple strings ("grouped_df", "tbl_df", ...); result will no
## longer be an S4 object

## Warning in class(x) <- c(setdiff(subclass, tibble_class), tibble_class):
Setting
## class(x) to multiple strings ("tbl_df", "tbl", ...); result will no longer
be an
## S4 object

## Warning in class(x) <- c(setdiff(subclass, tibble_class), tibble_class):
Setting
## class(x) to multiple strings ("grouped_df", "tbl_df", ...); result will no
## longer be an S4 object

## Warning in class(x) <- c(setdiff(subclass, tibble_class), tibble_class):
Setting
## class(x) to multiple strings ("tbl_df", "tbl", ...); result will no longer
be an
## S4 object

## Warning in class(x) <- c(setdiff(subclass, tibble_class), tibble_class):
Setting
```

```
## class(x) to multiple strings ("grouped_df", "tbl_df", ...); result will no
## longer be an S4 object

## Warning in class(x) <- c(setdiff(subclass, tibble_class), tibble_class):
Setting
## class(x) to multiple strings ("tbl_df", "tbl", ...); result will no longer
be an
## S4 object

## Warning in class(x) <- c(setdiff(subclass, tibble_class), tibble_class):
Setting
## class(x) to multiple strings ("grouped_df", "tbl_df", ...); result will no
## longer be an S4 object

## Warning in class(x) <- c(setdiff(subclass, tibble_class), tibble_class):
Setting
## class(x) to multiple strings ("tbl_df", "tbl", ...); result will no longer
be an
## S4 object

## Warning in class(x) <- c(setdiff(subclass, tibble_class), tibble_class):
Setting
## class(x) to multiple strings ("grouped_df", "tbl_df", ...); result will no
## longer be an S4 object

## Warning in class(x) <- c(setdiff(subclass, tibble_class), tibble_class):
Setting
## class(x) to multiple strings ("tbl_df", "tbl", ...); result will no longer
be an
## S4 object

## Warning in class(x) <- c(setdiff(subclass, tibble_class), tibble_class):
Setting
## class(x) to multiple strings ("grouped_df", "tbl_df", ...); result will no
## longer be an S4 object

## Warning in class(x) <- c(setdiff(subclass, tibble_class), tibble_class):
Setting
## class(x) to multiple strings ("tbl_df", "tbl", ...); result will no longer
be an
## S4 object

## Warning in class(x) <- c(setdiff(subclass, tibble_class), tibble_class):
Setting
## class(x) to multiple strings ("grouped_df", "tbl_df", ...); result will no
## longer be an S4 object

## Warning in class(x) <- c(setdiff(subclass, tibble_class), tibble_class):
Setting
## class(x) to multiple strings ("tbl_df", "tbl", ...); result will no longer
be an
## S4 object
```

```
## Warning in class(x) <- c(setdiff(subclass, tibble_class), tibble_class):
Setting
## class(x) to multiple strings ("grouped_df", "tbl_df", ...); result will no
## longer be an S4 object

## Warning in class(x) <- c(setdiff(subclass, tibble_class), tibble_class):
Setting
## class(x) to multiple strings ("tbl_df", "tbl", ...); result will no longer
be an
## S4 object

## Warning in class(x) <- c(setdiff(subclass, tibble_class), tibble_class):
Setting
## class(x) to multiple strings ("grouped_df", "tbl_df", ...); result will no
## longer be an S4 object

## Warning in class(x) <- c(setdiff(subclass, tibble_class), tibble_class):
Setting
## class(x) to multiple strings ("tbl_df", "tbl", ...); result will no longer
be an
## S4 object

## Warning in class(x) <- c(setdiff(subclass, tibble_class), tibble_class):
Setting
## class(x) to multiple strings ("grouped_df", "tbl_df", ...); result will no
## longer be an S4 object

## Warning in class(x) <- c(setdiff(subclass, tibble_class), tibble_class):
Setting
## class(x) to multiple strings ("tbl_df", "tbl", ...); result will no longer
be an
## S4 object

## Warning in class(x) <- c(setdiff(subclass, tibble_class), tibble_class):
Setting
## class(x) to multiple strings ("grouped_df", "tbl_df", ...); result will no
## longer be an S4 object

## Warning in class(x) <- c(setdiff(subclass, tibble_class), tibble_class):
Setting
## class(x) to multiple strings ("tbl_df", "tbl", ...); result will no longer
be an
## S4 object

## Warning in class(x) <- c(setdiff(subclass, tibble_class), tibble_class):
Setting
## class(x) to multiple strings ("grouped_df", "tbl_df", ...); result will no
## longer be an S4 object

## Warning in class(x) <- c(setdiff(subclass, tibble_class), tibble_class):
Setting
## class(x) to multiple strings ("tbl_df", "tbl", ...); result will no longer
```

```
be an
## S4 object

## Warning in class(x) <- c(setdiff(subclass, tibble_class), tibble_class):
Setting
## class(x) to multiple strings ("grouped_df", "tbl_df", ...); result will no
## longer be an S4 object

## Warning in class(x) <- c(setdiff(subclass, tibble_class), tibble_class):
Setting
## class(x) to multiple strings ("tbl_df", "tbl", ...); result will no longer
be an
## S4 object

## Warning in class(x) <- c(setdiff(subclass, tibble_class), tibble_class):
Setting
## class(x) to multiple strings ("grouped_df", "tbl_df", ...); result will no
## longer be an S4 object

## Warning in class(x) <- c(setdiff(subclass, tibble_class), tibble_class):
Setting
## class(x) to multiple strings ("tbl_df", "tbl", ...); result will no longer
be an
## S4 object

## Warning in class(x) <- c(setdiff(subclass, tibble_class), tibble_class):
Setting
## class(x) to multiple strings ("grouped_df", "tbl_df", ...); result will no
## longer be an S4 object

## Warning in class(x) <- c(setdiff(subclass, tibble_class), tibble_class):
Setting
## class(x) to multiple strings ("tbl_df", "tbl", ...); result will no longer
be an
## S4 object

## Warning in class(x) <- c(setdiff(subclass, tibble_class), tibble_class):
Setting
## class(x) to multiple strings ("grouped_df", "tbl_df", ...); result will no
## longer be an S4 object

## Warning in class(x) <- c(setdiff(subclass, tibble_class), tibble_class):
Setting
## class(x) to multiple strings ("tbl_df", "tbl", ...); result will no longer
be an
## S4 object

## Warning in class(x) <- c(setdiff(subclass, tibble_class), tibble_class):
Setting
## class(x) to multiple strings ("grouped_df", "tbl_df", ...); result will no
## longer be an S4 object
```

```
## Warning in class(x) <- c(setdiff(subclass, tibble_class), tibble_class):
Setting
## class(x) to multiple strings ("tbl_df", "tbl", ...); result will no longer
be an
## S4 object

## Warning in class(x) <- c(setdiff(subclass, tibble_class), tibble_class):
Setting
## class(x) to multiple strings ("grouped_df", "tbl_df", ...); result will no
## longer be an S4 object

## Warning in class(x) <- c(setdiff(subclass, tibble_class), tibble_class):
Setting
## class(x) to multiple strings ("tbl_df", "tbl", ...); result will no longer
be an
## S4 object

## Warning in class(x) <- c(setdiff(subclass, tibble_class), tibble_class):
Setting
## class(x) to multiple strings ("grouped_df", "tbl_df", ...); result will no
## longer be an S4 object

## Warning in class(x) <- c(setdiff(subclass, tibble_class), tibble_class):
Setting
## class(x) to multiple strings ("tbl_df", "tbl", ...); result will no longer
be an
## S4 object

## Warning in class(x) <- c(setdiff(subclass, tibble_class), tibble_class):
Setting
## class(x) to multiple strings ("grouped_df", "tbl_df", ...); result will no
## longer be an S4 object

## Warning in class(x) <- c(setdiff(subclass, tibble_class), tibble_class):
Setting
## class(x) to multiple strings ("tbl_df", "tbl", ...); result will no longer
be an
## S4 object

## Warning in class(x) <- c(setdiff(subclass, tibble_class), tibble_class):
Setting
## class(x) to multiple strings ("grouped_df", "tbl_df", ...); result will no
## longer be an S4 object

## Warning in class(x) <- c(setdiff(subclass, tibble_class), tibble_class):
Setting
## class(x) to multiple strings ("tbl_df", "tbl", ...); result will no longer
be an
## S4 object

## Warning in class(x) <- c(setdiff(subclass, tibble_class), tibble_class):
Setting
```

```
## class(x) to multiple strings ("grouped_df", "tbl_df", ...); result will no
## longer be an S4 object

## Warning in class(x) <- c(setdiff(subclass, tibble_class), tibble_class):
Setting
## class(x) to multiple strings ("tbl_df", "tbl", ...); result will no longer
be an
## S4 object

## Warning in class(x) <- c(setdiff(subclass, tibble_class), tibble_class):
Setting
## class(x) to multiple strings ("grouped_df", "tbl_df", ...); result will no
## longer be an S4 object

## Warning in class(x) <- c(setdiff(subclass, tibble_class), tibble_class):
Setting
## class(x) to multiple strings ("tbl_df", "tbl", ...); result will no longer
be an
## S4 object

## Warning in class(x) <- c(setdiff(subclass, tibble_class), tibble_class):
Setting
## class(x) to multiple strings ("grouped_df", "tbl_df", ...); result will no
## longer be an S4 object

## Warning in class(x) <- c(setdiff(subclass, tibble_class), tibble_class):
Setting
## class(x) to multiple strings ("tbl_df", "tbl", ...); result will no longer
be an
## S4 object

## Warning in class(x) <- c(setdiff(subclass, tibble_class), tibble_class):
Setting
## class(x) to multiple strings ("grouped_df", "tbl_df", ...); result will no
## longer be an S4 object

## Warning in class(x) <- c(setdiff(subclass, tibble_class), tibble_class):
Setting
## class(x) to multiple strings ("tbl_df", "tbl", ...); result will no longer
be an
## S4 object

## Warning in class(x) <- c(setdiff(subclass, tibble_class), tibble_class):
Setting
## class(x) to multiple strings ("grouped_df", "tbl_df", ...); result will no
## longer be an S4 object

## Warning in class(x) <- c(setdiff(subclass, tibble_class), tibble_class):
Setting
## class(x) to multiple strings ("tbl_df", "tbl", ...); result will no longer
be an
## S4 object
```

```
## Warning in class(x) <- c(setdiff(subclass, tibble_class), tibble_class):
Setting
## class(x) to multiple strings ("grouped_df", "tbl_df", ...); result will no
## longer be an S4 object

## Warning in class(x) <- c(setdiff(subclass, tibble_class), tibble_class):
Setting
## class(x) to multiple strings ("tbl_df", "tbl", ...); result will no longer
be an
## S4 object

## Warning in class(x) <- c(setdiff(subclass, tibble_class), tibble_class):
Setting
## class(x) to multiple strings ("grouped_df", "tbl_df", ...); result will no
## longer be an S4 object

## Warning in class(x) <- c(setdiff(subclass, tibble_class), tibble_class):
Setting
## class(x) to multiple strings ("tbl_df", "tbl", ...); result will no longer
be an
## S4 object

## Warning in class(x) <- c(setdiff(subclass, tibble_class), tibble_class):
Setting
## class(x) to multiple strings ("grouped_df", "tbl_df", ...); result will no
## longer be an S4 object

## Warning in class(x) <- c(setdiff(subclass, tibble_class), tibble_class):
Setting
## class(x) to multiple strings ("tbl_df", "tbl", ...); result will no longer
be an
## S4 object

## Warning in class(x) <- c(setdiff(subclass, tibble_class), tibble_class):
Setting
## class(x) to multiple strings ("grouped_df", "tbl_df", ...); result will no
## longer be an S4 object

## Warning in class(x) <- c(setdiff(subclass, tibble_class), tibble_class):
Setting
## class(x) to multiple strings ("tbl_df", "tbl", ...); result will no longer
be an
## S4 object

## Warning in class(x) <- c(setdiff(subclass, tibble_class), tibble_class):
Setting
## class(x) to multiple strings ("grouped_df", "tbl_df", ...); result will no
## longer be an S4 object

## Warning in class(x) <- c(setdiff(subclass, tibble_class), tibble_class):
Setting
## class(x) to multiple strings ("tbl_df", "tbl", ...); result will no longer
```

```
be an
## S4 object

## Warning in class(x) <- c(setdiff(subclass, tibble_class), tibble_class):
Setting
## class(x) to multiple strings ("grouped_df", "tbl_df", ...); result will no
## longer be an S4 object

## Warning in class(x) <- c(setdiff(subclass, tibble_class), tibble_class):
Setting
## class(x) to multiple strings ("tbl_df", "tbl", ...); result will no longer
be an
## S4 object

## Warning in class(x) <- c(setdiff(subclass, tibble_class), tibble_class):
Setting
## class(x) to multiple strings ("grouped_df", "tbl_df", ...); result will no
## longer be an S4 object

## Warning in class(x) <- c(setdiff(subclass, tibble_class), tibble_class):
Setting
## class(x) to multiple strings ("tbl_df", "tbl", ...); result will no longer
be an
## S4 object

## Warning in class(x) <- c(setdiff(subclass, tibble_class), tibble_class):
Setting
## class(x) to multiple strings ("grouped_df", "tbl_df", ...); result will no
## longer be an S4 object

## Warning in class(x) <- c(setdiff(subclass, tibble_class), tibble_class):
Setting
## class(x) to multiple strings ("tbl_df", "tbl", ...); result will no longer
be an
## S4 object

## Warning in class(x) <- c(setdiff(subclass, tibble_class), tibble_class):
Setting
## class(x) to multiple strings ("grouped_df", "tbl_df", ...); result will no
## longer be an S4 object

## Warning in class(x) <- c(setdiff(subclass, tibble_class), tibble_class):
Setting
## class(x) to multiple strings ("tbl_df", "tbl", ...); result will no longer
be an
## S4 object

## Warning in class(x) <- c(setdiff(subclass, tibble_class), tibble_class):
Setting
## class(x) to multiple strings ("grouped_df", "tbl_df", ...); result will no
## longer be an S4 object
```

```
## Warning in class(x) <- c(setdiff(subclass, tibble_class), tibble_class):
Setting
## class(x) to multiple strings ("tbl_df", "tbl", ...); result will no longer
be an
## S4 object

## Warning in class(x) <- c(setdiff(subclass, tibble_class), tibble_class):
Setting
## class(x) to multiple strings ("grouped_df", "tbl_df", ...); result will no
## longer be an S4 object

## Warning in class(x) <- c(setdiff(subclass, tibble_class), tibble_class):
Setting
## class(x) to multiple strings ("tbl_df", "tbl", ...); result will no longer
be an
## S4 object

## Warning in class(x) <- c(setdiff(subclass, tibble_class), tibble_class):
Setting
## class(x) to multiple strings ("grouped_df", "tbl_df", ...); result will no
## longer be an S4 object

## Warning in class(x) <- c(setdiff(subclass, tibble_class), tibble_class):
Setting
## class(x) to multiple strings ("tbl_df", "tbl", ...); result will no longer
be an
## S4 object

## Warning in class(x) <- c(setdiff(subclass, tibble_class), tibble_class):
Setting
## class(x) to multiple strings ("grouped_df", "tbl_df", ...); result will no
## longer be an S4 object

## Warning in class(x) <- c(setdiff(subclass, tibble_class), tibble_class):
Setting
## class(x) to multiple strings ("tbl_df", "tbl", ...); result will no longer
be an
## S4 object

## Warning in class(x) <- c(setdiff(subclass, tibble_class), tibble_class):
Setting
## class(x) to multiple strings ("grouped_df", "tbl_df", ...); result will no
## longer be an S4 object

## Warning in class(x) <- c(setdiff(subclass, tibble_class), tibble_class):
Setting
## class(x) to multiple strings ("tbl_df", "tbl", ...); result will no longer
be an
## S4 object

## Warning in class(x) <- c(setdiff(subclass, tibble_class), tibble_class):
Setting
```

```
## class(x) to multiple strings ("grouped_df", "tbl_df", ...); result will no
## longer be an S4 object

## Warning in class(x) <- c(setdiff(subclass, tibble_class), tibble_class):
Setting
## class(x) to multiple strings ("tbl_df", "tbl", ...); result will no longer
be an
## S4 object

## Warning in class(x) <- c(setdiff(subclass, tibble_class), tibble_class):
Setting
## class(x) to multiple strings ("grouped_df", "tbl_df", ...); result will no
## longer be an S4 object

## Warning in class(x) <- c(setdiff(subclass, tibble_class), tibble_class):
Setting
## class(x) to multiple strings ("tbl_df", "tbl", ...); result will no longer
be an
## S4 object

## Warning in class(x) <- c(setdiff(subclass, tibble_class), tibble_class):
Setting
## class(x) to multiple strings ("grouped_df", "tbl_df", ...); result will no
## longer be an S4 object

## Warning in class(x) <- c(setdiff(subclass, tibble_class), tibble_class):
Setting
## class(x) to multiple strings ("tbl_df", "tbl", ...); result will no longer
be an
## S4 object

## Warning in class(x) <- c(setdiff(subclass, tibble_class), tibble_class):
Setting
## class(x) to multiple strings ("grouped_df", "tbl_df", ...); result will no
## longer be an S4 object

## Warning in class(x) <- c(setdiff(subclass, tibble_class), tibble_class):
Setting
## class(x) to multiple strings ("tbl_df", "tbl", ...); result will no longer
be an
## S4 object

## Warning in class(x) <- c(setdiff(subclass, tibble_class), tibble_class):
Setting
## class(x) to multiple strings ("grouped_df", "tbl_df", ...); result will no
## longer be an S4 object

## Warning in class(x) <- c(setdiff(subclass, tibble_class), tibble_class):
Setting
## class(x) to multiple strings ("tbl_df", "tbl", ...); result will no longer
be an
## S4 object
```

```
## Warning in class(x) <- c(setdiff(subclass, tibble_class), tibble_class):
Setting
## class(x) to multiple strings ("grouped_df", "tbl_df", ...); result will no
## longer be an S4 object

## Warning in class(x) <- c(setdiff(subclass, tibble_class), tibble_class):
Setting
## class(x) to multiple strings ("tbl_df", "tbl", ...); result will no longer
be an
## S4 object

## Warning in class(x) <- c(setdiff(subclass, tibble_class), tibble_class):
Setting
## class(x) to multiple strings ("grouped_df", "tbl_df", ...); result will no
## longer be an S4 object

## Warning in class(x) <- c(setdiff(subclass, tibble_class), tibble_class):
Setting
## class(x) to multiple strings ("tbl_df", "tbl", ...); result will no longer
be an
## S4 object

## Warning in class(x) <- c(setdiff(subclass, tibble_class), tibble_class):
Setting
## class(x) to multiple strings ("grouped_df", "tbl_df", ...); result will no
## longer be an S4 object
```

```
#str(core.out.all.out)

core.out.df.sil <- bind_rows(core.out.all.out, .id = "column_label")

#summary(core.out.df.sil) #now need to add actual Mouse.IDs and ASV names
instead of their position in the lists
core.out.df.sil$ASV_name <- paste0("ASV", core.out.df.sil$ASV_name)

ASV_ave_prev_rand.sil <-  ddply(core.out.df.sil, c("ASV_name"), summarise,
                                N_samp    = length(Prevalence),
                                mean_prev = mean(Prevalence) ,
                                sd_prev   = sd(Prevalence),
                                se_prev   = sd_prev / sqrt(N_samp))
head(ASV_ave_prev_rand.sil)
```

```
##   ASV_name N_samp  mean_prev    sd_prev     se_prev
## 1     ASV1    100 1.00000000 0.00000000 0.000000000
## 2    ASV10    100 0.62670455 0.02899983 0.002899983
## 3   ASV100    100 0.71125000 0.02857938 0.002857938
## 4  ASV1000    100 0.03329545 0.01871457 0.001871457
## 5  ASV1001    100 0.09079545 0.02258305 0.002258305
## 6  ASV1002    100 0.06511364 0.01531038 0.001531038
```

##Plot persistence vs. prevalence in Wytham and Silwood

```
ASV_ave_pers_prev_comb.wyt <- merge(ASV_ave_pers.wyt, ASV_ave_prev_rand.wyt,
by="ASV_name")
ASV_ave_pers_prev_comb.wyt$Population <- "Wytham"
ASV_ave_pers_prev_comb.sil <- merge(ASV_ave_pers.sil, ASV_ave_prev_rand.sil,
by="ASV_name")
ASV_ave_pers_prev_comb.sil$Population <- "Silwood"

#add correct ASV names
### Wytham
ASV_names <- taxa_names(ps.wyt.ra)
ASV_names <- as.data.frame(ASV_names)
#head(ASV_names)
#tail(ASV_names)
ASV_names <- cbind(rownames(ASV_names), ASV_names)
colnames(ASV_names) <- c("ASV_name", "ASV_name_true")
ASV_names$ASV_name <- paste0("ASV", ASV_names$ASV_name)
ASV_ave_pers_prev_comb.wyt$ASV_name_true <-
ASV_names$ASV_name_true[match(ASV_ave_pers_prev_comb.wyt$ASV_name,
ASV_names$ASV_name)]
#head(ASV_ave_pers_prev_comb.wyt)
### Silwood
ASV_names <- taxa_names(ps.sil.ra)
ASV_names <- as.data.frame(ASV_names)
#head(ASV_names)
#tail(ASV_names)
ASV_names <- cbind(rownames(ASV_names), ASV_names)
colnames(ASV_names) <- c("ASV_name", "ASV_name_true")
ASV_names$ASV_name <- paste0("ASV", ASV_names$ASV_name)
ASV_ave_pers_prev_comb.sil$ASV_name_true <-
ASV_names$ASV_name_true[match(ASV_ave_pers_prev_comb.sil$ASV_name,
ASV_names$ASV_name)]
#head(ASV_ave_pers_prev_comb.sil)

### now add taxonomy of ASVs
tax_tab <- cbind(rownames(tax_table(ps.wyt.ra)),
tax_table(ps.wyt.ra)[,c(3,4,5)])
colnames(tax_tab) <- c("ASV_name_true", "Class", "Order", "Family")
tax_tab <- as.data.frame(tax_tab)
ASV_core_tax.wyt <- merge(ASV_ave_pers_prev_comb.wyt, tax_tab,
by="ASV_name_true", all.x = T)
tax_tab <- cbind(rownames(tax_table(ps.sil.ra)),
tax_table(ps.sil.ra)[,c(3,4,5)])
colnames(tax_tab) <- c("ASV_name_true", "Class", "Order", "Family")
tax_tab <- as.data.frame(tax_tab)
ASV_core_tax.sil <- merge(ASV_ave_pers_prev_comb.sil, tax_tab,
by="ASV_name_true", all.x = T)

#combine wytham and silwood data to make one plot, facet by population
ASV_core_tax_comb <- rbind(ASV_core_tax.wyt, ASV_core_tax.sil)
ASV_core_tax_comb$Population <- as.factor(ASV_core_tax_comb$Population)
```

```
all_tax_list <- as.list(unique(as.character(ASV_core_tax_comb$Order)))
keep_ord_list <- c("Clostridiales", "Bacteroidales", "Lactobacillales",
"Coriobacteriales",  "Desulfovibrionales",
                  "Enterobacteriales",  "Rickettsiales")
combine_ord_list <- setdiff(all_tax_list, keep_ord_list)
ASV_core_tax_comb$Order <- ASV_core_tax_comb$Order %>% fct_collapse(Other =
combine_ord_list)

ASV_core_tax_comb$Order <- as.character(ASV_core_tax_comb$Order)
ASV_core_tax_comb$Order[is.na(ASV_core_tax_comb$Order)] <- "Other"
ASV_core_tax_comb$Order <- as.factor(ASV_core_tax_comb$Order)
level_order <- c( "Bacteroidales", "Bifidobacteriales", "Clostridiales",
"Coriobacteriales",
                 "Desulfovibrionales", "Enterobacteriales",
"Lactobacillales",
                 "Rickettsiales", "Other")

ASV_core_tax_comb$Order <- factor(ASV_core_tax_comb$Order, ordered = F,
levels = level_order)
ASV_core_tax_comb$Population <- factor(ASV_core_tax_comb$Population, ordered
= F, levels = c("Wytham", "Silwood"))

ggplot(ASV_core_tax_comb, aes(x=mean_prev, y=mean_pers, group=Order)) +
  facet_wrap(~Population) +
  geom_point(aes(colour=Order)) +
  geom_abline(slope = 1, intercept = 0, linetype= "dashed") +
  theme_bw() +
  theme(axis.text = element_text(size=12), axis.title =
element_text(size=14), legend.text = element_text(size=12),
        legend.title = element_text(size=14), strip.text =
element_text(size=14)) +
  xlab("Mean prevalence across individuals") +
  ylab("Mean persistence within individuals") +
  scale_color_brewer(palette = "Paired")
```

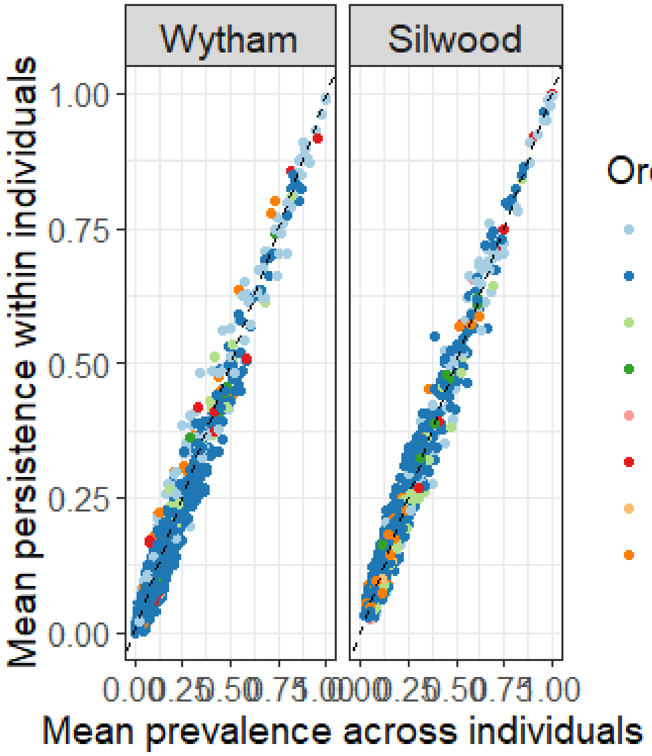

##Taxonomy along the core persistence/prevalence axis

```
#ASV_core_tax.wyt
#subset top right quadrant to look at taxonomic distribution compared to
other 3 quadrants/ all
top_right <- subset(ASV_core_tax.wyt, mean_pers>=0.5 & mean_prev>=0.5) #77
obs
bottom_3 <- subset(ASV_core_tax.wyt, mean_pers<0.5 & mean_prev<0.5 |
mean_pers>0.5 & mean_prev<0.5 | mean_pers<0.5 & mean_prev>0.5)
#summarise taxonomy for each subset and all to then combine for graph
top_right_order_sum <- as.data.frame(summary(as.factor(top_right$Order)))
top_right_order_sum <- cbind(rownames(top_right_order_sum),
top_right_order_sum)
colnames(top_right_order_sum) <- c("Order", "total_ASVs")
top_right_order_sum$Group <- "Top_right"
bottom_3_order_sum <- as.data.frame(summary(as.factor(bottom_3$Order)))
bottom_3_order_sum <- cbind(rownames(bottom_3_order_sum), bottom_3_order_sum)
colnames(bottom_3_order_sum) <- c("Order", "total_ASVs")
bottom_3_order_sum$Group <- "Bottom_3"
all_order_sum <- as.data.frame(summary(as.factor(ASV_core_tax.wyt$Order)))
all_order_sum <- cbind(rownames(all_order_sum), all_order_sum)
colnames(all_order_sum) <- c("Order", "total_ASVs")
all_order_sum$Group <- "All"
#combine
core_tax_sum <- rbind(top_right_order_sum, bottom_3_order_sum)
core_tax_sum2 <- rbind(core_tax_sum, all_order_sum)
core_tax_sum2 <- arrange(core_tax_sum2, desc(total_ASVs))
```

```
#group the rest of the families into 'other' category for plotting
core_tax_sum2$Order <- core_tax_sum2$Order %>% fct_collapse(Other =
c(combine_ord_list))

## Warning: Unknown levels in `f`: Elusimicrobiales, Frankiales

ASV_core_tax_comb$Order <- as.character(ASV_core_tax_comb$Order)
ASV_core_tax_comb$Order[is.na(ASV_core_tax_comb$Order)] <- "Other"
ASV_core_tax_comb$Order <- as.factor(ASV_core_tax_comb$Order)
level_order <- c( "Bacteroidales", "Bifidobacteriales", "Clostridiales",
"Coriobacteriales",
                  "Desulfovibrionales", "Enterobacteriales",
"Lactobacillales",
                  "Rickettsiales", "Other")
ASV_core_tax_comb$Order <- factor(ASV_core_tax_comb$Order, ordered = F,
levels = level_order)
SV_order_sum <-  ddply(core_tax_sum2, c( "Order", "Group"), summarise,
                       N    = sum(total_ASVs))
SV_order_sum$Group <- as.factor(SV_order_sum$Group)
SV_order_sum$Order <- factor(SV_order_sum$Order, ordered = F, levels =
level_order)

SV_order_sum_wyt <- SV_order_sum
SV_order_sum_wyt$Pop <- "Wytham"

#repeat for Silwood
#subset top right quadrant to look at taxonomic distribution compared to
other 3 quadrants/ all
top_right <- subset(ASV_core_tax.sil, mean_pers>=0.5 & mean_prev>=0.5) #77
obs
bottom_3 <- subset(ASV_core_tax.sil, mean_pers<0.5 & mean_prev<0.5 |
mean_pers>0.5 & mean_prev<0.5 | mean_pers<0.5 & mean_prev>0.5)
#summarise taxonomy for each subset and all to then combine for graph
top_right_order_sum <- as.data.frame(summary(as.factor(top_right$Order)))
top_right_order_sum <- cbind(rownames(top_right_order_sum),
top_right_order_sum)
colnames(top_right_order_sum) <- c("Order", "total_ASVs")
top_right_order_sum$Group <- "Top_right"
bottom_3_order_sum <- as.data.frame(summary(as.factor(bottom_3$Order)))
bottom_3_order_sum <- cbind(rownames(bottom_3_order_sum), bottom_3_order_sum)
colnames(bottom_3_order_sum) <- c("Order", "total_ASVs")
bottom_3_order_sum$Group <- "Bottom_3"
all_order_sum <- as.data.frame(summary(as.factor(ASV_core_tax.wyt$Order)))
all_order_sum <- cbind(rownames(all_order_sum), all_order_sum)
colnames(all_order_sum) <- c("Order", "total_ASVs")
all_order_sum$Group <- "All"
#combine
core_tax_sum <- rbind(top_right_order_sum, bottom_3_order_sum)
core_tax_sum2 <- rbind(core_tax_sum, all_order_sum)
```

```
core_tax_sum2 <- arrange(core_tax_sum2, desc(total_ASVs))

#group the rest of the families into 'other' category for plotting
core_tax_sum2$Order <- core_tax_sum2$Order %>% fct_collapse(Other =
c(combine_ord_list))
ASV_core_tax_comb$Order <- as.character(ASV_core_tax_comb$Order)
ASV_core_tax_comb$Order[is.na(ASV_core_tax_comb$Order)] <- "Other"
level_order <- c( "Bacteroidales", "Bifidobacteriales", "Clostridiales",
"Coriobacteriales",
                  "Desulfovibrionales", "Enterobacteriales",
"Lactobacillales",
                  "Rickettsiales", "Other")
ASV_core_tax_comb$Order <- factor(ASV_core_tax_comb$Order, ordered = F,
levels = level_order)

SV_order_sum <-  ddply(core_tax_sum2, c( "Order", "Group"), summarise,
                       N    = sum(total_ASVs))
SV_order_sum$Group <- as.factor(SV_order_sum$Group)
SV_order_sum$Order <- factor(SV_order_sum$Order, ordered = F, levels =
level_order)

SV_order_sum_sil <- SV_order_sum
SV_order_sum_sil$Pop <- "Silwood"

#Combine Wytham and Silwood taxonomy summaries for plotting
SV_order_sum_comb <- rbind(SV_order_sum_wyt, SV_order_sum_sil)
SV_order_sum_comb$Pop <- factor(SV_order_sum_comb$Pop, ordered = F, levels =
c("Wytham", "Silwood"))

ggplot(SV_order_sum_comb, aes( factor(x=Group), y=N))  +
  geom_bar(aes(fill=Order), position='fill', stat="identity") +
  facet_wrap(~Pop)+
  #geom_text(data=temp, size=6,aes(x=x, y=y ,label=as.factor(size))) +
  labs(y= "Proportion of ASVs", x = "Position along prevalence/persistence
axis") +
  theme_bw() +
  theme(axis.text=element_text(size=12), axis.title=element_text(size=14),
        title = element_text(size=14), legend.text = element_text(size=12),
        strip.text = element_text(size=14)) +
  scale_x_discrete(labels=c( "All" = "All", "Bottom_3"= "Under 50%",
"Top_right"="Over 50%")) +
  scale_fill_manual(values = brewer.pal(9,"Paired"))
```

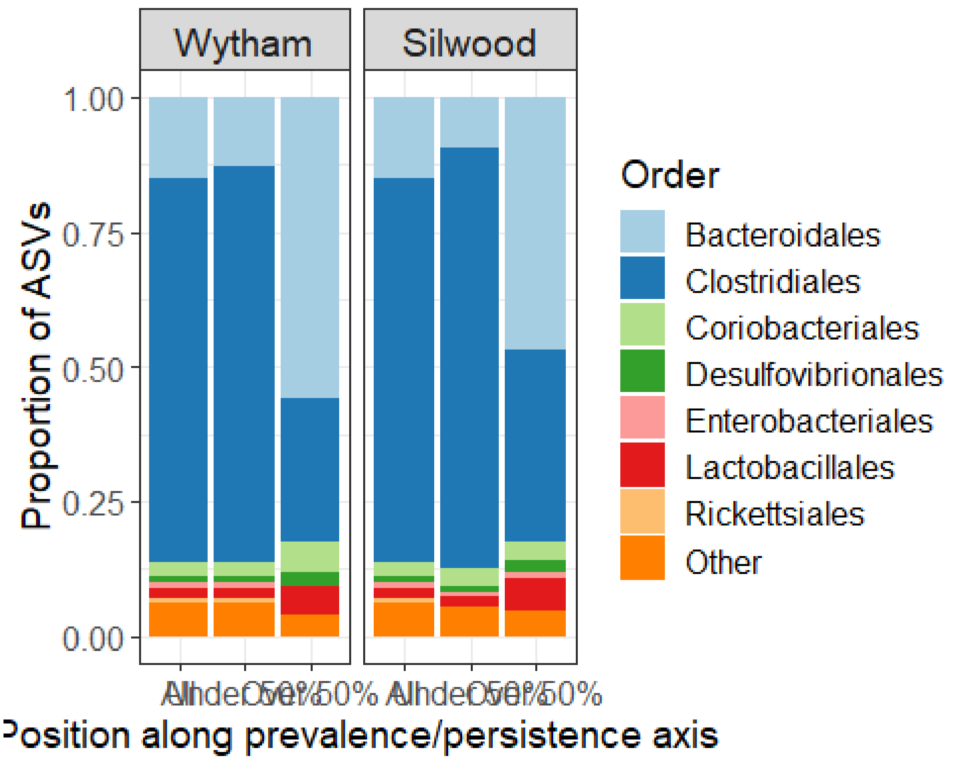

Section 5: Predictors of gut microbiota composition

##Wytham

PERMANOVA

```
library(phyloseq)
library(ggplot2)
library(plyr); library(dplyr)
library(reshape2)
library(forcats)
library(vegan)

#Clean data
M <- as(sample_data(ps.wyt.ra), 'data.frame')
#colnames(M)
#summary(M$XPIT_tag)
M$XPIT_tag <- as.factor(M$XPIT_tag)
levels(M$XPIT_tag)[levels(M$XPIT_tag)==''] <- NA
#length(levels(M$XPIT_tag))
M <- as.data.frame(M)

summary(M)

##  Sequence_sample_code       X          Collection_date        Grid
##  Length:421             Min.   :  1.0   Length:421          Length:421
##  Class :character       1st Qu.:135.0   Class :character    Class :character
```

```
##  Mode  :character     Median :279.0   Mode  :character   Mode  :character
##                       Mean   :267.6
##                       3rd Qu.:395.0
##                       Max.   :525.0
##
##        XPIT_tag    Animal_ID          Trap_num       Grid_sq
##  X041894910F: 11   Length:421         Min.   :   1.0   Length:421
##  X0418EB80D2:  8   Class :character   1st Qu.: 100.0   Class :character
##  X0418EBC464:  8   Mode  :character   Median : 401.0   Mode  :character
##  X041917F78C:  8                      Mean   : 388.7
##  X041917F99A:  8                      3rd Qu.: 617.0
##  (Other)    :364                      Max.   :1047.0
##  NA's       : 14
##   Species.x             Year         Month
Zymo_FS_DNA_extraction_YN
##  Length:421         Min.   :2015   Length:421         Length:421
##  Class :character   1st Qu.:2016   Class :character   Class :character
##  Mode  :character   Median :2017   Mode  :character   Mode  :character
##                     Mean   :2017
##                     3rd Qu.:2017
##                     Max.   :2018
##
##  Extraction_round   Extraction_batch.x Extraction_sample_code
##  Length:421         Length:421         Length:421
##  Class :character   Class :character   Class :character
##  Mode  :character   Mode  :character   Mode  :character
##
##
##
##
##  Poo_mass_mg_used_Zymo_extraction Sequenced_YN       Sequencing_round.x
##  Min.   :20.00                    Length:421         Length:421
##  1st Qu.:32.00                    Class :character   Class :character
##  Median :40.00                    Mode  :character   Mode  :character
##  Mean   :41.32
##  3rd Qu.:48.00
##  Max.   :80.00
##  NA's   :91
##   Miseq_run.x    SCFA_sample_YN     Habitat_type           N_R
##  Min.   :1.000   Length:421         Length:421         Length:421
##  1st Qu.:1.000   Class :character   Class :character   Class :character
##  Median :1.000   Mode  :character   Mode  :character   Mode  :character
##  Mean   :1.437
##  3rd Qu.:2.000
##  Max.   :2.000
##
##      Sex              Reprod             Age              PIT_tag
##  Length:421         Length:421         Length:421         Length:421
##  Class :character   Class :character   Class :character   Class :character
##  Mode  :character   Mode  :character   Mode  :character   Mode  :character
```

```
##
##
##
##
##   Body_mass_g        AGD_1          AGD_2              AGD_3
##  Min.   : 3.10   Min.   : 0.48   Length:421         Min.   :  0.500
##  1st Qu.:17.00   1st Qu.: 5.00   Class :character   1st Qu.:  4.500
##  Median :19.30   Median : 9.50   Mode  :character   Median :  7.950
##  Mean   :19.86   Mean   :10.05                      Mean   :  9.909
##  3rd Qu.:23.30   3rd Qu.:14.60                      3rd Qu.: 12.625
##  Max.   :30.70   Max.   :22.50                      Max.   :211.000
##  NA's   :6       NA's   :45                         NA's   :161
##      Foot_1          Foot_2          Foot_3          Tick_L
##  Min.   : 7.40   Min.   : 7.50   Min.   : 7.10   Min.   : 0.00000
##  1st Qu.:20.00   1st Qu.:19.90   1st Qu.:19.90   1st Qu.: 0.00000
##  Median :20.80   Median :20.50   Median :20.60   Median : 0.00000
##  Mean   :20.52   Mean   :20.35   Mean   :20.35   Mean   : 0.09154
##  3rd Qu.:21.50   3rd Qu.:21.35   3rd Qu.:21.30   3rd Qu.: 0.00000
##  Max.   :24.30   Max.   :27.50   Max.   :27.50   Max.   :17.23000
##  NA's   :35      NA's   :162     NA's   :162     NA's   :200
##     Tick_N             Tick_A             Fleas             Mites
##  Length:421         Min.   :0.00000   Min.   : 0.0000   Min.   :0.00000
##  Class :character   1st Qu.:0.00000   1st Qu.: 0.0000   1st Qu.:0.00000
##  Mode  :character   Median :0.00000   Median : 0.0000   Median :0.00000
##                     Mean   :0.02727   Mean   : 0.1829   Mean   :0.04545
##                     3rd Qu.:0.00000   3rd Qu.: 0.0000   3rd Qu.:0.00000
##                     Max.   :2.00000   Max.   :20.4300   Max.   :3.00000
##                     NA's   :201       NA's   :200       NA's   :201
##       Lept      Body_condition  Peanuts_left        Apple_left
##  Min.   :0.00   Min.   :1.000   Length:421         Length:421
##  1st Qu.:0.00   1st Qu.:1.500   Class :character   Class :character
##  Median :0.00   Median :1.500   Mode  :character   Mode  :character
##  Mean   :0.05   Mean   :1.772
##  3rd Qu.:0.00   3rd Qu.:2.000
##  Max.   :4.00   Max.   :3.000
##  NA's   :201    NA's   :46
##    X.1            X.2            X.3            X.4              X.5
##  Mode:logical   Mode:logical   Mode:logical   Mode:logical   Min.   :1.5
##  NA's:421       NA's:421       NA's:421       NA's:421       1st Qu.:1.5
##                                                              Median :1.5
##                                                              Mean   :1.5
##                                                              3rd Qu.:1.5
##                                                              Max.   :1.5
##                                                              NA's   :420
##    Mouse.ID          Species.y          Diet_Month
Extraction_batch.y
##  Length:421         Length:421         Length:421         Length:421
##  Class :character   Class :character   Class :character   Class :character
##  Mode  :character   Mode  :character   Mode  :character   Mode  :character
##
```

```
##
##
##
##   Sequencing_round.y Miseq_run.y           System      readDepth
##   Length:421         Length:421         Wytham:421   Min.   :  9130
##   Class :character   Class :character                1st Qu.: 34154
##   Mode  :character   Mode  :character                Median : 42355
##                                                      Mean   : 43633
##                                                      3rd Qu.: 51059
##                                                      Max.   :106451
##

M <- subset(M, Sex=="F" | Sex=="M")
# summary(M$Reprod)
M$Reprod <- M$Reprod%>% fct_collapse(active = c("IMP_NIPP","LACT", "NIPP",
"PERF", "Perf, plug", "PREG", "PREG/LACT", "Preg?","PREG?", "Preg? Imp",
"TL", "TL+"))

## Warning: Unknown levels in `f`: PREG?

M$Reprod <- M$Reprod%>% fct_collapse(inactive = c("TA", "TA?","TS", "IMP",
"IMP?"))

## Warning: Unknown levels in `f`: IMP?

levels(M$Reprod)[levels(M$Reprod)==''] <- NA
M <- subset(M, Reprod=="active" | Reprod=="inactive") #inactive=257
active=109
# summary(M$Reprod)
# summary(M$Body_mass_g)
# summary(M$Body_condition)
M$Body_condition <- as.factor(M$Body_condition)
#summary(M$Year)
M$Year <- as.factor(M$Year)
#summary(M$Age)
M <- subset(M, Age=="A" | Age=="J" | Age=="SA") #a=317, j=20, sa=46
#summary(M$Extraction_batch)
#summary(M$Miseq_run)
M$Miseq_run <- as.factor(M$Miseq_run.x)
#summary(M$Extraction_round)
#summary(M$Sequencing_round.x)
M$Seq_run_comb <- paste(M$Sequencing_round.x, M$Miseq_run, sep="_")
#summary(M$Seq_run_comb)
M$Seq_run_comb <- as.factor(M$Seq_run_comb) #WE_1=101, WE_2=114, WD_1=114,
WD_2=54
#dim(M)

#remove missing data
M2 <- M %>%
  subset(
    !is.na(XPIT_tag) &
```

```
      !is.na(readDepth) &
      !is.na(Seq_run_comb) &
      !is.na(Month) &
      !is.na(Year) &
      !is.na(Sex) &
      !is.na(Reprod) &
      !is.na(Body_mass_g) &
      !is.na(Age) &
      !is.na(Body_condition)
  )

#choose 1 sample per mosue
M3 <- M2 %>% group_by(XPIT_tag) %>% sample_n(1)
#length(levels(M3$XPIT_tag))
#levels(M3$XPIT_tag)
M3 <- subset(M3, !is.na(XPIT_tag)) #141obs

M3 <- as.data.frame(M3)
rownames(M3) <- M3$Sequence_sample_code
wyt_physeq_ra_tidy <- phyloseq(sample_data(M3),
otu_table(otu_table(ps.wyt.ra)), tax_table(tax_table(ps.wyt.ra)))
wyt_physeq_ra_tidy <- prune_taxa(taxa_sums(wyt_physeq_ra_tidy) > 0,
wyt_physeq_ra_tidy)
#wyt_physeq_ra_tidy

#### PERMANOVA
D<- phyloseq::distance(wyt_physeq_ra_tidy, method = "bray", type = "samples")
M4<-sample_data(wyt_physeq_ra_tidy)
#summary(M4)

#1. including sex*reprod interaction
adonis2(D ~ M4$readDepth  +  M4$Seq_run_comb  +
          M4$Month + M4$Year +
          M4$Sex + M4$Reprod  + M4$Sex* M4$Reprod + M4$Age + M4$Body_mass_g +
M4$Body_condition, na.rm=TRUE,
        by="margin")

## Permutation test for adonis under reduced model
## Marginal effects of terms
## Permutation: free
## Number of permutations: 999
##
## adonis2(formula = D ~ M4$readDepth + M4$Seq_run_comb + M4$Month + M4$Year
+ M4$Sex + M4$Reprod + M4$Sex * M4$Reprod + M4$Age + M4$Body_mass_g +
M4$Body_condition, by = "margin", na.rm = TRUE)
##                      Df SumOfSqs      R2      F Pr(>F)
## M4$readDepth          1    0.485 0.01103 1.7117  0.017 *
## M4$Seq_run_comb       3    1.297 0.02951 1.5266  0.005 **
## M4$Month             11    5.253 0.11948 1.6861  0.001 ***
## M4$Year               3    1.955 0.04447 2.3011  0.001 ***
```

```
## M4$Age              2    0.638 0.01451 1.1262  0.235
## M4$Body_mass_g      1    0.364 0.00827 1.2841  0.146
## M4$Body_condition   4    1.231 0.02800 1.0866  0.226
## M4$Sex:M4$Reprod    1    0.247 0.00563 0.8732  0.652
## Residual          106   30.025 0.68289
## Total             134   43.967 1.00000
## ---
## Signif. codes:  0 '***' 0.001 '**' 0.01 '*' 0.05 '.' 0.1 ' ' 1

#2. no sex*reprod interaction
adonis2(D ~ M4$readDepth  +  M4$Seq_run_comb  +
          M4$Month + M4$Year +
          M4$Sex + M4$Reprod + M4$Age + M4$Body_mass_g + M4$Body_condition,
na.rm=TRUE,
        by="margin")

## Permutation test for adonis under reduced model
## Marginal effects of terms
## Permutation: free
## Number of permutations: 999
##
## adonis2(formula = D ~ M4$readDepth + M4$Seq_run_comb + M4$Month + M4$Year
+ M4$Sex + M4$Reprod + M4$Age + M4$Body_mass_g + M4$Body_condition, by =
"margin", na.rm = TRUE)
##                    Df SumOfSqs      R2      F Pr(>F)
## M4$readDepth        1    0.512 0.01164 1.8096  0.012 *
## M4$Seq_run_comb     3    1.300 0.02958 1.5322  0.004 **
## M4$Month           11    5.316 0.12090 1.7081  0.001 ***
## M4$Year             3    1.956 0.04448 2.3041  0.001 ***
## M4$Sex              1    0.308 0.00701 1.0893  0.289
## M4$Reprod           1    0.312 0.00710 1.1039  0.287
## M4$Age              2    0.638 0.01450 1.1267  0.197
## M4$Body_mass_g      1    0.363 0.00825 1.2818  0.125
## M4$Body_condition   4    1.222 0.02780 1.0799  0.272
## Residual          107   30.272 0.68852
## Total             134   43.967 1.00000
## ---
## Signif. codes:  0 '***' 0.001 '**' 0.01 '*' 0.05 '.' 0.1 ' ' 1

#test for homogeniety of variance for processing batch, month, year and body
mass using betadisper
beta <- betadisper(D, M4$Month)
permutest(beta)

##
## Permutation test for homogeneity of multivariate dispersions
## Permutation: free
## Number of permutations: 999
##
## Response: Distances
##             Df  Sum Sq   Mean Sq      F N.Perm Pr(>F)
```

```
## Groups     11 0.08493 0.0077206 1.4603    999  0.138
## Residuals 123 0.65029 0.0052869

beta <- betadisper(D, M4$Year)
permutest(beta)

##
## Permutation test for homogeneity of multivariate dispersions
## Permutation: free
## Number of permutations: 999
##
## Response: Distances
##            Df  Sum Sq   Mean Sq     F N.Perm Pr(>F)
## Groups      3 0.06360 0.0211988 5.587    999  0.002 **
## Residuals 131 0.49705 0.0037943
## ---
## Signif. codes:  0 '***' 0.001 '**' 0.01 '*' 0.05 '.' 0.1 ' ' 1

beta <- betadisper(D, M4$Seq_run_comb)
permutest(beta)

##
## Permutation test for homogeneity of multivariate dispersions
## Permutation: free
## Number of permutations: 999
##
## Response: Distances
##            Df  Sum Sq   Mean Sq      F N.Perm Pr(>F)
## Groups      3 0.03264 0.0108784 3.2298    999  0.032 *
## Residuals 131 0.44122 0.0033681
## ---
## Signif. codes:  0 '***' 0.001 '**' 0.01 '*' 0.05 '.' 0.1 ' ' 1
```

Wytham pRDA

```
##Hellinger transformed phyloseq object
ps.wyt.hell <- transform_sample_counts(ps.wyt.ra, function(x) sqrt(x))
#(already relative abundance transformed so just need to square root it)
ps.wyt.hell <- phyloseq(otu_table(otu_table(ps.wyt.hell)),
tax_table(tax_table(ps.wyt.hell)), sample_data(M2))#remake with clean
meatadata
#ps.wyt.hell

#Partial RDA
pRDA <- ordinate(ps.wyt.hell, method="RDA", formula = ~ Month + Year +
readDepth + Seq_run_comb +
                                    Age + Sex + Reprod + Body_mass_g +
Body_condition +
                                   Condition(XPIT_tag),
                                   na.action=na.fail)
pRDA #Examine output
```

```
## Call: rda(formula = OTU ~ Month + Year + readDepth + Seq_run_comb + Age
## + Sex + Reprod + Body_mass_g + Body_condition + Condition(XPIT_tag),
## data = data)
##
##               Inertia Proportion Rank
## Total         0.64300    1.00000
## Conditional   0.35393    0.55043  134
## Constrained   0.06207    0.09653   27
## Unconstrained 0.22700    0.35304  156
## Inertia is variance
##
## Eigenvalues for constrained axes:
##     RDA1     RDA2     RDA3     RDA4     RDA5     RDA6     RDA7     RDA8
## 0.012743 0.009131 0.004906 0.003834 0.003062 0.002611 0.002281 0.002148
##     RDA9    RDA10    RDA11    RDA12    RDA13    RDA14    RDA15    RDA16
## 0.001850 0.001716 0.001663 0.001523 0.001381 0.001336 0.001286 0.001214
##    RDA17    RDA18    RDA19    RDA20    RDA21    RDA22    RDA23    RDA24
## 0.001176 0.001108 0.001081 0.000984 0.000906 0.000834 0.000808 0.000794
##    RDA25    RDA26    RDA27
## 0.000709 0.000667 0.000316
##
## Eigenvalues for unconstrained axes:
##      PC1      PC2      PC3      PC4      PC5      PC6      PC7      PC8
## 0.020368 0.017364 0.011100 0.008897 0.007273 0.006168 0.005227 0.004660
## (Showing 8 of 156 unconstrained eigenvalues)
```

```
RsquareAdj(pRDA)$adj.r.squared
```

```
## [1] 0.06136834
```

```
anova(pRDA)
```

```
## Permutation test for rda under reduced model
## Permutation: free
## Number of permutations: 999
##
## Model: rda(formula = OTU ~ Month + Year + readDepth + Seq_run_comb + Age +
Sex + Reprod + Body_mass_g + Body_condition + Condition(XPIT_tag), data =
data)
##           Df Variance      F Pr(>F)
## Model     27 0.062069 1.5798  0.001 ***
## Residual 156 0.227004
## ---
## Signif. codes:  0 '***' 0.001 '**' 0.01 '*' 0.05 '.' 0.1 ' ' 1
```

```
anova(pRDA, by="margin", model="reduced")
```

```
## Permutation test for rda under reduced model
## Marginal effects of terms
## Permutation: free
## Number of permutations: 999
```

```
##
## Model: rda(formula = OTU ~ Month + Year + readDepth + Seq_run_comb + Age +
Sex + Reprod + Body_mass_g + Body_condition + Condition(XPIT_tag), data =
data)
##                 Df Variance      F Pr(>F)
## Month           11 0.024111 1.5063  0.001 ***
## Year             3 0.008273 1.8950  0.001 ***
## readDepth        1 0.002084 1.4319  0.058 .
## Seq_run_comb     3 0.005803 1.3292  0.019 *
## Age              2 0.003602 1.2375  0.089 .
## Sex              1 0.000635 0.4361  0.807
## Reprod           1 0.001179 0.8103  0.828
## Body_mass_g      1 0.001680 1.1549  0.190
## Body_condition   4 0.005844 1.0040  0.434
## Residual       156 0.227004
## ---
## Signif. codes:  0 '***' 0.001 '**' 0.01 '*' 0.05 '.' 0.1 ' ' 1
```

##Silwood

PERMANOVA

```
M <- as(sample_data(ps.sil.ra), 'data.frame')
M <- as.data.frame(M)
# summary(M)
# summary(M$ID)
# summary(M$Reprod)
M$Reprod <- M$Reprod%>% fct_collapse(active = c("NIPPLES", "PERF", "PREG",
"TL", "TL+"))
M$Reprod <- M$Reprod%>% fct_collapse(inactive = c("IMP", "TA", "TS"))
M <- subset(M, Reprod=="active" | Reprod=="inactive")
# summary(M$Body_mass_grams)
# summary(M$Body_condition)
M$Body_condition <- as.factor(M$Body_condition)
#summary(M$year)
M$year <- as.factor(M$year)
#summary(M$Age)
#summary(M$pcr_plate)
M$pcr_plate <- as.factor(M$pcr_plate)

M2 <- M %>%
  subset(
    !is.na(ID) &
      !is.na(readDepth) &
      !is.na(month) &
      !is.na(year) &
      !is.na(Sex) &
      !is.na(Reprod) &
      !is.na(Body_mass_grams) &
      !is.na(Age) &
```

```
      !is.na(Body_condition)
  )

M3 <- M2 %>% group_by(ID) %>% sample_n(1) #choose one random sample per
individual
M3 <- as.data.frame(M3)
rownames(M3) <- M3$Sequence_sample_code
sil_physeq_ra_tidy <- phyloseq(sample_data(M3),
otu_table(otu_table(ps.sil.ra)), tax_table(tax_table(ps.sil.ra)))
sil_physeq_ra_tidy <- prune_taxa(taxa_sums(sil_physeq_ra_tidy) > 0,
sil_physeq_ra_tidy)
#sil_physeq_ra_tidy

#prep data fro permanova
D<- phyloseq::distance(sil_physeq_ra_tidy, method = "bray", type = "samples")
M<-sample_data(sil_physeq_ra_tidy)
#summary(M)

#1. including sex*reprod interaction
adonis2(D ~ M$readDepth  +  M$pcr_plate  +
          M$month + M$year +
          M$Sex*M$Reprod + M$Age + M$Body_mass_grams + M$Body_condition,
na.rm=TRUE,
        by="margin")

## Permutation test for adonis under reduced model
## Marginal effects of terms
## Permutation: free
## Number of permutations: 999
##
## adonis2(formula = D ~ M$readDepth + M$pcr_plate + M$month + M$year + M$Sex
* M$Reprod + M$Age + M$Body_mass_grams + M$Body_condition, by = "margin",
na.rm = TRUE)
##                   Df SumOfSqs      R2      F Pr(>F)
## M$readDepth        1   0.2544 0.01134 1.1801  0.221
## M$pcr_plate        3   0.6181 0.02756 0.9557  0.535
## M$month           11   3.9995 0.17833 1.6865  0.001 ***
## M$year             1   0.9609 0.04284 4.4572  0.001 ***
## M$Age              2   0.4868 0.02171 1.1290  0.248
## M$Body_mass_grams  1   0.2198 0.00980 1.0196  0.403
## M$Body_condition   4   1.2515 0.05580 1.4513  0.014 *
## M$Sex:M$Reprod     1   0.1733 0.00773 0.8038  0.676
## Residual          60  12.9350 0.57675
## Total             86  22.4274 1.00000
## ---
## Signif. codes:  0 '***' 0.001 '**' 0.01 '*' 0.05 '.' 0.1 ' ' 1

#2. not including sex*reprod interaction
adonis2(D ~ M$readDepth  +
          M$month +
```

```
          M$Sex + M$Reprod  + M$Age + M$Body_mass_grams + M$Body_condition, 
na.rm=TRUE,
        by="margin")

## Permutation test for adonis under reduced model
## Marginal effects of terms
## Permutation: free
## Number of permutations: 999
## 
## adonis2(formula = D ~ M$readDepth + M$month + M$Sex + M$Reprod + M$Age + 
M$Body_mass_grams + M$Body_condition, by = "margin", na.rm = TRUE)
##                    Df SumOfSqs      R2      F Pr(>F)    
## M$readDepth         1   0.2327 0.01037 1.0275  0.378    
## M$month            11   3.8611 0.17216 1.5501  0.001 ***
## M$Sex               1   0.2051 0.00914 0.9056  0.521    
## M$Reprod            1   0.2339 0.01043 1.0331  0.370    
## M$Age               2   0.5866 0.02616 1.2952  0.150    
## M$Body_mass_grams   1   0.2530 0.01128 1.1172  0.276    
## M$Body_condition    4   1.4566 0.06495 1.6081  0.003 ** 
## Residual           65  14.7191 0.65630                  
## Total              86  22.4274 1.00000                  
## ---
## Signif. codes:  0 '***' 0.001 '**' 0.01 '*' 0.05 '.' 0.1 ' ' 1

#test for homogeniety of variance for  month, year and reprod using 
betadisper
beta <- betadisper(D, M$month)
permutest(beta)

## 
## Permutation test for homogeneity of multivariate dispersions
## Permutation: free
## Number of permutations: 999
## 
## Response: Distances
##           Df  Sum Sq  Mean Sq      F N.Perm Pr(>F)    
## Groups    11 0.39886 0.036260 4.4792    999  0.001 ***
## Residuals 75 0.60713 0.008095                         
## ---
## Signif. codes:  0 '***' 0.001 '**' 0.01 '*' 0.05 '.' 0.1 ' ' 1

beta <- betadisper(D, M$Reprod)
permutest(beta)

## 
## Permutation test for homogeneity of multivariate dispersions
## Permutation: free
## Number of permutations: 999
## 
## Response: Distances
##           Df  Sum Sq   Mean Sq     F N.Perm Pr(>F)
```

```
## Groups     1 0.00079 0.0007942 0.145    999  0.699
## Residuals 85 0.46572 0.0054790
```

Silwood pRDA

```
#remake phyloseq object
ps.sil.hell <- transform_sample_counts(ps.sil.ra, function(x) sqrt(x))
ps.sil.hell <- phyloseq(otu_table(otu_table(ps.sil.hell)),
tax_table(tax_table(ps.sil.hell)), sample_data(M2))
#ps.sil.hell

##### #Partial RDA
pRDA.sil <- ordinate(ps.sil.hell, method="RDA", formula = ~ month + readDepth
+
                   Age + Sex + Reprod + Body_mass_grams + Body_condition +
                   Condition(ID),
                 na.action=na.fail)
#Examine output
pRDA.sil

## Call: rda(formula = OTU ~ month + readDepth + Age + Sex + Reprod +
## Body_mass_grams + Body_condition + Condition(ID), data = data)
##
##                Inertia Proportion Rank
## Total          0.53220    1.00000
## Conditional    0.26110    0.49060   86
## Constrained    0.04783    0.08988   21
## Unconstrained 0.22327    0.41951  162
## Inertia is variance
## Some constraints were aliased because they were collinear (redundant)
##
## Eigenvalues for constrained axes:
##     RDA1     RDA2     RDA3     RDA4     RDA5     RDA6     RDA7     RDA8
## 0.009623 0.005774 0.004741 0.004088 0.003297 0.002295 0.002033 0.001921
##     RDA9    RDA10    RDA11    RDA12    RDA13    RDA14    RDA15    RDA16
## 0.001528 0.001444 0.001289 0.001223 0.001155 0.001128 0.001080 0.001025
##    RDA17    RDA18    RDA19    RDA20    RDA21
## 0.000974 0.000860 0.000820 0.000782 0.000756
##
## Eigenvalues for unconstrained axes:
##      PC1      PC2      PC3      PC4      PC5      PC6      PC7      PC8
## 0.027932 0.020541 0.009235 0.007452 0.005923 0.004793 0.004290 0.004140
## (Showing 8 of 162 unconstrained eigenvalues)

RsquareAdj(pRDA.sil)$adj.r.squared

## [1] 0.05218246

anova(pRDA.sil)
```

```
## Permutation test for rda under reduced model
## Permutation: free
## Number of permutations: 999
##
## Model: rda(formula = OTU ~ month + readDepth + Age + Sex + Reprod +
Body_mass_grams + Body_condition + Condition(ID), data = data)
##           Df Variance      F Pr(>F)
## Model     21 0.047835 1.6528  0.001 ***
## Residual 162 0.223266
## ---
## Signif. codes:  0 '***' 0.001 '**' 0.01 '*' 0.05 '.' 0.1 ' ' 1
```

```
anova(pRDA.sil, by="margin", model="reduced")
```

```
## Permutation test for rda under reduced model
## Marginal effects of terms
## Permutation: free
## Number of permutations: 999
##
## Model: rda(formula = OTU ~ month + readDepth + Age + Sex + Reprod +
Body_mass_grams + Body_condition + Condition(ID), data = data)
##                  Df Variance      F Pr(>F)
## month            11 0.027736 1.8296  0.001 ***
## readDepth         1 0.001641 1.1910  0.192
## Age               2 0.002835 1.0283  0.420
## Sex               0 0.000000
## Reprod            1 0.001704 1.2367  0.166
## Body_mass_grams   1 0.002541 1.8434  0.013 *
## Body_condition    5 0.008339 1.2102  0.061 .
## Residual        162 0.223266
## ---
## Signif. codes:  0 '***' 0.001 '**' 0.01 '*' 0.05 '.' 0.1 ' ' 1
```

Section 6: Seasonal restructuring

##Wytham

Ordination and PC1 GAMM

```
library(phyloseq)
library(ggplot2)
library(forcats)
library(reshape2)
library(plyr) ; library(dplyr)
library(gridExtra)
library(mgcv)
```

```
## Loading required package: nlme
```

```
##
## Attaching package: 'nlme'
```

```
## The following object is masked from 'package:Biostrings':
##
##     collapse

## The following object is masked from 'package:IRanges':
##
##     collapse

## The following object is masked from 'package:dplyr':
##
##     collapse

## This is mgcv 1.8-33. For overview type 'help("mgcv-package")'.

##
## Attaching package: 'mgcv'

## The following object is masked from 'package:futile.logger':
##
##     scat

library(vegan)

#ordinate - wytham (Bray-Curtis)
sample_ord_bray_wyt <- ordinate(ps.wyt.ra, method= "PCoA", distance= 'bray')
##extract first few axes as variables in metadata
axis_sub_wyt<- sample_ord_bray_wyt$vectors[,1:6]
axis_sub_wyt <- cbind(rownames(axis_sub_wyt), axis_sub_wyt)
colnames(axis_sub_wyt)[1] <- "Sequence_sample_code"
axis_sub_wyt <- as.data.frame(axis_sub_wyt)
#ordinate - wytham (Jaccard)
ps.wyt.ra.bin <- transform_sample_counts(ps.wyt.ra, function(x) 1*(x>0))
sample_ord_jac_wyt <- ordinate(ps.wyt.ra.bin, method= "PCoA", distance=
'jaccard')
##extract first axis as variable in metadata
axis_sub_wyt_jac<- sample_ord_jac_wyt$vectors[,1]
axis_sub_wyt_jac <- as.data.frame(axis_sub_wyt_jac)
axis_sub_wyt_jac <- cbind(rownames(axis_sub_wyt_jac), axis_sub_wyt_jac)
colnames(axis_sub_wyt_jac) <- c("Sequence_sample_code", "JaccardPC1")
axis_sub_wyt_comb <- merge(axis_sub_wyt, axis_sub_wyt_jac,
by="Sequence_sample_code")

physeq_met_wyt <- as(sample_data(ps.wyt.ra), "data.frame")
physeq_merge <- merge(physeq_met_wyt, axis_sub_wyt_comb,
by="Sequence_sample_code")
rownames(physeq_merge) <- physeq_merge$Sequence_sample_code
ps.wyt.ra <- phyloseq(sample_data(physeq_merge),
otu_table(otu_table(ps.wyt.ra)), tax_table(tax_table(ps.wyt.ra)))

sample_data(ps.wyt.ra)$Axis.1 <-
as.numeric(as.character(sample_data(ps.wyt.ra)$Axis.1))
```

```
sample_data(ps.wyt.ra)$Axis.2 <-
as.numeric(as.character(sample_data(ps.wyt.ra)$Axis.2))
sample_data(ps.wyt.ra)$Axis.3 <-
as.numeric(as.character(sample_data(ps.wyt.ra)$Axis.3))
sample_data(ps.wyt.ra)$Axis.4 <-
as.numeric(as.character(sample_data(ps.wyt.ra)$Axis.4))
sample_data(ps.wyt.ra)$Axis.5 <-
as.numeric(as.character(sample_data(ps.wyt.ra)$Axis.5))
sample_data(ps.wyt.ra)$Axis.6 <-
as.numeric(as.character(sample_data(ps.wyt.ra)$Axis.6))
sample_data(ps.wyt.ra)$JaccardPC1 <-
as.numeric(as.character(sample_data(ps.wyt.ra)$JaccardPC1))

#metadata manipulations

#summary(sample_data(ps.wyt.ra))
sample_data(ps.wyt.ra)$Year <- as.factor(sample_data(ps.wyt.ra)$Year)
sample_data(ps.wyt.ra)$M_Y<-
paste(sample_data(ps.wyt.ra)$Month,sample_data(ps.wyt.ra)$Year,sep="/")
#sample_data(ps.wyt.ra)$M_Y
level_order1 <- c("October/2015", "November/2015", "December/2015",
"January/2016",
                  "February/2016","March/2016", "April/2016", "May/2016",
"June/2016",
                  "July/2016", "August/2016", "September/2016",
"October/2016",
                  "November/2016", "December/2016", "January/2017",
"February/2017",
                  "March/2017","April/2017", "May/2017", "June/2017",
"July/2017",
                  "August/2017", "September/2017", "October/2017",
"November/2017",
                  "December/2017", "January/2018", "February/2018",
"March/2018",
                  "April/2018", "May/2018", "June/2018", "July/2018",
"August/2018",
                  "September/2018", "October/2018")
sample_data(ps.wyt.ra)$M_Y <- factor(sample_data(ps.wyt.ra)$M_Y, ordered = T,
levels = level_order1)
#sample_data(ps.wyt.ra)$M_Y
#Group into 15/16 and 16/17 using this new column
Year15_16 <- c("October/2015", "November/2015", "December/2015",
"January/2016",
               "February/2016", "March/2016", "April/2016", "May/2016",
"June/2016",
               "July/2016", "August/2016","September/2016", "October/2016")
Year16_17 <- c( "November/2016", "December/2016", "January/2017",
"February/2017",
                "March/2017", "April/2017", "May/2017", "June/2017",
"July/2017",
```

```
                "August/2017", "September/2017", "October/2017")
Year17_18 <- c( "November/2017", "December/2017", "January/2018",
"February/2018",
                "March/2018", "April/2018", "May/2018", "June/2018",
"July/2018",
                "August/2018", "September/2018", "October/2018")
sample_data(ps.wyt.ra)$Sampling_year<- "NA"
sample_data(ps.wyt.ra)[which(sample_data(ps.wyt.ra)$M_Y%in%Year15_16),]$Sampl
ing_year<-"Year15_16"
sample_data(ps.wyt.ra)[which(sample_data(ps.wyt.ra)$M_Y%in%Year16_17),]$Sampl
ing_year<-"Year16_17"
sample_data(ps.wyt.ra)[which(sample_data(ps.wyt.ra)$M_Y%in%Year17_18),]$Sampl
ing_year<-"Year17_18"
sample_data(ps.wyt.ra)$Sampling_year <-
as.factor(sample_data(ps.wyt.ra)$Sampling_year)
#summary(sample_data(ps.wyt.ra)$Sampling_year)
levels(sample_data(ps.wyt.ra)$Sampling_year)[levels(sample_data(ps.wyt.ra)$Sa
mpling_year)=='NA'] <- NA

physeq_met <- as(sample_data(ps.wyt.ra), 'data.frame')
#colnames(physeq_met)
#summary(physeq_met$Collection_date) #format is dd/mm/yyyy, currently a
factor
physeq_met$Collection_date <- as.Date(physeq_met$Collection_date, format=
"%d/%m/%Y") #now collection date is recognised as in date format
physeq_met$Day_of_year <- format(physeq_met$Collection_date, "%j")#now get
day of the year
#summary(physeq_met$Day_of_year)
physeq_met$Day_of_year <- as.numeric(physeq_met$Day_of_year)
#summary(physeq_met$XPIT_tag)
physeq_met$XPIT_tag <- as.factor(physeq_met$XPIT_tag)
levels(physeq_met$XPIT_tag)[levels(physeq_met$XPIT_tag)==''] <- NA
physeq_met <- subset(physeq_met, Grid=="Main" & !is.na(XPIT_tag))
physeq_met$Year <- as.factor(physeq_met$Year)
physeq_met$Seq_run_comb <- paste(physeq_met$Sequencing_round.x,
physeq_met$Miseq_run.x, sep="_")
#summary(physeq_met$Seq_run_comb)
physeq_met$Seq_run_comb <- as.factor(physeq_met$Seq_run_comb)
#summary(physeq_met$Sex)
physeq_met$Sex <- physeq_met$Sex %>% fct_collapse(F = c("F", "F?"), M=c("M",
"M?"))
levels(physeq_met$Sex)[levels(physeq_met$Sex)==''] <- NA
#summary(physeq_met$Age)
physeq_met$Age <- physeq_met$Age %>% fct_collapse(J = c("J", "J?"),
SA=c("SA", "SA?"))
levels(physeq_met$Age)[levels(physeq_met$Age)==''] <- NA
#summary(physeq_met$Reprod)
physeq_met$Reprod <- physeq_met$Reprod%>% fct_collapse(
  active = c("IMP_NIPP","LACT", "NIPP", "PERF", "Perf, plug", "PREG",
"PREG/LACT",
```

```
              "Preg?","PREG?", "Preg? Imp", "TL", "TL+"))
physeq_met$Reprod <- physeq_met$Reprod%>% fct_collapse(
  inactive = c("TA", "TA?","TS", "IMP", "IMP?"))
levels(physeq_met$Reprod)[levels(physeq_met$Reprod)==''] <- NA
#summary(physeq_met$Body_condition)
physeq_met$Body_condition <- as.factor(physeq_met$Body_condition)
#summary(physeq_met$Body_mass_g)

physeq_met_sub <- subset(physeq_met, !is.na(Age) & !is.na(Sex) &
!is.na(Reprod) &
                           !is.na(Body_mass_g) & !is.na(Body_condition))

### PC1 GAMM
model1 <- gamm(Axis.1 ~ s(Day_of_year, bs = "cc", k=24, by=Sampling_year) +
                  Sampling_year + Sex + Reprod + Age + Body_mass_g +
Body_condition + Seq_run_comb,
               data = physeq_met_sub,
               random=list(XPIT_tag=~1),
               na.action = na.exclude)

#check model assumptions
# sresid <- model1$gam$residuals
# hist(sresid)#normal with arcsine, not clr, logit doesn't work
# fitted.glmm <- model1$gam$fitted              # Extract the fitted
(predicted) values
# plot(sresid ~ fitted.glmm)                    # Check for homoscedasticity
# plot(sresid ~ physeq_met_sub$Day_of_year)  # plot the residuals against the
independent variable(s)
#
# #look at residuals using partial autocorrelation function
# layout(matrix(1:2, ncol = 2))
# acf(resid(model1$lme), lag.max = 36, main = "ACF")
# pacf(resid(model1$lme), lag.max = 36, main = "pACF")
# layout(1)
#lots of residual autocorrelation
#try fitting model with correlated errors using ARMA process
#order 1
# model2 <- gamm(Axis.1 ~ s(Day_of_year, bs = "cc", k=24, by=Sampling_year) +
#                   Sampling_year + Sex + Reprod + Age + Body_mass_g +
Body_condition + Seq_run_comb,
#                data = physeq_met_sub,
#                random=list(XPIT_tag=~1),
#                na.action = na.exclude,
#                correlation = corARMA( p = 1))
#
# #order 2
# model3 <- gamm(Axis.1 ~ s(Day_of_year, bs = "cc", k=24, by=Sampling_year) +
#                   Sampling_year + Sex + Reprod + Age + Body_mass_g +
Body_condition + Seq_run_comb,
#                data = physeq_met_sub,
```

```
#                 random=list(XPIT_tag=~1),
#                 na.action = na.exclude,
#                 correlation = corARMA(p = 2))
# #order 3
# model4 <- gamm(Axis.1 ~ s(Day_of_year, bs = "cc", k=24, by=Sampling_year) +
#                    Sampling_year + Sex + Reprod + Age + Body_mass_g +
Body_condition + Seq_run_comb,
#                 data = physeq_met_sub,
#                 random=list(XPIT_tag=~1),
#                 na.action = na.exclude,
#                 correlation = corARMA( p = 3))
#which model fits the data best - generalized likelihood ratio test
#anova(model1$lme, model2$lme, model3$lme, model4$lme)
#models with correlated errors aren't significantly better than model1

summary(model1$mer)

## Length  Class   Mode
##      0   NULL   NULL

summary(model1$gam)

##
## Family: gaussian
## Link function: identity
##
## Formula:
## Axis.1 ~ s(Day_of_year, bs = "cc", k = 24, by = Sampling_year) +
##     Sampling_year + Sex + Reprod + Age + Body_mass_g + Body_condition +
##     Seq_run_comb
##
## Parametric coefficients:
##                         Estimate Std. Error t value Pr(>|t|)
## (Intercept)             0.004571   0.072433   0.063  0.94972
## Sampling_yearYear16_17  0.143904   0.029412   4.893 1.64e-06 ***
## Sampling_yearYear17_18  0.201779   0.050610   3.987 8.45e-05 ***
## SexM                   -0.017273   0.020490  -0.843  0.39992
## Reprodactive           -0.010720   0.022343  -0.480  0.63173
## AgeJ                   -0.030515   0.039696  -0.769  0.44269
## AgeSA                  -0.055589   0.027618  -2.013  0.04505 *
## Body_mass_g            -0.005931   0.003095  -1.916  0.05631 .
## Body_condition1.5      -0.019808   0.026063  -0.760  0.44786
## Body_condition2        -0.028354   0.027225  -1.041  0.29852
## Body_condition2.5      -0.050138   0.034810  -1.440  0.15083
## Body_condition3        -0.011577   0.042461  -0.273  0.78531
## Seq_run_combExp_Wyt_2   0.076600   0.023459   3.265  0.00122 **
## Seq_run_combWyt_Diss_1  0.034248   0.045512   0.753  0.45235
## Seq_run_combWyt_Diss_2  0.051116   0.044107   1.159  0.24743
## ---
## Signif. codes:  0 '***' 0.001 '**' 0.01 '*' 0.05 '.' 0.1 ' ' 1
```

```
##
## Approximate significance of smooth terms:
##                                        edf Ref.df     F  p-value
## s(Day_of_year):Sampling_yearYear15_16 3.238     12 2.673 1.23e-06 ***
## s(Day_of_year):Sampling_yearYear16_17 7.219     20 3.178  < 2e-16 ***
## s(Day_of_year):Sampling_yearYear17_18 8.411     19 3.609  < 2e-16 ***
## ---
## Signif. codes:  0 '***' 0.001 '**' 0.01 '*' 0.05 '.' 0.1 ' ' 1
##
## R-sq.(adj) =  0.554
##   Scale est. = 0.014432  n = 328

anova(model1$gam)

##
## Family: gaussian
## Link function: identity
##
## Formula:
## Axis.1 ~ s(Day_of_year, bs = "cc", k = 24, by = Sampling_year) +
##     Sampling_year + Sex + Reprod + Age + Body_mass_g + Body_condition +
##     Seq_run_comb
##
## Parametric Terms:
##                df      F  p-value
## Sampling_year   2 13.033 3.77e-06
## Sex             1  0.711   0.3999
## Reprod          1  0.230   0.6317
## Age             2  2.034   0.1327
## Body_mass_g     1  3.672   0.0563
## Body_condition  4  0.620   0.6485
## Seq_run_comb    3  3.680   0.0125
##
## Approximate significance of smooth terms:
##                                         edf Ref.df     F  p-value
## s(Day_of_year):Sampling_yearYear15_16  3.238 12.000 2.673 1.23e-06
## s(Day_of_year):Sampling_yearYear16_17  7.219 20.000 3.178  < 2e-16
## s(Day_of_year):Sampling_yearYear17_18  8.411 19.000 3.609  < 2e-16

#Plot from model
#interested in the seasonal component, so need to extract individual terms
from model
# summary(physeq_met_sub$Body_condition)
# summary(physeq_met_sub$Body_mass_g)
# summary(physeq_met_sub$Year)
# summary(physeq_met_sub$Seq_run_comb)

pdat <- expand.grid(Day_of_year=seq(0,365,14), Age="A", Sex="F",
Reprod="inactive", Body_condition="2",
                    Body_mass_g=20.24,
```

```r
Sampling_year=c("Year15_16","Year16_17","Year17_18"),
Seq_run_comb="Exp_Wyt_2")
p  <- predict(model1$gam,  newdata = pdat, type = "response", se.fit = TRUE,
level=1)
pdat$p <- p$fit
pdat$se <- p$se.fit
#shouldn't plot for sampling_year15_16 start of July-end Oct because these
#dates weren't trapped in that year (--> day of the year between 189-301)
remove <- subset(pdat, Day_of_year>165 & Day_of_year<285 &
Sampling_year=="Year15_16")
remove #rows 15-22
```

```
##    Day_of_year Age Sex   Reprod Body_condition Body_mass_g Sampling_year
## 13         168   A   F inactive              2       20.24     Year15_16
## 14         182   A   F inactive              2       20.24     Year15_16
## 15         196   A   F inactive              2       20.24     Year15_16
## 16         210   A   F inactive              2       20.24     Year15_16
## 17         224   A   F inactive              2       20.24     Year15_16
## 18         238   A   F inactive              2       20.24     Year15_16
## 19         252   A   F inactive              2       20.24     Year15_16
## 20         266   A   F inactive              2       20.24     Year15_16
## 21         280   A   F inactive              2       20.24     Year15_16
##    Seq_run_comb           p         se
## 13    Exp_Wyt_2 -0.09147808 0.05699065
## 14    Exp_Wyt_2 -0.05529245 0.06975367
## 15    Exp_Wyt_2 -0.01637227 0.08213484
## 16    Exp_Wyt_2  0.02169673 0.09114639
## 17    Exp_Wyt_2  0.05627595 0.09541742
## 18    Exp_Wyt_2  0.08455199 0.09434227
## 19    Exp_Wyt_2  0.10365751 0.08812144
## 20    Exp_Wyt_2  0.11076936 0.07731742
## 21    Exp_Wyt_2  0.10305915 0.06372281
```

```r
pdat$se[rownames(pdat)%in%rownames(remove)] <- NA
pdat$p[rownames(pdat)%in%rownames(remove)] <- NA
#pdat

#plot showing raw PC1 values
names(pdat)[names(pdat)=="p"] <- "Axis.1"
#summary(physeq_met_sub$Axis.1)#min=-0.34793, max=0.40633 (use to scale axis
acros plots)

p1 <- ggplot(pdat, aes(x = Day_of_year, y = -Axis.1, group=Sampling_year)) +
  geom_line(aes(colour=Sampling_year), size=1) +
  geom_ribbon(aes(ymin=-Axis.1-se, ymax=-Axis.1+se), alpha=0.15) +
  geom_point(data=physeq_met_sub, alpha=0.3, aes(colour=Sampling_year)) +
  theme_bw() +
  ylab("PC1 (12.89% variance)") +
  #ggtitle("Wytham") +
  theme(axis.text=element_text(size=13),axis.title=element_text(size=14),
```

```
      title = element_text(size=14), axis.text.x = element_blank(),
      axis.title.x = element_blank(),
      legend.position = "none")  +
  scale_x_continuous(breaks=cumsum(c(1,31,28,31,30,31,30,31,31,30,31,30,30)),
                     labels =c("Jan","Feb","Mar","Apr","May","Jun","Jul","Aug",
                               "Sep","Oct","Nov","Dec", "Jan"),
                     expand = c(0,0)) +
  ylim(-0.40633,0.34793) +
  scale_colour_manual(values=c("deepskyblue1", "dodgerblue1", "dodgerblue4"))
p1

## Warning: Removed 2 rows containing missing values (geom_point).
```

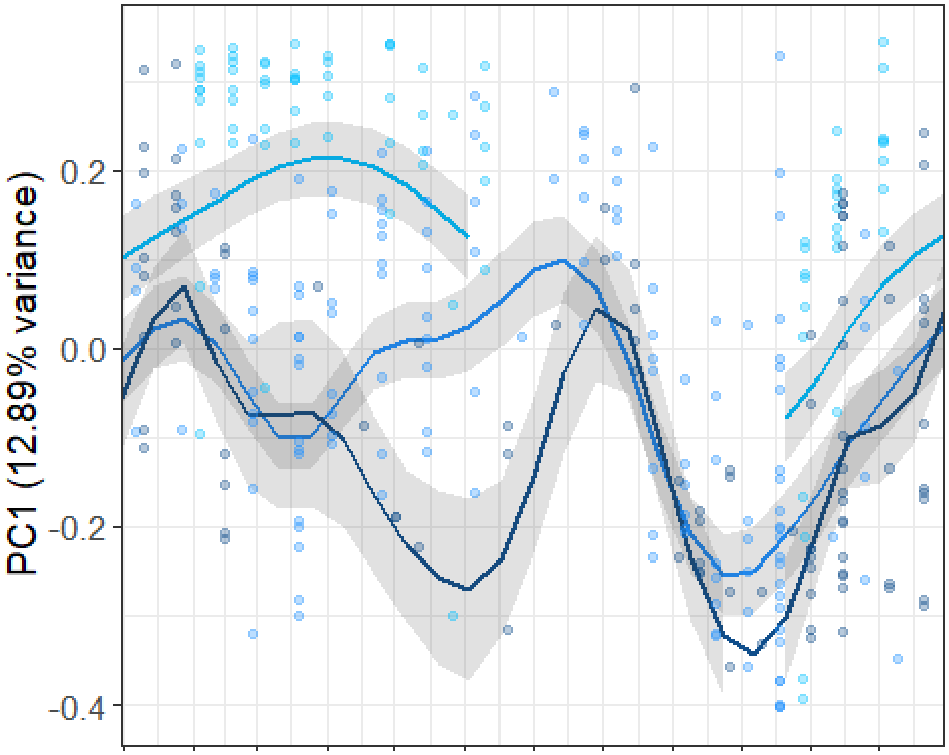

Wytham GAMM with Jaccard PC1

```
##GAMM with Jaccard PC1 instead of Bray-curtis
model1_jac <- gamm(JaccardPC1 ~ s(Day_of_year, bs = "cc", k = 24) + Year +
Sex + Reprod + Age + Body_mass_g + Body_condition + Seq_run_comb,
                data = physeq_met_sub,
                random=list(XPIT_tag=~1),
                na.action = na.exclude)

# #check model assumptions
# sresid <- model1_jac$gam$residuals
# hist(sresid)#
# fitted.glmm <- model1_jac$gam$fitted              # Extract the fitted
(predicted) values
# plot(sresid ~ fitted.glmm)                        # Check for homoscedasticity
```

```
# plot(sresid ~ physeq_met_sub$Day_of_year)  # plot the residuals against the
independent variable(s)

summary(model1_jac$gam)

##
## Family: gaussian
## Link function: identity
##
## Formula:
## JaccardPC1 ~ s(Day_of_year, bs = "cc", k = 24) + Year + Sex +
##     Reprod + Age + Body_mass_g + Body_condition + Seq_run_comb
##
## Parametric coefficients:
##                           Estimate Std. Error t value Pr(>|t|)
## (Intercept)               0.014179   0.071818   0.197   0.8436
## Year2016                 -0.073755   0.041065  -1.796   0.0735 .
## Year2017                 -0.214428   0.042336  -5.065 7.11e-07 ***
## Year2018                 -0.249382   0.058897  -4.234 3.04e-05 ***
## SexM                      0.009172   0.018100   0.507   0.6127
## Reprodactive              0.015011   0.020418   0.735   0.4628
## AgeJ                      0.022827   0.036225   0.630   0.5291
## AgeSA                     0.034719   0.025133   1.381   0.1682
## Body_mass_g               0.005376   0.002724   1.974   0.0493 *
## Body_condition1.5         0.033968   0.024036   1.413   0.1586
## Body_condition2           0.032449   0.024842   1.306   0.1925
## Body_condition2.5         0.054896   0.031865   1.723   0.0860 .
## Body_condition3           0.070965   0.038500   1.843   0.0663 .
## Seq_run_combExp_Wyt_2    -0.010123   0.020730  -0.488   0.6257
## Seq_run_combWyt_Diss_1    0.040826   0.030499   1.339   0.1817
## Seq_run_combWyt_Diss_2   -0.007997   0.030940  -0.258   0.7962
## ---
## Signif. codes:  0 '***' 0.001 '**' 0.01 '*' 0.05 '.' 0.1 ' ' 1
##
## Approximate significance of smooth terms:
##                  edf Ref.df    F p-value
## s(Day_of_year) 8.503     22 7.85  <2e-16 ***
## ---
## Signif. codes:  0 '***' 0.001 '**' 0.01 '*' 0.05 '.' 0.1 ' ' 1
##
## R-sq.(adj) =   0.57
##   Scale est. = 0.013065  n = 328

anova(model1_jac$gam)

##
## Family: gaussian
## Link function: identity
##
## Formula:
```

```
## JaccardPC1 ~ s(Day_of_year, bs = "cc", k = 24) + Year + Sex +
##     Reprod + Age + Body_mass_g + Body_condition + Seq_run_comb
##
## Parametric Terms:
##                df      F  p-value
## Year            3 15.553 1.95e-09
## Sex             1  0.257   0.6127
## Reprod          1  0.541   0.4628
## Age             2  0.976   0.3780
## Body_mass_g     1  3.896   0.0493
## Body_condition  4  1.107   0.3534
## Seq_run_comb    3  1.669   0.1736
##
## Approximate significance of smooth terms:
##                  edf Ref.df    F p-value
## s(Day_of_year)  8.503 22.000 7.85  <2e-16

pdat <- expand.grid(Day_of_year=seq(0,365,14), Age="A", Sex="F",
Reprod="inactive", Body_condition="2",
                    Body_mass_g=20.24, Year="2017", Seq_run_comb="Exp_Wyt_2")
p  <- predict(model1_jac$gam,  newdata = pdat, type = "response", se.fit =
TRUE, level=0)
pdat$p <- p$fit
pdat$se <- p$se.fit

#plot showing raw PC1 values
names(pdat)[names(pdat)=="p"] <- "JaccardPC1"

ggplot(pdat, aes(x = Day_of_year, y = JaccardPC1)) +
  geom_line(colour="dodgerblue", size=1) +
  geom_ribbon(aes(ymin=JaccardPC1-se, ymax=JaccardPC1+se), alpha=0.15) +
  geom_point(data=physeq_met_sub, alpha=0.3) +
  theme_bw() +
  xlab("Month") + ylab("Jaccard PC1 (15.77% variance)") +
  ggtitle("Wytham") +
  theme(axis.text.x=element_text(size=12),axis.title=element_text(size=14),
        title = element_text(size=14))  +
  scale_x_continuous(breaks=cumsum(c(1,31,28,31,30,31,30,31,31,30,31,30,30)),
                     labels
=c("Jan","Feb","Mar","Apr","May","Jun","Jul","Aug",
                              "Sep","Oct","Nov","Dec", "Jan"),
                     expand = c(0,0))
```

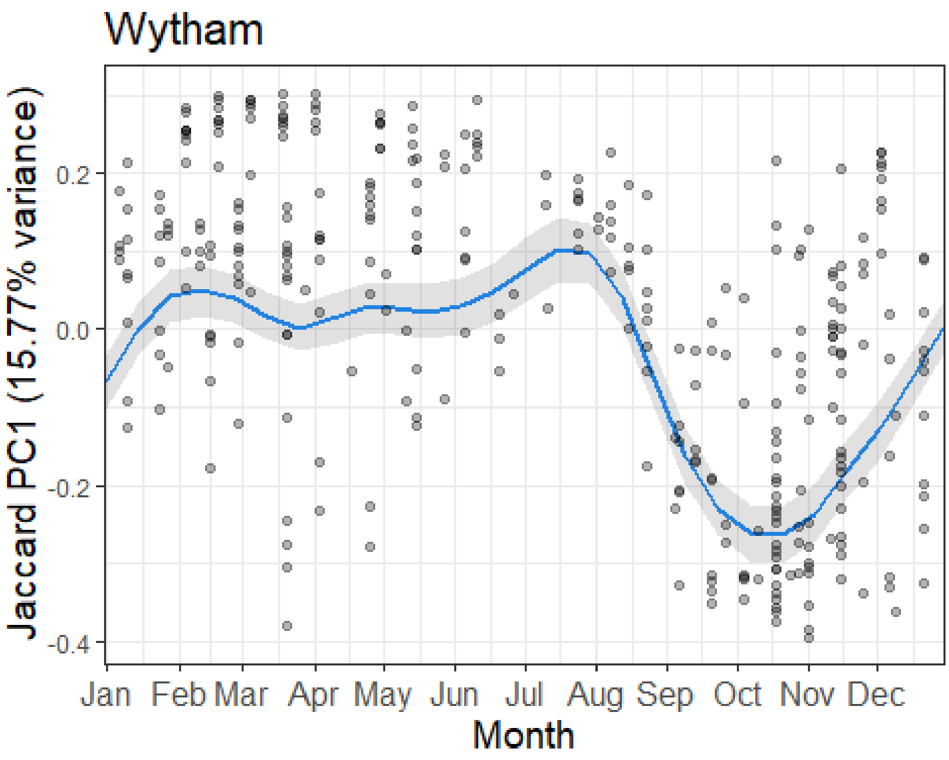

Check seasonal patterns in other PCoA axes

```
physeq_PC_sub <- physeq_met[,c(56:58, 65)]
colnames(physeq_PC_sub) <- c("PC1 (12.89%)", "PC2 (9.41%)", "PC3 (6.23%)",
"Day_of_year")
physeq_PC_sub$`PC1 (12.89%)` <- -(physeq_PC_sub$`PC1 (12.89%)`)
physeq_PC_sub_melt <- melt(physeq_PC_sub, id.vars = "Day_of_year")

ggplot(physeq_PC_sub_melt, aes(x=Day_of_year, y=value)) +
  facet_wrap(~variable, scales = "free_y") +
  geom_point() +
  stat_smooth() +
  theme_bw() +
  xlab("Month") + ylab("PCoA value") + ggtitle("Wytham") +
  scale_x_continuous(breaks=cumsum(c(1,31,28,31,30,31,30,31,31,30,31,30,30)),
                     labels
=c("Jan","Feb","Mar","Apr","May","Jun","Jul","Aug",
                          "Sep","Oct","Nov","Dec", "Jan"),
                     expand = c(0,0)) +
  theme(axis.title = element_text(size=14), axis.text =
element_text(size=12),
        strip.text = element_text(size=14), title = element_text(size=14))

## `geom_smooth()` using method = 'loess' and formula 'y ~ x'
```

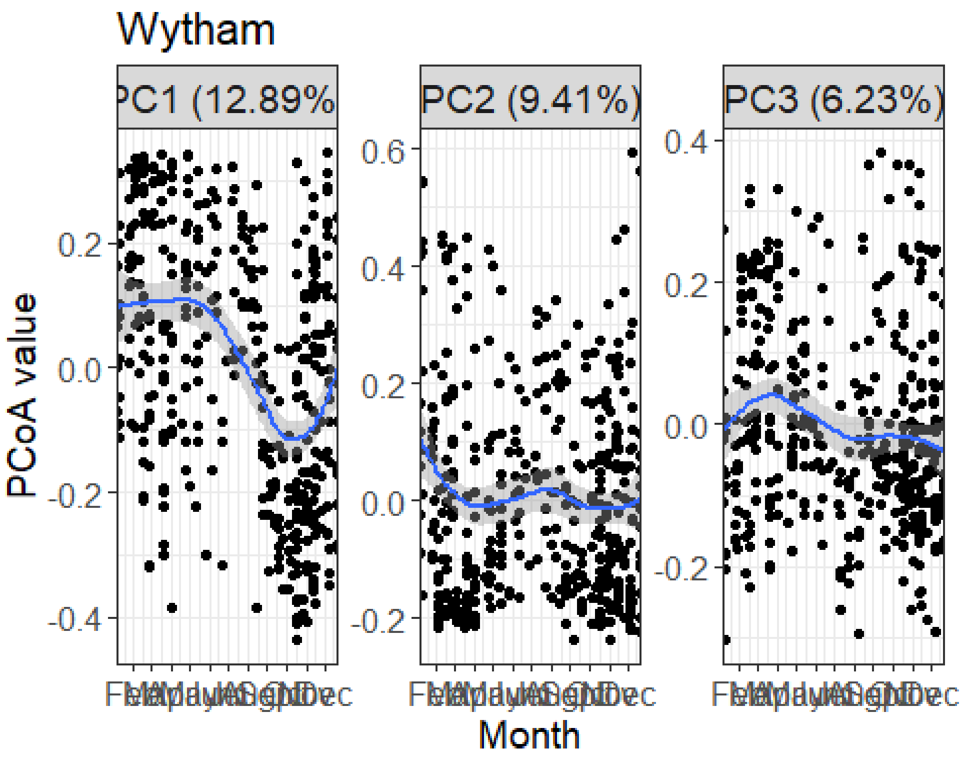

Plot within-individual changes between july-oct-feb

```
#find mice captured in July, October and February and plot within-individual
#change in pC1 between these months
#group months into jun/july / oct/nov and jan/feb for each year

MonthJ_A <- c("June", "July", "August")
MonthO_N <- c("September", "October", "November")
MonthJ_F <- c( "January", "February", "March")
physeq_met$Month_group<- "NA"
physeq_met[which(physeq_met$Month%in%MonthJ_A),]$Month_group<-"Jun_Aug"
physeq_met[which(physeq_met$Month%in%MonthO_N),]$Month_group<-"Sept_Nov"
physeq_met[which(physeq_met$Month%in%MonthJ_F),]$Month_group<-"Jan_March"

physeq_met$Month_group <- as.factor(physeq_met$Month_group)
levels(physeq_met$Month_group)[levels(physeq_met$Month_group)=='NA'] <- NA

physeq_met_month_sub <- subset(physeq_met, Month_group!="NA")
#choose one sample per mouse per month
physeq_met_mouse_sub <- physeq_met_month_sub %>% group_by(Month_group,
XPIT_tag) %>% sample_n(1)
#remove mice with only one sample
mouse_recaps <-
as.data.frame(summary(as.factor(physeq_met_mouse_sub$XPIT_tag), maxsum =
300))
mouse_recaps_multi <- subset(mouse_recaps,
```

```
summary(physeq_met_mouse_sub$XPIT_tag, maxsum = 300)>1)
mouse_recap_list <- as.list(rownames(mouse_recaps_multi))
physeq_met_recaps <- subset(physeq_met_mouse_sub,
XPIT_tag%in%mouse_recap_list)
physeq_met_recaps$XPIT_tag <- as.factor(physeq_met_recaps$XPIT_tag)
levels(physeq_met_recaps$XPIT_tag)[levels(physeq_met_recaps$XPIT_tag)==''] <-
NA
wyt_physeq_met_recaps <- subset(physeq_met_recaps, XPIT_tag!="NA")

level_order5 <- c("Jun_Aug", "Sept_Nov", "Jan_March")

#remove the feb-jul within year comparison - X0419180163, X04191807F4,
X041917FDD5,X041917E2C0, X04191808A7
#Ones that don't follow population-level trend;
#X0418EBC072, X041917F99A, X04191829A9, X0419182DBC, X041894C6C7
against_pop <- c("X041917F99A", "X0419182DBC", "X041894C6C7", "X0418EBBF1E",
"X0418EBB77E", "X0419182B6F", "X04191825F4")
wyt_physeq_met_recaps$JOF_trend<- "with_pop"
wyt_physeq_met_recaps[which(wyt_physeq_met_recaps$XPIT_tag%in%against_pop),]$
JOF_trend<-"against_pop"

p3 <- ggplot(subset(wyt_physeq_met_recaps, XPIT_tag!="X0419180163" &
XPIT_tag!="X041917FDD5" & XPIT_tag!="X04191807F4" &
                XPIT_tag!="X041917E2C0" & XPIT_tag!="X04191808A7"),
       aes(x=factor(Month_group, levels = level_order5), y=-Axis.1,
group=XPIT_tag)) +
  geom_point(aes(colour=JOF_trend)) +
  geom_line(aes(colour=JOF_trend)) +
  theme_bw() +
  ylab("PC1") +
  #ggtitle("Wytham") +
  ylab("PC1 (12.89% variance)") +
  xlab("Time of year") +
  scale_color_manual(values = c("grey", "dodgerblue")) +
  theme(legend.position = "none", axis.text = element_text(size=14),
axis.title = element_text(size=16),
        axis.title.x = element_blank(), axis.title.y = element_blank(),
        axis.text.x = element_blank(), axis.text.y = element_blank()) +
  scale_x_discrete(labels=c("Jun_Aug"="Jun-Aug", "Sept_Nov"="Sept-Nov",
"Jan_March"="Jan-March")) +
  ylim(-0.40633,0.34793)
p3
```

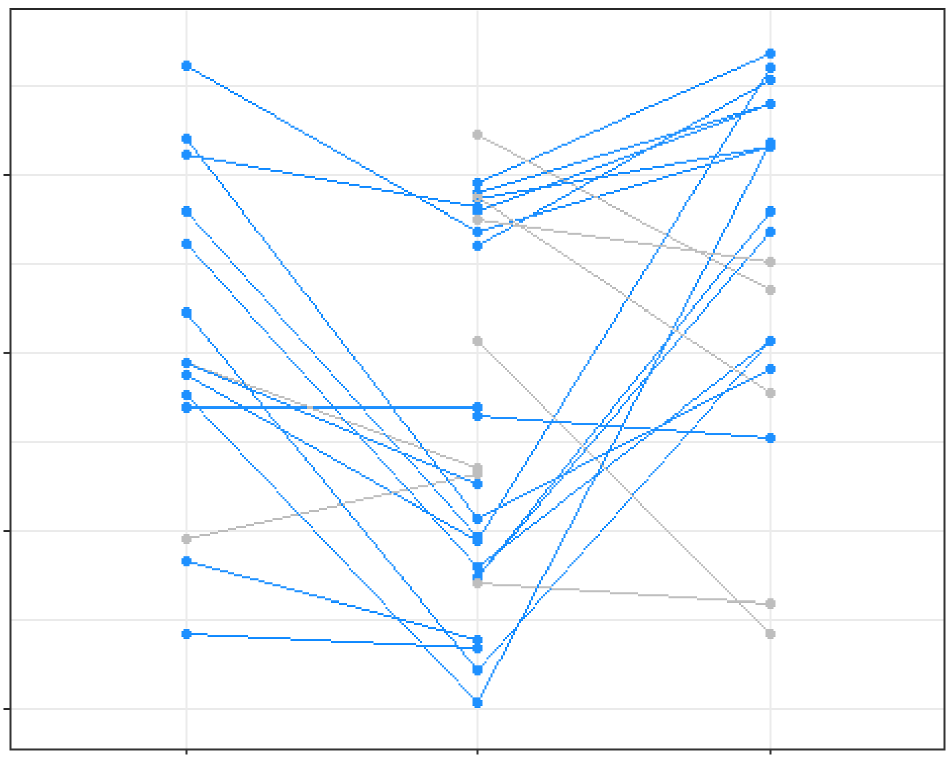

```
wyt_physeq_met_recaps <- as.data.frame(wyt_physeq_met_recaps)
#saveRDS(wyt_physeq_met_recaps,
'~/Wytham_season_PC1_individual_shifts_random_sampling_per_month_JOF_plot.rds
')

#test within-individual changes -paired 2-sample t test
hist(wyt_physeq_met_recaps$Axis.1)
```

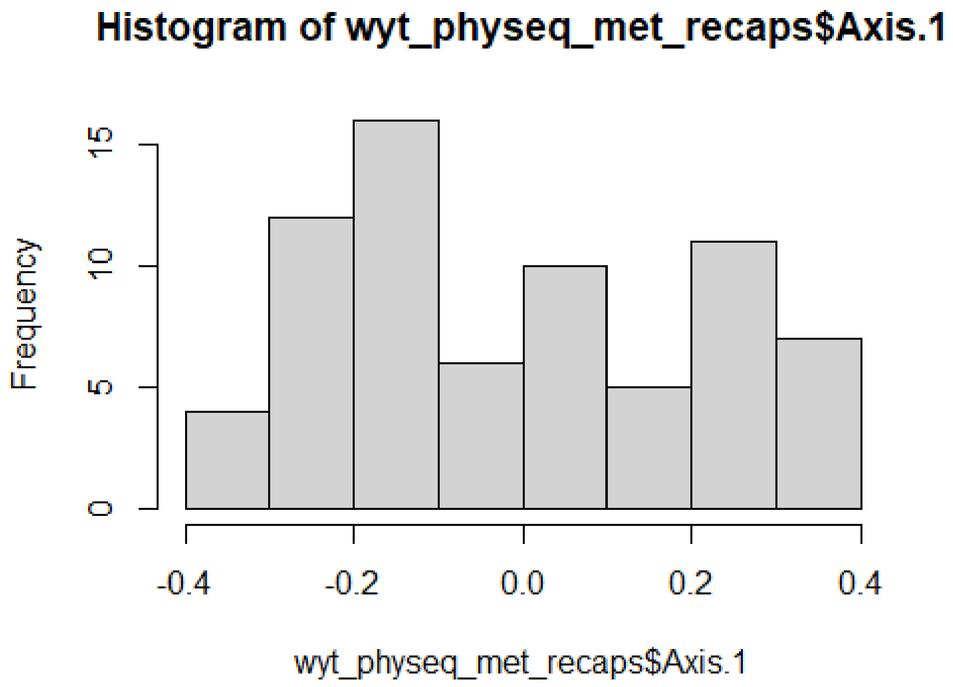


```
colnames(wyt_physeq_met_recaps) #43,51,60

##  [1] "Sequence_sample_code"            "X"
##  [3] "Collection_date"                 "Grid"
##  [5] "XPIT_tag"                        "Animal_ID"
##  [7] "Trap_num"                        "Grid_sq"
##  [9] "Species.x"                       "Year"
## [11] "Month"                           "Zymo_FS_DNA_extraction_YN"
## [13] "Extraction_round"                "Extraction_batch.x"
## [15] "Extraction_sample_code"          "Poo_mass_mg_used_Zymo_extraction"
## [17] "Sequenced_YN"                    "Sequencing_round.x"
## [19] "Miseq_run.x"                     "SCFA_sample_YN"
## [21] "Habitat_type"                    "N_R"
## [23] "Sex"                             "Reprod"
## [25] "Age"                             "PIT_tag"
## [27] "Body_mass_g"                     "AGD_1"
## [29] "AGD_2"                           "AGD_3"
## [31] "Foot_1"                          "Foot_2"
## [33] "Foot_3"                          "Tick_L"
## [35] "Tick_N"                          "Tick_A"
## [37] "Fleas"                           "Mites"
## [39] "Lept"                            "Body_condition"
## [41] "Peanuts_left"                    "Apple_left"
## [43] "X.1"                             "X.2"
## [45] "X.3"                             "X.4"
## [47] "X.5"                             "Mouse.ID"
## [49] "Species.y"                       "Diet_Month"
```

```
## [51] "Extraction_batch.y"                  "Sequencing_round.y"
## [53] "Miseq_run.y"                         "System"
## [55] "readDepth"                           "Axis.1"
## [57] "Axis.2"                              "Axis.3"
## [59] "Axis.4"                              "Axis.5"
## [61] "Axis.6"                              "JaccardPC1"
## [63] "M_Y"                                 "Sampling_year"
## [65] "Day_of_year"                         "Seq_run_comb"
## [67] "Month_group"                         "JOF_trend"
```

```
physeq_meta_test1 <- wyt_physeq_met_recaps[,c(5,56,67)]

physeq_meta_J_O_test <- subset(physeq_meta_test1, Month_group=="Jun_Aug"
|Month_group=="Sept_Nov" )
test.dat.mat <- acast(physeq_meta_J_O_test, XPIT_tag ~ Month_group, value.var
= "Axis.1")
test.dat.mat <- as.data.frame(test.dat.mat)
nrow(subset(test.dat.mat, !is.na(Jun_Aug) & !is.na(Sept_Nov)))
```

```
## [1] 14
```

```
t.test(test.dat.mat$Jun_Aug, test.dat.mat$Sept_Nov, paired = T,
na.action=na.omit)
```

```
##
##  Paired t-test
##
## data:  test.dat.mat$Jun_Aug and test.dat.mat$Sept_Nov
## t = -4.2219, df = 13, p-value = 0.000998
## alternative hypothesis: true difference in means is not equal to 0
## 95 percent confidence interval:
##  -0.28216960 -0.09114355
## sample estimates:
## mean of the differences
##              -0.1866566
```

```
physeq_meta_OF_test <- subset(physeq_meta_test1, Month_group=="Jan_March"
|Month_group=="Sept_Nov" )
test.dat.mat <- acast(physeq_meta_OF_test, XPIT_tag ~ Month_group, value.var
= "Axis.1")
test.dat.mat <- as.data.frame(test.dat.mat)
nrow(subset(test.dat.mat, !is.na(Jan_March) & !is.na(Sept_Nov)))
```

```
## [1] 20
```

```
t.test(test.dat.mat$Jan_March, test.dat.mat$Sept_Nov, paired = T,
na.action=na.omit)
```

```
##
##  Paired t-test
##
## data:  test.dat.mat$Jan_March and test.dat.mat$Sept_Nov
```

```
## t = -2.7044, df = 19, p-value = 0.01406
## alternative hypothesis: true difference in means is not equal to 0
## 95 percent confidence interval:
##  -0.27565129 -0.03512736
## sample estimates:
## mean of the differences
##              -0.1553893
```

##Silwood

Ordination and PC1 GAMM

```
#ordinate - silwood (Bray-Curtis)
sample_ord_bray_sil <- ordinate(ps.sil.ra, method= "PCoA", distance= 'bray')
##extract first few axes as variables in metadata
axis_sub_sil<- sample_ord_bray_sil$vectors[,1:6]
axis_sub_sil <- cbind(rownames(axis_sub_sil), axis_sub_sil)
colnames(axis_sub_sil)[1] <- "Sequence_sample_code"
axis_sub_sil <- as.data.frame(axis_sub_sil)
#ordinate - Silwood (Jaccard)
ps.sil.ra.bin <- transform_sample_counts(ps.sil.ra, function(x) 1*(x>0))
sample_ord_jac_sil <- ordinate(ps.sil.ra.bin, method= "PCoA", distance=
'jaccard')
##extract first few axes as variables in metadata
axis_sub_sil_jac<- sample_ord_jac_sil$vectors[,1]
axis_sub_sil_jac <- as.data.frame(axis_sub_sil_jac)
axis_sub_sil_jac <- cbind(rownames(axis_sub_sil_jac), axis_sub_sil_jac)
colnames(axis_sub_sil_jac) <- c("Sequence_sample_code", "JaccardPC1")
axis_sub_sil_comb <- merge(axis_sub_sil, axis_sub_sil_jac,
by="Sequence_sample_code")

physeq_met_sil <- as(sample_data(ps.sil.ra), "data.frame")
physeq_merge <- merge(physeq_met_sil, axis_sub_sil_comb,
by="Sequence_sample_code")
rownames(physeq_merge) <- physeq_merge$Sequence_sample_code
ps.sil.ra <- phyloseq(sample_data(physeq_merge),
                      otu_table(otu_table(ps.sil.ra)),
                      tax_table(tax_table(ps.sil.ra)))

sample_data(ps.sil.ra)$Axis.1 <-
as.numeric(as.character(sample_data(ps.sil.ra)$Axis.1))
sample_data(ps.sil.ra)$Axis.2 <-
as.numeric(as.character(sample_data(ps.sil.ra)$Axis.2))
sample_data(ps.sil.ra)$Axis.3 <-
as.numeric(as.character(sample_data(ps.sil.ra)$Axis.3))
sample_data(ps.sil.ra)$Axis.4 <-
as.numeric(as.character(sample_data(ps.sil.ra)$Axis.4))
sample_data(ps.sil.ra)$Axis.5 <-
as.numeric(as.character(sample_data(ps.sil.ra)$Axis.5))
sample_data(ps.sil.ra)$Axis.6 <-
```

```
as.numeric(as.character(sample_data(ps.sil.ra)$Axis.6))
sample_data(ps.sil.ra)$JaccardPC1 <-
as.numeric(as.character(sample_data(ps.sil.ra)$JaccardPC1))

physeq_met_sil <- as(sample_data(ps.sil.ra), 'data.frame')
physeq_met_sil$date <- as.character(physeq_met_sil$date)
physeq_met_sil$date <- as.Date(physeq_met_sil$date, format= "%d/%m/%Y") #now
collection date is recognised as in date format
#now get day of the year
physeq_met_sil$Day_of_year <- format(physeq_met_sil$date, "%j")
physeq_met_sil$Day_of_year <- as.numeric(physeq_met_sil$Day_of_year)
# summary(physeq_met_sil$ID)
# summary(physeq_met_sil$Sex)
# summary(physeq_met_sil$Body_mass_grams)
# summary(physeq_met_sil$Age)
# summary(physeq_met_sil$Reprod)
physeq_met_sil$Reprod <- physeq_met_sil$Reprod%>% fct_collapse(
  active = c("NIPPLES", "PERF", "PREG", "TL", "TL+"),
  inactive = c("IMP", "TA", "TS"))

#summary(physeq_met_sil$Body_condition)
physeq_met_sil$Body_condition <- as.factor(physeq_met_sil$Body_condition)
#summary(physeq_met_sil$Miseq_run)
physeq_met_sil$Miseq_run <- as.factor(physeq_met_sil$Miseq_run)

physeq_met_sil2 <- subset(physeq_met_sil, months!="Nov-14" &
!is.na(Body_condition) & !is.na(Body_mass_grams))

model_sil <- gamm(Axis.1 ~ s(Day_of_year, k=18) + Age + Sex + Reprod +
Body_condition + Body_mass_grams,
                   family=gaussian(link="identity"),
                   random=list(ID=~1),
                   data=physeq_met_sil2,
                   na.action = na.exclude)

#check model assumptions
# sresid <- model_sil$gam$residuals
# hist(sresid)
# fitted.glmm <- model_sil$gam$fitted              # Extract the fitted
(predicted) values
# plot(sresid ~ fitted.glmm)                       # Check for homoscedasticity
# plot(sresid ~ physeq_met_sil2$Day_of_year)  # plot the residuals against
the independent variable(s)

summary(model_sil$gam)

##
## Family: gaussian
## Link function: identity
```

```
##
## Formula:
## Axis.1 ~ s(Day_of_year, k = 18) + Age + Sex + Reprod + Body_condition +
##     Body_mass_grams
##
## Parametric coefficients:
##                     Estimate Std. Error t value Pr(>|t|)
## (Intercept)        -0.174980   0.091757  -1.907   0.0580 .
## AgeJ                0.087104   0.077224   1.128   0.2608
## AgeSu               0.070904   0.038915   1.822   0.0700 .
## SexM                0.002579   0.029782   0.087   0.9311
## Reprodactive        0.016028   0.038843   0.413   0.6803
## Body_condition1.5  -0.006444   0.028975  -0.222   0.8242
## Body_condition1.75 -0.185948   0.096703  -1.923   0.0560 .
## Body_condition2    -0.024707   0.038181  -0.647   0.5184
## Body_condition2.5  -0.034443   0.080920  -0.426   0.6708
## Body_condition3    -0.016640   0.097495  -0.171   0.8647
## Body_mass_grams     0.008953   0.004205   2.129   0.0345 *
## ---
## Signif. codes:  0 '***' 0.001 '**' 0.01 '*' 0.05 '.' 0.1 ' ' 1
##
## Approximate significance of smooth terms:
##                  edf Ref.df     F p-value
## s(Day_of_year) 6.46   6.46 13.79  <2e-16 ***
## ---
## Signif. codes:  0 '***' 0.001 '**' 0.01 '*' 0.05 '.' 0.1 ' ' 1
##
## R-sq.(adj) =  0.417
##   Scale est. = 0.021066  n = 209
```

```
anova(model_sil$gam)
```

```
##
## Family: gaussian
## Link function: identity
##
## Formula:
## Axis.1 ~ s(Day_of_year, k = 18) + Age + Sex + Reprod + Body_condition +
##     Body_mass_grams
##
## Parametric Terms:
##                  df     F p-value
## Age               2 1.735  0.1792
## Sex               1 0.007  0.9311
## Reprod            1 0.170  0.6803
## Body_condition    5 0.803  0.5488
## Body_mass_grams   1 4.533  0.0345
##
## Approximate significance of smooth terms:
```

```
##                   edf Ref.df     F p-value
## s(Day_of_year) 6.46   6.46 13.79  <2e-16
```

```
summary(model_sil$mer)
```

```
## Length  Class   Mode 
##      0   NULL   NULL
```

```
#Plot fitted values
# summary(physeq_met_sil$Day_of_year) #min=15 max=345
# summary(phseq_met_sil2$Body_condition)
# summary(phseq_met_sil2$Body_mass_grams)
pdat <- expand.grid(Day_of_year=seq(0,365,14), Age="A", Sex="F",
Reprod="active", Body_condition="2", Body_mass_grams=19.19)

pred <- predict (model_sil$gam, newdata = pdat, na.rm = T,
                 type= "response", se.fit = TRUE, level=0)
predframe <- data.frame (pdat, preds = pred$fit, se = pred$se.fit)
predframe
```

```
##    Day_of_year Age Sex Reprod Body_condition Body_mass_grams       preds
## 1            0   A   F active              2           19.19 -0.08125623
## 2           14   A   F active              2           19.19 -0.09072533
## 3           28   A   F active              2           19.19 -0.09867770
## 4           42   A   F active              2           19.19 -0.09778992
## 5           56   A   F active              2           19.19 -0.08566855
## 6           70   A   F active              2           19.19 -0.07078464
## 7           84   A   F active              2           19.19 -0.06786210
## 8           98   A   F active              2           19.19 -0.07808687
## 9          112   A   F active              2           19.19 -0.09667903
## 10         126   A   F active              2           19.19 -0.11698938
## 11         140   A   F active              2           19.19 -0.13227170
## 12         154   A   F active              2           19.19 -0.14597160
## 13         168   A   F active              2           19.19 -0.16533821
## 14         182   A   F active              2           19.19 -0.17397314
## 15         196   A   F active              2           19.19 -0.15812139
## 16         210   A   F active              2           19.19 -0.11159288
## 17         224   A   F active              2           19.19 -0.04164459
## 18         238   A   F active              2           19.19  0.03916187
## 19         252   A   F active              2           19.19  0.11586847
## 20         266   A   F active              2           19.19  0.17484367
## 21         280   A   F active              2           19.19  0.20692421
## 22         294   A   F active              2           19.19  0.20881154
## 23         308   A   F active              2           19.19  0.18136201
## 24         322   A   F active              2           19.19  0.13451071
## 25         336   A   F active              2           19.19  0.07572688
## 26         350   A   F active              2           19.19  0.01213083
## 27         364   A   F active              2           19.19 -0.05179280
##            se
## 1  0.08264547
## 2  0.06551233
```

```
## 3  0.06057214
## 4  0.06051538
## 5  0.06003841
## 6  0.05918333
## 7  0.05855451
## 8  0.05797750
## 9  0.05741625
## 10 0.05743084
## 11 0.05595058
## 12 0.05469475
## 13 0.05428312
## 14 0.05526899
## 15 0.05648292
## 16 0.05530223
## 17 0.05530474
## 18 0.05666014
## 19 0.05672637
## 20 0.05699170
## 21 0.05799382
## 22 0.06014527
## 23 0.06262184
## 24 0.06378953
## 25 0.06287537
## 26 0.06968198
## 27 0.08707140
```

```
summary(physeq_met_sil2$Axis.1)#min=-0.44869, max=0.44622
```

```
##     Min.  1st Qu.   Median     Mean  3rd Qu.     Max.
## -0.46850 -0.16132  0.03497  0.01881  0.21937  0.42972
```

```
p2 <- ggplot(physeq_met_sil2, aes(x = Day_of_year, y = -Axis.1)) +
  geom_point(alpha=0.3) +
  xlab("Month") + ylab("PC1 (20% of variance)") +
  #ggtitle("Silwood") +
  theme_bw() +
  geom_line(aes(y=-preds), data=predframe, colour="springgreen3", size=1) +
  geom_ribbon(data = predframe, aes(y = NULL, ymin = -preds-se, ymax = -
preds+se), alpha=0.15) +
  theme(axis.text=element_text(size=14),axis.title=element_text(size=16),
        title = element_text(size=16), axis.text.y = element_text(size=11),
        axis.title.y=element_text(size=15))  +
  scale_x_continuous(breaks=cumsum(c(1,31,28,31,30,31,30,31,31,30,31,30,30)),
                     labels
=c("Jan","Feb","Mar","Apr","May","Jun","Jul","Aug",
                               "Sep","Oct","Nov","Dec", "Jan"),
                     expand = c(0,0)) +
  ylim(-0.44622,0.44869)
p2
```

```
## Warning: Removed 2 rows containing missing values (geom_point).
```

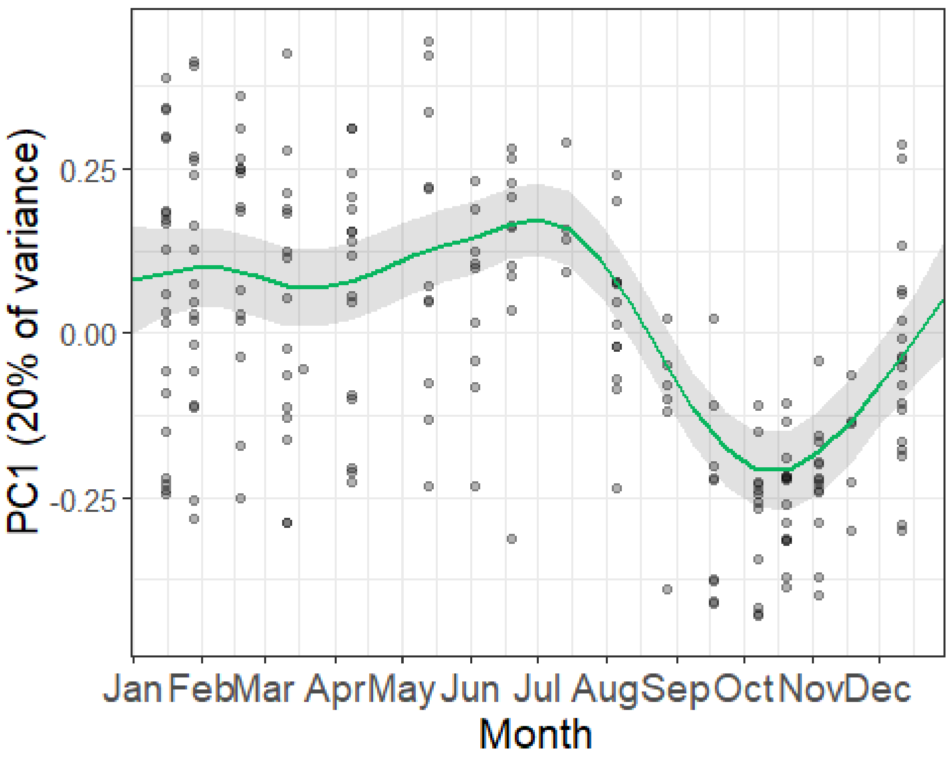

Silwood PC1 GAMM with Jaccard

```
model_sil_jac <- gamm(JaccardPC1 ~ s(Day_of_year, k = 18) + Age + Sex +
Reprod +
                        Body_condition + Body_mass_grams,
                    data = physeq_met_sil2,
                    random=list(ID=~1),
                    na.action = na.exclude)

#check model assumptions
# sresid <- model_sil_jac$gam$residuals
# hist(sresid)
# fitted.glmm <- model_sil_jac$gam$fitted              # Extract the fitted
(predicted) values
# plot(sresid ~ fitted.glmm)                          # Check for homoscedasticity
# plot(sresid ~ physeq_met_sil2$Day_of_year)  # plot the residuals against
the independent variable(s)

summary(model_sil_jac$mer)

## Length  Class   Mode
##      0   NULL   NULL

summary(model_sil_jac$gam)

##
## Family: gaussian
```

```
## Link function: identity
##
## Formula:
## JaccardPC1 ~ s(Day_of_year, k = 18) + Age + Sex + Reprod + Body_condition
+
##     Body_mass_grams
##
## Parametric coefficients:
##                      Estimate Std. Error t value Pr(>|t|)
## (Intercept)        -0.082193   0.036357  -2.261   0.0249 *
## AgeJ                0.030600   0.029593   1.034   0.3025
## AgeSu              -0.002688   0.014749  -0.182   0.8556
## SexM                0.006786   0.012459   0.545   0.5866
## Reprodactive        0.019248   0.014414   1.335   0.1834
## Body_condition1.5   0.003883   0.010566   0.368   0.7137
## Body_condition1.75  0.020800   0.034642   0.600   0.5489
## Body_condition2     0.002250   0.013801   0.163   0.8706
## Body_condition2.5  -0.054812   0.030009  -1.826   0.0694 .
## Body_condition3     0.008560   0.035664   0.240   0.8106
## Body_mass_grams     0.002208   0.001670   1.323   0.1876
## ---
## Signif. codes:  0 '***' 0.001 '**' 0.01 '*' 0.05 '.' 0.1 ' ' 1
##
## Approximate significance of smooth terms:
##                   edf Ref.df     F p-value
## s(Day_of_year) 12.27  12.27 82.25  <2e-16 ***
## ---
## Signif. codes:  0 '***' 0.001 '**' 0.01 '*' 0.05 '.' 0.1 ' ' 1
##
## R-sq.(adj) =  0.898
##   Scale est. = 0.0023965  n = 209
```

```
anova(model_sil_jac$gam)
```

```
##
## Family: gaussian
## Link function: identity
##
## Formula:
## JaccardPC1 ~ s(Day_of_year, k = 18) + Age + Sex + Reprod + Body_condition
+
##     Body_mass_grams
##
## Parametric Terms:
##                 df     F p-value
## Age              2 0.772   0.464
## Sex              1 0.297   0.587
## Reprod           1 1.783   0.183
## Body_condition   5 0.975   0.434
## Body_mass_grams  1 1.749   0.188
```

```
##
## Approximate significance of smooth terms:
##                    edf Ref.df     F p-value
## s(Day_of_year) 12.27  12.27 82.25  <2e-16

pdat <- expand.grid(Day_of_year=seq(0,350,14), Age="A", Sex="F",
Reprod="active", Body_condition="2", Body_mass_grams=19.19)
pred <- predict (model_sil_jac$gam, newdata = pdat, na.rm = T,
                 type= "response", se.fit = TRUE, level=0)
predframe <- data.frame (pdat, preds = pred$fit, se = pred$se.fit)

ggplot(physeq_met_sil2, aes(x = Day_of_year, y = JaccardPC1)) +
  geom_point(alpha=0.3) +
  xlab("Month") + ylab("Jaccard PC1 (8.40% of variance)") +
  ggtitle("Silwood") +
  theme_bw() +
  geom_line(aes(y=preds), data=predframe, colour="springgreen3", size=1) +
  geom_ribbon(data = predframe, aes(y = NULL, ymin = preds-se, ymax =
preds+se), alpha=0.15) +
  theme(axis.text.x=element_text(size=12),axis.title=element_text(size=14),
        title = element_text(size=14))  +
  scale_x_continuous(breaks=cumsum(c(1,31,28,31,30,31,30,31,31,30,31,30,30)),
                     labels
=c("Jan","Feb","Mar","Apr","May","Jun","Jul","Aug","Sep",
                          "Oct","Nov","Dec", "Jan"),
                     expand = c(0,0))
```

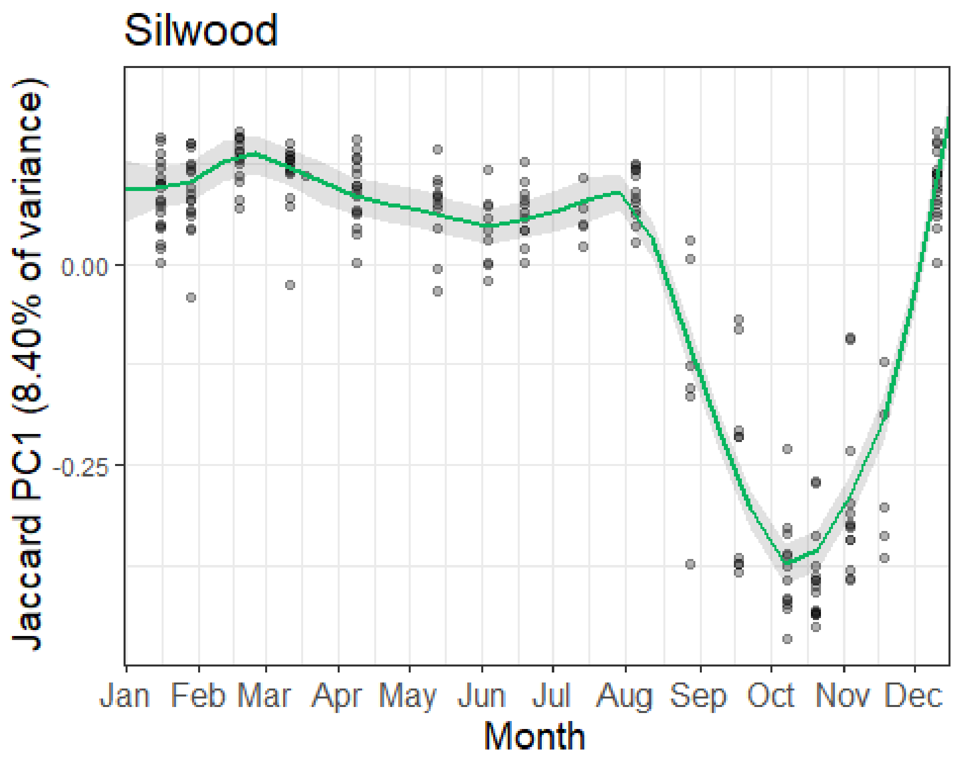

Check other axes for seasonality

```
physeq_PC_sub <- physeq_met_sil[,c(35:37, 42)]
colnames(physeq_PC_sub) <- c("PC1 (20%)", "PC2 (11.3%)", "PC3 (6.5%)",
"Day_of_year")
physeq_PC_sub$`PC1 (20%)` <- -(physeq_PC_sub$`PC1 (20%)`)
physeq_PC_sub_melt <- melt(physeq_PC_sub, id.vars = "Day_of_year")

ggplot(physeq_PC_sub_melt, aes(x=Day_of_year, y=value)) +
  facet_wrap(~variable, scales = "free_y") +
  geom_point() +
  stat_smooth() +
  theme_bw() +
  xlab("Month") + ylab("PCoA value") + ggtitle("Silwood") +
  scale_x_continuous(breaks=cumsum(c(1,31,28,31,30,31,30,31,31,30,31,30,30)),
                     labels
=c("Jan","Feb","Mar","Apr","May","Jun","Jul","Aug",
                                "Sep","Oct","Nov","Dec", "Jan"),
                     expand = c(0,0)) +
  theme(axis.title = element_text(size=14), axis.text =
element_text(size=12),
                                             strip.text =
element_text(size=14),
        title = element_text(size=14))

## `geom_smooth()` using method = 'loess' and formula 'y ~ x'
```

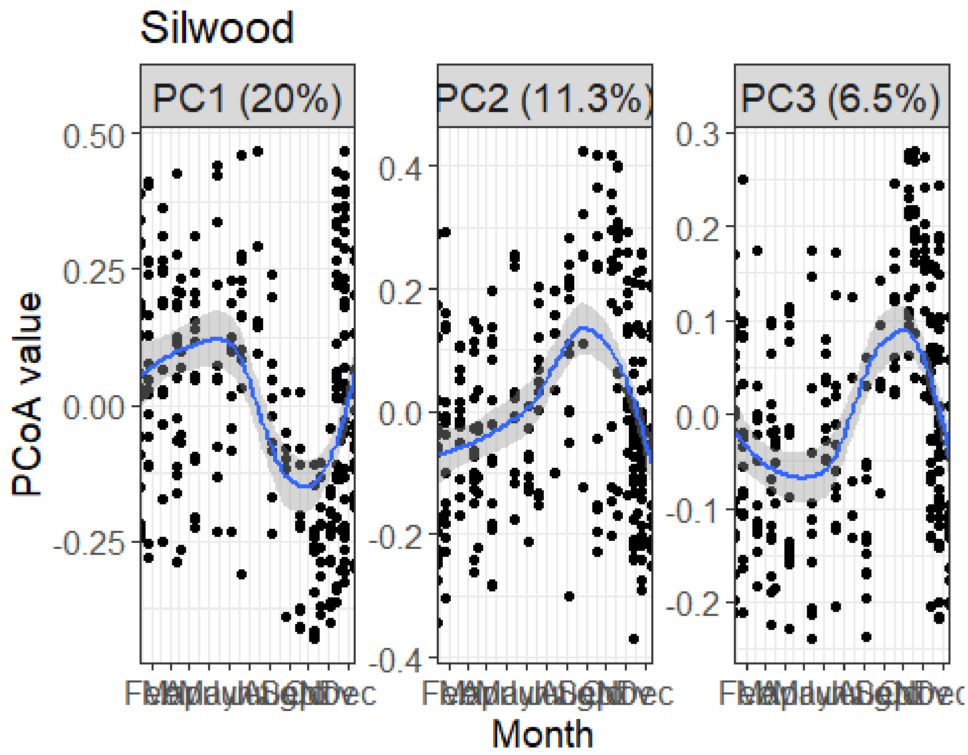

Plot within-individual changes between july-oct-feb

```
MonthJ_A <- c("June", "July", "August")
MonthO_N <- c("September", "October", "November")
MonthJ_F <- c( "January", "February", "March")
physeq_met_sil$Month_group<- "NA"
physeq_met_sil[which(physeq_met_sil$month%in%MonthJ_A),]$Month_group<-
"Jun_Aug"
physeq_met_sil[which(physeq_met_sil$month%in%MonthO_N),]$Month_group<-
"Sept_Nov"
physeq_met_sil[which(physeq_met_sil$month%in%MonthJ_F),]$Month_group<-
"Jan_March"

physeq_met_sil$Month_group <- as.factor(physeq_met_sil$Month_group)
levels(physeq_met_sil$Month_group)[levels(physeq_met_sil$Month_group)=='NA']
<- NA
physeq_met_month_sub <- subset(physeq_met_sil, Month_group!="NA")#
#choose one sample per mouse per month
physeq_met_mouse_sub <- physeq_met_month_sub %>% group_by(Month_group, ID)
%>% sample_n(1)
#remove mice with only one sample
mouse_recaps <- as.data.frame(summary(as.factor(physeq_met_mouse_sub$ID),
maxsum = 300))
mouse_recaps_multi <- subset(mouse_recaps, summary(physeq_met_mouse_sub$ID,
maxsum = 300)>1)
mouse_recap_list <- as.list(rownames(mouse_recaps_multi))
physeq_met_recaps <- subset(physeq_met_mouse_sub, ID%in%mouse_recap_list) #77
obs
levels(physeq_met_recaps$ID)[levels(physeq_met_recaps$ID)==''] <- NA
sil_physeq_met_recaps <- subset(physeq_met_recaps, ID!="NA")

level_order5 <- c("Jun_Aug", "Sept_Nov", "Jan_March")

#remove the feb-jul within year comparison - M904
#Ones that don't follow population-level trend;
#M842,M845,M899,M879, M873A,M662,M802,M756
against_pop <- c("M845","M899","M882","M879","M662","M802","M756", "M900",
"M870", "M878", "M889")
sil_physeq_met_recaps$JOF_trend<- "with_pop"
sil_physeq_met_recaps[which(sil_physeq_met_recaps$ID%in%against_pop),]$JOF_tr
end<-"against_pop"

p4 <- ggplot(subset(sil_physeq_met_recaps, ID!="M904"),
       aes(x=factor(Month_group, levels = level_order5), y=-Axis.1,
group=ID)) +
  geom_point(aes(colour=JOF_trend)) +
  geom_line(aes(colour=JOF_trend)) +
  theme_bw() +
  ylab("PC1") +
  #ggtitle("Silwood") +
```

```
  ylab("PC1 (20% variance)") +
  xlab("Time of year") +
  scale_color_manual(values = c("grey", "springgreen3")) +
  theme(legend.position = "none", axis.text = element_text(size=14),
        axis.title = element_text(size=16),
        axis.title.y = element_blank(), axis.text.y = element_blank()) +
  scale_x_discrete(labels=c("Jun_Aug"="Jun-Aug", "Sept_Nov"="Sept-Nov",
                            "Jan_March"="Jan-March"))+
  ylim(-0.44622,0.44869)
p4

## Warning: Removed 2 rows containing missing values (geom_point).

## Warning: Removed 1 row(s) containing missing values (geom_path).
```

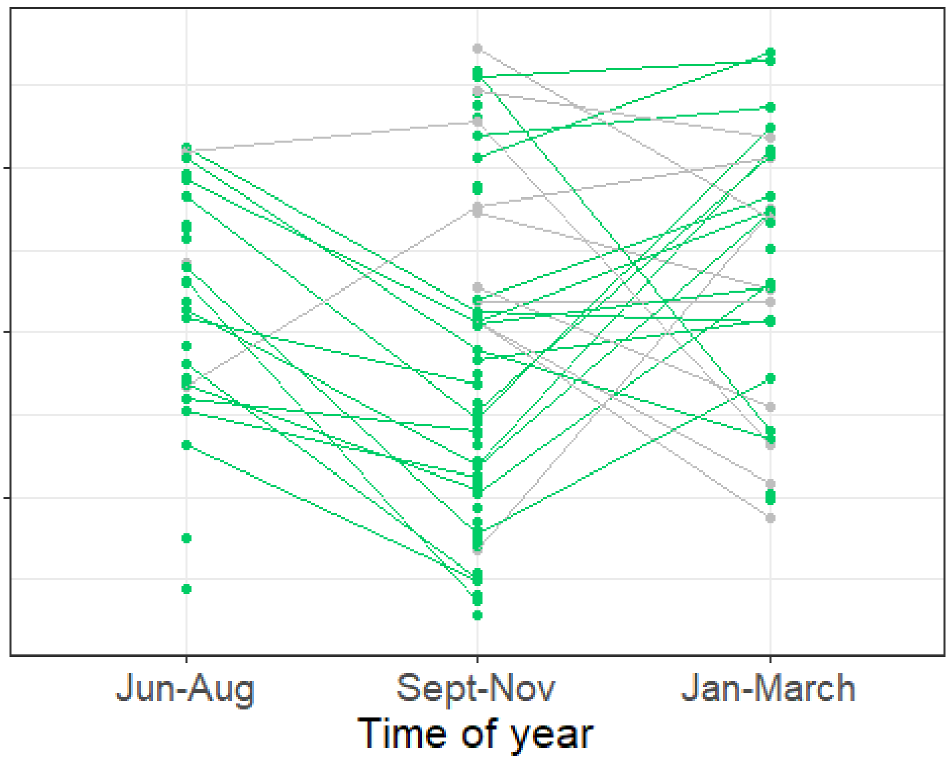


```
sil_physeq_met_recaps <- as.data.frame(sil_physeq_met_recaps)
#saveRDS(sil_physeq_met_recaps,
'~/Silwood_season_PC1_individual_shifts_random_sampling_per_month_JOF_plot.rd
s')

#test within-individual changes -paired 2-sample t test
colnames(sil_physeq_met_recaps) #43,51,60

##  [1] "Sequence_sample_code" "X"                    "ID"
##  [4] "trapdate"             "date"                 "trap"
##  [7] "pcr_plate"            "success"              "Species.x"
## [10] "Sex"                  "Body_mass_grams"      "Age"
```

```
## [13] "Reprod"                "Ectoparasites_seen"   "Body_condition"
## [16] "AGD"                   "month"                "year"
## [19] "months"                "season"               "degree"
## [22] "evc"                   "betweenness"          "habitat.common"
## [25] "grid.x_mean"           "grid.y_mean"          "Mouse.ID"
## [28] "Species.y"             "Diet_Month"           "Extraction_batch"
## [31] "Sequencing_round"      "Miseq_run"            "System"
## [34] "readDepth"             "Axis.1"               "Axis.2"
## [37] "Axis.3"                "Axis.4"               "Axis.5"
## [40] "Axis.6"                "JaccardPC1"           "Day_of_year"
## [43] "Month_group"           "JOF_trend"
```

```
physeq_meta_test1 <- sil_physeq_met_recaps[,c(3,35,43)]
head(physeq_meta_test1)
```

```
##        ID      Axis.1 Month_group
## 1   AF002  0.25398153   Jan_March
## 2 AF5672  0.24410912   Jan_March
## 3 AF5733 -0.07540429   Jan_March
## 4    M642 -0.42606444   Jan_March
## 5    M662 -0.04764568   Jan_March
## 6    M700 -0.01923483   Jan_March
```

```
summary(physeq_meta_test1)
```

```
##        ID                Axis.1            Month_group
##  Length:130         Min.   :-0.46850   Jan_March:32
##  Class :character   1st Qu.:-0.18712   Jun_Aug  :30
##  Mode  :character   Median :-0.01825   Sept_Nov :68
##                     Mean   :-0.00741
##                     3rd Qu.: 0.18985
##                     Max.   : 0.42972
```

```
physeq_meta_J_O_test <- subset(physeq_meta_test1, Month_group=="Jun_Aug"
|Month_group=="Sept_Nov" )
head(physeq_meta_J_O_test)
```

```
##        ID     Axis.1 Month_group
## 33 AF004  0.3114240     Jun_Aug
## 34 AF006  0.1183448     Jun_Aug
## 35  M792 -0.0336120     Jun_Aug
## 36  M803 -0.2793888     Jun_Aug
## 37  M842 -0.4684972     Jun_Aug
## 38  M845 -0.1056520     Jun_Aug
```

```
test.dat.mat <- acast(physeq_meta_J_O_test, ID ~ Month_group, value.var =
"Axis.1")
test.dat.mat <- as.data.frame(test.dat.mat)
test.dat.mat
```

```
##             Jun_Aug    Sept_Nov
## AF004   0.31142400          NA
```

```
## AF006   0.11834483  0.21944452
## AF007           NA  0.22022176
## AF010           NA  0.15506374
## AF014           NA  0.13716383
## AF4300          NA  0.22263194
## AF5278          NA  0.23133125
## AF5532          NA -0.18664390
## AF5733          NA  0.24428350
## AF5832          NA -0.39855327
## M642            NA -0.26666582
## M662            NA -0.04801093
## M684            NA -0.36501741
## M699            NA -0.21541093
## M700            NA  0.04337296
## M708            NA -0.32823931
## M756            NA  0.33254016
## M792   -0.03361200  0.20346781
## M802            NA -0.18288893
## M803   -0.27938877 -0.03077644
## M834            NA  0.32393636
## M838            NA  0.26645155
## M842   -0.46849721 -0.29880888
## M845   -0.10565200 -0.46673643
## M850            NA -0.04948624
## M854            NA  0.20504118
## M860   -0.09949140  0.30722593
## M864            NA -0.34480248
## M868   -0.20839784  0.13137255
## M870    0.08331207 -0.19016845
## M871            NA  0.17275806
## M873A  -0.23043292 -0.01546210
## M877            NA  0.12703645
## M878            NA -0.43134866
## M879            NA -0.06869415
## M882            NA -0.01630948
## M886            NA -0.39406157
## M889            NA -0.01727296
## M890            NA  0.11762246
## M891            NA -0.22296112
## M892            NA -0.01247962
## M899   -0.27543730 -0.32035489
## M900            NA -0.36631753
## M901            NA -0.38940721
## M902            NA -0.01004682
## M903            NA -0.03238961
## M904   -0.18907746          NA
## M905   -0.26509240  0.02810611
## M910   -0.23964161          NA
## M911    0.07027044          NA
## M912   -0.16534368          NA
```

```
## M913   -0.16132141          NA
## M914    0.16994197  0.37773767
## M916   -0.07717093  0.40732262
## M917   -0.14316270          NA
## M918    0.04908335  0.37560796
## M919    0.07794497  0.23828127
## M922   -0.15670625          NA
## M924    0.01935567          NA
## M925    0.38855517          NA
## M926   -0.07529408          NA
## M927   -0.07688169          NA
## M928   -0.04644206          NA
## M929            NA  0.21993392
## M930   -0.02372623  0.07865690
## M931    0.10002875  0.14868611
## M932            NA  0.21668544
## M934            NA -0.02375903
## M935            NA  0.31429330
## M936            NA  0.06353547
## M937            NA  0.37057237
## M938            NA  0.42972178
## M939            NA  0.28744989
## M940            NA  0.36444012
## M941            NA  0.19554542
## M942            NA  0.37176362
## M943            NA  0.22129163
## M944            NA  0.39970106
## M945            NA  0.13513247
## M947            NA  0.10768379
## M950            NA  0.30183686
```

```
nrow(subset(test.dat.mat, !is.na(Jun_Aug) & !is.na(Sept_Nov))) #n=16
```

```
## [1] 17
```

```
t.test(test.dat.mat$Jun_Aug, test.dat.mat$Sept_Nov, paired = T,
na.action=na.omit)
```

```
##
##  Paired t-test
##
## data:  test.dat.mat$Jun_Aug and test.dat.mat$Sept_Nov
## t = -2.9173, df = 16, p-value = 0.01007
## alternative hypothesis: true difference in means is not equal to 0
## 95 percent confidence interval:
##  -0.27035983 -0.04279845
## sample estimates:
## mean of the differences
##              -0.1565791
```

```
physeq_meta_OF_test <- subset(physeq_meta_test1, Month_group=="Jan_March"
|Month_group=="Sept_Nov" )
test.dat.mat <- acast(physeq_meta_OF_test, ID ~ Month_group, value.var =
"Axis.1")
test.dat.mat <- as.data.frame(test.dat.mat)
nrow(subset(test.dat.mat, !is.na(Jan_March) & !is.na(Sept_Nov)))#n=26

## [1] 27

t.test(test.dat.mat$Jan_March, test.dat.mat$Sept_Nov, paired = T,
na.action=na.omit)

##
##  Paired t-test
##
## data:  test.dat.mat$Jan_March and test.dat.mat$Sept_Nov
## t = -0.59034, df = 26, p-value = 0.5601
## alternative hypothesis: true difference in means is not equal to 0
## 95 percent confidence interval:
##  -0.14536843  0.08049972
## sample estimates:
## mean of the differences
##             -0.03243436
```

Combine population-level and individual-level plots for Wytham and Silwood

```
grid.arrange(p1,p3,p2,p4, ncol=2)

## Warning: Removed 2 rows containing missing values (geom_point).

## Warning: Removed 2 rows containing missing values (geom_point).

## Warning: Removed 2 rows containing missing values (geom_point).

## Warning: Removed 1 row(s) containing missing values (geom_path).
```

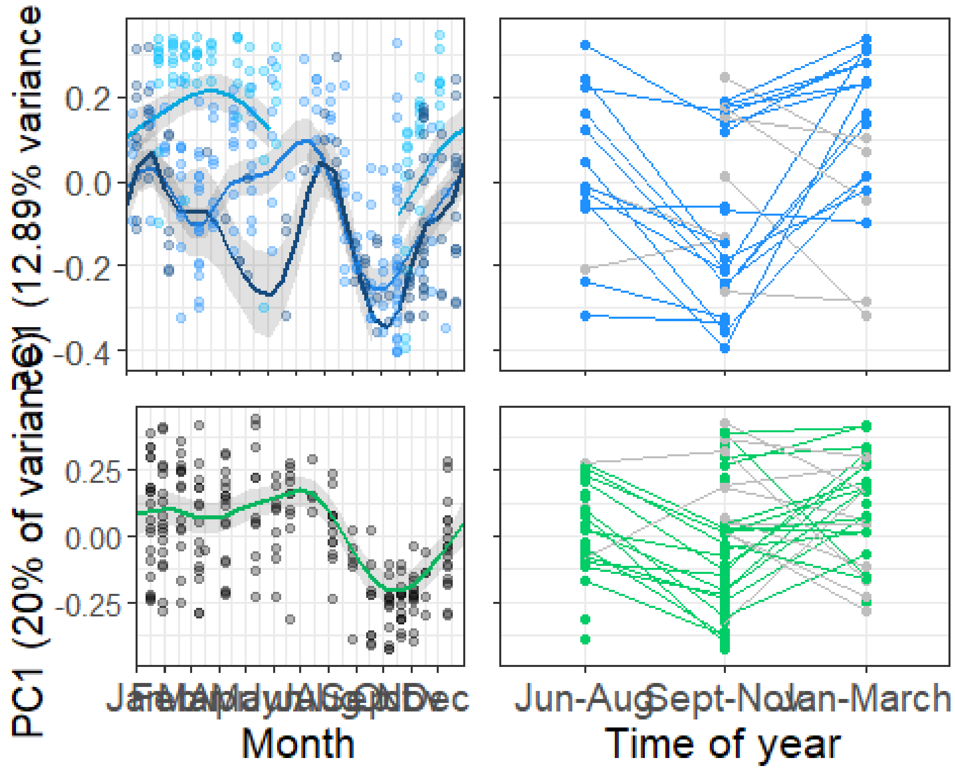

Stacked barplot of composition per month

```
ps.wyt.ra.FAMglom <- ps.wyt.ra %>%
  tax_glom(taxrank = "Family") %>%                     # agglomerate at
phylum level
  #transform_sample_counts(function(x) {x/sum(x)} ) %>% # Transform to rel.
abundance
  psmelt() %>%                                         # Melt to long format
  #filter(Abundance > 0.02) %>%                         # Filter out low
abundance taxa
  arrange(Family)
ps.wyt.ra.FAMglom$Population <- "Wytham"

ps.sil.ra.FAMglom <- ps.sil.ra %>%
  tax_glom(taxrank = "Family") %>%                     # agglomerate at
phylum level
  psmelt() %>%                                         # Melt to long format
  arrange(Family)
ps.sil.ra.FAMglom$Population <- "Silwood"
colnames(ps.sil.ra.FAMglom)

##  [1] "OTU"                  "Sample"               "Abundance"
##  [4] "Sequence_sample_code" "X"                    "ID"
##  [7] "trapdate"             "date"                 "trap"
## [10] "pcr_plate"            "success"              "Species.x"
## [13] "Sex"                  "Body_mass_grams"      "Age"
## [16] "Reprod"               "Ectoparasites_seen"   "Body_condition"
```

```
## [19] "AGD"                  "month"                "year"
## [22] "months"               "season"               "degree"
## [25] "evc"                  "betweenness"          "habitat.common"
## [28] "grid.x_mean"          "grid.y_mean"          "Mouse.ID"
## [31] "Species.y"            "Diet_Month"           "Extraction_batch"
## [34] "Sequencing_round"     "Miseq_run"            "System"
## [37] "readDepth"            "Axis.1"               "Axis.2"
## [40] "Axis.3"               "Axis.4"               "Axis.5"
## [43] "Axis.6"               "JaccardPC1"           "Kingdom"
## [46] "Phylum"               "Class"                "Order"
## [49] "Family"               "Population"
```

```
ps.sil.ra.FAMglom <- ps.sil.ra.FAMglom[,c(1:4,20,49,50)]
colnames(ps.sil.ra.FAMglom)[colnames(ps.sil.ra.FAMglom)=="month"] <- "Month"
colnames(ps.wyt.ra.FAMglom)
```

```
##  [1] "OTU"                               "Sample"
##  [3] "Abundance"                         "Sequence_sample_code"
##  [5] "X"                                 "Collection_date"
##  [7] "Grid"                              "XPIT_tag"
##  [9] "Animal_ID"                         "Trap_num"
## [11] "Grid_sq"                           "Species.x"
## [13] "Year"                              "Month"
## [15] "Zymo_FS_DNA_extraction_YN"         "Extraction_round"
## [17] "Extraction_batch.x"                "Extraction_sample_code"
## [19] "Poo_mass_mg_used_Zymo_extraction"  "Sequenced_YN"
## [21] "Sequencing_round.x"                "Miseq_run.x"
## [23] "SCFA_sample_YN"                    "Habitat_type"
## [25] "N_R"                               "Sex"
## [27] "Reprod"                            "Age"
## [29] "PIT_tag"                           "Body_mass_g"
## [31] "AGD_1"                             "AGD_2"
## [33] "AGD_3"                             "Foot_1"
## [35] "Foot_2"                            "Foot_3"
## [37] "Tick_L"                            "Tick_N"
## [39] "Tick_A"                            "Fleas"
## [41] "Mites"                             "Lept"
## [43] "Body_condition"                    "Peanuts_left"
## [45] "Apple_left"                        "X.1"
## [47] "X.2"                               "X.3"
## [49] "X.4"                               "X.5"
## [51] "Mouse.ID"                          "Species.y"
## [53] "Diet_Month"                        "Extraction_batch.y"
## [55] "Sequencing_round.y"                "Miseq_run.y"
## [57] "System"                            "readDepth"
## [59] "Axis.1"                            "Axis.2"
## [61] "Axis.3"                            "Axis.4"
## [63] "Axis.5"                            "Axis.6"
## [65] "JaccardPC1"                        "M_Y"
## [67] "Sampling_year"                     "Kingdom"
```

```
## [69] "Phylum"                              "Class"
## [71] "Order"                               "Family"
## [73] "Population"

ps.wyt.ra.FAMglom <- ps.wyt.ra.FAMglom[,c(1:4,14,72,73)]
ps.wyt.sil.FAMglom.comb <- rbind(ps.wyt.ra.FAMglom, ps.sil.ra.FAMglom)

all_tax_list <- as.list(unique(as.character(ps.wyt.sil.FAMglom.comb$Family)))
keep_tax_list <- c("Bacteroidales_S24-7_group", "Bifidobacteriaceae",
"Coriobacteriaceae",

"Deferribacteraceae","Desulfovibrionaceae","Enterococcaceae",
                   "Lachnospiraceae", "Lactobacillaceae",  "Prevotellaceae",
                   "Rikenellaceae", "Ruminococcaceae")

diff_list <-as.character(setdiff(all_tax_list, keep_tax_list))
ps.wyt.sil.FAMglom.comb$Family <- ps.wyt.sil.FAMglom.comb$Family %>%
fct_collapse(Other = diff_list)

colourCount = length(unique(ps.wyt.sil.FAMglom.comb$Family))
colourCount

## [1] 12

getPalette = colorRampPalette(brewer.pal(12, "Paired"))
level_order_month <- c("January", "February", "March", "April", "May",
"June",
                       "July", "August", "September", "October", "November",
"December")
level_order_fam <- c("Bacteroidales_S24-7_group", "Bifidobacteriaceae",
                     "Coriobacteriaceae","Deferribacteraceae",
"Desulfovibrionaceae",
                     "Enterococcaceae","Lachnospiraceae",
"Lactobacillaceae",
                     "Prevotellaceae", "Rikenellaceae", "Ruminococcaceae",
"Other")
ps.wyt.sil.FAMglom.comb$Family <- factor(ps.wyt.sil.FAMglom.comb$Family,
levels = level_order_fam)
ps.wyt.sil.FAMglom.comb$Population <-
factor(ps.wyt.sil.FAMglom.comb$Population, levels = c("Wytham", "Silwood"))

ggplot(ps.wyt.sil.FAMglom.comb, aes(x = factor(Month, levels =
level_order_month), y = Abundance, fill = Family)) +
  geom_bar(stat = "identity", position="fill", width = 1)  +
  facet_wrap(~Population)+
  ylab("Relative Abundance") + xlab("Month") +
  theme_bw() +
  theme(axis.text=element_text(size=12),
        axis.text.x = element_text(angle = 90),
        axis.title=element_text(size=14),
```

```
        strip.text = element_text(size=14),
        legend.text = element_text(size=12),
        legend.title = element_text(size=14))+
  scale_fill_manual(values = getPalette(colourCount)) +
  scale_x_discrete(label=abbreviate, drop=F)
```

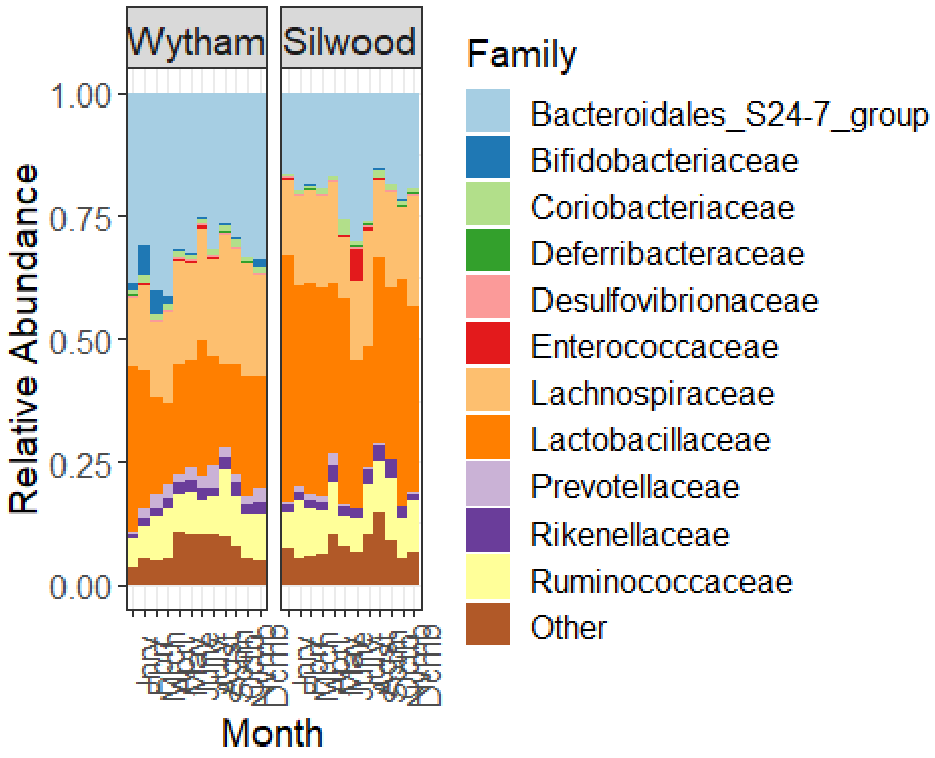

Random forest analyses of PC1

#Wytham

```
library(randomForest)

## randomForest 4.6-14

## Type rfNews() to see new features/changes/bug fixes.

##
## Attaching package: 'randomForest'

## The following object is masked from 'package:BiocGenerics':
##
##     combine

## The following object is masked from 'package:gridExtra':
##
##     combine
```

```
## The following object is masked from 'package:dplyr':
##
##     combine

## The following object is masked from 'package:ggplot2':
##
##     margin

predictors <- otu_table(ps.wyt.ra)
dim(predictors)

## [1]  421 1507

PC1 <- as.numeric(sample_data(ps.wyt.ra)$Axis.1)
data <- data.frame(PC1, predictors)

set.seed(321)
wyt_PC1_RFR <- randomForest(PC1 ~ ., data = data, ntree=10000, importance=T)
print(wyt_PC1_RFR)

##
## Call:
##  randomForest(formula = PC1 ~ ., data = data, ntree = 10000, importance =
T)
##                Type of random forest: regression
##                      Number of trees: 10000
## No. of variables tried at each split: 502
##
##           Mean of squared residuals: 0.003144631
##                     % Var explained: 92.83

## look at SV importance (mean decrease gini)
wyt_imp_RF <- importance(wyt_PC1_RFR)
wyt_imp_RF_dat <- data.frame(predictors = rownames(wyt_imp_RF), wyt_imp_RF)
wyt_imp_RF_sort <- arrange(wyt_imp_RF_dat, desc(IncNodePurity))
wyt_imp_RF_sort$predictors <- factor(wyt_imp_RF_sort$predictors, levels =
wyt_imp_RF_sort$predictors)
#saveRDS(imp_RF_sort, "~/Wyt_PC1_RandomForestRegression_ASV_importance.rds")

wyt_imp_RF_sub <- wyt_imp_RF_sort[1:30, ]

## coloured by family
tax_tab <- tax_table(ps.wyt.ra)
colnames(wyt_imp_RF_sub) <- c('ASV_name', '%.IncMSE', 'IncNodePurity')
tax_SV_fam <- tax_tab[,5]
tax_SV_fam <- as.data.frame(tax_SV_fam)
tax_SV_fam$ASV_name <- rownames(tax_SV_fam)
wyt_imp_plusfam <- merge(wyt_imp_RF_sub, tax_SV_fam, by='ASV_name', all.x =
T)
wyt_imp_plusfam_sort <- arrange(wyt_imp_plusfam, desc(IncNodePurity))
```

Plot relative abudnance of top 6 ASVs across seasons

```
top6_wyt <- as.list(as.character(wyt_imp_plusfam_sort$ASV_name[c(1:6)]))
ps.wyt.top6 <- subset_taxa(ps.wyt.ra, taxa_names(ps.wyt.ra)%in%top6_wyt)

sept_nov <- c("September", "October", "November")
other_months <- c("January", "February", "March", "April", "May", "June",
"July",
                  "August", "December")
sample_data(ps.wyt.top6)$Seasons <- "NA"
sample_data(ps.wyt.top6)[which(sample_data(ps.wyt.top6)$Month%in%sept_nov),]$
Seasons<-"Sept_Nov"
sample_data(ps.wyt.top6)[which(sample_data(ps.wyt.top6)$Month%in%other_months
),]$Seasons<-"Other_months"
summary(as.factor(sample_data(ps.wyt.top6)$Seasons))

## Other_months     Sept_Nov
##          269          152

ps.wyt.top6.melt <- psmelt(ps.wyt.top6)

#calculate mean and se of abundances per season per ASV
top6_season_sum <-  ddply(ps.wyt.top6.melt, c("OTU", "Seasons"), summarise,
                       N    = length(Abundance),
                       mean = mean(Abundance) ,
                       sd   = sd(Abundance),
                       se   = sd / sqrt(N))
top6_season_sum

##       OTU      Seasons   N         mean          sd           se
## 1    ASV1 Other_months 269 0.0567564724 0.115225285 0.0070254096
## 2    ASV1     Sept_Nov 152 0.0937970974 0.145499583 0.0118015745
## 3   ASV11 Other_months 269 0.0179618226 0.024805051 0.0015123907
## 4   ASV11     Sept_Nov 152 0.0067546007 0.015271725 0.0012387005
## 5  ASV117 Other_months 269 0.0036656829 0.005088275 0.0003102376
## 6  ASV117     Sept_Nov 152 0.0004203128 0.001464235 0.0001187651
## 7   ASV16 Other_months 269 0.0146096751 0.022159735 0.0013511029
## 8   ASV16     Sept_Nov 152 0.0054215434 0.013756730 0.0011158181
## 9    ASV2 Other_months 269 0.0623454540 0.101789699 0.0062062275
## 10   ASV2     Sept_Nov 152 0.0328716692 0.072457204 0.0058770553
## 11  ASV26 Other_months 269 0.0036947355 0.010800624 0.0006585257
## 12  ASV26     Sept_Nov 152 0.0159316194 0.021245310 0.0017232222

colnames(top6_season_sum)[colnames(top6_season_sum)=="mean"] <- "Abundance"
#add in families
top6_season_sum$Family <- ps.wyt.top6.melt$Family[match(top6_season_sum$OTU,
ps.wyt.top6.melt$OTU)]

#plot barplot showing mean and se per season per asv
p2 <- ggplot(top6_season_sum, aes(x=Seasons, y=Abundance)) +
  geom_bar(stat = "identity",colour="black",aes(fill=Family)) +
```

```
  geom_errorbar(data=top6_season_sum,
                aes(ymax=Abundance+se, ymin=Abundance),
                width=0.5) +
  facet_wrap(~OTU, scales = "free_y", nrow = 1) +
  theme_bw() +
  theme(axis.text.x = element_text(angle=45, hjust=1, size=10),
        axis.text.y = element_text(size=8), legend.position = "none") +
  ggtitle("Wytham")
p2#sort out colours in AffDes
```

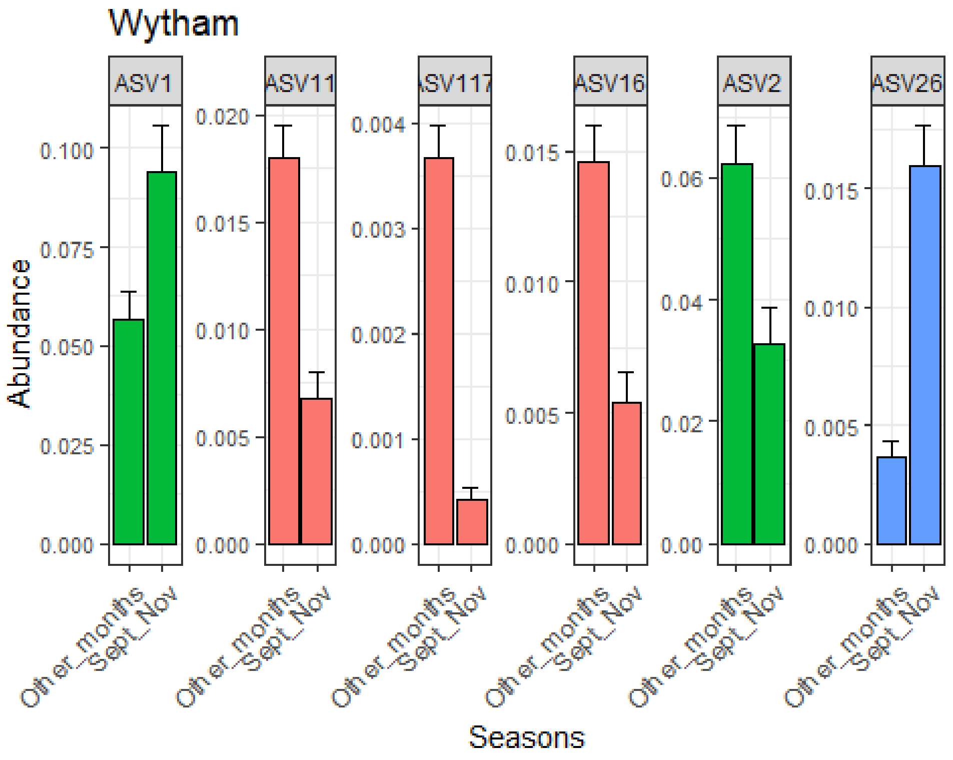

#Silwood

```
predictors <- otu_table(ps.sil.ra)
dim(predictors) #1045 asvs in 253 samples

## [1]  285 1130

PC1 <- as.numeric(sample_data(ps.sil.ra)$Axis.1) # Make one column for our
outcome/response variable
data <- data.frame(PC1, predictors) # Combine them into 1 data frame
set.seed(321)
sil_PC1_RFR <- randomForest(PC1 ~ ., data = data, ntree=10000, importance=T)
print(sil_PC1_RFR)

##
## Call:
##  randomForest(formula = PC1 ~ ., data = data, ntree = 10000, importance =
T)
##                Type of random forest: regression
```

```
##                      Number of trees: 10000
## No. of variables tried at each split: 376
##
##           Mean of squared residuals: 0.003019607
##                     % Var explained: 94.02

sil_imp_RF <- importance(sil_PC1_RFR)
sil_imp_RF_dat <- data.frame(predictors = rownames(sil_imp_RF), sil_imp_RF)
sil_imp_RF_sort <- arrange(sil_imp_RF_dat, desc(IncNodePurity))# Order the
predictor levels by importance
sil_imp_RF_sort$predictors <- factor(sil_imp_RF_sort$predictors, levels =
sil_imp_RF_sort$predictors)
#saveRDS(imp_RF_sort,
"~/Silwood_PC1_RandomForestRegression_ASV_importance.rds")

sil_imp_RF_sub <- sil_imp_RF_sort[1:30, ]

tax_tab <- tax_table(ps.sil.ra)
colnames(sil_imp_RF_sub) <- c('ASV_name', '%.IncMSE', 'IncNodePurity')
tax_SV_fam <- tax_tab[,5]
tax_SV_fam <- as.data.frame(tax_SV_fam)
tax_SV_fam$ASV_name <- rownames(tax_SV_fam)
sil_imp_plusfam <- merge(sil_imp_RF_sub, tax_SV_fam, by='ASV_name', all.x =
T)
sil_imp_plusfam_sort <- arrange(sil_imp_plusfam, desc(IncNodePurity))

#combine wytham and silwood RF results for plotting
sil_imp_plusfam_sort$System <- "Silwood"
colnames(sil_imp_plusfam_sort)

## [1] "ASV_name"      "%.IncMSE"      "IncNodePurity" "Family"
## [5] "System"

wyt_imp_plusfam_sort$System <- "Wytham"
colnames(wyt_imp_plusfam_sort)

## [1] "ASV_name"      "%.IncMSE"      "IncNodePurity" "Family"
## [5] "System"

imp_comb_dat <- rbind(wyt_imp_plusfam_sort, sil_imp_plusfam_sort)
imp_comb_dat$System <- factor(imp_comb_dat$System, levels = c("Wytham",
"Silwood"))

colourCount = length(unique(imp_comb_dat$Family))
getPalette = colorRampPalette(brewer.pal(10, "Paired"))

p1 <- ggplot(imp_comb_dat, aes(x = ASV_name, y = IncNodePurity, fill=Family))
+
  facet_wrap(~System, ncol=2, scales="free_x") +
  geom_bar(stat = "identity") +
```

```
  xlab("Amplicon sequence variant") +
  theme_bw() +
  theme(axis.text=element_text(size=14), axis.title=element_text(size=16),
        axis.text.x = element_blank(),
        strip.text = element_text(size=16),
        legend.text = element_text(size=14),
        legend.title = element_text(size=16)) +
  scale_fill_manual(values = getPalette(colourCount))
p1
```

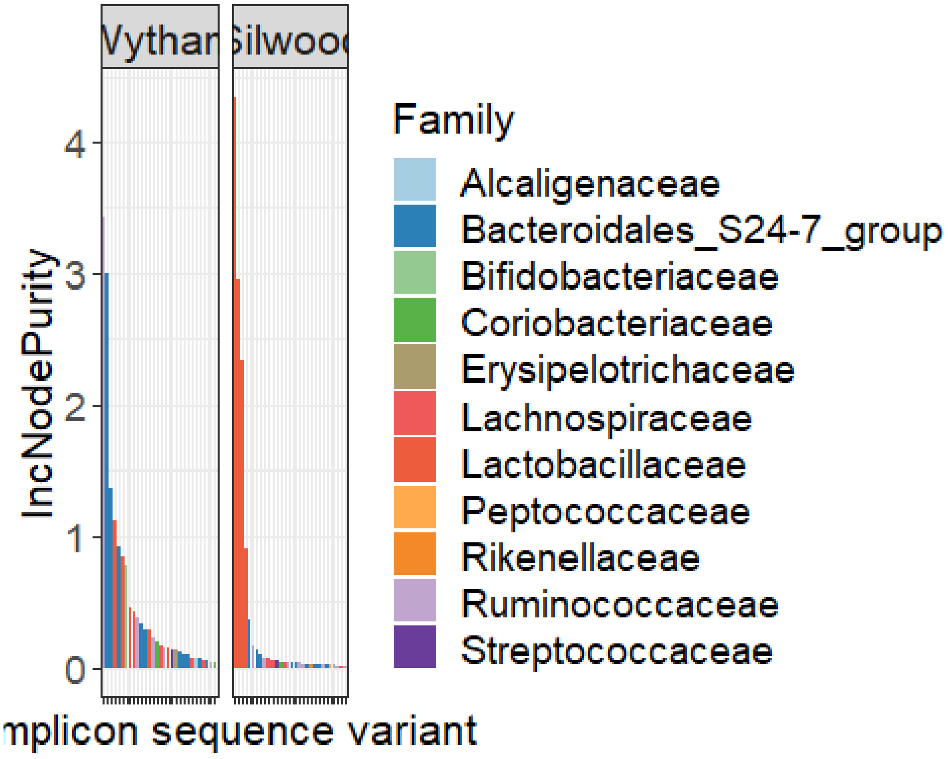

Relative abundance of top 6 Silwood ASVs across seasons

```
top6_sil <- as.list(as.character(sil_imp_plusfam_sort$ASV_name[c(1:6)]))
ps.sil.top6 <- subset_taxa(ps.sil.ra, taxa_names(ps.sil.ra)%in%top6_sil)

sept_nov <- c("September", "October", "November")
other_months <- c("January", "February", "March", "April", "May", "June",
"July",
                  "August", "December")
sample_data(ps.sil.top6)$Seasons <- "NA"
sample_data(ps.sil.top6)[which(sample_data(ps.sil.top6)$month%in%sept_nov),]$
Seasons<-"Sept_Nov"
sample_data(ps.sil.top6)[which(sample_data(ps.sil.top6)$month%in%other_months
),]$Seasons<-"Other_months"
summary(as.factor(sample_data(ps.sil.top6)$Seasons))

## Other_months     Sept_Nov
##          165          120
```

```r
ps.sil.top6.melt <- psmelt(ps.sil.top6)

#calculate mean and se of abundances per season per ASV
top6_season_sum_sil <-  ddply(ps.sil.top6.melt, c("OTU", "Seasons"),
summarise,
                          N    = length(Abundance),
                          mean = mean(Abundance) ,
                          sd   = sd(Abundance),
                          se   = sd / sqrt(N))
top6_season_sum_sil

##        OTU      Seasons   N        mean          sd           se
## 1  ASV5676 Other_months 165 0.169980684 0.147437982 0.0114780314
## 2  ASV5676     Sept_Nov 120 0.228685607 0.187516544 0.0171178402
## 3  ASV5677 Other_months 165 0.090690332 0.123797873 0.0096376514
## 4  ASV5677     Sept_Nov 120 0.069288301 0.115577085 0.0105506961
## 5  ASV5678 Other_months 165 0.078582747 0.078359182 0.0061002540
## 6  ASV5678     Sept_Nov 120 0.056292945 0.076854036 0.0070157815
## 7  ASV5679 Other_months 165 0.047867150 0.068507843 0.0053333283
## 8  ASV5679     Sept_Nov 120 0.026981528 0.066422643 0.0060635300
## 9  ASV5709 Other_months 165 0.001050673 0.005321106 0.0004142475
## 10 ASV5709     Sept_Nov 120 0.007193567 0.013540285 0.0012360533
## 11 ASV5751 Other_months 165 0.001996823 0.005150855 0.0004009935
## 12 ASV5751     Sept_Nov 120 0.001481639 0.003153678 0.0002878901

colnames(top6_season_sum_sil)[colnames(top6_season_sum_sil)=="mean"] <-
"Abundance"
top6_season_sum_sil$Family <-
ps.sil.top6.melt$Family[match(top6_season_sum_sil$OTU, ps.sil.top6.melt$OTU)]

#plot barplot showing mean and se per season per asv
p3 <- ggplot(top6_season_sum_sil, aes(x=Seasons, y=Abundance)) +
  geom_bar(stat = "identity",colour="black",aes(fill=Family)) +
  geom_errorbar(data=top6_season_sum_sil,
                aes(ymax=Abundance+se, ymin=Abundance),
                width=0.5) +
  facet_wrap(~OTU, scales = "free_y", nrow = 1) +
  theme_bw() +
  theme(axis.text.x = element_text(angle=45, hjust=1, size=10),
        axis.text.y = element_text(size=8), legend.position = "none") +
  ggtitle("Silwood")
p3#sort out colours in AffDes
```

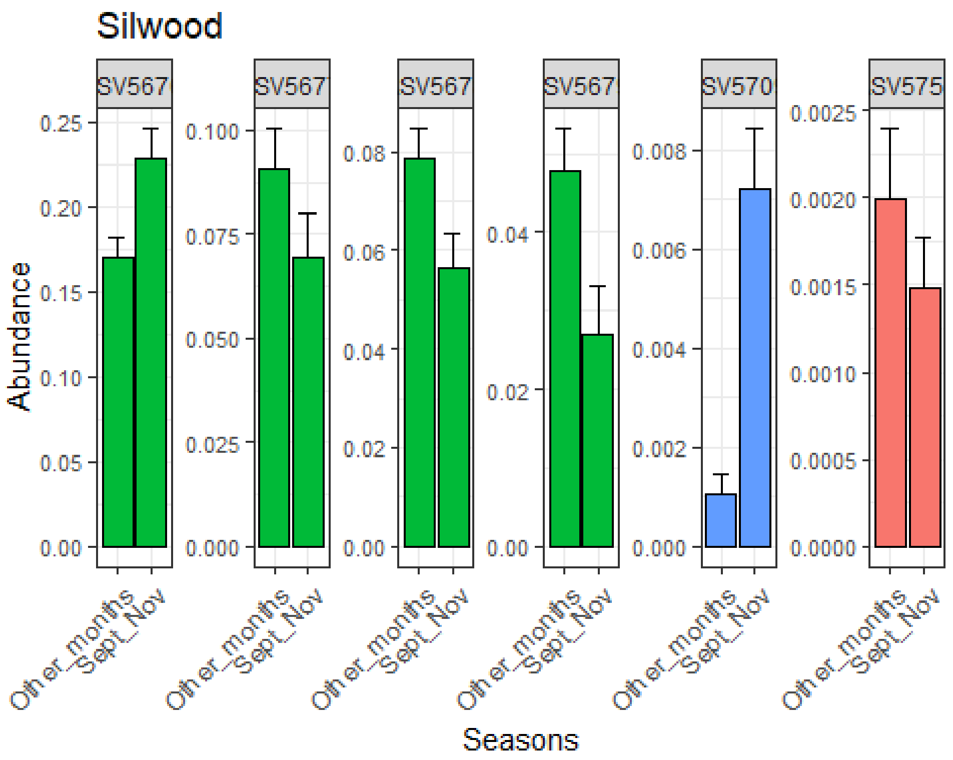


```
gridExtra::grid.arrange(p1,p2,p3, nrow=2)#sort out colours in AffDes
```

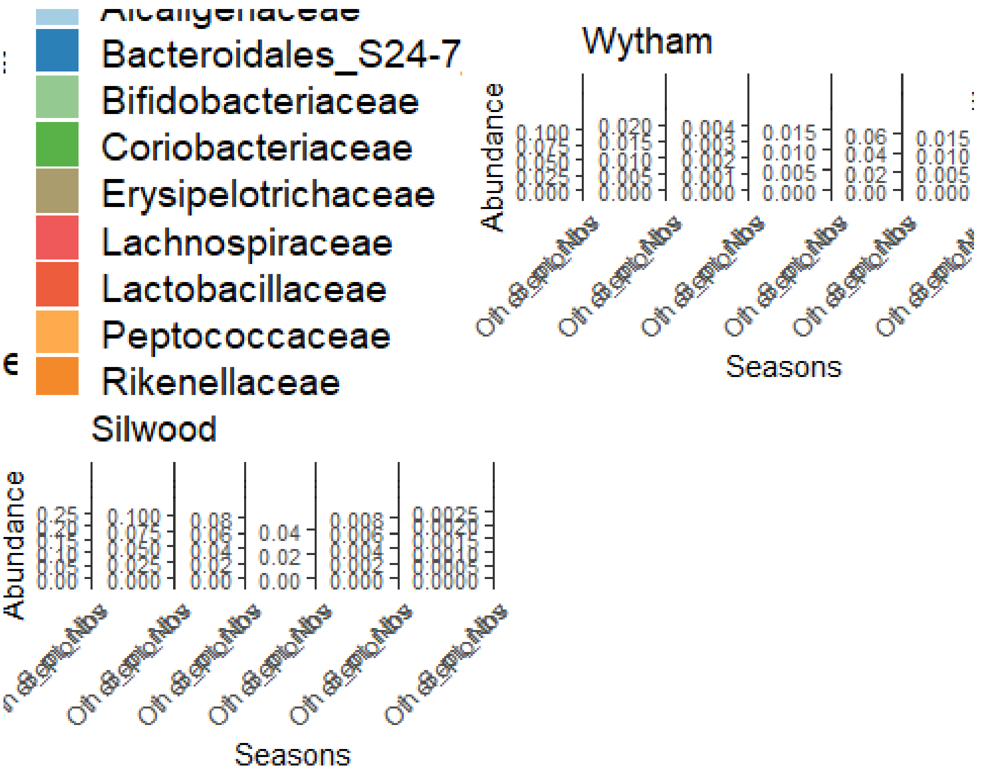

Seasonal changes in richness

#Wytham

```
library(breakaway)
library(mgcv)
#use phyloseq object that has taxon and sample-wise filtering but not ASV
prev/abund filtering
#ps.all.prune
#separate wytham and silwood data, and add in full metadat for each pop

#Wytham
ps.wyt.nf <- subset_samples(ps.all.prune, System=="Wytham" & Species=="AS")
ps.wyt.nf <- prune_taxa(taxa_sums(ps.wyt.nf) > 0, ps.wyt.nf)
M <- as(sample_data(ps.wyt.ra), 'data.frame')
rownames(M) <- M$Sequence_sample_code
wyt_physeq_alpha <- phyloseq(sample_data(M), otu_table(otu_table(ps.wyt.nf)),
tax_table(tax_table(ps.wyt.nf)))
#saveRDS(wyt_physeq_alpha,
'~/Wytham_phyloseq_WEWDSIL_combfilt_alphadiv_raw.rds')

#richness estimates using breakaway
ba <- breakaway(wyt_physeq_alpha)
#make a dataframe of estimates (same order of samples as in physeq object)
rich_dat <- as.data.frame(summary(ba))
rich_dat2 <- rich_dat[,c(1,2,5)]
colnames(rich_dat2) <- c("richness_estimate", "rich_est_error",
"Sequence_sample_code")

#add to main wytham data for modelling
wyt.main.dat <- as(sample_data(wyt_physeq_alpha), 'data.frame')
main.dat.rich <- merge(wyt.main.dat, rich_dat2, by="Sequence_sample_code")

#prep metadata for GAMM model
main.dat.rich <- subset(main.dat.rich, Grid=="Main")
main.dat.rich$Collection_date <- as.Date(main.dat.rich$Collection_date,
format= "%d/%m/%Y")
main.dat.rich$Day_of_year <- format(main.dat.rich$Collection_date, "%j")
main.dat.rich$Day_of_year <- as.numeric(main.dat.rich$Day_of_year)
M$XPIT_tag <- as.factor(M$XPIT_tag)
levels(main.dat.rich$XPIT_tag)[levels(main.dat.rich$XPIT_tag)==''] <- NA
main.dat.rich$Year <- as.factor(main.dat.rich$Year)
main.dat.rich <- subset(main.dat.rich, Grid=="Main" & !is.na(XPIT_tag))
main.dat.rich$Seq_run_comb <- paste(main.dat.rich$Sequencing_round.x,
main.dat.rich$Miseq_run.x, sep="_")
main.dat.rich$Seq_run_comb <- as.factor(main.dat.rich$Seq_run_comb)
main.dat.rich$Sex <- main.dat.rich$Sex %>% fct_collapse(F = c("F", "F?"),
M=c("M", "M?"))
levels(main.dat.rich$Sex)[levels(main.dat.rich$Sex)==''] <- NA
main.dat.rich$Age <- main.dat.rich$Age %>% fct_collapse(J = c("J", "J?"),
```

```
SA=c("SA", "SA?"))
levels(main.dat.rich$Age)[levels(main.dat.rich$Age)==''] <- NA
main.dat.rich$Reprod <- main.dat.rich$Reprod%>%
  fct_collapse(active = c("IMP_NIPP","LACT", "NIPP", "PERF", "Perf, plug",
                          "PREG", "PREG/LACT", "Preg?","PREG?", "Preg? Imp",
                          "TL", "TL+"))
main.dat.rich$Reprod <- main.dat.rich$Reprod%>%
  fct_collapse(inactive = c("TA", "TA?","TS", "IMP", "IMP?"))
levels(main.dat.rich$Reprod)[levels(main.dat.rich$Reprod)==''] <- NA
main.dat.rich$Body_condition <- as.factor(main.dat.rich$Body_condition)
#need to get rid of all NA's to get Rsq values
main.dat.rich_sub <- subset(main.dat.rich, !is.na(Age) & !is.na(Sex) &
!is.na(Reprod) &
                           !is.na(Body_mass_g) & !is.na(Body_condition))


#GAMM of seasonal richness
model1 <- gamm(richness_estimate ~ s(Day_of_year, bs = "cc", k = 24) +
                 Year + Sex + Reprod + Age + Body_mass_g + Body_condition +
Seq_run_comb,
               data = main.dat.rich_sub,
               random=list(XPIT_tag=~1),
               na.action = na.exclude)

summary(model1$gam)

##
## Family: gaussian
## Link function: identity
##
## Formula:
## richness_estimate ~ s(Day_of_year, bs = "cc", k = 24) + Year +
##     Sex + Reprod + Age + Body_mass_g + Body_condition + Seq_run_comb
##
## Parametric coefficients:
##                          Estimate Std. Error t value Pr(>|t|)
## (Intercept)              187.2780    31.0112   6.039 4.28e-09 ***
## Year2016                  22.5698    18.0595   1.250 0.212302
## Year2017                  72.1001    16.6360   4.334 1.96e-05 ***
## Year2018                  60.1997    20.9901   2.868 0.004404 **
## SexM                       0.8956     8.1355   0.110 0.912406
## Reprodactive               0.9142     9.5771   0.095 0.924011
## AgeJ                      12.0213    17.9184   0.671 0.502773
## AgeSA                      2.8981    12.5081   0.232 0.816921
## Body_mass_g                0.6150     1.2262   0.502 0.616354
## Body_condition1.5         -0.3718    12.5071  -0.030 0.976302
## Body_condition2            2.5509    12.9362   0.197 0.843803
## Body_condition2.5         -7.7773    16.6859  -0.466 0.641461
## Body_condition3          -26.7577    20.0204  -1.337 0.182326
## Seq_run_combExp_Wyt_2    -18.1952    10.5607  -1.723 0.085867 .
```

```
## Seq_run_combWyt_Diss_1 -49.9592    12.8133  -3.899 0.000118 ***
## Seq_run_combWyt_Diss_2 -15.6249    13.8280  -1.130 0.259343
## ---
## Signif. codes:  0 '***' 0.001 '**' 0.01 '*' 0.05 '.' 0.1 ' ' 1
##
## Approximate significance of smooth terms:
##                      edf Ref.df F p-value
## s(Day_of_year) 2.088e-06     22 0   0.561
##
## R-sq.(adj) =  0.0945
##   Scale est. = 4282.4    n = 337
```

```
anova(model1$gam)
```

```
##
## Family: gaussian
## Link function: identity
##
## Formula:
## richness_estimate ~ s(Day_of_year, bs = "cc", k = 24) + Year +
##     Sex + Reprod + Age + Body_mass_g + Body_condition + Seq_run_comb
##
## Parametric Terms:
##                 df      F  p-value
## Year             3 12.180 1.44e-07
## Sex              1  0.012  0.91241
## Reprod           1  0.009  0.92401
## Age              2  0.227  0.79727
## Body_mass_g      1  0.252  0.61635
## Body_condition   4  0.710  0.58553
## Seq_run_comb     3  5.534  0.00103
##
## Approximate significance of smooth terms:
##                      edf    Ref.df F p-value
## s(Day_of_year) 2.088e-06 2.200e+01 0   0.561
```

```
plot(model1$gam)
```

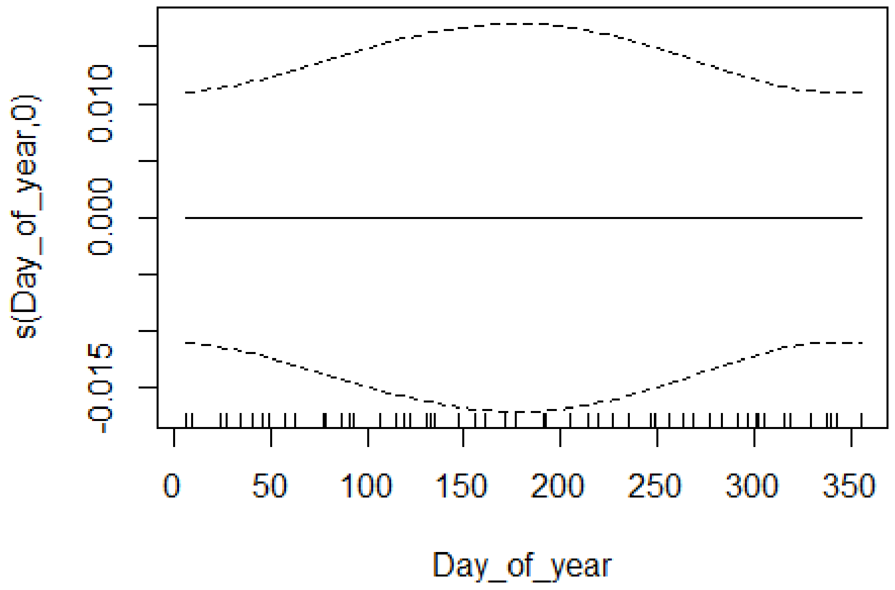

#Silwood

```
ps.sil.nf <- subset_samples(ps.all.prune, System=="Silwood")
ps.sil.nf <- prune_taxa(taxa_sums(ps.sil.nf) > 0, ps.sil.nf)
M <- as(sample_data(ps.sil.ra), 'data.frame')
rownames(M) <- M$Sequence_sample_code
ps.sil.alpha <- phyloseq(sample_data(M), otu_table(otu_table(ps.sil.nf)),
tax_table(tax_table(ps.sil.nf)))
#saveRDS(ps.sil.alpha,
'~/Silwood_phyloseq_WEWDSIL_combfilt_alphadiv_raw.rds')

#richness estimates using breakaway
ba <- breakaway(ps.sil.alpha)
#make a dataframe of estimates (same order of samples as in physeq object)
rich_dat <- as.data.frame(summary(ba))
rich_dat2 <- rich_dat[,c(1,2,5)]
colnames(rich_dat2) <- c("richness_estimate", "rich_est_error",
"Sequence_sample_code")

#add to main wytham data for modelling
sil.main.dat <- as(sample_data(ps.sil.alpha), 'data.frame')
main.dat.rich <- merge(sil.main.dat, rich_dat2, by="Sequence_sample_code")
main.dat.rich$date <- as.character(main.dat.rich$date)
main.dat.rich$date <- as.Date(main.dat.rich$date, format= "%d/%m/%Y") #now
collection date is recognised as in date format
main.dat.rich$Day_of_year <- format(main.dat.rich$date, "%j")#now get day of
the year
```

```r
main.dat.rich$Day_of_year <- as.numeric(main.dat.rich$Day_of_year)
main.dat.rich$Reprod <- main.dat.rich$Reprod%>%
  fct_collapse(active = c("NIPPLES", "PERF", "PREG", "TL", "TL+"),
               inactive = c("IMP", "TA", "TS"))
main.dat.rich$Body_condition <- as.factor(main.dat.rich$Body_condition)

physeq_met_sil2 <- subset(main.dat.rich, months!="Nov-14" &
                           !is.na(Body_condition) & !is.na(Body_mass_grams))

#GAMM of richness ~ DOY
model2 <- gamm(richness_estimate ~ s(Day_of_year, bs = "cc", k = 18) +
                 Sex + Reprod + Age + Body_mass_grams + Body_condition,
               data = physeq_met_sil2,
               random=list(ID=~1),
               na.action = na.exclude)

summary(model2$gam)

##
## Family: gaussian
## Link function: identity
##
## Formula:
## richness_estimate ~ s(Day_of_year, bs = "cc", k = 18) + Sex +
##     Reprod + Age + Body_mass_grams + Body_condition
##
## Parametric coefficients:
##                     Estimate Std. Error t value Pr(>|t|)
## (Intercept)          267.945     30.596   8.758 9.94e-16 ***
## SexM                  -2.794      8.940  -0.313   0.7549
## Reprodactive           9.533     13.863   0.688   0.4925
## AgeJ                  21.864     27.044   0.808   0.4198
## AgeSu                  2.385     13.420   0.178   0.8591
## Body_mass_grams       -1.007      1.405  -0.717   0.4743
## Body_condition1.5    -15.045     10.546  -1.427   0.1553
## Body_condition1.75   -60.984     36.214  -1.684   0.0938 .
## Body_condition2        6.609     13.892   0.476   0.6348
## Body_condition2.5      7.430     29.084   0.255   0.7986
## Body_condition3      -12.970     36.222  -0.358   0.7207
## ---
## Signif. codes:  0 '***' 0.001 '**' 0.01 '*' 0.05 '.' 0.1 ' ' 1
##
## Approximate significance of smooth terms:
##                   edf Ref.df    F  p-value
## s(Day_of_year) 4.477     16 1.29 0.000742 ***
## ---
## Signif. codes:  0 '***' 0.001 '**' 0.01 '*' 0.05 '.' 0.1 ' ' 1
##
## R-sq.(adj) =  0.118
##   Scale est. = 3460.7    n = 209
```

```
anova(model2$gam)

##
## Family: gaussian
## Link function: identity
##
## Formula:
## richness_estimate ~ s(Day_of_year, bs = "cc", k = 18) + Sex +
##     Reprod + Age + Body_mass_grams + Body_condition
##
## Parametric Terms:
##                 df     F p-value
## Sex              1 0.098   0.755
## Reprod           1 0.473   0.493
## Age              2 0.341   0.712
## Body_mass_grams  1 0.514   0.474
## Body_condition   5 1.275   0.276
##
## Approximate significance of smooth terms:
##                   edf Ref.df    F  p-value
## s(Day_of_year)  4.477 16.000 1.29 0.000742

plot(model2$gam)
```

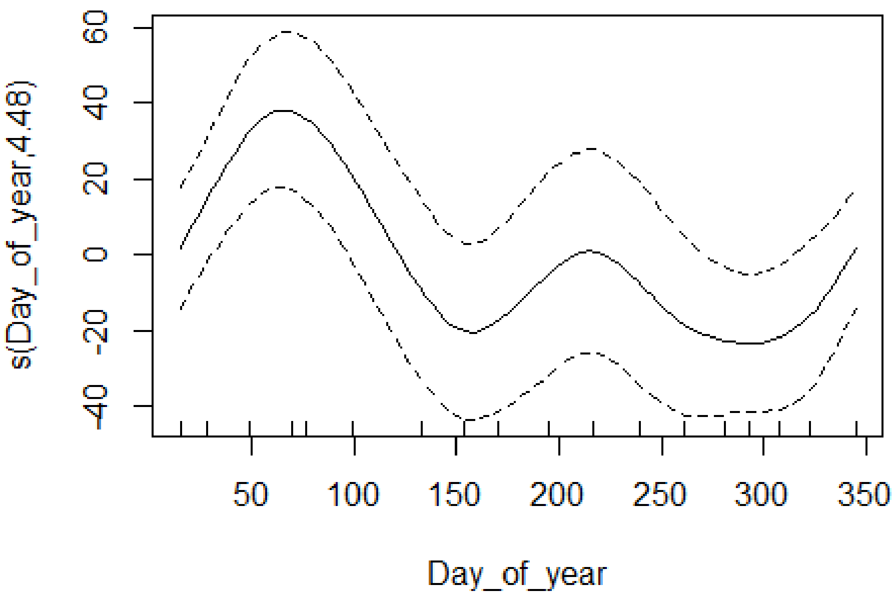


```
#check model assumptions
# sresid <- model2$gam$residuals
# hist(sresid)
```

```
# fitted.glmm <- model2$gam$fitted
# plot(sresid ~ fitted.glmm)
# plot(sresid ~ phseq_met_sil2$Day_of_year)

pdat <- expand.grid(Day_of_year=seq(0,365,14), Age="A", Sex="F",
Reprod="active",
                    Body_condition="2", Body_mass_grams=19.19)
pred <- predict (model2$gam, newdata = pdat, na.rm = T,
                 type= "response", se.fit = TRUE, level=0)
predframe <- data.frame (pdat, preds = pred$fit, se = pred$se.fit)

#plot fitted values with raw data
ggplot(physeq_met_sil2, aes(x = Day_of_year, y = richness_estimate)) +
  geom_point(alpha=0.3) +
  xlab("Month") + ylab("Microbial richness estimate") +
  #ggtitle("Silwood") +
  theme_bw() +
  geom_line(aes(y=preds), data=predframe, colour="springgreen3", size=1) +
  geom_ribbon(data = predframe, aes(y = NULL, ymin = preds-se, ymax =
preds+se),
              alpha=0.15) +
  theme(axis.text.x=element_text(size=12),axis.title=element_text(size=14),
        title = element_text(size=14))  +
  scale_x_continuous(breaks=cumsum(c(1,31,28,31,30,31,30,31,31,30,31,30,30)),
                     labels
=c("Jan","Feb","Mar","Apr","May","Jun","Jul","Aug",
                               "Sep","Oct","Nov","Dec", "Jan"),
                     expand = c(0,0))
```

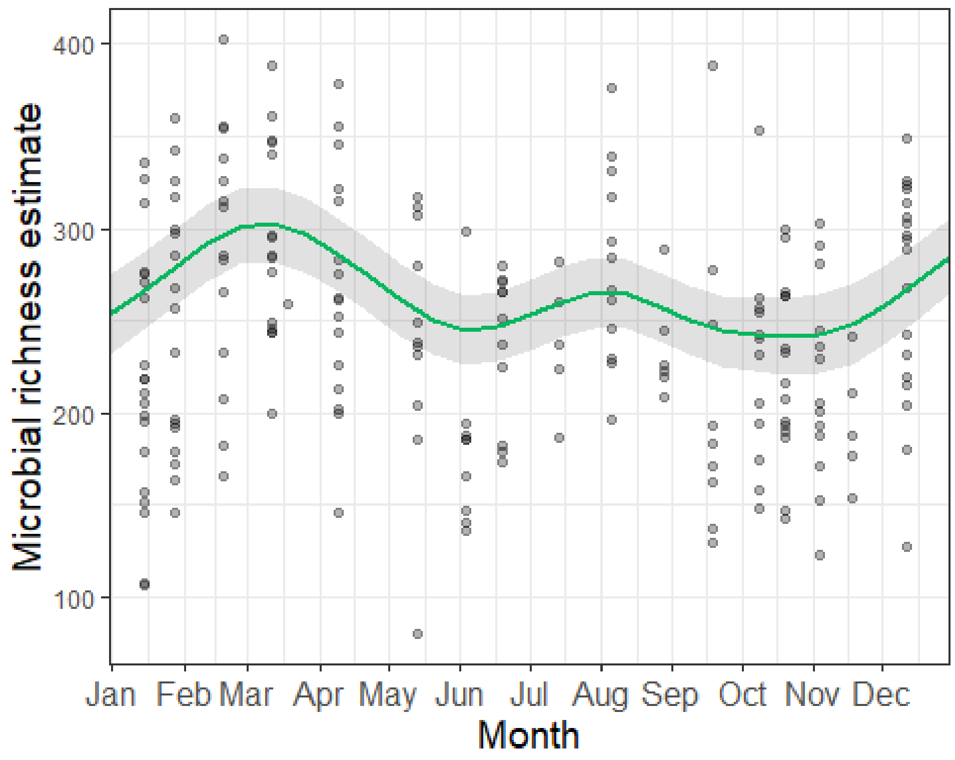

Section 7: Individuality and seasonal convergence

##Wytham

Prep data

```
library(phyloseq)
library(ggplot2)
library(plyr); library(dplyr)
library(reshape2)
library(forcats)
library(vegan)

ps.wyt.ra

## phyloseq-class experiment-level object
## otu_table()   OTU Table:         [ 1507 taxa and 421 samples ]
## sample_data() Sample Data:       [ 421 samples by 64 sample variables ]
## tax_table()   Taxonomy Table:    [ 1507 taxa by 7 taxonomic ranks ]

M <- as(sample_data(ps.wyt.ra), 'data.frame')
summary(M$Collection_date) #format is dd/mm/yyyy, currently a factor

##    Length     Class      Mode
##       421 character character

M$Collection_date <- as.Date(M$Collection_date, format= "%d/%m/%Y")
M$Time_cont <- as.numeric(M$Collection_date)
```

```
M$Day_of_year <- format(M$Collection_date, "%j")#now get day of the year
M$Day_of_year <- as.numeric(M$Day_of_year)
M$M_Y<-paste(M$Month,M$Year,sep="/")
M$XPIT_tag <- as.factor(M$XPIT_tag)
levels(M$XPIT_tag)[levels(M$XPIT_tag)==''] <- NA
length(levels(M$XPIT_tag))
```

```
## [1] 160
```

```
M <- subset(M, !is.na(XPIT_tag))
ps.wyt.ra.known.ind <- phyloseq(otu_table(otu_table(ps.wyt.ra)),
                                tax_table(tax_table(ps.wyt.ra)),
sample_data(M))

#dissimilarity matrix (Bray-curtis)
D<- phyloseq::distance(ps.wyt.ra.known.ind, method = "bray", type =
"samples")
D <- as.matrix(D)

#time difference matrix
#now make matrix of pairwise time differences using Time_0
Time <- as.matrix(dist(sample_data(ps.wyt.ra.known.ind)$Time_cont),
labels=TRUE, dimnames=list(rownames(sample_data(ps.wyt.ra.known.ind)),
rownames(sample_data(ps.wyt.ra.known.ind))))
colnames(Time) <- rownames(Time) <-
rownames(sample_data(ps.wyt.ra.known.ind))

#Now melt to long format and combine D and Time for each pair of sampels
Time_melt <- melt(Time)
D_melt <- melt(D)
Time_melt$Sample_pair <- paste(Time_melt$Var1, Time_melt$Var2, sep="-")
D_melt$Sample_pair <- paste(D_melt$Var1, D_melt$Var2, sep="-")

#want to make 2 new columns with corresponding pit tags to each sample
colnames(D_melt)[1] <- "Sequence_sample_code"
D_melt$PIT.A <- "NA"
D_melt$PIT.A = M[match(D_melt$Sequence_sample_code,
M$Sequence_sample_code),"XPIT_tag"]
D_melt$Time0.A <- "NA"
D_melt$Time0.A = M[match(D_melt$Sequence_sample_code,
M$Sequence_sample_code),"Time_cont"]
D_melt$Collection_date.A <- "NA"
D_melt$Collection_date.A = M[match(D_melt$Sequence_sample_code,
M$Sequence_sample_code),"Collection_date"]
D_melt$DOY.A <- "NA"
D_melt$DOY.A = M[match(D_melt$Sequence_sample_code,
M$Sequence_sample_code),"Day_of_year"]
D_melt$Month.A <- "NA"
D_melt$Month.A = M[match(D_melt$Sequence_sample_code,
M$Sequence_sample_code),"Month"]
```

```
D_melt$Year.A <- "NA"
D_melt$Year.A = M[match(D_melt$Sequence_sample_code,
M$Sequence_sample_code),"Sampling_year"]
D_melt$M_Y.A <- "NA"
D_melt$M_Y.A = M[match(D_melt$Sequence_sample_code,
M$Sequence_sample_code),"M_Y"]
colnames(D_melt)[1] <- "Var1"
colnames(D_melt)[2] <- "Sequence_sample_code"
D_melt$PIT.B <- "NA"
D_melt$PIT.B = M[match(D_melt$Sequence_sample_code,
M$Sequence_sample_code),"XPIT_tag"]
D_melt$Time0.B <- "NA"
D_melt$Time0.B = M[match(D_melt$Sequence_sample_code,
M$Sequence_sample_code),"Time_cont"]
D_melt$Collection_date.B <- "NA"
D_melt$Collection_date.B = M[match(D_melt$Sequence_sample_code,
M$Sequence_sample_code),"Collection_date"]
D_melt$DOY.B <- "NA"
D_melt$DOY.B = M[match(D_melt$Sequence_sample_code,
M$Sequence_sample_code),"Day_of_year"]
D_melt$Month.B <- "NA"
D_melt$Month.B = M[match(D_melt$Sequence_sample_code,
M$Sequence_sample_code),"Month"]
D_melt$Year.B <- "NA"
D_melt$Year.B = M[match(D_melt$Sequence_sample_code,
M$Sequence_sample_code),"Sampling_year"]
D_melt$M_Y.B <- "NA"
D_melt$M_Y.B = M[match(D_melt$Sequence_sample_code,
M$Sequence_sample_code),"M_Y"]
colnames(D_melt)[2] <- "Var2"

Distance_Time_merge <- merge(Time_melt, D_melt, by="Sample_pair")
head(Distance_Time_merge)

##   Sample_pair Var1.x Var2.x value.x Var1.y Var2.y   value.y       PIT.A
Time0.A
## 1 WD100-WD100  WD100  WD100       0  WD100  WD100 0.0000000 X0418EBC464
17457
## 2 WD100-WD101  WD100  WD101       0  WD100  WD101 0.6686529 X0418EBC464
17457
## 3 WD100-WD102  WD100  WD102       0  WD100  WD102 0.6401957 X0418EBC464
17457
## 4 WD100-WD103  WD100  WD103       0  WD100  WD103 0.7322196 X0418EBC464
17457
## 5 WD100-WD104  WD100  WD104       0  WD100  WD104 0.6308379 X0418EBC464
17457
## 6 WD100-WD105  WD100  WD105      14  WD100  WD105 0.7638095 X0418EBC464
17457
##   Collection_date.A DOY.A Month.A    Year.A        M_Y.A       PIT.B
Time0.B
```

```
## 1        2017-10-18   291 October Year16_17 October/2017 X0418EBC464
17457
## 2        2017-10-18   291 October Year16_17 October/2017 X0418EBB77E
17457
## 3        2017-10-18   291 October Year16_17 October/2017 X0418EB9FC7
17457
## 4        2017-10-18   291 October Year16_17 October/2017 X0418EB834B
17457
## 5        2017-10-18   291 October Year16_17 October/2017 X0418EBBF1E
17457
## 6        2017-10-18   291 October Year16_17 October/2017 X041918134D
17471
##   Collection_date.B DOY.B  Month.B    Year.B         M_Y.B
## 1        2017-10-18   291  October Year16_17  October/2017
## 2        2017-10-18   291  October Year16_17  October/2017
## 3        2017-10-18   291  October Year16_17  October/2017
## 4        2017-10-18   291  October Year16_17  October/2017
## 5        2017-10-18   291  October Year16_17  October/2017
## 6        2017-11-01   305 November Year17_18 November/2017

Distance_Time_merge <-
Distance_Time_merge[Distance_Time_merge$Var1.x!=Distance_Time_merge$Var2.x, ]

#save BC distance data
#write.csv(Distance_Time_merge, '~/Wytham_season_BC_time_dist_full_data.csv')
```

Within-individual change at different time lags

```
# now look at within-individual similarity at different time lags
Dist_time_withind <-
Distance_Time_merge[Distance_Time_merge$PIT.A==Distance_Time_merge$PIT.B, ]
Dist_time_withind_2 <- subset(Dist_time_withind, value.x!=0)
#Dist_time_withind_0 <- subset(Dist_time_withind, value.x==0)
#head(Dist_time_withind_0)
#head(Dist_time_withind_2)

#now keep only mice caught 3 or more times
M <- as(sample_data(ps.wyt.ra), 'data.frame')
M$XPIT_tag <- as.factor(M$XPIT_tag)
levels(M$XPIT_tag)[levels(M$XPIT_tag)==''] <- NA
#length(levels(M$XPIT_tag))
M <- as.data.frame(M)
M <- subset(M, !(is.na(XPIT_tag)))

mouse_recaps <- as.data.frame(summary(as.factor(M$XPIT_tag), maxsum = 300))
mouse_recaps_multi <- subset(mouse_recaps, summary(as.factor(M$XPIT_tag),
maxsum = 300)>=3)
mouse_recap_list <- as.list(rownames(mouse_recaps_multi))
#mouse_recap_list
```

```r
Dist_time_withind_3 <- subset(Dist_time_withind_2, PIT.A%in%mouse_recap_list)

p1 <- ggplot(Dist_time_withind_3, aes(x=value.x, y=value.y)) +
  geom_point() +
  #geom_line(aes(group=PIT.A)) + looks messy with individual lines
  stat_smooth(method = "loess", colour="dodgerblue") +
  ylab("Bray-Curtis dissimilarity") +
  xlab("Number of days between sampling") +
  theme_bw() +
  theme(axis.text.x=element_text(angle=60, hjust=1,
size=11),axis.title=element_text(size=14),
        title = element_text(size=14)) +
  ggtitle("Wytham")
p1

## `geom_smooth()` using formula 'y ~ x'
```

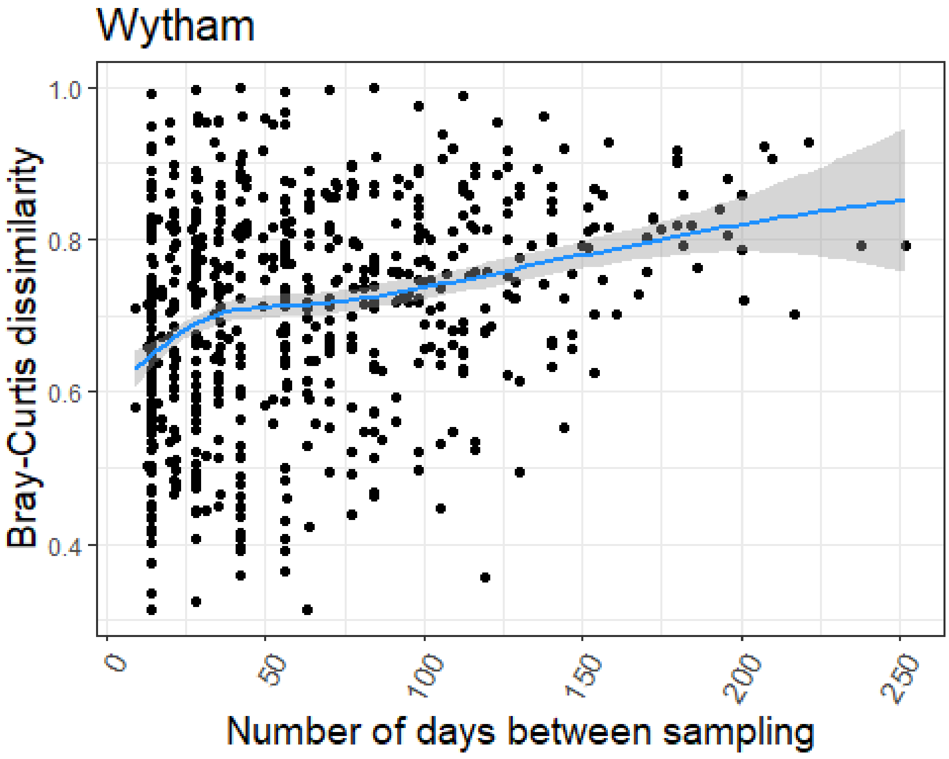


```r
### log-linear analysis
Dist_time_withind_3$BC_sim = 1- Dist_time_withind_3$value.y#convert BC to
similarity by 1-BC

mdl.1 <- lm(log(BC_sim+1) ~ value.x, data = Dist_time_withind_3)
summary(mdl.1)

##
## Call:
## lm(formula = log(BC_sim + 1) ~ value.x, data = Dist_time_withind_3)
```

```
##
## Residuals:
##       Min        1Q    Median        3Q       Max
## -0.270890 -0.075062 -0.004027  0.073090  0.285149
##
## Coefficients:
##               Estimate Std. Error t value Pr(>|t|)
## (Intercept)  2.893e-01  4.941e-03   58.55   <2e-16 ***
## value.x     -6.510e-04  6.283e-05  -10.36   <2e-16 ***
## ---
## Signif. codes:  0 '***' 0.001 '**' 0.01 '*' 0.05 '.' 0.1 ' ' 1
##
## Residual standard error: 0.105 on 1268 degrees of freedom
## Multiple R-squared:  0.07804,    Adjusted R-squared:  0.07732
## F-statistic: 107.3 on 1 and 1268 DF,  p-value: < 2.2e-16

# plot(mdl.1)
# AIC(mdl.1)

#use it for prediction
summary(Dist_time_withind_3$value.x)#predict between 9-252 days

##    Min. 1st Qu.  Median    Mean 3rd Qu.    Max.
##    9.00   28.00   52.00   63.13   92.00  252.00

pdat <- expand.grid(value.x=seq(9,252,7))
pdat[36,] <- 252

pred <- predict (mdl.1, newdata = pdat, na.rm = T,
                 type= "response", se.fit = TRUE, level=0,
                 interval = "prediction")
predframe <- data.frame (pdat, preds = pred$fit, se = pred$se.fit)
Dist_time_withind_wyt <- Dist_time_withind_3
predframe_wyt <- predframe

#plot fitted values with raw data
p1 <- ggplot(Dist_time_withind_wyt, aes(x=value.x, y=log(BC_sim+1))) +
  geom_point(alpha=0.3) +
  #geom_line(aes(group=PIT.A)) + looks messy with individual lines
  #stat_smooth(method = "loess", colour="dodgerblue", se=F) +
  geom_line(aes(y=preds.fit), data=predframe_wyt, colour="dodgerblue",
size=1) +
  geom_ribbon(data = predframe_wyt, aes(y = NULL, ymin = preds.fit-(1.96*se),
                                        ymax = preds.fit+(1.96*se)),
alpha=0.15) +
  ylab("log((1- Bray-Curtis dissimilarity) +1)") +
  xlab("Number of days between sampling") +
  theme_bw() +
  theme(axis.text.x=element_text(size=11),axis.title=element_text(size=14),
        title = element_text(size=14)) +
```

```
  ggtitle("Wytham")
p1
```

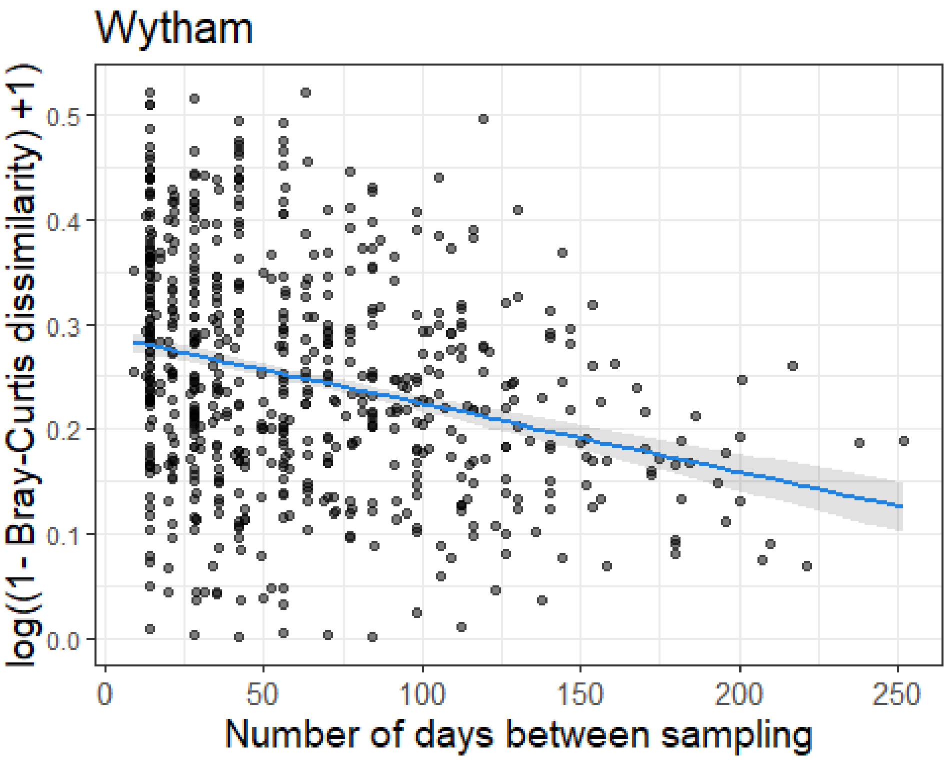


```
#see how the rate of change over time changes voer different timescales
Dist_time_withind_3$rate_change = Dist_time_withind_3$BC_sim /
Dist_time_withind_3$value.x
summary(Dist_time_withind_3$value.x) #min=9 max=252

##    Min. 1st Qu.  Median    Mean 3rd Qu.    Max.
##    9.00   28.00   52.00   63.13   92.00  252.00

#look at average rate of change within individuals over different timescales;
#<30 , 30<x>90, 90<x>180, 180<x>365

#within 1 month
summary(subset(Dist_time_withind_3, value.x<30)$rate_change)#mean=0.019029

##      Min.   1st Qu.    Median      Mean   3rd Qu.      Max.
## 0.0001479 0.0101183 0.0179961 0.0190400 0.0254549 0.0489356

sd(subset(Dist_time_withind_3, value.x<30)$rate_change)#sd=0.0108052

## [1] 0.01081321

length(subset(Dist_time_withind_3, value.x<30)$rate_change) #n=418 pairwise
comaprisions

## [1] 418
```

```r
#between 1 and 3 months
summary(subset(Dist_time_withind_3, value.x>=30 &
value.x<90)$rate_change)#mean=5.685e-03 (0.005685)

##      Min.   1st Qu.    Median      Mean   3rd Qu.      Max.
## 5.433e-06 3.074e-03 4.694e-03 5.684e-03 7.450e-03 1.793e-02

sd(subset(Dist_time_withind_3, value.x>=30 &
value.x<90)$rate_change)#sd=0.003528637

## [1] 0.003529764

length(subset(Dist_time_withind_3, value.x>=30 &
value.x<90)$rate_change)#n=520

## [1] 520

#between 3 and 6 months
summary(subset(Dist_time_withind_3, value.x>=90 &
value.x<180)$rate_change)#mean=0.0021112

##      Min.   1st Qu.    Median      Mean   3rd Qu.      Max.
## 9.069e-05 1.238e-03 1.977e-03 2.113e-03 2.837e-03 5.410e-03

sd(subset(Dist_time_withind_3, value.x>=90 &
value.x<180)$rate_change)#sd=0.001080227

## [1] 0.001081139

length(subset(Dist_time_withind_3, value.x>=90 &
value.x<180)$rate_change)#n=292

## [1] 292

#between 6 months - 1 year
summary(subset(Dist_time_withind_3, value.x>=180 &
value.x<365)$rate_change)#mean=0.0008271

##      Min.   1st Qu.    Median      Mean   3rd Qu.      Max.
## 0.0003271 0.0005454 0.0008266 0.0008261 0.0010175 0.0013936

sd(subset(Dist_time_withind_3, value.x>=180 &
value.x<365)$rate_change)#sd=0.0003225315

## [1] 0.0003218978

length(subset(Dist_time_withind_3, value.x>=180 &
value.x<365)$rate_change)#n=40

## [1] 40
```

Between-individual variation per month

```r
#are individuals more similar at certain times of year?
Dist_time_same_time <-
Distance_Time_merge[Distance_Time_merge$M_Y.A==Distance_Time_merge$M_Y.B, ]
Dist_time_same_time <-
Dist_time_same_time[Dist_time_same_time$PIT.A!=Dist_time_same_time$PIT.B, ]

level_order <- c("January", "February", "March", "April", "May", "June",
"July", "August", "September", "October", "November", "December")

#plot BC dist per per trap date
names(Distance_Time_merge)

##  [1] "Sample_pair"       "Var1.x"            "Var2.x"
##  [4] "value.x"           "Var1.y"            "Var2.y"
##  [7] "value.y"           "PIT.A"             "Time0.A"
## [10] "Collection_date.A" "DOY.A"             "Month.A"
## [13] "Year.A"            "M_Y.A"             "PIT.B"
## [16] "Time0.B"           "Collection_date.B" "DOY.B"
## [19] "Month.B"           "Year.B"            "M_Y.B"

Dist_time_same_time <-
Distance_Time_merge[Distance_Time_merge$Collection_date.A==Distance_Time_merg
e$Collection_date.B, ]
Dist_time_same_time <-
Dist_time_same_time[Dist_time_same_time$PIT.A!=Dist_time_same_time$PIT.B, ]

# add average within-individual BC as a dashed reference line
# mean(Dist_time_withind_2$value.y)
# sd(Dist_time_withind_2$value.y)
se = sd(Dist_time_withind_2$value.y)/ sqrt(nrow(Dist_time_withind_2))

p2b <- ggplot(Dist_time_same_time, aes(x=DOY.A, y=value.y)) +
  geom_point(alpha=0.3) +
  geom_smooth(method="gam", formula = y ~ s(x, bs = "cc"),
colour="dodgerblue") +
  geom_hline(yintercept = 0.7102018, colour="red", size=1, linetype=2) +
  geom_ribbon(aes(ymin=0.7102018-se, ymax=0.7102018+se), alpha=0.2) +
  ggtitle("Wytham") +
  ylab("Bray-Curtis dissimilarity") +
  xlab("Month") +
  theme_bw() +
  theme(axis.text.x=element_text( size=11),axis.title=element_text(size=14),
        title = element_text(size=14)) +
  scale_x_continuous(breaks=cumsum(c(1,31,28,31,30,31,30,31,31,30,31,30)),
                     labels =c("Jan","Feb","Mar","Apr","May","Jun","Jul",
                               "Aug","Sep","Oct","Nov","Dec"),
                     expand = c(0.015,0.015))
p2b
```

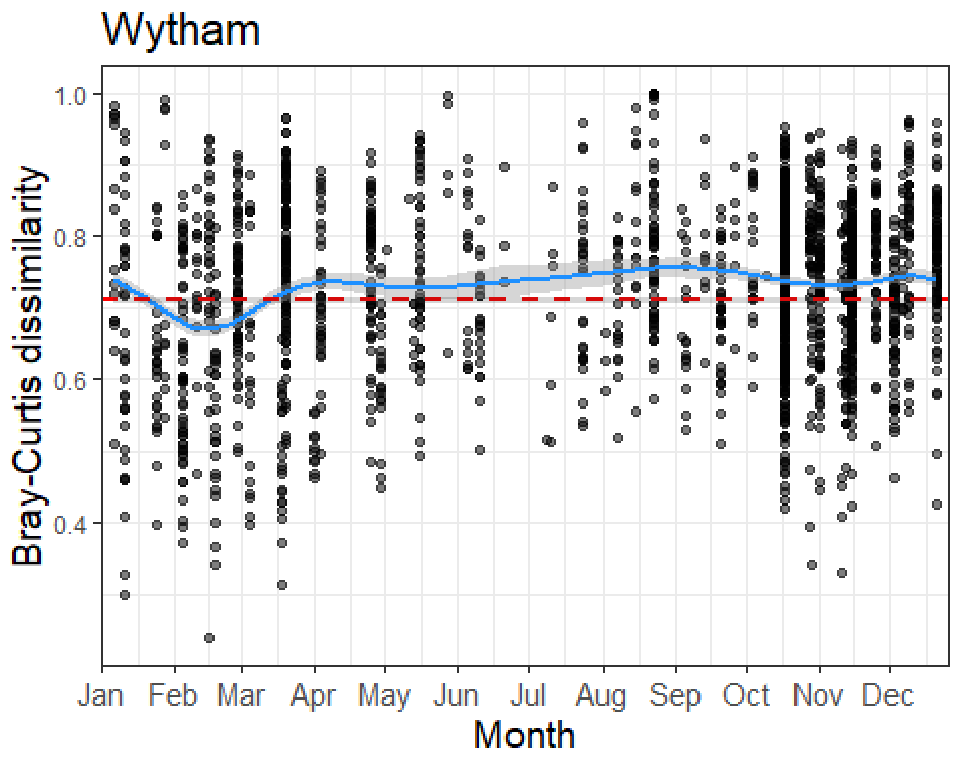

Overall within vs between individual BC boxplots

```
Dist_time_same_time$comparison <- "Same_time"
Dist_time_withind_2$comparison <- "Same_mouse"

Dist_merge_within_betwn <- rbind(Dist_time_same_time, Dist_time_withind_2)

p5 <- ggplot(Dist_merge_within_betwn, aes(x=comparison, y=value.y)) +
  geom_boxplot(notch = T, colour="dodgerblue", fill="dodgerblue", alpha=0.3)
+
  theme_bw() +
  xlab("Comparison") + ylab("Bray-Curtis dissimilarity") +
  ggtitle("Wytham") +
  scale_x_discrete(labels=c("Same mouse", "Same date")) +
  theme(axis.text.x=element_text(size=11),
        axis.title=element_text(size=12),
        title = element_text(size=14)) +
  ylim(0.125,1)
p5
```

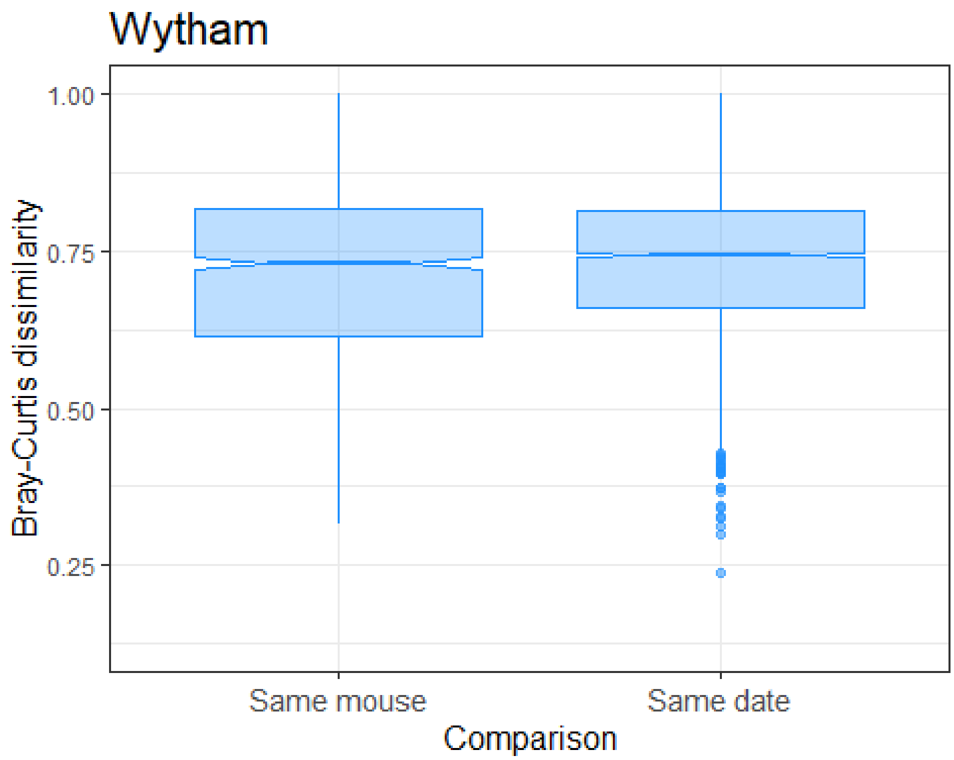

##Silwood

Prep data

```
#make continuous time since start variable
sample_data(ps.sil.ra)$Collection_date <-
as.Date(sample_data(ps.sil.ra)$date, format= "%d/%m/%Y")
sample_data(ps.sil.ra)$Time_0 <-
as.numeric(sample_data(ps.sil.ra)$Collection_date)
sample_data(ps.sil.ra)$Day_of_year <-
format(sample_data(ps.sil.ra)$Collection_date, "%j")
sample_data(ps.sil.ra)$Day_of_year <-
as.numeric(sample_data(ps.sil.ra)$Day_of_year)
sample_data(ps.sil.ra)$M_Y<-
paste(sample_data(ps.sil.ra)$month,sample_data(ps.sil.ra)$year,sep="/")
M <- as(sample_data(ps.sil.ra), 'data.frame')

#dissimilarity matrix (Bray-curtis)
D<- phyloseq::distance(ps.sil.ra, method = "bray", type = "samples")
D <- as.matrix(D)

#time difference matrix
#now make matrix of pairwise time differences using Time_0
Time <- as.matrix(dist(sample_data(ps.sil.ra)$Time_0), labels=TRUE,
dimnames=list(rownames(sample_data(ps.sil.ra)),
rownames(sample_data(ps.sil.ra))))
colnames(Time) <- rownames(Time) <- rownames(sample_data(ps.sil.ra))
```

```
#Now melt to long format and combine D and Time for each pair of sampels
Time_melt <- melt(Time)
D_melt <- melt(D)
Time_melt$Sample_pair <- paste(Time_melt$Var1, Time_melt$Var2, sep="-")
D_melt$Sample_pair <- paste(D_melt$Var1, D_melt$Var2, sep="-")

#want to make 2 new columns with corresponding pit tags to each sample
colnames(D_melt)[1] <- "Sequence_sample_code"
D_melt$PIT.A <- "NA"
D_melt$PIT.A = M[match(D_melt$Sequence_sample_code,
M$Sequence_sample_code),"ID"]
D_melt$Time0.A <- "NA"
D_melt$Time0.A = M[match(D_melt$Sequence_sample_code,
M$Sequence_sample_code),"Time_0"]
D_melt$M_Y.A <- "NA"
D_melt$M_Y.A = M[match(D_melt$Sequence_sample_code,
M$Sequence_sample_code),"M_Y"]
D_melt$month.A <- "NA"
D_melt$month.A = M[match(D_melt$Sequence_sample_code,
M$Sequence_sample_code),"month"]
D_melt$Collection_date.A <- "NA"
D_melt$Collection_date.A = M[match(D_melt$Sequence_sample_code,
M$Sequence_sample_code),"Collection_date"]
D_melt$DOY.A <- "NA"
D_melt$DOY.A = M[match(D_melt$Sequence_sample_code,
M$Sequence_sample_code),"Day_of_year"]
colnames(D_melt)[1] <- "Var1"
colnames(D_melt)[2] <- "Sequence_sample_code"
D_melt$PIT.B <- "NA"
D_melt$PIT.B = M[match(D_melt$Sequence_sample_code,
M$Sequence_sample_code),"ID"]
D_melt$Time0.B <- "NA"
D_melt$Time0.B = M[match(D_melt$Sequence_sample_code,
M$Sequence_sample_code),"Time_0"]
D_melt$M_Y.B <- "NA"
D_melt$M_Y.B = M[match(D_melt$Sequence_sample_code,
M$Sequence_sample_code),"M_Y"]
D_melt$month.B <- "NA"
D_melt$month.B = M[match(D_melt$Sequence_sample_code,
M$Sequence_sample_code),"month"]
D_melt$Collection_date.B <- "NA"
D_melt$Collection_date.B = M[match(D_melt$Sequence_sample_code,
M$Sequence_sample_code),"Collection_date"]
D_melt$DOY.B <- "NA"
D_melt$DOY.B = M[match(D_melt$Sequence_sample_code,
M$Sequence_sample_code),"Day_of_year"]
colnames(D_melt)[2] <- "Var2"

Distance_Time_merge <- merge(Time_melt, D_melt, by="Sample_pair")
head(Distance_Time_merge)
```

```
##   Sample_pair Var1.x Var2.x value.x Var1.y Var2.y   value.y PIT.A Time0.A
## 1   Sil1-Sil1   Sil1   Sil1       0   Sil1   Sil1 0.0000000  M708   16386
## 2  Sil1-Sil10   Sil1  Sil10     182   Sil1  Sil10 0.5111972  M708   16386
## 3 Sil1-Sil100   Sil1 Sil100     126   Sil1 Sil100 0.6591242  M708   16386
## 4 Sil1-Sil101   Sil1 Sil101      14   Sil1 Sil101 0.4697034  M708   16386
## 5 Sil1-Sil102   Sil1 Sil102       0   Sil1 Sil102 0.4536366  M708   16386
## 6 Sil1-Sil103   Sil1 Sil103      98   Sil1 Sil103 0.7012088  M708   16386
##         M_Y.A  month.A Collection_date.A DOY.A PIT.B Time0.B       M_Y.B
## 1 November/14 November        2014-11-12   316  M708   16386 November/14
## 2 November/14 November        2014-11-12   316  M842   16568      May/15
## 3 November/14 November        2014-11-12   316  M700   16512    March/15
## 4 November/14 November        2014-11-12   316  M842   16400 November/14
## 5 November/14 November        2014-11-12   316  M802   16386 November/14
## 6 November/14 November        2014-11-12   316  M886   16484 February/15
##    month.B Collection_date.B DOY.B
## 1 November        2014-11-12   316
## 2      May        2015-05-13   133
## 3    March        2015-03-18    77
## 4 November        2014-11-26   330
## 5 November        2014-11-12   316
## 6 February        2015-02-18    49
```

```
Distance_Time_merge <-
Distance_Time_merge[Distance_Time_merge$Var1.x!=Distance_Time_merge$Var2.x, ]
#save full BC / time dist data
#write.csv(Distance_Time_merge,
'~/Silwood_season_BC_time_dist_full_data.csv')
```

Within-individual similarity at different time lags

```
Dist_time_withind <-
Distance_Time_merge[Distance_Time_merge$PIT.A==Distance_Time_merge$PIT.B, ]
Dist_time_withind_2 <- subset(Dist_time_withind, value.x!=0)
#keep only mice caught 3 or more times
mouse_recaps <- as.data.frame(summary(as.factor(sample_data(ps.sil.ra)$ID),
maxsum = 300))
mouse_recaps_multi <- subset(mouse_recaps,
summary(as.factor(sample_data(ps.sil.ra)$ID), maxsum = 300)>=3)
mouse_recap_list <- as.list(rownames(mouse_recaps_multi))
Dist_time_withind_3 <- subset(Dist_time_withind_2, PIT.A%in%mouse_recap_list)
physeq_sil_recaps <- subset_samples(ps.sil.ra, ID%in%mouse_recap_list)

#run time-decay log linear model
#convert BC to similarity by 1-BC
summary(Dist_time_withind_3$value.x)#min=7 max=281 days between samples

##    Min. 1st Qu.  Median    Mean 3rd Qu.    Max.
##    7.00   29.00   63.00   74.22  105.00  281.00

Dist_time_withind_3$BC_sim = 1- Dist_time_withind_3$value.y
```

```r
mdl.2 <- lm(log(BC_sim+1) ~ value.x, data = Dist_time_withind_3)
summary(mdl.2)

##
## Call:
## lm(formula = log(BC_sim + 1) ~ value.x, data = Dist_time_withind_3)
##
## Residuals:
##      Min       1Q   Median       3Q      Max
## -0.26791 -0.06193  0.00397  0.07224  0.23057
##
## Coefficients:
##               Estimate Std. Error t value Pr(>|t|)
## (Intercept)  0.3365708  0.0049220  68.381  < 2e-16 ***
## value.x     -0.0002123  0.0000534  -3.976 7.51e-05 ***
## ---
## Signif. codes:  0 '***' 0.001 '**' 0.01 '*' 0.05 '.' 0.1 ' ' 1
##
## Residual standard error: 0.09184 on 988 degrees of freedom
## Multiple R-squared:  0.01575,    Adjusted R-squared:  0.01475
## F-statistic: 15.81 on 1 and 988 DF,  p-value: 7.512e-05

plot(mdl.2)
```

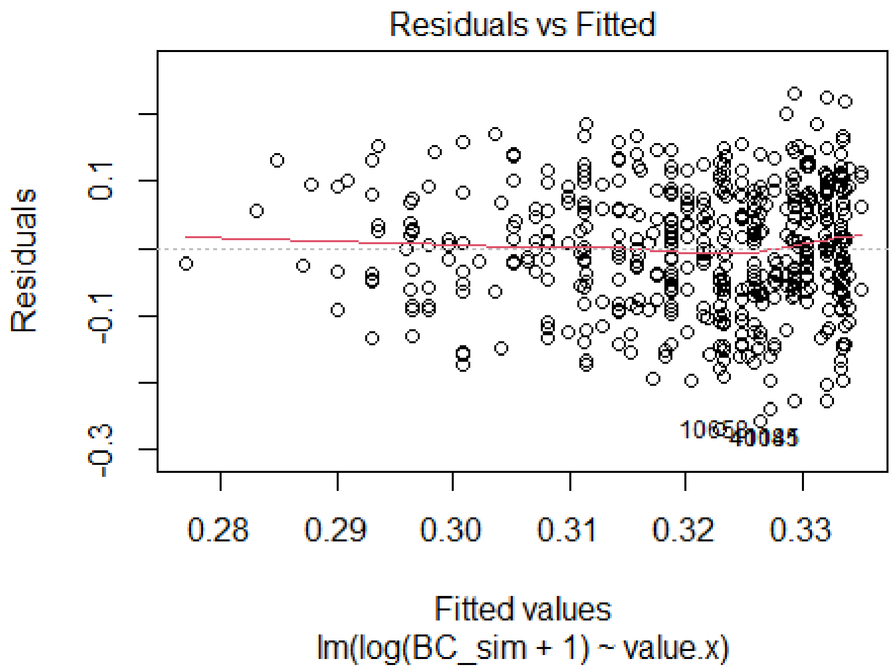
Residuals vs Fitted
Residuals
0.1
-0.1
-0.3
0.28
0.29
0.30
0.31
0.32
0.33
Fitted values
lm(log(BC_sim + 1) ~ value.x)

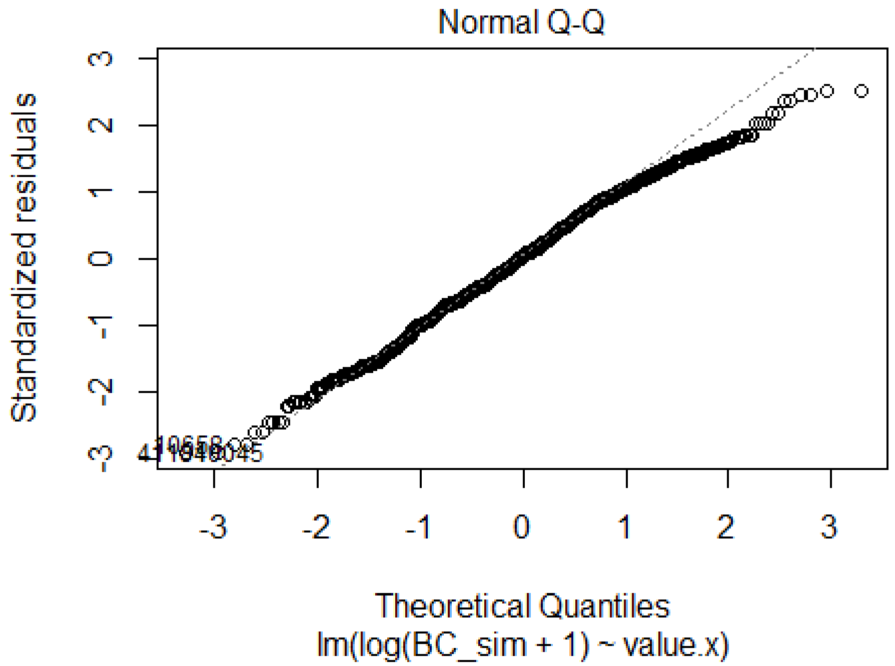
Normal Q-Q
Standardized residuals
3
2
1
0
-1
-2
-3
-3
-2
-1
0
1
2
3
Theoretical Quantiles
lm(log(BC_sim + 1) ~ value.x)

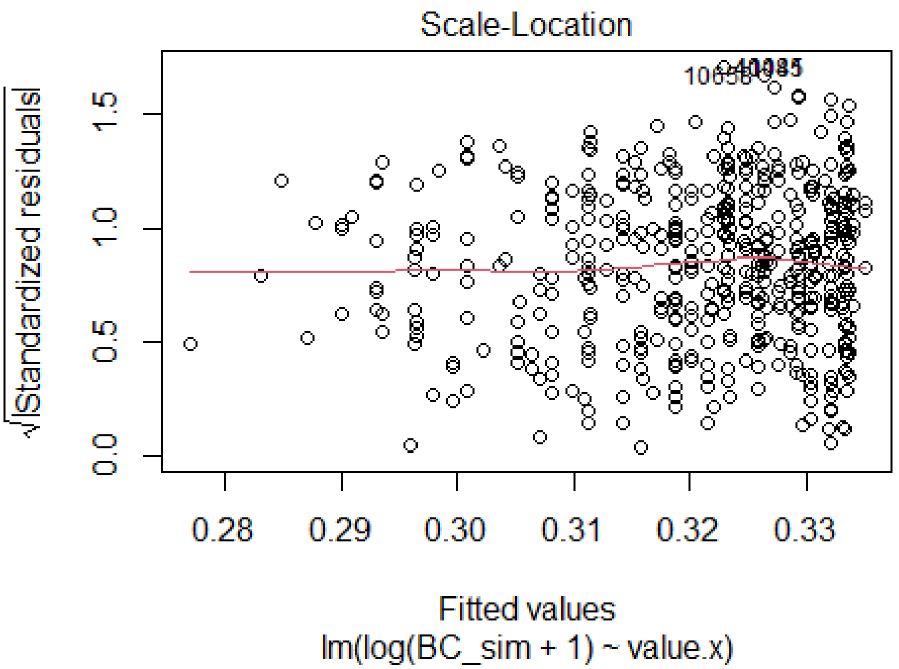

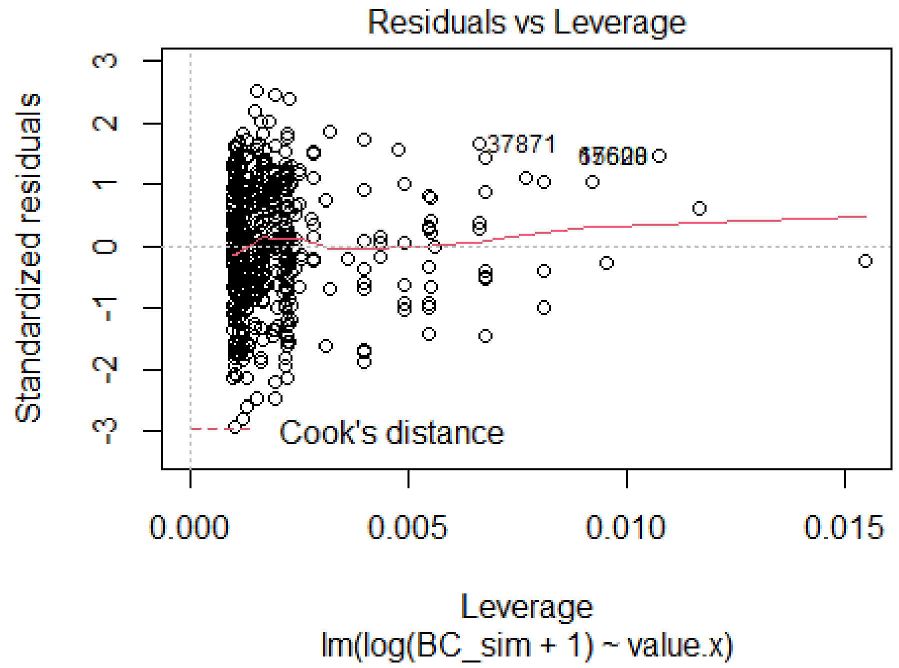


```
AIC(mdl.2)

## [1] -1914.269
```

```r
#use it for prediction
pdat <- expand.grid(value.x=seq(7,281,7))
pred <- predict (mdl.2, newdata = pdat, na.rm = T,
                 type= "response", se.fit = TRUE, level=0)
predframe <- data.frame (pdat, preds = pred$fit, se = pred$se.fit)

Dist_time_withind_sil <- Dist_time_withind_3
predframe_sil <- predframe

#plot fitted values with raw data
p3 <- ggplot(Dist_time_withind_sil, aes(x=value.x, y=log(BC_sim+1))) +
  geom_point(alpha=0.3) +
  geom_line(aes(y=preds), data=predframe_sil, colour="springgreen3", size=1)
+
  geom_ribbon(data = predframe_sil, aes(y = NULL, ymin = preds-se, ymax =
preds+se), alpha=0.15) +
  ylab("log((1- Bray-Curtis dissimilarity) +1)") +
  xlab("Number of days between sampling") +
  theme_bw() +
  theme(axis.text.x=element_text(size=11),axis.title=element_text(size=14),
        title = element_text(size=14)) +
  ggtitle("Silwood")
p3
```

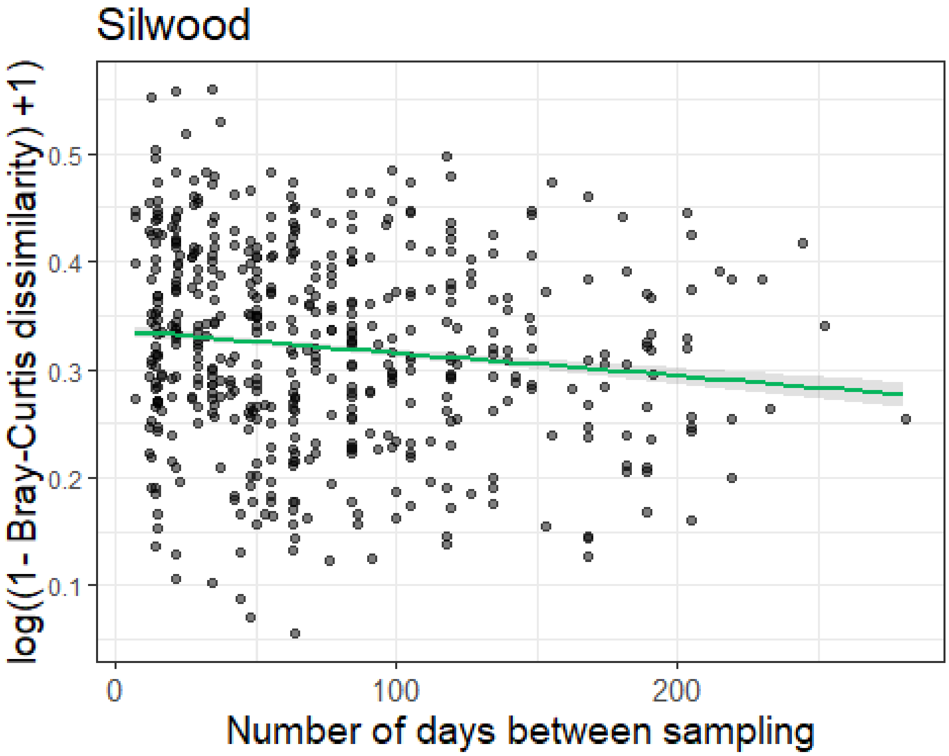


```r
#see how the rate of change over time changes voer different timescales
Dist_time_withind_3$rate_change = Dist_time_withind_3$BC_sim /
Dist_time_withind_3$value.x
```

```
# summary(Dist_time_withind_3$value.x) #min=9 max=252
#look at average rate of change within individuals over different timescales;
<30 , 30<x>90, 90<x>180, 180<x>365

#within 1 month
summary(subset(Dist_time_withind_3, value.x<30)$rate_change)

##     Min.  1st Qu.   Median     Mean  3rd Qu.     Max.
## 0.005333 0.017563 0.021707 0.024458 0.028503 0.080675

sd(subset(Dist_time_withind_3, value.x<30)$rate_change)

## [1] 0.01235575

length(subset(Dist_time_withind_3, value.x<30)$rate_change)

## [1] 258

#between 1 and 3 months
summary(subset(Dist_time_withind_3, value.x>=30 & value.x<90)$rate_change)

##      Min.   1st Qu.    Median      Mean   3rd Qu.      Max.
## 0.0008846 0.0045632 0.0063022 0.0071065 0.0089430 0.0220747

sd(subset(Dist_time_withind_3, value.x>=30 & value.x<90)$rate_change)

## [1] 0.003630514

length(subset(Dist_time_withind_3, value.x>=30 & value.x<90)$rate_change)

## [1] 418

#between 3 and 6 months
summary(subset(Dist_time_withind_3, value.x>=90 & value.x<180)$rate_change)

##      Min.   1st Qu.    Median      Mean   3rd Qu.      Max.
## 0.0008098 0.0022458 0.0029610 0.0031545 0.0038094 0.0065490

sd(subset(Dist_time_withind_3, value.x>=90 & value.x<180)$rate_change)

## [1] 0.001234632

length(subset(Dist_time_withind_3, value.x>=90 & value.x<180)$rate_change)

## [1] 242

#between 6 months - 1 year
summary(subset(Dist_time_withind_3, value.x>=180 & value.x<365)$rate_change)

##      Min.   1st Qu.    Median      Mean   3rd Qu.      Max.
## 0.0008453 0.0013171 0.0018322 0.0017727 0.0021278 0.0030832

sd(subset(Dist_time_withind_3, value.x>=180 & value.x<365)$rate_change)
```

```
## [1] 0.000550525

length(subset(Dist_time_withind_3, value.x>=180 & value.x<365)$rate_change)

## [1] 72
```

Between-individual variation per month

```
#are individuals more similar at certain times of year?
Dist_time_same_time <-
Distance_Time_merge[Distance_Time_merge$M_Y.A==Distance_Time_merge$M_Y.B, ]
Dist_time_same_time <-
Dist_time_same_time[Dist_time_same_time$PIT.A!=Dist_time_same_time$PIT.B, ]
Dist_time_same_time$M_Y.A <- as.factor(Dist_time_same_time$M_Y.A)
level_order <- c("January", "February", "March", "April", "May", "June",
"July", "August", "September", "October", "November", "December")

#BC per trap date
# add average within-individual BC as a dashed reference line
mean(Dist_time_withind_2$value.y)

## [1] 0.6163654

sd(Dist_time_withind_2$value.y)

## [1] 0.1274039

se = sd(Dist_time_withind_2$value.y)/ sqrt(nrow(Dist_time_withind_2))
se

## [1] 0.003981371

Dist_time_same_time2 <-
Distance_Time_merge[Distance_Time_merge$Collection_date.A==Distance_Time_merg
e$Collection_date.B, ]

p4b <- ggplot(Dist_time_same_time2, aes(x=DOY.A, y=value.y)) +
  geom_point( alpha=0.3) +
  geom_smooth(method="gam", formula = y ~ s(x, bs = "cc"),
colour="springgreen3") +
  geom_hline(yintercept = 0.612938, colour="red", size=1, linetype=2) +
  geom_ribbon(aes(ymin=0.612938-se, ymax=0.612938+se), alpha=0.2) +
  ggtitle("Silwood") +
  #facet_wrap(~Year.B) +
  ylab("Bray-Curtis dissimilarity") +
  xlab("Month") +
  theme_bw() +
  theme(axis.text.x=element_text( size=11),axis.title=element_text(size=14),
        title = element_text(size=14)) +
  scale_x_continuous(breaks=cumsum(c(15,31,28,31,30,31,30,31,31,30,31,30)),
                        labels
=c("Jan","Feb","Mar","Apr","May","Jun","Jul","Aug","Sep","Oct","Nov","Dec"),
```

```
                         expand = c(0.015,0.015))
p4b
```

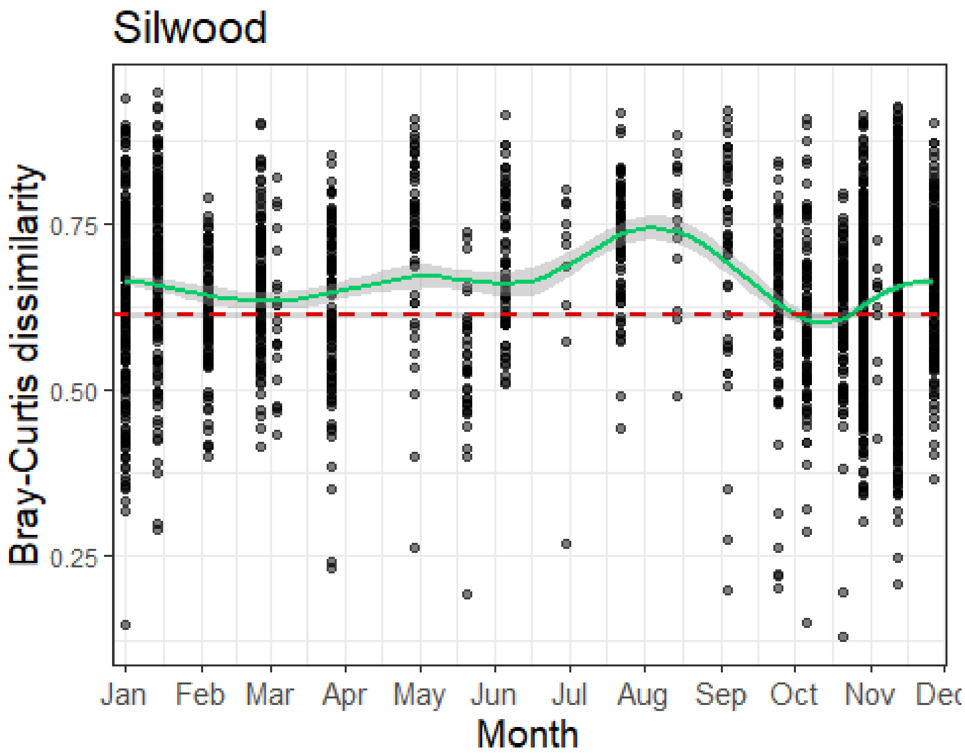

Overall within vs. between individual BC

```
Dist_time_same_time$comparison <- "Same_time"
Dist_time_withind_2$comparison <- "Same_mouse"
Dist_merge_within_betwn <- rbind(Dist_time_same_time, Dist_time_withind_2)

p6 <- ggplot(Dist_merge_within_betwn, aes(x=comparison, y=value.y)) +
  geom_boxplot(notch = T, colour="springgreen3", fill="springgreen3",
alpha=0.3) +
  theme_bw() +
  xlab("Comparison") + ylab("Bray-Curtis dissimilarity") +
  ggtitle("Silwood") +
  scale_x_discrete(labels=c("Same mouse", "Same date")) +
  theme(axis.text.x=element_text(size=11),
        axis.title=element_text(size=12),
        title = element_text(size=14)) +
  ylim(0.125,1)
p6
```

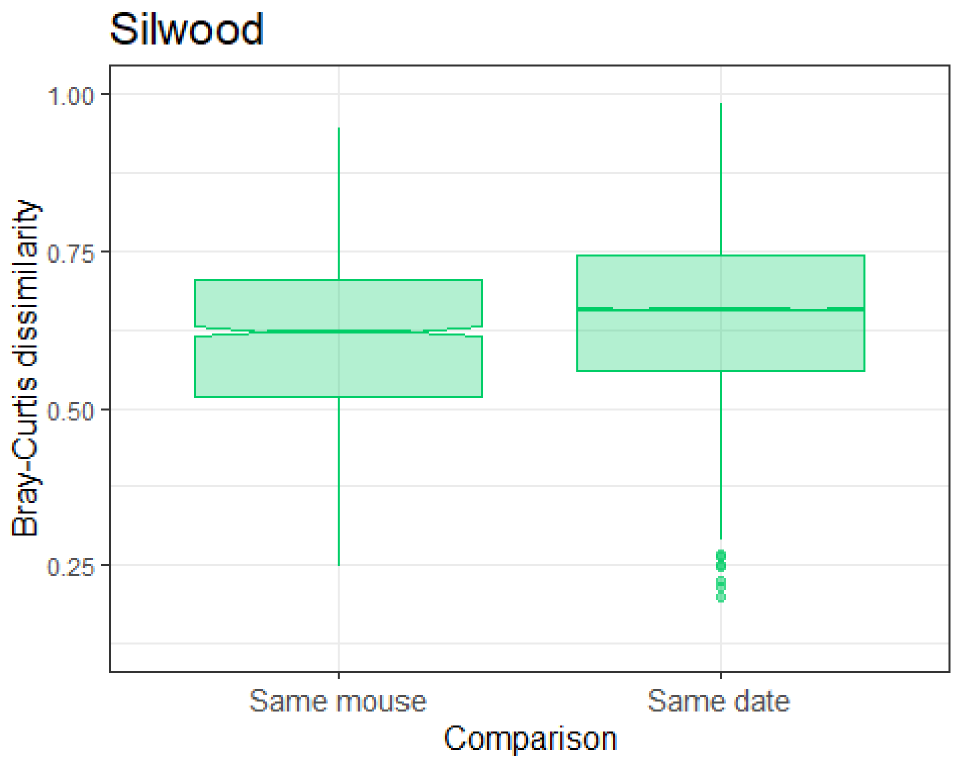

Plot all together

```
library(gridExtra)
#make y axes on same scale and get rid of labels for silwood
#summary(log(Dist_time_withind_3$BC_sim +1)) #min=0.05519, max=0.55959
p1<- p1+ylim(0,0.6)
p3<- p3+ylim(0,0.6)
p2b <- p2b + ylim(0.125,1)
p4b <- p4b + ylim(0.125,1)

grid.arrange(p1,p3,p2b, p4b, p5, p6, ncol=2)
```

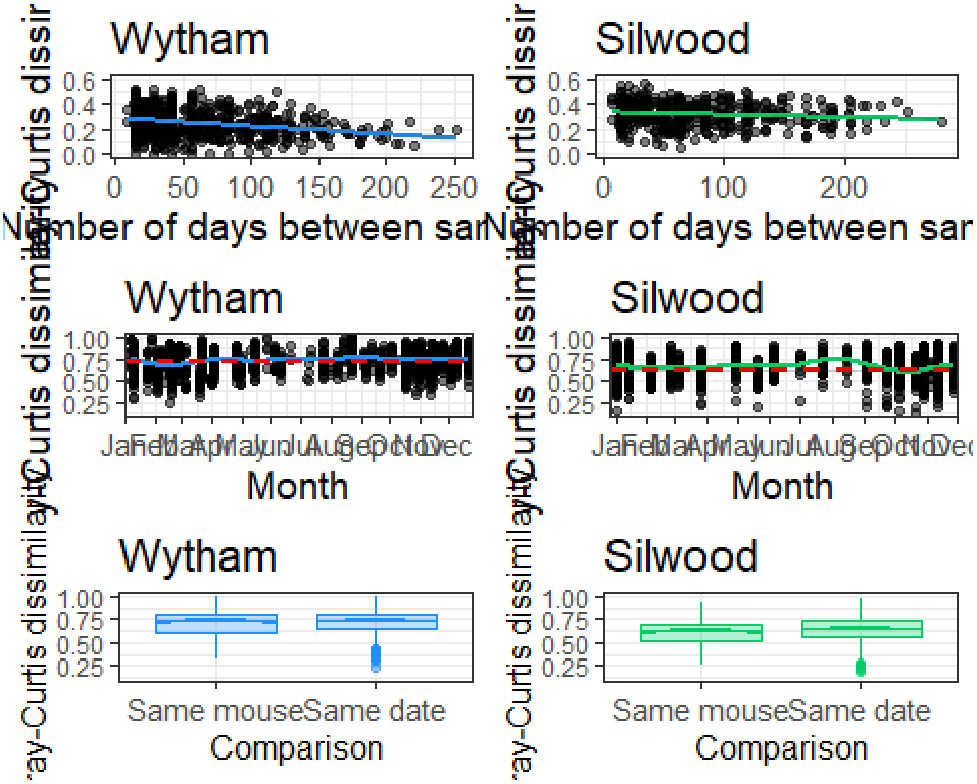

Wytham
Silwood
Number of days between sar
Month
Comparison
Same mouse
Same date
Jan Feb Mar Apr May Jun Jul Aug Sep Oct Nov Dec
